# Encompassing new use cases - level 3.0 of the HUPO-PSI format for molecular interactions

**DOI:** 10.1186/s12859-018-2118-1

**Published:** 2018-04-11

**Authors:** M. Sivade (Dumousseau), D. Alonso-López, M. Ammari, G. Bradley, N. H. Campbell, A. Ceol, G. Cesareni, C. Combe, J. De Las Rivas, N. del-Toro, J. Heimbach, H. Hermjakob, I. Jurisica, M. Koch, L. Licata, R. C. Lovering, D. J. Lynn, B. H. M. Meldal, G. Micklem, S. Panni, P. Porras, S. Ricard-Blum, B. Roechert, L. Salwinski, A. Shrivastava, J. Sullivan, N. Thierry-Mieg, Y. Yehudi, K. Van Roey, S. Orchard

**Affiliations:** 1 European Bioinformatics Institute (EMBL-EBI), European Molecular Biology Laboratory, Wellcome Genome Campus, Hinxton, CB10 1SD UK; 2 Cancer Research Center (CiC-IBMCC, CSIC/USAL/IBSAL), Consejo Superior de Investigaciones Científicas (CSIC) and Universidad de Salamanca (USAL), 37007 Salamanca, Spain; 3 School of Animal and Comparative Biomedical Sciences, University of Arizona, Tucson, USA; 4 Target Sciences, GSK, Stevenage, UK; 5 Institute of Cardiovascular Science, University College London, Rayne Building, 5 University Street, London, WC1E 6JF UK; 6 Center for Genomic Science of IIT@SEMM, Fondazione Istituto Italiano di Tecnologia (IIT), Via Adamello 16, I-20139 Milan, Italy; 7 Department of Biology, University of Rome Tor Vergata, Via della Ricerca Scientifica, Rome, Italy; 8 Wellcome Trust Centre for Cell Biology, School of Biological Sciences, University of Edinburgh, Edinburgh, EH9 3BF UK; 9 Cambridge Systems Biology Centre, University of Cambridge, Cambridge, UK; 10 Department of Genetics, University of Cambridge, Cambridge, UK; 11 State Key Laboratory of Proteomics, Beijing Proteome Research Center, Beijing Institute of Radiation Medicine, National Center for Protein Sciences (The PHOENIX Center, Beijing), Beijing, China; 12 Krembil Research Institute, University Health Network, Toronto, ON M5T 2S8 Canada; 13 Departments of Medical Biophysics and Computer Science, University of Toronto, Toronto, ON Canada; 14 EMBL Australia Group, South Australian Health and Medical Research Institute, Adelaide, Australia; 15 School of Medicine, Flinders University, Bedford Park, Adelaide, Australia; 16 Department of Biology, Ecology and Earth Sciences, Università della Calabria, Rende, Italy; 17 Univ Lyon, University Claude Bernard Lyon 1, INSA Lyon, CPE, Institute of Molecular and Supramolecular Chemistry and Biochemistry (ICBMS), UMR 5246, F-69622 Villeurbanne, France; 18 SIB Swiss Institute of Bioinformatics, Centre Medical Universitaire, 1 rue Michel Servet, 1211 Geneva 4, Switzerland; 19 UCLA-DOE Institute for Genomics and Proteomics, Los Angeles, USA; 20 TIMC-IMAG, CNRS, Univ. Grenoble Alpes, F-38000 Grenoble, France; 21 Structural and Computational Biology Unit, European Molecular Biology Laboratory (EMBL), Meyerhofstrasse 1, D-69117 Heidelberg, Germany

**Keywords:** Molecular interactions, Protein-protein interaction, Protein complexes, Data standards, XML, HUPO-PSI, PSI-MI

## Abstract

**Background:**

Systems biologists study interaction data to understand the behaviour of whole cell systems, and their environment, at a molecular level. In order to effectively achieve this goal, it is critical that researchers have high quality interaction datasets available to them, in a standard data format, and also a suite of tools with which to analyse such data and form experimentally testable hypotheses from them. The PSI-MI XML standard interchange format was initially published in 2004, and expanded in 2007 to enable the download and interchange of molecular interaction data. PSI-XML2.5 was designed to describe experimental data and to date has fulfilled this basic requirement. However, new use cases have arisen that the format cannot properly accommodate. These include data abstracted from more than one publication such as allosteric/cooperative interactions and protein complexes, dynamic interactions and the need to link kinetic and affinity data to specific mutational changes.

**Results:**

The Molecular Interaction workgroup of the HUPO-PSI has extended the existing, well-used XML interchange format for molecular interaction data to meet new use cases and enable the capture of new data types, following extensive community consultation. PSI-MI XML3.0 expands the capabilities of the format beyond simple experimental data, with a concomitant update of the tool suite which serves this format. The format has been implemented by key data producers such as the International Molecular Exchange (IMEx) Consortium of protein interaction databases and the Complex Portal.

**Conclusions:**

PSI-MI XML3.0 has been developed by the data producers, data users, tool developers and database providers who constitute the PSI-MI workgroup. This group now actively supports PSI-MI XML2.5 as the main interchange format for experimental data, PSI-MI XML3.0 which additionally handles more complex data types, and the simpler, tab-delimited MITAB2.5, 2.6 and 2.7 for rapid parsing and download**.**

**Electronic supplementary material:**

The online version of this article (10.1186/s12859-018-2118-1) contains supplementary material, which is available to authorized users.

## Background

Understanding the interaction networks that govern biological systems is essential to fully decipher the molecular mechanisms ensuring cellular biology and tissue homeostasis. Interactions between molecules result in both the assembly of stable functional protein complexes, which form the molecular machinery of the cell, and transient, often regulatory, networks of weakly associating molecules. Together these drive and regulate cellular processes, cell-cell interactions and cell-matrix interactions. The capture and curation of published interaction data has been the work of interaction databases for many years, and many of these resources have collaborated through the Molecular Interaction workgroup of the Human Proteome Organization Proteomics Standards Initiative (HUPO-PSI) to create and maintain community data formats and standards [1]. These formats and standards have enabled the systematic capture, reuse and exchange of these data and the building of tools to enable network contextualization and analysis of -omics data.

Version 1.0 of PSI-MI XML was published in 2004 and enabled the description of simple protein interaction data [2]. The format was widely implemented and supported by both software tool developers and data providers, but was soon found to be too limited in its scope. To facilitate rich, integrative analyses, many databases wished to describe and exchange the full wealth of data generated by interaction experiments, including a detailed description of experimental conditions and features such as binding sites or affinity tags on participating molecules. In order to make this possible, the Molecular Interactions working group of the HUPO-PSI further extended the XML schema to enable the annotation of a wider range of data. PSI-MI XML2.5 expanded the type of interactors to encompass any molecule or complex of molecules which can be described in the ‘interactor type’ branch of the accompanying controlled vocabulary (PSI-MI CV) [3]. Sequence or positional features on a *participant* molecule that are relevant for the interaction can be described in a *featureList*, again using an appropriate controlled vocabulary term*.* The PSI-MI XML2.5 schema allows two different representations of interactions. The compact format was designed for larger datasets. In this, the repetitive elements of a larger set of interactions, such as the interactors and experiments, are only described once, in the respective list elements, and subsequently referred to. The extended format groups all related data closely together and was designed to simplify parsing. This version of the schema also supports the hierarchical build-up of complexes from component sub-complexes.

Version 2.5 has proven to be, and will continue to be, capable of capturing the vast majority of molecular interaction data, generated by techniques such as protein complementation assays, affinity capture, biophysical measurements and enzyme assays. It successfully describes genetic as well as physical interactions, and can also be used to hold predicted interactions or the results of text-mining exercises, all clearly described as such by appropriate controlled vocabulary terms. Consequently, this version of the format will continue to be supported by the PSI-MI community for the foreseeable future. However, use cases have arisen which cannot be adequately described within this XML schema, and in 2013 it was decided that the field had advanced sufficiently to justify moving to the next level in this deliberately tiered approached to describing interaction data, and to produce PSI-MI XML3.0.

## Implementation

A community standard will only remain of use to that community if it meets the needs of current and future users, and if these users have bought into, and contributed to, the update process. Prior to creating any changes in the schema, a questionnaire was sent out to known users of the format to establish how PSI-MI XML2.5 was currently being utilised, and to identify cases in which the format was not meeting user needs. Once an initial list of requirements had been established, use cases and examples of each were collated. Initial proposals or, in some cases, multiple proposals for tackling each case were drawn up and circulated to mailing lists and known format users. Each proposal, and any subsequent feedback, was then discussed in detail at the 2014 HUPO-PSI meeting by attendees to the MI work track [4]. The final list of use cases was agreed upon and the changes to PSI-MI XML2.5 described below approved and subsequently implemented. Additional file 1 contains an example file showing the representation of the molecular interaction data from a single publication in PSI-MI XML3.0.

### Enhancements to the description of molecule features

In PSI-MI XML 2.5 the *featureList* element describes the sequence features of the *participant* that are relevant to the interaction, using the appropriate term or terms from the corresponding controlled vocabulary, for example ‘sufficient binding region’ (MI:0442) or experimental modifications such as ‘green fluorescent protein tag’ (MI:0367) linked from the *featureType* element. The *featureRangeList* describes the location of a feature on the participant sequence. In PSI-MI XML3.0 a series of changes, listed below, have been implemented to enable more details to be added to the description of a feature.

**Fig. 1:**
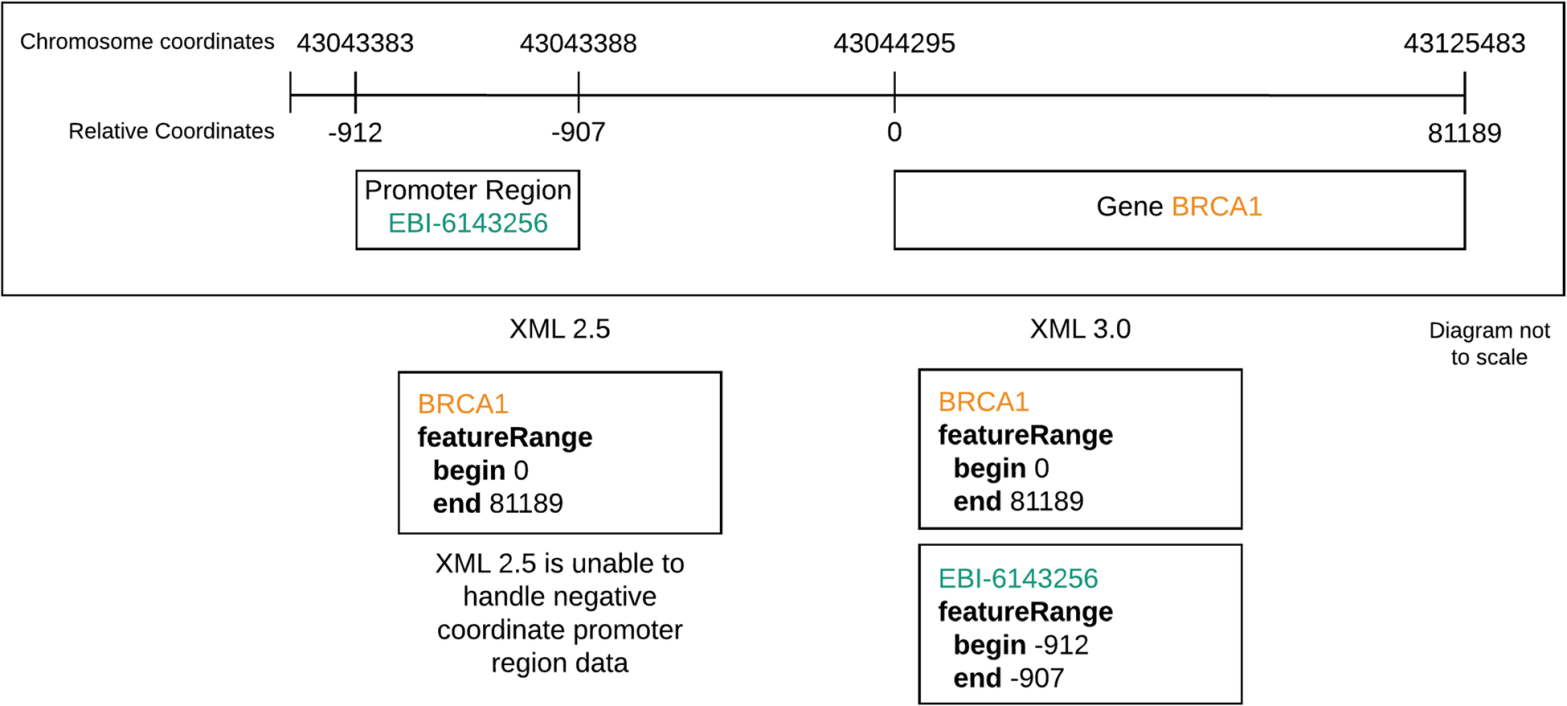
The *position* attribute type and *interval* attribute type for *featureRange* have been updated to enable the description of negative values, thus allowing the full description of gene coordinates

**Fig. 2:**
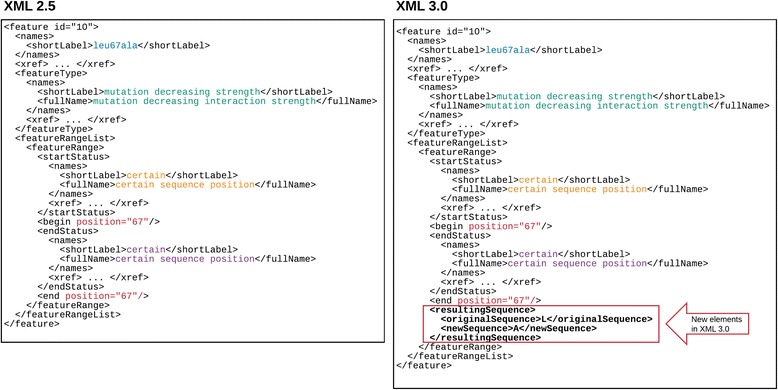
The position, effect of a mutation and now also the new sequence replacing the original sequence in a site-directed mutation can be systematically captured using the *featureRange* positions, the *featureType* element and a new element named *resultingSequence* added at the level of the *featureRange* element

**Fig. 3:**
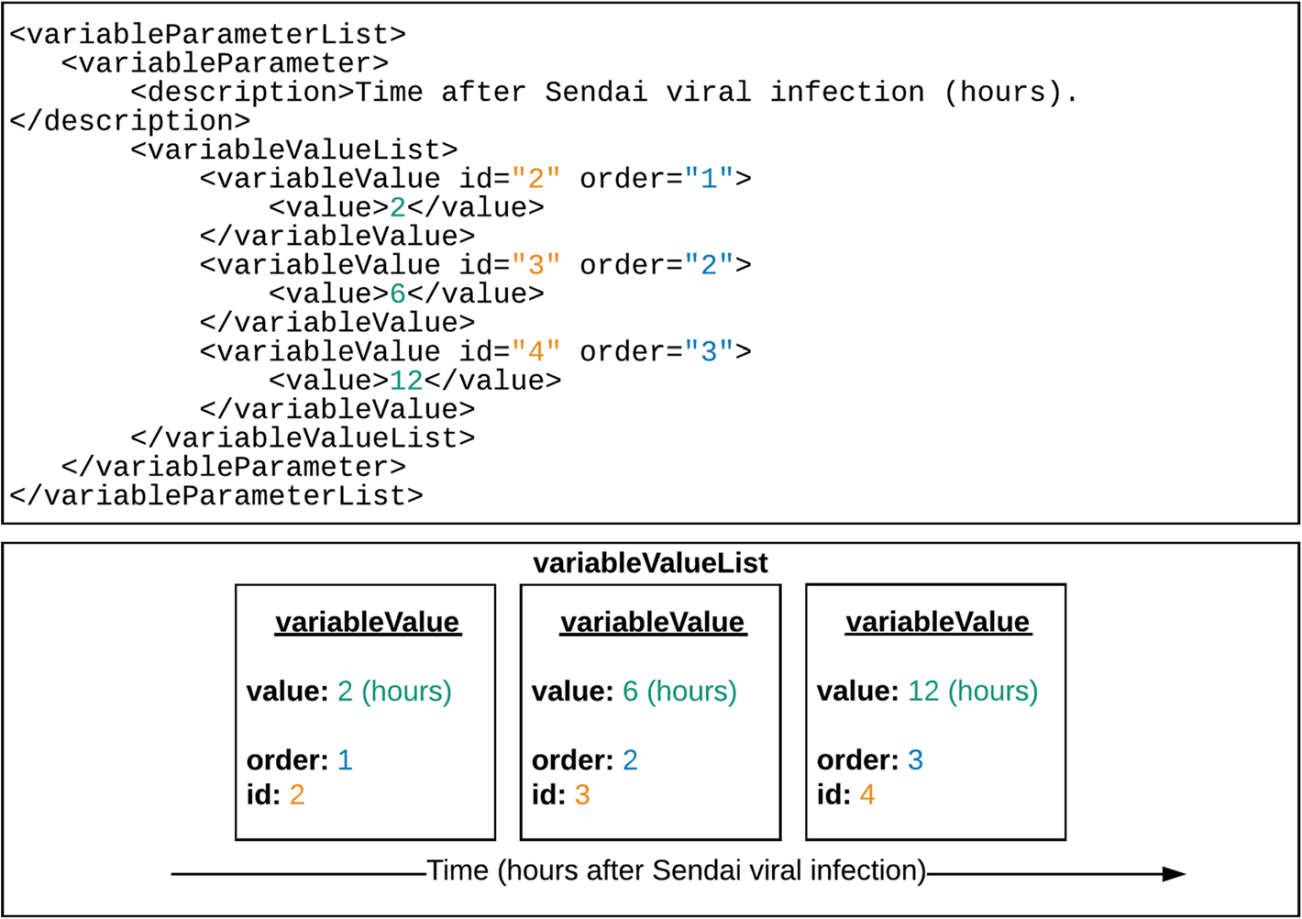
Dynamic interactions resulting from a progressive change in the experimental environment can be described using a *variableParameterList* element added to the experiment element, which contains one-to-many *variableParameter* elements

- The *position* attribute type and *interval* attribute type for *featureRange* have been updated. In PSI-MI XML2.5 these are of the type ‘*unsignedLong*’, which means that features described in this version can only have positive range positions. This has been updated to ‘*long*’ in PSI-MI XML3.0 to enable negative positions, for example designated gene promoter regions, to be captured (Fig. 1, Additional file 2)**.**
- The position and effect of a mutation can be systematically captured using the *featureRange* positions and the *featureType* element. However, in PSI-MI XML2.5 there is no defined way to capture the actual sequence change. In PSI-MI XML3.0, a new element named *resultingSequence* has been added at the level of the *featureRange* element (Fig. 2, Additional file 3). The *resultingSequence* element contains an *originalSequence* element to describe the original sequence, a *newSequence* element which contains the mutated sequence and an *xref* element, which would be optional, and could be used to add external cross references such as Ensembl cross references to single nucleotide polymorphisms (SNPs). The *newSequence* and *originalSequence* are not required if an *xref* element is provided.
- It is now possible to add several feature detection methods in the *feature* element by making the *featureDetectionMethod* element repeatable in the *feature* element (Additional file 4). This will enable users to describe cases in which a feature has been recognized by more than one method, for example a post-translational modification (PTM) being identified by both a specific antibody and by mass spectrometry. The change was made to maintain backwards compatibility with earlier versions of the schema, a goal that was set by the work group when version 1.0 was published. When several feature detection methods are described in a file, most existing parsers will simply use the last feature detection method they have parsed.
- The *feature* element has been extended in PSI-MI XML3.0 to capture the dependency of an interaction on a particular feature, for example the presence of a specific PTM and also the effect of an interaction, such as the phosphorylation of a tyrosine residue by a protein kinase. In PSI-XML 2.5 this information is stored as an attribute of a feature. An optional *featureRole* element has been added to the *feature* element, which can be used to describe PTMs existing in/resulting from the context of the interaction. This element would be populated from a list of new controlled vocabulary terms added to the PSI-MI ontology, such as ‘prerequisite-PTM (MI:0638)’ or ‘observed-PTM (MI:0925)’.
- The equilibrium dissociation constant or parameters, such as k_on_ or k_off_ can be added at the interaction level in PSI-MI XML2.5; however, this does not enable the systematic capture of changes in this parameter when a sequence is mutated at the feature level. The kinetic and the equilibrium dissociation constant parameters that are linked to a specific mutation have been moved from interaction *parameterList* to the feature *parameterList* (Fig. 3, Additional file 5). However, the kinetic and the equilibrium dissociation constant parameters associated with the wild type protein will still be at the interaction level in PSI-MI XML3.0.

### Description of New data types

The use of controlled vocabulary terms to populate both the XML and the accompanying tab-delimited schemas has proven to be an effective way of enabling the capture of data generated by novel techniques without a need to update the data format. However, the type of information generated by these techniques, or increasingly assembled from evidence generated by multiple techniques, is becoming more complex. The XML format has therefore been adapted to accommodate new types of information, either derived from a single, multi-faceted experiment or by combining the results of multiple investigations.

- Dynamic interactions: interaction sub-networks may be rewired in response to changes in the environmental conditions in which the experiment is performed. Examples of such changes include applying increasing concentration of an agonist onto a cell or a single concentration for an increasing amount of time, or merely sampling the interactome at different stages of the cell cycle. In PSI-MI XML3.0 an optional *variableParameterList* element has been added to the experiment element, which contains one-to-many *variableParameter* elements. Each *variableParameter* element contains the required *description* element to define the variable condition, an optional *unit* element to describe the unit of the different parameters in the *variableValueList* and a required *variableValueList* element to list all the existing variable parameter values used in the experiment. A *variableValueList* contains one-to-many *variableValue* elements, which may themselves contain an optional *order* attribute, an integer defining the position of the given *variableValue* within its containing *variableValueList* parent element (Fig. 3, Additional file 6). The format can also handle multiple changes in condition, such as parallel time courses of an increasing concentration of an agonist. The example given in Additional file 4 shows the changing profile of proteins that interact with STAT6 as the number of hours post-Sendai viral infection increases.
- Abstracted interactions: The PSI-XML2.5 schema was designed to represent experimental interactions, therefore an experiment description is required for each interaction. However, groups are increasingly looking to capture and exchange data collated from several publications. Examples of these include reference protein complexes described in the Complex Portal (www.ebi.ac.uk/complexportal, Additional file 7) [5] and the descriptions of cooperative binding when distinct molecular interactions influence each other either positively or negatively (Additional file 8). A version of the XML2.5 schema (PSI-PAR) was created to describe the production of protein binders such as antibodies, including detail such as antibody cross-reactivity – data that also cannot be described by a single experiment, and often not even in a single publication [6]. In order to describe such cases, the ‘*interactionDetectionMethod*’ element within an ‘*experimentDescription*’ element does not have a specific method assigned as a value in entries in the PSI-XML 2.5 format. Instead the CV terms ‘inferred by author’ (MI:0363) or ‘inferred by curator’ (MI:0364) are used to indicate that the interaction was inferred from multiple experiments or from several publications, respectively. Within the ‘*experimentDescription*’ element, the ‘*bibref*’ element refers to a related publication. In PSI-MI XML3.0, a new optional *abstractInteraction* element has been added within the *interactionList*. This element can now be used to describe ‘abstract’ or ‘modelled’ interactions such as stable complexes or allosteric interactions. This element contains many optional elements, for example a *participantList, bindingFeaturesList,* an *interactorType* element to describe the type, such as a protein complex, a protein-RNA or an antibody-antigen complex and an *interactionType* element to differentiate between a stable or transient complex, a cooperative interaction, or an enzymatic reaction.
  PSI-PAR was designed to fulfil three anticipated use cases: 1) affinity reagent and target protein production data, 2) characterisation/quality control results, and 3) complete summaries of end products. In practice, there has been no requirement for the format to exchange reagent and target production data. The ability to describe abstracted data in PSI-MI XML3.0 format fulfils use cases 2 and 3, by enabling the capture of quality control and reagent specificity data which are rarely described in a single publication. It has therefore been decided to merge PSI-PAR back into the parent PSI-MI XML, and XML3.0 will be regarded as the standard format for exchanging binder-target data from this point onwards. The PAR CV which was created to populate PSI-PAR will be merged back into the PSI-MI CV, thus minimising both schema and CV maintenance overheads.
- Co-operative interactions: in a cellular and tissue context, interactions between biomolecules are rarely independent. Instead, distinct molecular binding events affect each other positively or negatively, i.e. they are cooperative [7]. The two main mechanisms underlying cooperative binding are allostery and pre-assembly [8, 9]. Allostery involves a change in binding or catalytic properties of a biomolecule at one site of the molecule by an event at a different distinct site of the same molecule [10, 11]. Pre-assembly involves the generation or abrogation of a binding site through an interaction or enzymatic modification [12–14]. This includes (i) complex assembly resulting in the formation of a continuous binding site spanning multiple subunits; (ii) competitive binding to overlapping or adjacent, mutually exclusive binding sites; (iii) enzymatic modification that changes the physicochemical compatibility for a binding partner; or (iv) configurational pre-organization involving multivalent ligands that engage in multiple discrete interactions with one or more binding partners for high-avidity binding.
  As cooperative binding is common between many molecules in vivo, and the number of experimentally validated, interdependent interactions reported in the literature is increasing, it should be possible to represent and exchange these data in a standard format. Previously, however, cooperativity was only captured by the PSI-MI XML2.5 format by using annotations at the interaction level [15]. This has several shortcomings, including difficulties with parsing and automatic validation, repetition and redundancy, and lack of experimental details [15]. Because the data required to describe cooperative interactions rarely comes from a single experiment, or may even need to be assembled from many distinct publications, they are treated as abstract interactions and in PSI-MI XML3.0, captured using the *abstractInteraction* element. Within this element, an optional *cooperativeEffectList* allows listing the cooperative effects a specific interaction has on one or more other interactions. The effect will be described in the *allostery* or *preassembly* child element, as appropriate. Within these elements, additional details are captured, including the experimental methods and publications from which the data were inferred, references to the interactions that are affected, and the outcome of the effect.

### Description of new molecule types

Molecule sets: PSI-MI XML2.5 contains a key element *interactorType*, to describe the type of molecule involved in an interaction. This qualifies an interactor with a term from the PSI-MI controlled vocabulary, for example ‘protein’ (MI:0326) or ‘polysaccharide’ (MI:0904). However, there are cases when the exact molecule cannot be described, where it may be one of several possible entities. Examples of such cases include a peptide identified as the result of a mass spectrometry experiment which can be redundantly assigned to any one of a family or closely related molecules, and a non-specific antibody which cannot distinguish between two proteins with a high degree of sequence homology. There are cases when the products of one or more genes cannot be distinguished at the protein level, for example human calmodulin is an identical protein produced by three genes (CALM1, CALM2, CALM3). In these cases it may be necessary to describe a ‘set’ of molecules. This is not a new concept – it has been common practice in pathway databases such as Reactome [16] for some years, and indeed the required CV terms have been taken from the Reactome definition. However, this cannot be a simple addition to the Participant type CV as the ability to add a feature to a specific molecule within that set may be necessary. In PSI-MI XML3.0, the *participant* element will now contain a choice between *interactor*, *interactorRef*, *interactionRef and interactorCandidateList*.The *interactorCandidateList* element would contain a *moleculeSetType* element (PSI-MI CV Type) followed by one to many *interactorCandidate* elements. The *interactorCandidate* node contains a required *id* attribute, a required *interactor* or *interactorRef* element to describe or reference an interactor and an optional *featureList* element with one to many features to describe binding features for each interactor candidate (Additional file 9).

### Additional updates

A number of minor updates were included, which improved the representation of aspects of a molecular interaction that can be described within the XML schema.

- Stoichiometry: in PSI-MI XML2.5 the stoichiometry of a molecule can only be described as free-text annotation or as an attribute of the participant. In PSI-MI XML3.0 the *participant* element has been updated to add an optional XML Schema Development (XSD) choice sub-element, which provides a choice between a *stoichiometry* element to describe the mean stoichiometry for this participant and a *stoichiometryRange* element to describe a stoichiometry range for this participant. If the *stoichiometry* element is selected, a value attribute is required to describe the stoichiometry as a decimal value. If the s*toichiometryRange* element is chosen, both *minValue* and *maxValue* attributes are required to describe the stoichiometry range as decimal values (Additional file 10).
- Update of the *bibref* element: the *bibref* element refers to a publication. PSI-MI XML2.5 allows either a cross reference (xref) element (to describe PubMed primary reference if it exists) or an *attributeList* element (to describe publication details such as publication title and publication date). To export both PubMed primary reference and publication details, the PubMed primary reference is added in *bibref* and the publication details attributes in the *attributeList* of the *experimentDescription*. In PSI-MI XML 3.0 the *bibref* element has been updated to accept both *xref* and *attributeList* so that the publication can be entirely described within *bibref*.

## Results

All data resources using the IntAct database as their data storage repository, i.e., members of the IMEx Consortium [17] including IntAct, IID, InnateDB, MINT, DIP, MatrixDB, HPIDB routinely make their data available in PSI-MI XML3.0 in addition to the existing PSI-MI XML2.5 and MITAB 2.7 formats. Manually curated protein complexes from the Complex Portal are also made available in PSI-MI XML3.0. The PSI-MI maker software (https://github.com/MICommunity/psimi-maker-flattener), a desktop application that helps users to create PSI-MI XML documents and extract data from them, has been updated to support PSI-MI XML3.0. In addition, the new features included in PSI-MI XML 3.0 are currently being used to extend an existing tool suite, the MI Bundle, that integrates molecular, structural and genomics data and that already relies on the PSI-MI standard [18].

## Conclusion

PSI-MI XML3.0 will enable the molecular interaction community to meet the demands of new data types and increase our ability to systematically describe important biological events such as the composition, topology and stoichiometry of protein complexes, the cooperative binding of molecules to form new binding sites, and to modulate the activity of enzymes through allosteric binding. The accompanying PSI-MI controlled vocabulary used to populate this schema is also constantly being updated and expanded to more fully describe new ways of measuring molecular interactions and meet the needs of novel data types. We have developed a Java library, JAMI [19], The PSICQUIC web service [20], that is capable of both reading and writing all the PSI-MI formats, PSI-MI XML, MI-JSON and MITAB, to ensure that software developers are not faced with having to create multiple version of a program to address all versions of the interchange formats. The PSICQUIC web service [19] is also being improved, to handle the increased volume of data traffic as we move towards a comprehensive understanding of the interactomes of model organism species.

## Availability and requirements

Project name: PSI-MI XML3.0.

Project home page: e.g. http://psidev.info/groups/molecular-interactions GitHub source:https://github.com/HUPO-PSI/miXML/tree/master/3.0

Operating system(s): Platform independent.

Programming language: XML.

Other requirements:

License: Apache2.0.

Any restrictions to use by non-academics: None.

Availability: All example files are available in both Supplementary Materials and in GitHub, as listed in the article. The data used in the example files is also freely available from the IntAct or Complex Portal databases, as appropriate, with the exception of the cooperative interaction described in Additional file 8, which is not available in any public repository.

## Additional files

Additional file 1: Example file showing the representation of all molecular interaction data from a single publication (PMID: 26919541) in PSI-MI XML3.0.0 – note, includes use case 1.3 k, rewrite of bibliography section. (https://github.com/HUPO-PSI/miXML/blob/master/3.0/pub/Appendix%202.docx). (DOCX 44 kb)

Additional file 2: Representation of a negative feature range (use case 1.3a). (https://github.com/HUPO-PSI/miXML/blob/master/3.0/pub/Appendix%203.docx). (DOCX 27 kb)

Additional file 3: Representation of the sequence change caused by introduction of a mutation (use case 1.3b). (https://github.com/HUPO-PSI/miXML/blob/master/3.0/pub/Appendix%204.docx). (DOCX 38 kb)

Additional file 4: Representation of multiple feature detection methods and feature roles (use case 1.3c, use case 1.3d). (https://github.com/HUPO-PSI/miXML/blob/master/3.0/pub/Appendix%205.docx). (DOCX 68 kb)

Additional file 5: Representation of kinetic parameters added at feature level (use case 1.3e). (https://github.com/HUPO-PSI/miXML/blob/master/3.0/pub/Appendix%206.docx). (DOCX 39 kb)

Additional file 6: Representation of variable conditions (dynamic interactions) in an experiment (use case 1.3f). (https://github.com/HUPO-PSI/miXML/blob/master/3.0/pub/Appendix%207.docx). (DOCX 43 kb)

Additional file 7: Representation of an abstracted interaction, a manually curated protein complex, in PSI-MI XML3.0.0 (use case 1.3 g). (https://github.com/HUPO-PSI/miXML/blob/master/3.0/pub/Appendix%208.docx). (DOCX 48 kb)

Additional file 8: Representation of a cooperative interaction in PSI-MI XML3.0.0 (Use case 1.3 h). (https://github.com/HUPO-PSI/miXML/blob/master/3.0/pub/Appendix%209.docx). (DOCX 28 kb)

Additional file 9: Representation of molecule sets i.e. cases where a participant may be one of a list of molecules (use case 1.3i). (https://github.com/HUPO-PSI/miXML/blob/master/3.0/pub/Appendix%2010.docx). (DOCX 25 kb)

Additional file 10: Representation of the systematic capture of the stoichiometry of molecules within an interaction (use case 1.3j). (https://github.com/HUPO-PSI/miXML/blob/master/3.0/pub/Appendix%2011.docx). (DOCX 31 kb)

## Abbreviations

HUPO: Human Proteomics Organization
IMEx Consortium: International Molecular Exchange Consortium
MI: Molecular Interactions
PSI: Proteomics Standards Initiative
