# Supplementary material for: Encompassing new use cases - level 3.0 of the HUPO-PSI format for molecular interactions
Source: BMC Bioinformatics. 2018 Apr 11;19:134. doi: 10.1186/s12859-018-2118-1 (PMC5896046; doi:10.1186/s12859-018-2118-1)
Supplement: Supplementary file 1 — Example file showing the representation of all molecular interaction data from a single publication (PMID: 26919541) in PSI-MI XML3.0.0 – note, includes use case 1.3 k, rewrite of bibliography section. (https://github.com/HUPO-PSI/miXML/blob/master/3.0/pub/Appendix%202.docx). (DOCX 44 kb) [file 12859_2018_2118_MOESM1_ESM.docx]

*<?***xml version="1.0" encoding="UTF-8"***?>*

<**entrySet xmlns:xsi="http://www.w3.org/2001/XMLSchema-instance"**

**xmlns="http://psi.hupo.org/mi/mif300"**

**xsi:schemaLocation="http://psi.hupo.org/mi/mif300 https://raw.githubusercontent.com/HUPO-PSI/miXML/master/3.0/src/MIF300.xsd"**

**level="3" version="0" minorVersion="0"**>

<**entry**>

<**source releaseDate="2017-05-17"**>

<**names**>

<**shortLabel**>MINT</**shortLabel**>

<**fullName**>MINT, Dpt of Biology, University of Rome Tor Vergata</**fullName**>

</**names**>

<**xref**>

<**primaryRef db="psi-mi" dbAc="MI:0488" id="MI:0471" refType="identity" refTypeAc="MI:0356"**/>

<**secondaryRef db="intact" dbAc="MI:0469" id="EBI-1579228" refType="identity" refTypeAc="MI:0356"**/>

</**xref**>

<**attributeList**>

<**attribute name="url" nameAc="MI:0614"**>http://mint.bio.uniroma2.it/mint</**attribute**>

</**attributeList**>

</**source**>

<**experimentList**>

<**experimentDescription id="1"**>

<**names**>

<**fullName**>Structural basis for a novel interaction between TXNIP and Vav2.</**fullName**>

</**names**>

<**bibref**>

<**xref**>

<**primaryRef db="pubmed" dbAc="MI:0446" id="26919541" refType="primary-reference" refTypeAc="MI:0358"**/>

<**secondaryRef db="intact" dbAc="MI:0469" id="EBI-11683673" refType="identity" refTypeAc="MI:0356"**/>

<**secondaryRef db="imex" dbAc="MI:0670" id="IM-25084" refType="imex-primary" refTypeAc="MI:0662"**/>

</**xref**>

<**attributeList**>

<**attribute name="publication title" nameAc="MI:1091"**>Structural basis for a novel interaction between TXNIP and Vav2.</**attribute**>

<**attribute name="journal" nameAc="MI:0885"**>FEBS letters</**attribute**>

<**attribute name="publication year" nameAc="MI:0886"**>2016</**attribute**>

<**attribute name="curation depth" nameAc="MI:0955"**>imex curation</**attribute**>

<**attribute name="imex curation" nameAc="MI:0959"**/>

<**attribute name="author-list" nameAc="MI:0636"**>Liu S., Wu X., Zong M., Tempel W., Loppnau P., Liu Y.</**attribute**>

<**attribute name="contact-email" nameAc="MI:0634"**>yanliliu@mail.ccnu.edu.cn</**attribute**>

<**attribute name="full coverage" nameAc="MI:0957"**>Only protein-protein interactions</**attribute**>

<**attribute name="imex curation" nameAc="MI:0959"**>imex curation</**attribute**>

<**attribute name="author-announcement"**>22-Jun-2016: Contacted by IntAct-Help.</**attribute**>

</**attributeList**>

</**bibref**>

<**xref**>

<**primaryRef db="pubmed" dbAc="MI:0446" id="26919541" refType="primary-reference" refTypeAc="MI:0358"**/>

<**secondaryRef db="imex" dbAc="MI:0670" id="IM-25084" refType="imex-primary" refTypeAc="MI:0662"**/>

</**xref**>

<**hostOrganismList**>

<**hostOrganism ncbiTaxId="-1"**>

<**names**>

<**shortLabel**>in vitro</**shortLabel**>

<**fullName**>In vitro</**fullName**>

</**names**>

</**hostOrganism**>

</**hostOrganismList**>

<**interactionDetectionMethod**>

<**names**>

<**shortLabel**>itc</**shortLabel**>

<**fullName**>isothermal titration calorimetry</**fullName**>

<**alias type="go synonym" typeAc="MI:0303"**>ITC</**alias**>

</**names**>

<**xref**>

<**primaryRef db="psi-mi" dbAc="MI:0488" id="MI:0065" refType="identity" refTypeAc="MI:0356"**/>

<**secondaryRef db="intact" dbAc="MI:0469" id="EBI-1159" refType="identity" refTypeAc="MI:0356"**/>

<**secondaryRef db="pubmed" dbAc="MI:0446" id="11785756" refType="primary-reference" refTypeAc="MI:0358"**/>

</**xref**>

</**interactionDetectionMethod**>

<**participantIdentificationMethod**>

<**names**>

<**shortLabel**>predetermined</**shortLabel**>

<**fullName**>predetermined participant</**fullName**>

<**alias type="synonym" typeAc="MI:1041"**>predetermined</**alias**>

</**names**>

<**xref**>

<**primaryRef db="psi-mi" dbAc="MI:0488" id="MI:0396" refType="identity" refTypeAc="MI:0356"**/>

<**secondaryRef db="intact" dbAc="MI:0469" id="EBI-1465" refType="identity" refTypeAc="MI:0356"**/>

<**secondaryRef db="pubmed" dbAc="MI:0446" id="14755292" refType="primary-reference" refTypeAc="MI:0358"**/>

</**xref**>

</**participantIdentificationMethod**>

<**attributeList**>

<**attribute name="journal" nameAc="MI:0885"**>FEBS letters</**attribute**>

<**attribute name="publication year" nameAc="MI:0886"**>2016</**attribute**>

<**attribute name="curation depth" nameAc="MI:0955"**>imex curation</**attribute**>

<**attribute name="contact-email" nameAc="MI:0634"**>yanliliu@mail.ccnu.edu.cn</**attribute**>

<**attribute name="author-list" nameAc="MI:0636"**>Liu S., Wu X., Zong M., Tempel W., Loppnau P., Liu Y.</**attribute**>

<**attribute name="full coverage" nameAc="MI:0957"**>Only protein-protein interactions</**attribute**>

<**attribute name="imex curation" nameAc="MI:0959"**>imex curation</**attribute**>

<**attribute name="accepted"**>Accepted 2016-MAY-18 AT 09:47 BST AT 09:47 BST by MARTA</**attribute**>

</**attributeList**>

</**experimentDescription**>

<**experimentDescription id="2"**>

<**names**>

<**fullName**>Structural basis for a novel interaction between TXNIP and Vav2.</**fullName**>

</**names**>

<**bibref**>

<**xref**>

<**primaryRef db="pubmed" dbAc="MI:0446" id="26919541" refType="primary-reference" refTypeAc="MI:0358"**/>

<**secondaryRef db="intact" dbAc="MI:0469" id="EBI-11683673" refType="identity" refTypeAc="MI:0356"**/>

<**secondaryRef db="imex" dbAc="MI:0670" id="IM-25084" refType="imex-primary" refTypeAc="MI:0662"**/>

</**xref**>

<**attributeList**>

<**attribute name="publication title" nameAc="MI:1091"**>Structural basis for a novel interaction between TXNIP and Vav2.</**attribute**>

<**attribute name="journal" nameAc="MI:0885"**>FEBS letters</**attribute**>

<**attribute name="publication year" nameAc="MI:0886"**>2016</**attribute**>

<**attribute name="curation depth" nameAc="MI:0955"**>imex curation</**attribute**>

<**attribute name="imex curation" nameAc="MI:0959"**/>

<**attribute name="author-list" nameAc="MI:0636"**>Liu S., Wu X., Zong M., Tempel W., Loppnau P., Liu Y.</**attribute**>

<**attribute name="contact-email" nameAc="MI:0634"**>yanliliu@mail.ccnu.edu.cn</**attribute**>

<**attribute name="full coverage" nameAc="MI:0957"**>Only protein-protein interactions</**attribute**>

<**attribute name="imex curation" nameAc="MI:0959"**>imex curation</**attribute**>

<**attribute name="author-announcement"**>22-Jun-2016: Contacted by IntAct-Help.</**attribute**>

</**attributeList**>

</**bibref**>

<**xref**>

<**primaryRef db="pubmed" dbAc="MI:0446" id="26919541" refType="primary-reference" refTypeAc="MI:0358"**/>

<**secondaryRef db="imex" dbAc="MI:0670" id="IM-25084" refType="imex-primary" refTypeAc="MI:0662"**/>

</**xref**>

<**hostOrganismList**>

<**hostOrganism ncbiTaxId="-1"**>

<**names**>

<**shortLabel**>in vitro</**shortLabel**>

<**fullName**>In vitro</**fullName**>

</**names**>

</**hostOrganism**>

</**hostOrganismList**>

<**interactionDetectionMethod**>

<**names**>

<**shortLabel**>x-ray diffraction</**shortLabel**>

<**fullName**>x-ray crystallography</**fullName**>

<**alias type="go synonym" typeAc="MI:0303"**>X-ray</**alias**>

</**names**>

<**xref**>

<**primaryRef db="psi-mi" dbAc="MI:0488" id="MI:0114" refType="identity" refTypeAc="MI:0356"**/>

<**secondaryRef db="intact" dbAc="MI:0469" id="EBI-1272" refType="identity" refTypeAc="MI:0356"**/>

<**secondaryRef db="pubmed" dbAc="MI:0446" id="14755292" refType="primary-reference" refTypeAc="MI:0358"**/>

</**xref**>

</**interactionDetectionMethod**>

<**participantIdentificationMethod**>

<**names**>

<**shortLabel**>predetermined</**shortLabel**>

<**fullName**>predetermined participant</**fullName**>

<**alias type="synonym" typeAc="MI:1041"**>predetermined</**alias**>

</**names**>

<**xref**>

<**primaryRef db="psi-mi" dbAc="MI:0488" id="MI:0396" refType="identity" refTypeAc="MI:0356"**/>

<**secondaryRef db="intact" dbAc="MI:0469" id="EBI-1465" refType="identity" refTypeAc="MI:0356"**/>

<**secondaryRef db="pubmed" dbAc="MI:0446" id="14755292" refType="primary-reference" refTypeAc="MI:0358"**/>

</**xref**>

</**participantIdentificationMethod**>

<**attributeList**>

<**attribute name="journal" nameAc="MI:0885"**>FEBS letters</**attribute**>

<**attribute name="publication year" nameAc="MI:0886"**>2016</**attribute**>

<**attribute name="curation depth" nameAc="MI:0955"**>imex curation</**attribute**>

<**attribute name="contact-email" nameAc="MI:0634"**>yanliliu@mail.ccnu.edu.cn</**attribute**>

<**attribute name="author-list" nameAc="MI:0636"**>Liu S., Wu X., Zong M., Tempel W., Loppnau P., Liu Y.</**attribute**>

<**attribute name="full coverage" nameAc="MI:0957"**>Only protein-protein interactions</**attribute**>

<**attribute name="imex curation" nameAc="MI:0959"**>imex curation</**attribute**>

<**attribute name="accepted"**>Accepted 2016-MAY-18 AT 09:47 BST AT 09:47 BST by MARTA</**attribute**>

</**attributeList**>

</**experimentDescription**>

</**experimentList**>

<**interactorList**>

<**interactor id="3"**>

<**names**>

<**shortLabel**>vav2_human</**shortLabel**>

<**fullName**>Guanine nucleotide exchange factor VAV2</**fullName**>

<**alias type="gene name" typeAc="MI:0301"**>VAV2</**alias**>

</**names**>

<**xref**>

<**primaryRef db="uniprotkb" dbAc="MI:0486" id="P52735" version="SP_76" refType="identity" refTypeAc="MI:0356"**/>

<**secondaryRef db="uniprotkb" dbAc="MI:0486" id="A2RUM4" version="SP_88" refType="secondary-ac" refTypeAc="MI:0360"**/>

<**secondaryRef db="uniprotkb" dbAc="MI:0486" id="A8MQ12" version="SP_88" refType="secondary-ac" refTypeAc="MI:0360"**/>

<**secondaryRef db="uniprotkb" dbAc="MI:0486" id="Q5SYV3" version="SP_88" refType="secondary-ac" refTypeAc="MI:0360"**/>

<**secondaryRef db="uniprotkb" dbAc="MI:0486" id="Q5SYV4" version="SP_88" refType="secondary-ac" refTypeAc="MI:0360"**/>

<**secondaryRef db="uniprotkb" dbAc="MI:0486" id="Q5SYV5" version="SP_88" refType="secondary-ac" refTypeAc="MI:0360"**/>

<**secondaryRef db="uniprotkb" dbAc="MI:0486" id="Q6N012" version="SP_88" refType="secondary-ac" refTypeAc="MI:0360"**/>

<**secondaryRef db="uniprotkb" dbAc="MI:0486" id="Q6PIJ9" version="SP_88" refType="secondary-ac" refTypeAc="MI:0360"**/>

<**secondaryRef db="uniprotkb" dbAc="MI:0486" id="Q6Q317" version="SP_88" refType="secondary-ac" refTypeAc="MI:0360"**/>

<**secondaryRef db="uniprotkb" dbAc="MI:0486" id="B6ZDF5" version="SP_106" refType="secondary-ac" refTypeAc="MI:0360"**/>

<**secondaryRef db="intact" dbAc="MI:0469" id="EBI-297549" refType="identity" refTypeAc="MI:0356"**/>

<**secondaryRef db="ensembl" dbAc="MI:0476" id="ENST00000371850"**/>

<**secondaryRef db="ensembl" dbAc="MI:0476" id="ENST00000371851"**/>

<**secondaryRef db="ensembl" dbAc="MI:0476" id="ENST00000406606"**/>

<**secondaryRef db="go" dbAc="MI:0448" id="GO:0005886"**/>

<**secondaryRef db="go" dbAc="MI:0448" id="GO:0016477"**/>

<**secondaryRef db="refseq" dbAc="MI:0481" id="NP_001127870.1"**/>

<**secondaryRef db="refseq" dbAc="MI:0481" id="NP_003362.2"**/>

<**secondaryRef db="interpro" dbAc="MI:0449" id="IPR001715"**/>

<**secondaryRef db="interpro" dbAc="MI:0449" id="IPR000219"**/>

<**secondaryRef db="interpro" dbAc="MI:0449" id="IPR001331"**/>

<**secondaryRef db="interpro" dbAc="MI:0449" id="IPR011993"**/>

<**secondaryRef db="interpro" dbAc="MI:0449" id="IPR001849"**/>

<**secondaryRef db="interpro" dbAc="MI:0449" id="IPR002219"**/>

<**secondaryRef db="interpro" dbAc="MI:0449" id="IPR000980"**/>

<**secondaryRef db="interpro" dbAc="MI:0449" id="IPR011511"**/>

<**secondaryRef db="interpro" dbAc="MI:0449" id="IPR001452"**/>

<**secondaryRef db="go" dbAc="MI:0448" id="GO:0005829"**/>

<**secondaryRef db="go" dbAc="MI:0448" id="GO:0046872"**/>

<**secondaryRef db="go" dbAc="MI:0448" id="GO:0005089"**/>

<**secondaryRef db="go" dbAc="MI:0448" id="GO:0001525"**/>

<**secondaryRef db="go" dbAc="MI:0448" id="GO:0030168"**/>

<**secondaryRef db="go" dbAc="MI:0448" id="GO:0007264"**/>

<**secondaryRef db="rcsb pdb" dbAc="MI:0460" id="2DLZ"**/>

<**secondaryRef db="rcsb pdb" dbAc="MI:0460" id="2DM1"**/>

<**secondaryRef db="go" dbAc="MI:0448" id="GO:0030032"**/>

<**secondaryRef db="go" dbAc="MI:0448" id="GO:0043552"**/>

<**secondaryRef db="go" dbAc="MI:0448" id="GO:0007165"**/>

<**secondaryRef db="go" dbAc="MI:0448" id="GO:0051056"**/>

<**secondaryRef db="rcsb pdb" dbAc="MI:0460" id="2LNW"**/>

<**secondaryRef db="rcsb pdb" dbAc="MI:0460" id="2LNX"**/>

<**secondaryRef db="go" dbAc="MI:0448" id="GO:0005085"**/>

<**secondaryRef db="go" dbAc="MI:0448" id="GO:0038096"**/>

<**secondaryRef db="refseq" dbAc="MI:0481" id="XP_005272270.1"**/>

<**secondaryRef db="go" dbAc="MI:0448" id="GO:0038095"**/>

<**secondaryRef db="go" dbAc="MI:0448" id="GO:0043065"**/>

<**secondaryRef db="go" dbAc="MI:0448" id="GO:0010468"**/>

<**secondaryRef db="go" dbAc="MI:0448" id="GO:0030193"**/>

<**secondaryRef db="ensembl" dbAc="MI:0476" id="ENSG00000160293"**/>

<**secondaryRef db="ensembl" dbAc="MI:0476" id="ENSP00000360916"**/>

<**secondaryRef db="ensembl" dbAc="MI:0476" id="ENSP00000360917"**/>

<**secondaryRef db="ensembl" dbAc="MI:0476" id="ENSP00000385362"**/>

<**secondaryRef db="go" dbAc="MI:0448" id="GO:0008361"**/>

<**secondaryRef db="go" dbAc="MI:0448" id="GO:0035023"**/>

<**secondaryRef db="go" dbAc="MI:0448" id="GO:0043087"**/>

<**secondaryRef db="interpro" dbAc="MI:0449" id="IPR022613"**/>

<**secondaryRef db="reactome" dbAc="MI:0467" id="R-HSA-114604"**/>

<**secondaryRef db="reactome" dbAc="MI:0467" id="R-HSA-193648"**/>

<**secondaryRef db="reactome" dbAc="MI:0467" id="R-HSA-194840"**/>

<**secondaryRef db="reactome" dbAc="MI:0467" id="R-HSA-2029482"**/>

<**secondaryRef db="reactome" dbAc="MI:0467" id="R-HSA-2424491"**/>

<**secondaryRef db="reactome" dbAc="MI:0467" id="R-HSA-2871796"**/>

<**secondaryRef db="reactome" dbAc="MI:0467" id="R-HSA-2871809"**/>

<**secondaryRef db="reactome" dbAc="MI:0467" id="R-HSA-3928665"**/>

<**secondaryRef db="reactome" dbAc="MI:0467" id="R-HSA-416482"**/>

<**secondaryRef db="reactome" dbAc="MI:0467" id="R-HSA-4420097"**/>

<**secondaryRef db="reactome" dbAc="MI:0467" id="R-HSA-445144"**/>

<**secondaryRef db="reactome" dbAc="MI:0467" id="R-HSA-5218920"**/>

<**secondaryRef db="rcsb pdb" dbAc="MI:0460" id="4ROJ"**/>

<**secondaryRef db="go" dbAc="MI:0448" id="GO:0048010"**/>

<**secondaryRef db="go" dbAc="MI:0448" id="GO:0048013"**/>

</**xref**>

<**interactorType**>

<**names**>

<**shortLabel**>protein</**shortLabel**>

<**fullName**>protein</**fullName**>

</**names**>

<**xref**>

<**primaryRef db="psi-mi" dbAc="MI:0488" id="MI:0326" refType="identity" refTypeAc="MI:0356"**/>

<**secondaryRef db="intact" dbAc="MI:0469" id="EBI-619654" refType="identity" refTypeAc="MI:0356"**/>

<**secondaryRef db="pubmed" dbAc="MI:0446" id="14755292" refType="primary-reference" refTypeAc="MI:0358"**/>

<**secondaryRef db="so" dbAc="MI:0601" id="SO:0000358" refType="see-also" refTypeAc="MI:0361"**/>

</**xref**>

</**interactorType**>

<**organism ncbiTaxId="9606"**>

<**names**>

<**shortLabel**>human</**shortLabel**>

<**fullName**>Homo sapiens</**fullName**>

<**alias type="synonym" typeAc="MI:1041"**>Human</**alias**>

</**names**>

</**organism**>

<**sequence**>

MEQWRQCGRWLIDCKVLPPNHRVVWPSAVVFDLAQALRDGVLLCQLLHNLSPGSIDLKDINFRPQMSQFLCLKNIRTFLKVCHDKFGLRNSELFDPFDLFDVRDFGKVISAVSRLSLHSIAQNKGIRPFPSEETTENDDDVYRSLEELADEHDLGEDIYDCVPCEDGGDDIYEDIIKVEVQQPMIRYMQKMGMTEDDKRNCCLLEIQETEAKYYRTLEDIEKNYMSPLRLVLSPADMAAVFINLEDLIKVHHSFLRAIDVSVMVGGSTLAKVFLDFKERLLIYGEYCSHMEHAQNTLNQLLASREDFRQKVEECTLKVQDGKFKLQDLLVVPMQRVLKYHLLLKELLSHSAERPERQQLKEALEAMQDLAMYINEVKRDKETLRKISEFQSSIENLQVKLEEFGRPKIDGELKVRSIVNHTKQDRYLFLFDKVVIVCKRKGYSYELKEIIELLFHKMTDDPMNNKDVKKSHGKMWSYGFYLIHLQGKQGFQFFCKTEDMKRKWMEQFEMAMSNIKPDKANANHHSFQMYTFDKTTNCKACKMFLRGTFYQGYMCTKCGVGAHKECLEVIPPCKFTSPADLDASGAGPGPKMVAMQNYHGNPAPPGKPVLTFQTGDVLELLRGDPESPWWEGRLVQTRKSGYFPSSSVKPCPVDGRPPISRPPSREIDYTAYPWFAGNMERQQTDNLLKSHASGTYLIRERPAEAERFAISIKFNDEVKHIKVVEKDNWIHITEAKKFDSLLELVEYYQCHSLKESFKQLDTTLKYPYKSRERSASRASSRSPASCASYNFSFLSPQGLSFASQGPSAPFWSVFTPRVIGTAVARYNFAARDMRELSLREGDVVRIYSRIGGDQGWWKGETNGRIGWFPSTYVEEEGIQ

</**sequence**>

<**attributeList**>

<**attribute name="crc64"**>C186911605FD5B73</**attribute**>

</**attributeList**>

</**interactor**>

<**interactor id="4"**>

<**names**>

<**shortLabel**>src_human</**shortLabel**>

<**fullName**>Proto-oncogene tyrosine-protein kinase Src</**fullName**>

<**alias type="gene name synonym" typeAc="MI:0302"**>pp60c-src</**alias**>

<**alias type="gene name" typeAc="MI:0301"**>SRC</**alias**>

<**alias type="gene name synonym" typeAc="MI:0302"**>SRC1</**alias**>

<**alias type="gene name synonym" typeAc="MI:0302"**>Proto-oncogene c-Src</**alias**>

</**names**>

<**xref**>

<**primaryRef db="uniprotkb" dbAc="MI:0486" id="P12931" version="SP_111" refType="identity" refTypeAc="MI:0356"**/>

<**secondaryRef db="uniprotkb" dbAc="MI:0486" id="E1P5V4" version="SP_147" refType="secondary-ac" refTypeAc="MI:0360"**/>

<**secondaryRef db="uniprotkb" dbAc="MI:0486" id="Q86VB9" version="SP_111" refType="secondary-ac" refTypeAc="MI:0360"**/>

<**secondaryRef db="uniprotkb" dbAc="MI:0486" id="Q9H5A8" version="SP_111" refType="secondary-ac" refTypeAc="MI:0360"**/>

<**secondaryRef db="intact" dbAc="MI:0469" id="EBI-1383910" refType="intact-secondary"**/>

<**secondaryRef db="uniprotkb" dbAc="MI:0486" id="Q76P87" version="SP_133" refType="secondary-ac" refTypeAc="MI:0360"**/>

<**secondaryRef db="intact" dbAc="MI:0469" id="EBI-621482" refType="identity" refTypeAc="MI:0356"**/>

<**secondaryRef db="rcsb pdb" dbAc="MI:0460" id="4K11"**/>

<**secondaryRef db="ensembl" dbAc="MI:0476" id="ENST00000358208"**/>

<**secondaryRef db="ensembl" dbAc="MI:0476" id="ENST00000373558"**/>

<**secondaryRef db="ensembl" dbAc="MI:0476" id="ENST00000373567"**/>

<**secondaryRef db="ensembl" dbAc="MI:0476" id="ENST00000373578"**/>

<**secondaryRef db="rcsb pdb" dbAc="MI:0460" id="4F59"**/>

<**secondaryRef db="rcsb pdb" dbAc="MI:0460" id="4F5A"**/>

<**secondaryRef db="rcsb pdb" dbAc="MI:0460" id="4F5B"**/>

<**secondaryRef db="go" dbAc="MI:0448" id="GO:0005901"**/>

<**secondaryRef db="go" dbAc="MI:0448" id="GO:0005829"**/>

<**secondaryRef db="go" dbAc="MI:0448" id="GO:0005743"**/>

<**secondaryRef db="go" dbAc="MI:0448" id="GO:0005524"**/>

<**secondaryRef db="go" dbAc="MI:0448" id="GO:0020037"**/>

<**secondaryRef db="go" dbAc="MI:0448" id="GO:0004715"**/>

<**secondaryRef db="rcsb pdb" dbAc="MI:0460" id="1A07"**/>

<**secondaryRef db="rcsb pdb" dbAc="MI:0460" id="1A08"**/>

<**secondaryRef db="rcsb pdb" dbAc="MI:0460" id="1A09"**/>

<**secondaryRef db="rcsb pdb" dbAc="MI:0460" id="1A1A"**/>

<**secondaryRef db="rcsb pdb" dbAc="MI:0460" id="1A1B"**/>

<**secondaryRef db="go" dbAc="MI:0448" id="GO:0046777"**/>

<**secondaryRef db="rcsb pdb" dbAc="MI:0460" id="3VRO"**/>

<**secondaryRef db="refseq" dbAc="MI:0481" id="NP_005408.1"**/>

<**secondaryRef db="refseq" dbAc="MI:0481" id="NP_938033.1"**/>

<**secondaryRef db="interpro" dbAc="MI:0449" id="IPR011009"**/>

<**secondaryRef db="interpro" dbAc="MI:0449" id="IPR000719"**/>

<**secondaryRef db="interpro" dbAc="MI:0449" id="IPR017441"**/>

<**secondaryRef db="interpro" dbAc="MI:0449" id="IPR001245"**/>

<**secondaryRef db="interpro" dbAc="MI:0449" id="IPR000980"**/>

<**secondaryRef db="interpro" dbAc="MI:0449" id="IPR001452"**/>

<**secondaryRef db="interpro" dbAc="MI:0449" id="IPR008266"**/>

<**secondaryRef db="interpro" dbAc="MI:0449" id="IPR020635"**/>

<**secondaryRef db="ensembl" dbAc="MI:0476" id="ENSG00000197122"**/>

<**secondaryRef db="go" dbAc="MI:0448" id="GO:0045453"**/>

<**secondaryRef db="go" dbAc="MI:0448" id="GO:0007173"**/>

<**secondaryRef db="go" dbAc="MI:0448" id="GO:0050900"**/>

<**secondaryRef db="go" dbAc="MI:0448" id="GO:0048011"**/>

<**secondaryRef db="go" dbAc="MI:0448" id="GO:0030168"**/>

<**secondaryRef db="go" dbAc="MI:0448" id="GO:0033625"**/>

<**secondaryRef db="go" dbAc="MI:0448" id="GO:0045124"**/>

<**secondaryRef db="go" dbAc="MI:0448" id="GO:0043114"**/>

<**secondaryRef db="go" dbAc="MI:0448" id="GO:0070555"**/>

<**secondaryRef db="go" dbAc="MI:0448" id="GO:0007172"**/>

<**secondaryRef db="go" dbAc="MI:0448" id="GO:0031295"**/>

<**secondaryRef db="rcsb pdb" dbAc="MI:0460" id="4MXZ"**/>

<**secondaryRef db="go" dbAc="MI:0448" id="GO:0051902"**/>

<**secondaryRef db="rcsb pdb" dbAc="MI:0460" id="4MXO"**/>

<**secondaryRef db="rcsb pdb" dbAc="MI:0460" id="4MXX"**/>

<**secondaryRef db="rcsb pdb" dbAc="MI:0460" id="4MXY"**/>

<**secondaryRef db="go" dbAc="MI:0448" id="GO:0043393"**/>

<**secondaryRef db="go" dbAc="MI:0448" id="GO:0005884"**/>

<**secondaryRef db="go" dbAc="MI:0448" id="GO:0032587"**/>

<**secondaryRef db="go" dbAc="MI:0448" id="GO:0034446"**/>

<**secondaryRef db="go" dbAc="MI:0448" id="GO:0036120"**/>

<**secondaryRef db="go" dbAc="MI:0448" id="GO:0048010"**/>

<**secondaryRef db="go" dbAc="MI:0448" id="GO:0048013"**/>

<**secondaryRef db="go" dbAc="MI:0448" id="GO:0048471"**/>

<**secondaryRef db="go" dbAc="MI:0448" id="GO:0060491"**/>

<**secondaryRef db="go" dbAc="MI:0448" id="GO:0038096"**/>

<**secondaryRef db="go" dbAc="MI:0448" id="GO:0045087"**/>

<**secondaryRef db="rcsb pdb" dbAc="MI:0460" id="1A1C"**/>

<**secondaryRef db="rcsb pdb" dbAc="MI:0460" id="1A1E"**/>

<**secondaryRef db="rcsb pdb" dbAc="MI:0460" id="1FMK"**/>

<**secondaryRef db="rcsb pdb" dbAc="MI:0460" id="1HCS"**/>

<**secondaryRef db="rcsb pdb" dbAc="MI:0460" id="1HCT"**/>

<**secondaryRef db="rcsb pdb" dbAc="MI:0460" id="1KSW"**/>

<**secondaryRef db="rcsb pdb" dbAc="MI:0460" id="1O41"**/>

<**secondaryRef db="rcsb pdb" dbAc="MI:0460" id="1O42"**/>

<**secondaryRef db="rcsb pdb" dbAc="MI:0460" id="1O43"**/>

<**secondaryRef db="rcsb pdb" dbAc="MI:0460" id="1O44"**/>

<**secondaryRef db="rcsb pdb" dbAc="MI:0460" id="1O45"**/>

<**secondaryRef db="rcsb pdb" dbAc="MI:0460" id="1O46"**/>

<**secondaryRef db="rcsb pdb" dbAc="MI:0460" id="1O47"**/>

<**secondaryRef db="rcsb pdb" dbAc="MI:0460" id="1O48"**/>

<**secondaryRef db="rcsb pdb" dbAc="MI:0460" id="1O49"**/>

<**secondaryRef db="rcsb pdb" dbAc="MI:0460" id="1O4A"**/>

<**secondaryRef db="rcsb pdb" dbAc="MI:0460" id="1O4B"**/>

<**secondaryRef db="rcsb pdb" dbAc="MI:0460" id="1O4C"**/>

<**secondaryRef db="rcsb pdb" dbAc="MI:0460" id="1O4D"**/>

<**secondaryRef db="rcsb pdb" dbAc="MI:0460" id="1O4E"**/>

<**secondaryRef db="rcsb pdb" dbAc="MI:0460" id="1O4F"**/>

<**secondaryRef db="rcsb pdb" dbAc="MI:0460" id="1O4G"**/>

<**secondaryRef db="rcsb pdb" dbAc="MI:0460" id="1O4H"**/>

<**secondaryRef db="rcsb pdb" dbAc="MI:0460" id="1O4I"**/>

<**secondaryRef db="rcsb pdb" dbAc="MI:0460" id="1O4J"**/>

<**secondaryRef db="rcsb pdb" dbAc="MI:0460" id="1O4K"**/>

<**secondaryRef db="rcsb pdb" dbAc="MI:0460" id="1O4L"**/>

<**secondaryRef db="rcsb pdb" dbAc="MI:0460" id="1O4M"**/>

<**secondaryRef db="rcsb pdb" dbAc="MI:0460" id="1O4N"**/>

<**secondaryRef db="rcsb pdb" dbAc="MI:0460" id="1O4O"**/>

<**secondaryRef db="rcsb pdb" dbAc="MI:0460" id="1O4P"**/>

<**secondaryRef db="rcsb pdb" dbAc="MI:0460" id="1O4Q"**/>

<**secondaryRef db="rcsb pdb" dbAc="MI:0460" id="1O4R"**/>

<**secondaryRef db="rcsb pdb" dbAc="MI:0460" id="1SHD"**/>

<**secondaryRef db="rcsb pdb" dbAc="MI:0460" id="1Y57"**/>

<**secondaryRef db="rcsb pdb" dbAc="MI:0460" id="1YI6"**/>

<**secondaryRef db="rcsb pdb" dbAc="MI:0460" id="1YOJ"**/>

<**secondaryRef db="rcsb pdb" dbAc="MI:0460" id="1YOL"**/>

<**secondaryRef db="rcsb pdb" dbAc="MI:0460" id="1YOM"**/>

<**secondaryRef db="rcsb pdb" dbAc="MI:0460" id="2BDF"**/>

<**secondaryRef db="rcsb pdb" dbAc="MI:0460" id="2BDJ"**/>

<**secondaryRef db="rcsb pdb" dbAc="MI:0460" id="2H8H"**/>

<**secondaryRef db="rcsb pdb" dbAc="MI:0460" id="2SRC"**/>

<**secondaryRef db="go" dbAc="MI:0448" id="GO:0070062"**/>

<**secondaryRef db="rcsb pdb" dbAc="MI:0460" id="3ZMP"**/>

<**secondaryRef db="rcsb pdb" dbAc="MI:0460" id="3ZMQ"**/>

<**secondaryRef db="go" dbAc="MI:0448" id="GO:0005634"**/>

<**secondaryRef db="go" dbAc="MI:0448" id="GO:0007049"**/>

<**secondaryRef db="go" dbAc="MI:0448" id="GO:2001237"**/>

<**secondaryRef db="go" dbAc="MI:0448" id="GO:2001243"**/>

<**secondaryRef db="go" dbAc="MI:0448" id="GO:0071803"**/>

<**secondaryRef db="go" dbAc="MI:0448" id="GO:0005764"**/>

<**secondaryRef db="go" dbAc="MI:0448" id="GO:0005770"**/>

<**secondaryRef db="go" dbAc="MI:0448" id="GO:0090263"**/>

<**secondaryRef db="go" dbAc="MI:0448" id="GO:2000811"**/>

<**secondaryRef db="go" dbAc="MI:0448" id="GO:0030900"**/>

<**secondaryRef db="go" dbAc="MI:0448" id="GO:0033146"**/>

<**secondaryRef db="go" dbAc="MI:0448" id="GO:0048477"**/>

<**secondaryRef db="go" dbAc="MI:0448" id="GO:0050847"**/>

<**secondaryRef db="go" dbAc="MI:0448" id="GO:0051895"**/>

<**secondaryRef db="go" dbAc="MI:0448" id="GO:0060065"**/>

<**secondaryRef db="go" dbAc="MI:0448" id="GO:0060444"**/>

<**secondaryRef db="go" dbAc="MI:0448" id="GO:0071393"**/>

<**secondaryRef db="rcsb pdb" dbAc="MI:0460" id="4HXJ"**/>

<**secondaryRef db="go" dbAc="MI:0448" id="GO:0005070"**/>

<**secondaryRef db="go" dbAc="MI:0448" id="GO:0005178"**/>

<**secondaryRef db="go" dbAc="MI:0448" id="GO:0032463"**/>

<**secondaryRef db="go" dbAc="MI:0448" id="GO:0043154"**/>

<**secondaryRef db="go" dbAc="MI:0448" id="GO:0051897"**/>

<**secondaryRef db="go" dbAc="MI:0448" id="GO:0004672"**/>

<**secondaryRef db="go" dbAc="MI:0448" id="GO:0004713"**/>

<**secondaryRef db="go" dbAc="MI:0448" id="GO:0005102"**/>

<**secondaryRef db="go" dbAc="MI:0448" id="GO:0005737"**/>

<**secondaryRef db="go" dbAc="MI:0448" id="GO:0005739"**/>

<**secondaryRef db="go" dbAc="MI:0448" id="GO:0005886"**/>

<**secondaryRef db="go" dbAc="MI:0448" id="GO:0007165"**/>

<**secondaryRef db="go" dbAc="MI:0448" id="GO:0016301"**/>

<**secondaryRef db="go" dbAc="MI:0448" id="GO:0018108"**/>

<**secondaryRef db="go" dbAc="MI:0448" id="GO:0035556"**/>

<**secondaryRef db="go" dbAc="MI:0448" id="GO:0042169"**/>

<**secondaryRef db="go" dbAc="MI:0448" id="GO:0043066"**/>

<**secondaryRef db="go" dbAc="MI:0448" id="GO:0044325"**/>

<**secondaryRef db="go" dbAc="MI:0448" id="GO:0046875"**/>

<**secondaryRef db="go" dbAc="MI:0448" id="GO:0051219"**/>

<**secondaryRef db="go" dbAc="MI:0448" id="GO:0070851"**/>

<**secondaryRef db="go" dbAc="MI:0448" id="GO:0002223"**/>

<**secondaryRef db="go" dbAc="MI:0448" id="GO:0010634"**/>

<**secondaryRef db="go" dbAc="MI:0448" id="GO:0050731"**/>

<**secondaryRef db="go" dbAc="MI:0448" id="GO:2000394"**/>

<**secondaryRef db="go" dbAc="MI:0448" id="GO:0007179"**/>

<**secondaryRef db="go" dbAc="MI:0448" id="GO:0007229"**/>

<**secondaryRef db="go" dbAc="MI:0448" id="GO:0019899"**/>

<**secondaryRef db="go" dbAc="MI:0448" id="GO:0022407"**/>

<**secondaryRef db="go" dbAc="MI:0448" id="GO:0043149"**/>

<**secondaryRef db="go" dbAc="MI:0448" id="GO:2000641"**/>

<**secondaryRef db="go" dbAc="MI:0448" id="GO:2001286"**/>

<**secondaryRef db="go" dbAc="MI:0448" id="GO:0010632"**/>

<**secondaryRef db="reactome" dbAc="MI:0467" id="R-HSA-418885"**/>

<**secondaryRef db="reactome" dbAc="MI:0467" id="R-HSA-418886"**/>

<**secondaryRef db="reactome" dbAc="MI:0467" id="R-HSA-430116"**/>

<**secondaryRef db="reactome" dbAc="MI:0467" id="R-HSA-437239"**/>

<**secondaryRef db="reactome" dbAc="MI:0467" id="R-HSA-4420097"**/>

<**secondaryRef db="reactome" dbAc="MI:0467" id="R-HSA-456926"**/>

<**secondaryRef db="reactome" dbAc="MI:0467" id="R-HSA-5218921"**/>

<**secondaryRef db="reactome" dbAc="MI:0467" id="R-HSA-5607764"**/>

<**secondaryRef db="reactome" dbAc="MI:0467" id="R-HSA-5663220"**/>

<**secondaryRef db="reactome" dbAc="MI:0467" id="R-HSA-5673000"**/>

<**secondaryRef db="reactome" dbAc="MI:0467" id="R-HSA-5674135"**/>

<**secondaryRef db="ensembl" dbAc="MI:0476" id="ENSP00000350941"**/>

<**secondaryRef db="ensembl" dbAc="MI:0476" id="ENSP00000362659"**/>

<**secondaryRef db="ensembl" dbAc="MI:0476" id="ENSP00000362668"**/>

<**secondaryRef db="ensembl" dbAc="MI:0476" id="ENSP00000362680"**/>

<**secondaryRef db="reactome" dbAc="MI:0467" id="R-HSA-1227986"**/>

<**secondaryRef db="reactome" dbAc="MI:0467" id="R-HSA-1295596"**/>

<**secondaryRef db="reactome" dbAc="MI:0467" id="R-HSA-1433557"**/>

<**secondaryRef db="reactome" dbAc="MI:0467" id="R-HSA-1433559"**/>

<**secondaryRef db="reactome" dbAc="MI:0467" id="R-HSA-171007"**/>

<**secondaryRef db="reactome" dbAc="MI:0467" id="R-HSA-177929"**/>

<**secondaryRef db="reactome" dbAc="MI:0467" id="R-HSA-180292"**/>

<**secondaryRef db="reactome" dbAc="MI:0467" id="R-HSA-186763"**/>

<**secondaryRef db="reactome" dbAc="MI:0467" id="R-HSA-191647"**/>

<**secondaryRef db="reactome" dbAc="MI:0467" id="R-HSA-2029481"**/>

<**secondaryRef db="reactome" dbAc="MI:0467" id="R-HSA-210990"**/>

<**secondaryRef db="reactome" dbAc="MI:0467" id="R-HSA-2682334"**/>

<**secondaryRef db="reactome" dbAc="MI:0467" id="R-HSA-354192"**/>

<**secondaryRef db="reactome" dbAc="MI:0467" id="R-HSA-354194"**/>

<**secondaryRef db="reactome" dbAc="MI:0467" id="R-HSA-372708"**/>

<**secondaryRef db="reactome" dbAc="MI:0467" id="R-HSA-375165"**/>

<**secondaryRef db="reactome" dbAc="MI:0467" id="R-HSA-389356"**/>

<**secondaryRef db="reactome" dbAc="MI:0467" id="R-HSA-389513"**/>

<**secondaryRef db="reactome" dbAc="MI:0467" id="R-HSA-391160"**/>

<**secondaryRef db="reactome" dbAc="MI:0467" id="R-HSA-3928662"**/>

<**secondaryRef db="reactome" dbAc="MI:0467" id="R-HSA-3928663"**/>

<**secondaryRef db="reactome" dbAc="MI:0467" id="R-HSA-3928664"**/>

<**secondaryRef db="reactome" dbAc="MI:0467" id="R-HSA-3928665"**/>

<**secondaryRef db="reactome" dbAc="MI:0467" id="R-HSA-418592"**/>

<**secondaryRef db="go" dbAc="MI:0448" id="GO:0007417"**/>

<**secondaryRef db="go" dbAc="MI:0448" id="GO:0030520"**/>

<**secondaryRef db="go" dbAc="MI:0448" id="GO:0038128"**/>

<**secondaryRef db="go" dbAc="MI:0448" id="GO:0048008"**/>

<**secondaryRef db="reactome" dbAc="MI:0467" id="R-HSA-6811558"**/>

<**secondaryRef db="go" dbAc="MI:0448" id="GO:0031234"**/>

<**secondaryRef db="go" dbAc="MI:0448" id="GO:0036035"**/>

<**secondaryRef db="go" dbAc="MI:0448" id="GO:0038083"**/>

<**secondaryRef db="go" dbAc="MI:0448" id="GO:0042127"**/>

<**secondaryRef db="go" dbAc="MI:0448" id="GO:0051427"**/>

<**secondaryRef db="go" dbAc="MI:0448" id="GO:0051726"**/>

<**secondaryRef db="go" dbAc="MI:0448" id="GO:0071375"**/>

<**secondaryRef db="go" dbAc="MI:0448" id="GO:0071801"**/>

<**secondaryRef db="go" dbAc="MI:0448" id="GO:0097110"**/>

<**secondaryRef db="go" dbAc="MI:0448" id="GO:0070374"**/>

<**secondaryRef db="go" dbAc="MI:0448" id="GO:0086098"**/>

<**secondaryRef db="go" dbAc="MI:0448" id="GO:0008283"**/>

<**secondaryRef db="go" dbAc="MI:0448" id="GO:0009612"**/>

<**secondaryRef db="go" dbAc="MI:0448" id="GO:0009615"**/>

<**secondaryRef db="go" dbAc="MI:0448" id="GO:0010641"**/>

<**secondaryRef db="go" dbAc="MI:0448" id="GO:0010907"**/>

<**secondaryRef db="go" dbAc="MI:0448" id="GO:0014069"**/>

<**secondaryRef db="go" dbAc="MI:0448" id="GO:0014911"**/>

<**secondaryRef db="go" dbAc="MI:0448" id="GO:0016337"**/>

<**secondaryRef db="go" dbAc="MI:0448" id="GO:0018105"**/>

<**secondaryRef db="go" dbAc="MI:0448" id="GO:0031667"**/>

<**secondaryRef db="go" dbAc="MI:0448" id="GO:0031954"**/>

<**secondaryRef db="go" dbAc="MI:0448" id="GO:0010447"**/>

<**secondaryRef db="go" dbAc="MI:0448" id="GO:0032148"**/>

<**secondaryRef db="go" dbAc="MI:0448" id="GO:0032869"**/>

<**secondaryRef db="go" dbAc="MI:0448" id="GO:0034332"**/>

<**secondaryRef db="go" dbAc="MI:0448" id="GO:0042493"**/>

<**secondaryRef db="go" dbAc="MI:0448" id="GO:0042542"**/>

<**secondaryRef db="go" dbAc="MI:0448" id="GO:0043005"**/>

<**secondaryRef db="go" dbAc="MI:0448" id="GO:0043065"**/>

<**secondaryRef db="go" dbAc="MI:0448" id="GO:0043406"**/>

<**secondaryRef db="go" dbAc="MI:0448" id="GO:0043552"**/>

<**secondaryRef db="go" dbAc="MI:0448" id="GO:0045056"**/>

<**secondaryRef db="go" dbAc="MI:0448" id="GO:0045737"**/>

<**secondaryRef db="go" dbAc="MI:0448" id="GO:0045892"**/>

<**secondaryRef db="go" dbAc="MI:0448" id="GO:0045893"**/>

<**secondaryRef db="go" dbAc="MI:0448" id="GO:0046628"**/>

<**secondaryRef db="go" dbAc="MI:0448" id="GO:0050715"**/>

<**secondaryRef db="go" dbAc="MI:0448" id="GO:0051385"**/>

<**secondaryRef db="go" dbAc="MI:0448" id="GO:0051602"**/>

<**secondaryRef db="go" dbAc="MI:0448" id="GO:0071222"**/>

<**secondaryRef db="go" dbAc="MI:0448" id="GO:0071398"**/>

<**secondaryRef db="go" dbAc="MI:0448" id="GO:0071456"**/>

<**secondaryRef db="go" dbAc="MI:0448" id="GO:2000573"**/>

<**secondaryRef db="go" dbAc="MI:0448" id="GO:0031648"**/>

<**secondaryRef db="go" dbAc="MI:0448" id="GO:0034614"**/>

<**secondaryRef db="go" dbAc="MI:0448" id="GO:0071498"**/>

<**secondaryRef db="refseq" dbAc="MI:0481" id="XP_011527315.1"**/>

<**secondaryRef db="go" dbAc="MI:0448" id="GO:0032211"**/>

<**secondaryRef db="go" dbAc="MI:0448" id="GO:0051974"**/>

<**secondaryRef db="go" dbAc="MI:0448" id="GO:0019900"**/>

<**secondaryRef db="go" dbAc="MI:0448" id="GO:0071902"**/>

<**secondaryRef db="go" dbAc="MI:0448" id="GO:0007411"**/>

<**secondaryRef db="go" dbAc="MI:0448" id="GO:0035635"**/>

<**secondaryRef db="go" dbAc="MI:0448" id="GO:0045296"**/>

<**secondaryRef db="reactome" dbAc="MI:0467" id="R-HSA-8876493"**/>

<**secondaryRef db="go" dbAc="MI:0448" id="GO:0002102"**/>

<**secondaryRef db="go" dbAc="MI:0448" id="GO:0010954"**/>

<**secondaryRef db="go" dbAc="MI:0448" id="GO:0051057"**/>

<**secondaryRef db="go" dbAc="MI:0448" id="GO:1900182"**/>

<**secondaryRef db="reactome" dbAc="MI:0467" id="R-HSA-8853659"**/>

<**secondaryRef db="refseq" dbAc="MI:0481" id="XP_016883513.1"**/>

<**secondaryRef db="refseq" dbAc="MI:0481" id="XP_016883514.1"**/>

<**secondaryRef db="refseq" dbAc="MI:0481" id="XP_016883515.1"**/>

<**secondaryRef db="refseq" dbAc="MI:0481" id="XP_016883516.1"**/>

<**secondaryRef db="reactome" dbAc="MI:0467" id="R-HSA-6802946"**/>

<**secondaryRef db="reactome" dbAc="MI:0467" id="R-HSA-6802948"**/>

<**secondaryRef db="reactome" dbAc="MI:0467" id="R-HSA-6802949"**/>

<**secondaryRef db="reactome" dbAc="MI:0467" id="R-HSA-6802952"**/>

<**secondaryRef db="reactome" dbAc="MI:0467" id="R-HSA-6802955"**/>

<**secondaryRef db="reactome" dbAc="MI:0467" id="R-HSA-8874081"**/>

</**xref**>

<**interactorType**>

<**names**>

<**shortLabel**>protein</**shortLabel**>

<**fullName**>protein</**fullName**>

</**names**>

<**xref**>

<**primaryRef db="psi-mi" dbAc="MI:0488" id="MI:0326" refType="identity" refTypeAc="MI:0356"**/>

<**secondaryRef db="intact" dbAc="MI:0469" id="EBI-619654" refType="identity" refTypeAc="MI:0356"**/>

<**secondaryRef db="pubmed" dbAc="MI:0446" id="14755292" refType="primary-reference" refTypeAc="MI:0358"**/>

<**secondaryRef db="so" dbAc="MI:0601" id="SO:0000358" refType="see-also" refTypeAc="MI:0361"**/>

</**xref**>

</**interactorType**>

<**organism ncbiTaxId="9606"**>

<**names**>

<**shortLabel**>human</**shortLabel**>

<**fullName**>Homo sapiens</**fullName**>

<**alias type="synonym" typeAc="MI:1041"**>Human</**alias**>

</**names**>

</**organism**>

<**sequence**>

MGSNKSKPKDASQRRRSLEPAENVHGAGGGAFPASQTPSKPASADGHRGPSAAFAPAAAEPKLFGGFNSSDTVTSPQRAGPLAGGVTTFVALYDYESRTETDLSFKKGERLQIVNNTEGDWWLAHSLSTGQTGYIPSNYVAPSDSIQAEEWYFGKITRRESERLLLNAENPRGTFLVRESETTKGAYCLSVSDFDNAKGLNVKHYKIRKLDSGGFYITSRTQFNSLQQLVAYYSKHADGLCHRLTTVCPTSKPQTQGLAKDAWEIPRESLRLEVKLGQGCFGEVWMGTWNGTTRVAIKTLKPGTMSPEAFLQEAQVMKKLRHEKLVQLYAVVSEEPIYIVTEYMSKGSLLDFLKGETGKYLRLPQLVDMAAQIASGMAYVERMNYVHRDLRAANILVGENLVCKVADFGLARLIEDNEYTARQGAKFPIKWTAPEAALYGRFTIKSDVWSFGILLTELTTKGRVPYPGMVNREVLDQVERGYRMPCPPECPESLHDLMCQCWRKEPEERPTFEYLQAFLEDYFTSTEPQYQPGENL

</**sequence**>

<**attributeList**>

<**attribute name="crc64"**>C1908084683E5DE8</**attribute**>

</**attributeList**>

</**interactor**>

<**interactor id="5"**>

<**names**>

<**shortLabel**>txnip_human</**shortLabel**>

<**fullName**>Thioredoxin-interacting protein</**fullName**>

<**alias type="gene name" typeAc="MI:0301"**>TXNIP</**alias**>

<**alias type="gene name synonym" typeAc="MI:0302"**>VDUP1</**alias**>

<**alias type="gene name synonym" typeAc="MI:0302"**>Vitamin D3 up-regulated protein 1</**alias**>

<**alias type="gene name synonym" typeAc="MI:0302"**>Thioredoxin-binding protein 2</**alias**>

</**names**>

<**xref**>

<**primaryRef db="uniprotkb" dbAc="MI:0486" id="Q9H3M7" version="SP_33" refType="identity" refTypeAc="MI:0356"**/>

<**secondaryRef db="uniprotkb" dbAc="MI:0486" id="B4E3D3" version="SP_100" refType="secondary-ac" refTypeAc="MI:0360"**/>

<**secondaryRef db="uniprotkb" dbAc="MI:0486" id="Q16226" version="SP_33" refType="secondary-ac" refTypeAc="MI:0360"**/>

<**secondaryRef db="uniprotkb" dbAc="MI:0486" id="Q6PML0" version="SP_33" refType="secondary-ac" refTypeAc="MI:0360"**/>

<**secondaryRef db="uniprotkb" dbAc="MI:0486" id="Q9BXG9" version="SP_33" refType="secondary-ac" refTypeAc="MI:0360"**/>

<**secondaryRef db="intact" dbAc="MI:0469" id="EBI-1369170" refType="identity" refTypeAc="MI:0356"**/>

<**secondaryRef db="go" dbAc="MI:0448" id="GO:0000122"**/>

<**secondaryRef db="rcsb pdb" dbAc="MI:0460" id="4GEI"**/>

<**secondaryRef db="rcsb pdb" dbAc="MI:0460" id="4GEJ"**/>

<**secondaryRef db="go" dbAc="MI:0448" id="GO:0005829"**/>

<**secondaryRef db="go" dbAc="MI:0448" id="GO:0007049"**/>

<**secondaryRef db="go" dbAc="MI:0448" id="GO:0071228"**/>

<**secondaryRef db="go" dbAc="MI:0448" id="GO:0030216"**/>

<**secondaryRef db="go" dbAc="MI:0448" id="GO:0051782"**/>

<**secondaryRef db="go" dbAc="MI:0448" id="GO:0006351"**/>

<**secondaryRef db="refseq" dbAc="MI:0481" id="NP_006463.3"**/>

<**secondaryRef db="interpro" dbAc="MI:0449" id="IPR011022"**/>

<**secondaryRef db="interpro" dbAc="MI:0449" id="IPR011021"**/>

<**secondaryRef db="interpro" dbAc="MI:0449" id="IPR014756"**/>

<**secondaryRef db="go" dbAc="MI:0448" id="GO:0004857"**/>

<**secondaryRef db="go" dbAc="MI:0448" id="GO:0005634"**/>

<**secondaryRef db="go" dbAc="MI:0448" id="GO:0005758"**/>

<**secondaryRef db="go" dbAc="MI:0448" id="GO:0006606"**/>

<**secondaryRef db="go" dbAc="MI:0448" id="GO:0009612"**/>

<**secondaryRef db="go" dbAc="MI:0448" id="GO:0009749"**/>

<**secondaryRef db="go" dbAc="MI:0448" id="GO:0032355"**/>

<**secondaryRef db="go" dbAc="MI:0448" id="GO:0032570"**/>

<**secondaryRef db="go" dbAc="MI:0448" id="GO:0042127"**/>

<**secondaryRef db="go" dbAc="MI:0448" id="GO:0042493"**/>

<**secondaryRef db="go" dbAc="MI:0448" id="GO:0042542"**/>

<**secondaryRef db="go" dbAc="MI:0448" id="GO:0043065"**/>

<**secondaryRef db="go" dbAc="MI:0448" id="GO:0048008"**/>

<**secondaryRef db="go" dbAc="MI:0448" id="GO:0051592"**/>

<**secondaryRef db="go" dbAc="MI:0448" id="GO:0005737"**/>

<**secondaryRef db="go" dbAc="MI:0448" id="GO:0031625"**/>

<**secondaryRef db="rcsb pdb" dbAc="MI:0460" id="4GFX"**/>

<**secondaryRef db="rcsb pdb" dbAc="MI:0460" id="4LL1"**/>

<**secondaryRef db="rcsb pdb" dbAc="MI:0460" id="4LL4"**/>

<**secondaryRef db="refseq" dbAc="MI:0481" id="NP_001300901.1"**/>

<**secondaryRef db="ensembl" dbAc="MI:0476" id="ENSP00000396322"**/>

<**secondaryRef db="ensembl" dbAc="MI:0476" id="ENST00000425134"**/>

<**secondaryRef db="ensembl" dbAc="MI:0476" id="ENSG00000265972"**/>

<**secondaryRef db="ensembl" dbAc="MI:0476" id="ENSP00000462521"**/>

<**secondaryRef db="ensembl" dbAc="MI:0476" id="ENST00000582401"**/>

<**secondaryRef db="reactome" dbAc="MI:0467" id="R-HSA-844456"**/>

<**secondaryRef db="rcsb pdb" dbAc="MI:0460" id="4ROF"**/>

<**secondaryRef db="rcsb pdb" dbAc="MI:0460" id="4ROJ"**/>

<**secondaryRef db="rcsb pdb" dbAc="MI:0460" id="5CQ2"**/>

<**secondaryRef db="rcsb pdb" dbAc="MI:0460" id="5DF6"**/>

<**secondaryRef db="rcsb pdb" dbAc="MI:0460" id="5DWS"**/>

<**secondaryRef db="rcsb pdb" dbAc="MI:0460" id="5DZD"**/>

<**secondaryRef db="interpro" dbAc="MI:0449" id="IPR014752"**/>

</**xref**>

<**interactorType**>

<**names**>

<**shortLabel**>protein</**shortLabel**>

<**fullName**>protein</**fullName**>

</**names**>

<**xref**>

<**primaryRef db="psi-mi" dbAc="MI:0488" id="MI:0326" refType="identity" refTypeAc="MI:0356"**/>

<**secondaryRef db="intact" dbAc="MI:0469" id="EBI-619654" refType="identity" refTypeAc="MI:0356"**/>

<**secondaryRef db="pubmed" dbAc="MI:0446" id="14755292" refType="primary-reference" refTypeAc="MI:0358"**/>

<**secondaryRef db="so" dbAc="MI:0601" id="SO:0000358" refType="see-also" refTypeAc="MI:0361"**/>

</**xref**>

</**interactorType**>

<**organism ncbiTaxId="9606"**>

<**names**>

<**shortLabel**>human</**shortLabel**>

<**fullName**>Homo sapiens</**fullName**>

<**alias type="synonym" typeAc="MI:1041"**>Human</**alias**>

</**names**>

</**organism**>

<**sequence**>

MVMFKKIKSFEVVFNDPEKVYGSGEKVAGRVIVEVCEVTRVKAVRILACGVAKVLWMQGSQQCKQTSEYLRYEDTLLLEDQPTGENEMVIMRPGNKYEYKFGFELPQGPLGTSFKGKYGCVDYWVKAFLDRPSQPTQETKKNFEVVDLVDVNTPDLMAPVSAKKEKKVSCMFIPDGRVSVSARIDRKGFCEGDEISIHADFENTCSRIVVPKAAIVARHTYLANGQTKVLTQKLSSVRGNHIISGTCASWRGKSLRVQKIRPSILGCNILRVEYSLLIYVSVPGSKKVILDLPLVIGSRSGLSSRTSSMASRTSSEMSWVDLNIPDTPEAPPCYMDVIPEDHRLESPTTPLLDDMDGSQDSPIFMYAPEFKFMPPPTYTEVDPCILNNNVQ

</**sequence**>

<**attributeList**>

<**attribute name="crc64"**>B0FE2D35D0B0735A</**attribute**>

</**attributeList**>

</**interactor**>

</**interactorList**>

<**interactionList**>

<**interaction id="6" imexId="IM-25084-1"**>

<**names**>

<**shortLabel**>txnip-vav2-1</**shortLabel**>

</**names**>

<**xref**>

<**primaryRef db="intact" dbAc="MI:0469" id="EBI-11686229" refType="identity" refTypeAc="MI:0356"**/>

<**secondaryRef db="rcsb pdb" dbAc="MI:0460" id="4ROJ"**/>

<**secondaryRef db="imex" dbAc="MI:0670" id="IM-25084-1" refType="imex-primary" refTypeAc="MI:0662"**/>

</**xref**>

<**experimentList**>

<**experimentRef**>2</**experimentRef**>

</**experimentList**>

<**participantList**>

<**participant id="7"**>

<**interactorRef**>5</**interactorRef**>

<**biologicalRole**>

<**names**>

<**shortLabel**>unspecified role</**shortLabel**>

<**fullName**>unspecified role</**fullName**>

</**names**>

<**xref**>

<**primaryRef db="psi-mi" dbAc="MI:0488" id="MI:0499" refType="identity" refTypeAc="MI:0356"**/>

<**secondaryRef db="intact" dbAc="MI:0469" id="EBI-77781" refType="identity" refTypeAc="MI:0356"**/>

<**secondaryRef db="pubmed" dbAc="MI:0446" id="14755292" refType="primary-reference" refTypeAc="MI:0358"**/>

</**xref**>

</**biologicalRole**>

<**experimentalRoleList**>

<**experimentalRole**>

<**names**>

<**shortLabel**>neutral component</**shortLabel**>

<**fullName**>neutral component</**fullName**>

</**names**>

<**xref**>

<**primaryRef db="psi-mi" dbAc="MI:0488" id="MI:0497" refType="identity" refTypeAc="MI:0356"**/>

<**secondaryRef db="intact" dbAc="MI:0469" id="EBI-55" refType="identity" refTypeAc="MI:0356"**/>

<**secondaryRef db="pubmed" dbAc="MI:0446" id="14755292" refType="primary-reference" refTypeAc="MI:0358"**/>

</**xref**>

</**experimentalRole**>

</**experimentalRoleList**>

<**experimentalPreparationList**>

<**experimentalPreparation**>

<**names**>

<**shortLabel**>purified</**shortLabel**>

<**fullName**>purified</**fullName**>

</**names**>

<**xref**>

<**primaryRef db="psi-mi" dbAc="MI:0488" id="MI:0350" refType="identity" refTypeAc="MI:0356"**/>

<**secondaryRef db="intact" dbAc="MI:0469" id="EBI-1537811" refType="identity" refTypeAc="MI:0356"**/>

<**secondaryRef db="pubmed" dbAc="MI:0446" id="14755292" refType="primary-reference" refTypeAc="MI:0358"**/>

</**xref**>

</**experimentalPreparation**>

</**experimentalPreparationList**>

<**featureList**>

<**feature id="8"**>

<**names**>

<**shortLabel**>binding range</**shortLabel**>

<**fullName**>binding range</**fullName**>

</**names**>

<**xref**>

<**primaryRef db="intact" dbAc="MI:0469" id="EBI-11686235" refType="identity" refTypeAc="MI:0356"**/>

</**xref**>

<**featureType**>

<**names**>

<**shortLabel**>binding region</**shortLabel**>

<**fullName**>binding-associated region</**fullName**>

</**names**>

<**xref**>

<**primaryRef db="psi-mi" dbAc="MI:0488" id="MI:0117" refType="identity" refTypeAc="MI:0356"**/>

<**secondaryRef db="intact" dbAc="MI:0469" id="EBI-456493" refType="identity" refTypeAc="MI:0356"**/>

<**secondaryRef db="pubmed" dbAc="MI:0446" id="14755292" refType="primary-reference" refTypeAc="MI:0358"**/>

</**xref**>

</**featureType**>

<**featureRangeList**>

<**featureRange**>

<**startStatus**>

<**names**>

<**shortLabel**>certain</**shortLabel**>

<**fullName**>certain sequence position</**fullName**>

<**alias type="synonym" typeAc="MI:1041"**>certain</**alias**>

</**names**>

<**xref**>

<**primaryRef db="psi-mi" dbAc="MI:0488" id="MI:0335" refType="identity" refTypeAc="MI:0356"**/>

<**secondaryRef db="intact" dbAc="MI:0469" id="EBI-540564" refType="identity" refTypeAc="MI:0356"**/>

<**secondaryRef db="pubmed" dbAc="MI:0446" id="14755292" refType="primary-reference" refTypeAc="MI:0358"**/>

</**xref**>

</**startStatus**>

<**begin position="327"**/>

<**endStatus**>

<**names**>

<**shortLabel**>certain</**shortLabel**>

<**fullName**>certain sequence position</**fullName**>

<**alias type="synonym" typeAc="MI:1041"**>certain</**alias**>

</**names**>

<**xref**>

<**primaryRef db="psi-mi" dbAc="MI:0488" id="MI:0335" refType="identity" refTypeAc="MI:0356"**/>

<**secondaryRef db="intact" dbAc="MI:0469" id="EBI-540564" refType="identity" refTypeAc="MI:0356"**/>

<**secondaryRef db="pubmed" dbAc="MI:0446" id="14755292" refType="primary-reference" refTypeAc="MI:0358"**/>

</**xref**>

</**endStatus**>

<**end position="338"**/>

</**featureRange**>

</**featureRangeList**>

</**feature**>

</**featureList**>

</**participant**>

<**participant id="9"**>

<**interactorRef**>3</**interactorRef**>

<**biologicalRole**>

<**names**>

<**shortLabel**>unspecified role</**shortLabel**>

<**fullName**>unspecified role</**fullName**>

</**names**>

<**xref**>

<**primaryRef db="psi-mi" dbAc="MI:0488" id="MI:0499" refType="identity" refTypeAc="MI:0356"**/>

<**secondaryRef db="intact" dbAc="MI:0469" id="EBI-77781" refType="identity" refTypeAc="MI:0356"**/>

<**secondaryRef db="pubmed" dbAc="MI:0446" id="14755292" refType="primary-reference" refTypeAc="MI:0358"**/>

</**xref**>

</**biologicalRole**>

<**experimentalRoleList**>

<**experimentalRole**>

<**names**>

<**shortLabel**>neutral component</**shortLabel**>

<**fullName**>neutral component</**fullName**>

</**names**>

<**xref**>

<**primaryRef db="psi-mi" dbAc="MI:0488" id="MI:0497" refType="identity" refTypeAc="MI:0356"**/>

<**secondaryRef db="intact" dbAc="MI:0469" id="EBI-55" refType="identity" refTypeAc="MI:0356"**/>

<**secondaryRef db="pubmed" dbAc="MI:0446" id="14755292" refType="primary-reference" refTypeAc="MI:0358"**/>

</**xref**>

</**experimentalRole**>

</**experimentalRoleList**>

<**experimentalPreparationList**>

<**experimentalPreparation**>

<**names**>

<**shortLabel**>purified</**shortLabel**>

<**fullName**>purified</**fullName**>

</**names**>

<**xref**>

<**primaryRef db="psi-mi" dbAc="MI:0488" id="MI:0350" refType="identity" refTypeAc="MI:0356"**/>

<**secondaryRef db="intact" dbAc="MI:0469" id="EBI-1537811" refType="identity" refTypeAc="MI:0356"**/>

<**secondaryRef db="pubmed" dbAc="MI:0446" id="14755292" refType="primary-reference" refTypeAc="MI:0358"**/>

</**xref**>

</**experimentalPreparation**>

</**experimentalPreparationList**>

<**featureList**>

<**feature id="10"**>

<**names**>

<**shortLabel**>binding range</**shortLabel**>

<**fullName**>binding range</**fullName**>

</**names**>

<**xref**>

<**primaryRef db="intact" dbAc="MI:0469" id="EBI-11686233" refType="identity" refTypeAc="MI:0356"**/>

<**secondaryRef db="interpro" dbAc="MI:0449" id="IPR000980"**/>

</**xref**>

<**featureType**>

<**names**>

<**shortLabel**>binding region</**shortLabel**>

<**fullName**>binding-associated region</**fullName**>

</**names**>

<**xref**>

<**primaryRef db="psi-mi" dbAc="MI:0488" id="MI:0117" refType="identity" refTypeAc="MI:0356"**/>

<**secondaryRef db="intact" dbAc="MI:0469" id="EBI-456493" refType="identity" refTypeAc="MI:0356"**/>

<**secondaryRef db="pubmed" dbAc="MI:0446" id="14755292" refType="primary-reference" refTypeAc="MI:0358"**/>

</**xref**>

</**featureType**>

<**featureRangeList**>

<**featureRange**>

<**startStatus**>

<**names**>

<**shortLabel**>certain</**shortLabel**>

<**fullName**>certain sequence position</**fullName**>

<**alias type="synonym" typeAc="MI:1041"**>certain</**alias**>

</**names**>

<**xref**>

<**primaryRef db="psi-mi" dbAc="MI:0488" id="MI:0335" refType="identity" refTypeAc="MI:0356"**/>

<**secondaryRef db="intact" dbAc="MI:0469" id="EBI-540564" refType="identity" refTypeAc="MI:0356"**/>

<**secondaryRef db="pubmed" dbAc="MI:0446" id="14755292" refType="primary-reference" refTypeAc="MI:0358"**/>

</**xref**>

</**startStatus**>

<**begin position="667"**/>

<**endStatus**>

<**names**>

<**shortLabel**>certain</**shortLabel**>

<**fullName**>certain sequence position</**fullName**>

<**alias type="synonym" typeAc="MI:1041"**>certain</**alias**>

</**names**>

<**xref**>

<**primaryRef db="psi-mi" dbAc="MI:0488" id="MI:0335" refType="identity" refTypeAc="MI:0356"**/>

<**secondaryRef db="intact" dbAc="MI:0469" id="EBI-540564" refType="identity" refTypeAc="MI:0356"**/>

<**secondaryRef db="pubmed" dbAc="MI:0446" id="14755292" refType="primary-reference" refTypeAc="MI:0358"**/>

</**xref**>

</**endStatus**>

<**end position="782"**/>

</**featureRange**>

</**featureRangeList**>

</**feature**>

</**featureList**>

</**participant**>

</**participantList**>

<**interactionType**>

<**names**>

<**shortLabel**>direct interaction</**shortLabel**>

<**fullName**>direct interaction</**fullName**>

</**names**>

<**xref**>

<**primaryRef db="psi-mi" dbAc="MI:0488" id="MI:0407" refType="identity" refTypeAc="MI:0356"**/>

<**secondaryRef db="intact" dbAc="MI:0469" id="EBI-608833" refType="identity" refTypeAc="MI:0356"**/>

<**secondaryRef db="pubmed" dbAc="MI:0446" id="14755292" refType="primary-reference" refTypeAc="MI:0358"**/>

</**xref**>

</**interactionType**>

<**attributeList**>

<**attribute name="figure legend" nameAc="MI:0599"**>t1 f2</**attribute**>

</**attributeList**>

</**interaction**>

<**interaction id="11" imexId="IM-25084-2"**>

<**names**>

<**shortLabel**>txnip-vav2-2</**shortLabel**>

</**names**>

<**xref**>

<**primaryRef db="intact" dbAc="MI:0469" id="EBI-11686246" refType="identity" refTypeAc="MI:0356"**/>

<**secondaryRef db="imex" dbAc="MI:0670" id="IM-25084-2" refType="imex-primary" refTypeAc="MI:0662"**/>

</**xref**>

<**experimentList**>

<**experimentRef**>1</**experimentRef**>

</**experimentList**>

<**participantList**>

<**participant id="12"**>

<**interactorRef**>5</**interactorRef**>

<**biologicalRole**>

<**names**>

<**shortLabel**>unspecified role</**shortLabel**>

<**fullName**>unspecified role</**fullName**>

</**names**>

<**xref**>

<**primaryRef db="psi-mi" dbAc="MI:0488" id="MI:0499" refType="identity" refTypeAc="MI:0356"**/>

<**secondaryRef db="intact" dbAc="MI:0469" id="EBI-77781" refType="identity" refTypeAc="MI:0356"**/>

<**secondaryRef db="pubmed" dbAc="MI:0446" id="14755292" refType="primary-reference" refTypeAc="MI:0358"**/>

</**xref**>

</**biologicalRole**>

<**experimentalRoleList**>

<**experimentalRole**>

<**names**>

<**shortLabel**>neutral component</**shortLabel**>

<**fullName**>neutral component</**fullName**>

</**names**>

<**xref**>

<**primaryRef db="psi-mi" dbAc="MI:0488" id="MI:0497" refType="identity" refTypeAc="MI:0356"**/>

<**secondaryRef db="intact" dbAc="MI:0469" id="EBI-55" refType="identity" refTypeAc="MI:0356"**/>

<**secondaryRef db="pubmed" dbAc="MI:0446" id="14755292" refType="primary-reference" refTypeAc="MI:0358"**/>

</**xref**>

</**experimentalRole**>

</**experimentalRoleList**>

<**experimentalPreparationList**>

<**experimentalPreparation**>

<**names**>

<**shortLabel**>purified</**shortLabel**>

<**fullName**>purified</**fullName**>

</**names**>

<**xref**>

<**primaryRef db="psi-mi" dbAc="MI:0488" id="MI:0350" refType="identity" refTypeAc="MI:0356"**/>

<**secondaryRef db="intact" dbAc="MI:0469" id="EBI-1537811" refType="identity" refTypeAc="MI:0356"**/>

<**secondaryRef db="pubmed" dbAc="MI:0446" id="14755292" refType="primary-reference" refTypeAc="MI:0358"**/>

</**xref**>

</**experimentalPreparation**>

</**experimentalPreparationList**>

<**featureList**>

<**feature id="13"**>

<**names**>

<**shortLabel**>binding range</**shortLabel**>

<**fullName**>binding range</**fullName**>

</**names**>

<**xref**>

<**primaryRef db="intact" dbAc="MI:0469" id="EBI-11686249" refType="identity" refTypeAc="MI:0356"**/>

</**xref**>

<**featureType**>

<**names**>

<**shortLabel**>binding region</**shortLabel**>

<**fullName**>binding-associated region</**fullName**>

</**names**>

<**xref**>

<**primaryRef db="psi-mi" dbAc="MI:0488" id="MI:0117" refType="identity" refTypeAc="MI:0356"**/>

<**secondaryRef db="intact" dbAc="MI:0469" id="EBI-456493" refType="identity" refTypeAc="MI:0356"**/>

<**secondaryRef db="pubmed" dbAc="MI:0446" id="14755292" refType="primary-reference" refTypeAc="MI:0358"**/>

</**xref**>

</**featureType**>

<**featureRangeList**>

<**featureRange**>

<**startStatus**>

<**names**>

<**shortLabel**>certain</**shortLabel**>

<**fullName**>certain sequence position</**fullName**>

<**alias type="synonym" typeAc="MI:1041"**>certain</**alias**>

</**names**>

<**xref**>

<**primaryRef db="psi-mi" dbAc="MI:0488" id="MI:0335" refType="identity" refTypeAc="MI:0356"**/>

<**secondaryRef db="intact" dbAc="MI:0469" id="EBI-540564" refType="identity" refTypeAc="MI:0356"**/>

<**secondaryRef db="pubmed" dbAc="MI:0446" id="14755292" refType="primary-reference" refTypeAc="MI:0358"**/>

</**xref**>

</**startStatus**>

<**begin position="327"**/>

<**endStatus**>

<**names**>

<**shortLabel**>certain</**shortLabel**>

<**fullName**>certain sequence position</**fullName**>

<**alias type="synonym" typeAc="MI:1041"**>certain</**alias**>

</**names**>

<**xref**>

<**primaryRef db="psi-mi" dbAc="MI:0488" id="MI:0335" refType="identity" refTypeAc="MI:0356"**/>

<**secondaryRef db="intact" dbAc="MI:0469" id="EBI-540564" refType="identity" refTypeAc="MI:0356"**/>

<**secondaryRef db="pubmed" dbAc="MI:0446" id="14755292" refType="primary-reference" refTypeAc="MI:0358"**/>

</**xref**>

</**endStatus**>

<**end position="338"**/>

</**featureRange**>

</**featureRangeList**>

</**feature**>

</**featureList**>

</**participant**>

<**participant id="14"**>

<**xref**>

<**primaryRef db="interpro" dbAc="MI:0449" id="IPR000980"**/>

</**xref**>

<**interactorRef**>3</**interactorRef**>

<**biologicalRole**>

<**names**>

<**shortLabel**>unspecified role</**shortLabel**>

<**fullName**>unspecified role</**fullName**>

</**names**>

<**xref**>

<**primaryRef db="psi-mi" dbAc="MI:0488" id="MI:0499" refType="identity" refTypeAc="MI:0356"**/>

<**secondaryRef db="intact" dbAc="MI:0469" id="EBI-77781" refType="identity" refTypeAc="MI:0356"**/>

<**secondaryRef db="pubmed" dbAc="MI:0446" id="14755292" refType="primary-reference" refTypeAc="MI:0358"**/>

</**xref**>

</**biologicalRole**>

<**experimentalRoleList**>

<**experimentalRole**>

<**names**>

<**shortLabel**>neutral component</**shortLabel**>

<**fullName**>neutral component</**fullName**>

</**names**>

<**xref**>

<**primaryRef db="psi-mi" dbAc="MI:0488" id="MI:0497" refType="identity" refTypeAc="MI:0356"**/>

<**secondaryRef db="intact" dbAc="MI:0469" id="EBI-55" refType="identity" refTypeAc="MI:0356"**/>

<**secondaryRef db="pubmed" dbAc="MI:0446" id="14755292" refType="primary-reference" refTypeAc="MI:0358"**/>

</**xref**>

</**experimentalRole**>

</**experimentalRoleList**>

<**experimentalPreparationList**>

<**experimentalPreparation**>

<**names**>

<**shortLabel**>purified</**shortLabel**>

<**fullName**>purified</**fullName**>

</**names**>

<**xref**>

<**primaryRef db="psi-mi" dbAc="MI:0488" id="MI:0350" refType="identity" refTypeAc="MI:0356"**/>

<**secondaryRef db="intact" dbAc="MI:0469" id="EBI-1537811" refType="identity" refTypeAc="MI:0356"**/>

<**secondaryRef db="pubmed" dbAc="MI:0446" id="14755292" refType="primary-reference" refTypeAc="MI:0358"**/>

</**xref**>

</**experimentalPreparation**>

</**experimentalPreparationList**>

<**featureList**>

<**feature id="15"**>

<**names**>

<**shortLabel**>binding range</**shortLabel**>

<**fullName**>binding range</**fullName**>

</**names**>

<**xref**>

<**primaryRef db="intact" dbAc="MI:0469" id="EBI-11686252" refType="identity" refTypeAc="MI:0356"**/>

</**xref**>

<**featureType**>

<**names**>

<**shortLabel**>binding region</**shortLabel**>

<**fullName**>binding-associated region</**fullName**>

</**names**>

<**xref**>

<**primaryRef db="psi-mi" dbAc="MI:0488" id="MI:0117" refType="identity" refTypeAc="MI:0356"**/>

<**secondaryRef db="intact" dbAc="MI:0469" id="EBI-456493" refType="identity" refTypeAc="MI:0356"**/>

<**secondaryRef db="pubmed" dbAc="MI:0446" id="14755292" refType="primary-reference" refTypeAc="MI:0358"**/>

</**xref**>

</**featureType**>

<**featureRangeList**>

<**featureRange**>

<**startStatus**>

<**names**>

<**shortLabel**>certain</**shortLabel**>

<**fullName**>certain sequence position</**fullName**>

<**alias type="synonym" typeAc="MI:1041"**>certain</**alias**>

</**names**>

<**xref**>

<**primaryRef db="psi-mi" dbAc="MI:0488" id="MI:0335" refType="identity" refTypeAc="MI:0356"**/>

<**secondaryRef db="intact" dbAc="MI:0469" id="EBI-540564" refType="identity" refTypeAc="MI:0356"**/>

<**secondaryRef db="pubmed" dbAc="MI:0446" id="14755292" refType="primary-reference" refTypeAc="MI:0358"**/>

</**xref**>

</**startStatus**>

<**begin position="667"**/>

<**endStatus**>

<**names**>

<**shortLabel**>certain</**shortLabel**>

<**fullName**>certain sequence position</**fullName**>

<**alias type="synonym" typeAc="MI:1041"**>certain</**alias**>

</**names**>

<**xref**>

<**primaryRef db="psi-mi" dbAc="MI:0488" id="MI:0335" refType="identity" refTypeAc="MI:0356"**/>

<**secondaryRef db="intact" dbAc="MI:0469" id="EBI-540564" refType="identity" refTypeAc="MI:0356"**/>

<**secondaryRef db="pubmed" dbAc="MI:0446" id="14755292" refType="primary-reference" refTypeAc="MI:0358"**/>

</**xref**>

</**endStatus**>

<**end position="782"**/>

</**featureRange**>

</**featureRangeList**>

</**feature**>

</**featureList**>

</**participant**>

</**participantList**>

<**interactionType**>

<**names**>

<**shortLabel**>direct interaction</**shortLabel**>

<**fullName**>direct interaction</**fullName**>

</**names**>

<**xref**>

<**primaryRef db="psi-mi" dbAc="MI:0488" id="MI:0407" refType="identity" refTypeAc="MI:0356"**/>

<**secondaryRef db="intact" dbAc="MI:0469" id="EBI-608833" refType="identity" refTypeAc="MI:0356"**/>

<**secondaryRef db="pubmed" dbAc="MI:0446" id="14755292" refType="primary-reference" refTypeAc="MI:0358"**/>

</**xref**>

</**interactionType**>

<**parameterList**>

<**parameter term="kd" termAc="MI:0646" unit="molar" unitAc="MI:0648" base="10" exponent="-6" factor="14"**/>

</**parameterList**>

<**attributeList**>

<**attribute name="figure legend" nameAc="MI:0599"**>f1c</**attribute**>

</**attributeList**>

</**interaction**>

<**interaction id="16" imexId="IM-25084-3"**>

<**names**>

<**shortLabel**>src-txnip-1</**shortLabel**>

</**names**>

<**xref**>

<**primaryRef db="intact" dbAc="MI:0469" id="EBI-11686298" refType="identity" refTypeAc="MI:0356"**/>

<**secondaryRef db="imex" dbAc="MI:0670" id="IM-25084-3" refType="imex-primary" refTypeAc="MI:0662"**/>

</**xref**>

<**experimentList**>

<**experimentRef**>1</**experimentRef**>

</**experimentList**>

<**participantList**>

<**participant id="17"**>

<**interactorRef**>5</**interactorRef**>

<**biologicalRole**>

<**names**>

<**shortLabel**>unspecified role</**shortLabel**>

<**fullName**>unspecified role</**fullName**>

</**names**>

<**xref**>

<**primaryRef db="psi-mi" dbAc="MI:0488" id="MI:0499" refType="identity" refTypeAc="MI:0356"**/>

<**secondaryRef db="intact" dbAc="MI:0469" id="EBI-77781" refType="identity" refTypeAc="MI:0356"**/>

<**secondaryRef db="pubmed" dbAc="MI:0446" id="14755292" refType="primary-reference" refTypeAc="MI:0358"**/>

</**xref**>

</**biologicalRole**>

<**experimentalRoleList**>

<**experimentalRole**>

<**names**>

<**shortLabel**>neutral component</**shortLabel**>

<**fullName**>neutral component</**fullName**>

</**names**>

<**xref**>

<**primaryRef db="psi-mi" dbAc="MI:0488" id="MI:0497" refType="identity" refTypeAc="MI:0356"**/>

<**secondaryRef db="intact" dbAc="MI:0469" id="EBI-55" refType="identity" refTypeAc="MI:0356"**/>

<**secondaryRef db="pubmed" dbAc="MI:0446" id="14755292" refType="primary-reference" refTypeAc="MI:0358"**/>

</**xref**>

</**experimentalRole**>

</**experimentalRoleList**>

<**experimentalPreparationList**>

<**experimentalPreparation**>

<**names**>

<**shortLabel**>purified</**shortLabel**>

<**fullName**>purified</**fullName**>

</**names**>

<**xref**>

<**primaryRef db="psi-mi" dbAc="MI:0488" id="MI:0350" refType="identity" refTypeAc="MI:0356"**/>

<**secondaryRef db="intact" dbAc="MI:0469" id="EBI-1537811" refType="identity" refTypeAc="MI:0356"**/>

<**secondaryRef db="pubmed" dbAc="MI:0446" id="14755292" refType="primary-reference" refTypeAc="MI:0358"**/>

</**xref**>

</**experimentalPreparation**>

</**experimentalPreparationList**>

<**featureList**>

<**feature id="18"**>

<**names**>

<**shortLabel**>binding range</**shortLabel**>

<**fullName**>binding range</**fullName**>

</**names**>

<**xref**>

<**primaryRef db="intact" dbAc="MI:0469" id="EBI-11686302" refType="identity" refTypeAc="MI:0356"**/>

</**xref**>

<**featureType**>

<**names**>

<**shortLabel**>binding region</**shortLabel**>

<**fullName**>binding-associated region</**fullName**>

</**names**>

<**xref**>

<**primaryRef db="psi-mi" dbAc="MI:0488" id="MI:0117" refType="identity" refTypeAc="MI:0356"**/>

<**secondaryRef db="intact" dbAc="MI:0469" id="EBI-456493" refType="identity" refTypeAc="MI:0356"**/>

<**secondaryRef db="pubmed" dbAc="MI:0446" id="14755292" refType="primary-reference" refTypeAc="MI:0358"**/>

</**xref**>

</**featureType**>

<**featureRangeList**>

<**featureRange**>

<**startStatus**>

<**names**>

<**shortLabel**>certain</**shortLabel**>

<**fullName**>certain sequence position</**fullName**>

<**alias type="synonym" typeAc="MI:1041"**>certain</**alias**>

</**names**>

<**xref**>

<**primaryRef db="psi-mi" dbAc="MI:0488" id="MI:0335" refType="identity" refTypeAc="MI:0356"**/>

<**secondaryRef db="intact" dbAc="MI:0469" id="EBI-540564" refType="identity" refTypeAc="MI:0356"**/>

<**secondaryRef db="pubmed" dbAc="MI:0446" id="14755292" refType="primary-reference" refTypeAc="MI:0358"**/>

</**xref**>

</**startStatus**>

<**begin position="327"**/>

<**endStatus**>

<**names**>

<**shortLabel**>certain</**shortLabel**>

<**fullName**>certain sequence position</**fullName**>

<**alias type="synonym" typeAc="MI:1041"**>certain</**alias**>

</**names**>

<**xref**>

<**primaryRef db="psi-mi" dbAc="MI:0488" id="MI:0335" refType="identity" refTypeAc="MI:0356"**/>

<**secondaryRef db="intact" dbAc="MI:0469" id="EBI-540564" refType="identity" refTypeAc="MI:0356"**/>

<**secondaryRef db="pubmed" dbAc="MI:0446" id="14755292" refType="primary-reference" refTypeAc="MI:0358"**/>

</**xref**>

</**endStatus**>

<**end position="338"**/>

</**featureRange**>

</**featureRangeList**>

</**feature**>

</**featureList**>

</**participant**>

<**participant id="19"**>

<**interactorRef**>4</**interactorRef**>

<**biologicalRole**>

<**names**>

<**shortLabel**>unspecified role</**shortLabel**>

<**fullName**>unspecified role</**fullName**>

</**names**>

<**xref**>

<**primaryRef db="psi-mi" dbAc="MI:0488" id="MI:0499" refType="identity" refTypeAc="MI:0356"**/>

<**secondaryRef db="intact" dbAc="MI:0469" id="EBI-77781" refType="identity" refTypeAc="MI:0356"**/>

<**secondaryRef db="pubmed" dbAc="MI:0446" id="14755292" refType="primary-reference" refTypeAc="MI:0358"**/>

</**xref**>

</**biologicalRole**>

<**experimentalRoleList**>

<**experimentalRole**>

<**names**>

<**shortLabel**>neutral component</**shortLabel**>

<**fullName**>neutral component</**fullName**>

</**names**>

<**xref**>

<**primaryRef db="psi-mi" dbAc="MI:0488" id="MI:0497" refType="identity" refTypeAc="MI:0356"**/>

<**secondaryRef db="intact" dbAc="MI:0469" id="EBI-55" refType="identity" refTypeAc="MI:0356"**/>

<**secondaryRef db="pubmed" dbAc="MI:0446" id="14755292" refType="primary-reference" refTypeAc="MI:0358"**/>

</**xref**>

</**experimentalRole**>

</**experimentalRoleList**>

<**experimentalPreparationList**>

<**experimentalPreparation**>

<**names**>

<**shortLabel**>purified</**shortLabel**>

<**fullName**>purified</**fullName**>

</**names**>

<**xref**>

<**primaryRef db="psi-mi" dbAc="MI:0488" id="MI:0350" refType="identity" refTypeAc="MI:0356"**/>

<**secondaryRef db="intact" dbAc="MI:0469" id="EBI-1537811" refType="identity" refTypeAc="MI:0356"**/>

<**secondaryRef db="pubmed" dbAc="MI:0446" id="14755292" refType="primary-reference" refTypeAc="MI:0358"**/>

</**xref**>

</**experimentalPreparation**>

</**experimentalPreparationList**>

<**featureList**>

<**feature id="20"**>

<**names**>

<**shortLabel**>binding range</**shortLabel**>

<**fullName**>binding range</**fullName**>

</**names**>

<**xref**>

<**primaryRef db="intact" dbAc="MI:0469" id="EBI-11686305" refType="identity" refTypeAc="MI:0356"**/>

<**secondaryRef db="interpro" dbAc="MI:0449" id="IPR000980"**/>

</**xref**>

<**featureType**>

<**names**>

<**shortLabel**>binding region</**shortLabel**>

<**fullName**>binding-associated region</**fullName**>

</**names**>

<**xref**>

<**primaryRef db="psi-mi" dbAc="MI:0488" id="MI:0117" refType="identity" refTypeAc="MI:0356"**/>

<**secondaryRef db="intact" dbAc="MI:0469" id="EBI-456493" refType="identity" refTypeAc="MI:0356"**/>

<**secondaryRef db="pubmed" dbAc="MI:0446" id="14755292" refType="primary-reference" refTypeAc="MI:0358"**/>

</**xref**>

</**featureType**>

<**featureRangeList**>

<**featureRange**>

<**startStatus**>

<**names**>

<**shortLabel**>certain</**shortLabel**>

<**fullName**>certain sequence position</**fullName**>

<**alias type="synonym" typeAc="MI:1041"**>certain</**alias**>

</**names**>

<**xref**>

<**primaryRef db="psi-mi" dbAc="MI:0488" id="MI:0335" refType="identity" refTypeAc="MI:0356"**/>

<**secondaryRef db="intact" dbAc="MI:0469" id="EBI-540564" refType="identity" refTypeAc="MI:0356"**/>

<**secondaryRef db="pubmed" dbAc="MI:0446" id="14755292" refType="primary-reference" refTypeAc="MI:0358"**/>

</**xref**>

</**startStatus**>

<**begin position="145"**/>

<**endStatus**>

<**names**>

<**shortLabel**>certain</**shortLabel**>

<**fullName**>certain sequence position</**fullName**>

<**alias type="synonym" typeAc="MI:1041"**>certain</**alias**>

</**names**>

<**xref**>

<**primaryRef db="psi-mi" dbAc="MI:0488" id="MI:0335" refType="identity" refTypeAc="MI:0356"**/>

<**secondaryRef db="intact" dbAc="MI:0469" id="EBI-540564" refType="identity" refTypeAc="MI:0356"**/>

<**secondaryRef db="pubmed" dbAc="MI:0446" id="14755292" refType="primary-reference" refTypeAc="MI:0358"**/>

</**xref**>

</**endStatus**>

<**end position="250"**/>

</**featureRange**>

</**featureRangeList**>

</**feature**>

</**featureList**>

</**participant**>

</**participantList**>

<**interactionType**>

<**names**>

<**shortLabel**>direct interaction</**shortLabel**>

<**fullName**>direct interaction</**fullName**>

</**names**>

<**xref**>

<**primaryRef db="psi-mi" dbAc="MI:0488" id="MI:0407" refType="identity" refTypeAc="MI:0356"**/>

<**secondaryRef db="intact" dbAc="MI:0469" id="EBI-608833" refType="identity" refTypeAc="MI:0356"**/>

<**secondaryRef db="pubmed" dbAc="MI:0446" id="14755292" refType="primary-reference" refTypeAc="MI:0358"**/>

</**xref**>

</**interactionType**>

<**parameterList**>

<**parameter term="kd" termAc="MI:0646" unit="molar" unitAc="MI:0648" base="10" exponent="-6" factor="12"**/>

</**parameterList**>

<**attributeList**>

<**attribute name="figure legend" nameAc="MI:0599"**>f1B</**attribute**>

</**attributeList**>

</**interaction**>

</**interactionList**>

</**entry**>

</**entrySet**>
