# Supplementary material for: Encompassing new use cases - level 3.0 of the HUPO-PSI format for molecular interactions
Source: BMC Bioinformatics. 2018 Apr 11;19:134. doi: 10.1186/s12859-018-2118-1 (PMC5896046; doi:10.1186/s12859-018-2118-1)
Supplement: Supplementary file 2 — Representation of a negative feature range (use case 1.3a). (https://github.com/HUPO-PSI/miXML/blob/master/3.0/pub/Appendix%203.docx). (DOCX 27 kb) [file 12859_2018_2118_MOESM2_ESM.docx]

**Negative feature range**

This will enable the description of genomic loci in features.

PMID:21668996

Describing binding region E box Y, coordinates -912 to -907 of the BRCA1 promoter region,

**<featureList>**

**<feature id="7">**

**<names>**

**<shortLabel>e box y</shortLabel>**

**</names>**

**<xref>**

**<primaryRef db="intact" dbAc="MI:0469" id="EBI-6143256" refType="identity" refTypeAc="MI:0356"/>**

**</xref>**

**<featureType>**

**<names>**

**<shortLabel>binding region</shortLabel>**

**<fullName>binding-associated region</fullName>**

**</names>**

**<xref>**

**<primaryRef db="psi-mi" dbAc="MI:0488" id="MI:0117" refType="identity" refTypeAc="MI:0356"/>**

**</xref>**

**</featureType>**

**<featureRangeList>**

**<featureRange>**

**<startStatus>**

**<names>**

**<shortLabel>certain</shortLabel>**

**<fullName>certain sequence position</fullName>**

**<alias type="synonym" typeAc="MI:1041">certain</alias>**

**</names>**

**<xref>**

**<primaryRef db="psi-mi" dbAc="MI:0488" id="MI:0335" refType="identity" refTypeAc="MI:0356"/>**

**</xref>**

**</startStatus>**

**<begin position="-912"/>**

**<endStatus>**

**<names>**

**<shortLabel>certain</shortLabel>**

**<fullName>certain sequence position</fullName>**

**<alias type="synonym" typeAc="MI:1041">certain</alias>**

**</names>**

**<xref>**

**<primaryRef db="psi-mi" dbAc="MI:0488" id="MI:0335" refType="identity" refTypeAc="MI:0356"/>**

**</xref>**

**</endStatus>**

**<end position="-907"/>**

**</featureRange>**

**</featureRangeList>**

**</feature>**

**</featureList>**

File:

*<?***xml version="1.0" encoding="UTF-8"***?>*

<**entrySet xmlns:xsi="http://www.w3.org/2001/XMLSchema-instance"**

**xmlns="http://psi.hupo.org/mi/mif300"**

**xsi:schemaLocation="http://psi.hupo.org/mi/mif300 https://raw.githubusercontent.com/HUPO-PSI/miXML/master/3.0/src/MIF300.xsd"**

**level="3" version="0" minorVersion="0"**>

<**entry**>

<**experimentList**>

<**experimentDescription id="1"**>

<**names**>

<**fullName**>c-Myc activates BRCA1 gene expression through distal promoter elements in breast cancer cells.</**fullName**>

</**names**>

<**bibref**>

<**xref**>

<**primaryRef db="pubmed" dbAc="MI:0446" id="21668996" refType="primary-reference" refTypeAc="MI:0358"**/>

</**xref**>

<**attributeList**>

<**attribute name="publication title" nameAc="MI:1091**

c-Myc activates BRCA1 gene expression through distal promoter elements in breast cancer cells

</**attribute**>

<**attribute name="journal" nameAc="MI:0885"**>BMC Cancer</**attribute**>

<**attribute name="publication year" nameAc="MI:0886"**>2011</**attribute**>

<**attribute name="curation depth" nameAc="MI:0955"**>imex curation</**attribute**>

<**attribute name="imex curation" nameAc="MI:0959"**/>

<**attribute name="author-list" nameAc="MI:0636"**>Chen Y., Xu J., Borowicz S., Collins C., Huo D., Olopade O.I.</**attribute**>

<**attribute name="contact-email" nameAc="MI:0634"**>folopade@bsd.uchicago.edu</**attribute**>

</**attributeList**>

</**bibref**>

<**xref**>

<**primaryRef db="pubmed" dbAc="MI:0446" id="21668996" refType="primary-reference" refTypeAc="MI:0358"**/>

</**xref**>

<**hostOrganismList**>

<**hostOrganism ncbiTaxId="9606"**>

<**names**>

<**shortLabel**>human-mda_mb_231</**shortLabel**>

<**fullName**>Homo sapiens Caucasian breast adenocarcinoma</**fullName**>

</**names**>

<**cellType**>

<**names**>

<**shortLabel**>mda_mb_231</**shortLabel**>

<**fullName**>Human Caucasian breast adenocarcinoma</**fullName**>

</**names**>

<**xref**>

<**primaryRef db="cabri" dbAc="MI:0246" id="ECACC 92020424" refType="identity" refTypeAc="MI:0356"**/>

<**secondaryRef db="intact" dbAc="MI:0469" id="IA:0581" refType="identity" refTypeAc="MI:0356"**/>

<**secondaryRef db="mint" dbAc="MI:0471" id="MINT-8415708" refType="identity" refTypeAc="MI:0356"**/>

<**secondaryRef db="intact" dbAc="MI:0469" id="EBI-1199826" refType="identity" refTypeAc="MI:0356"**/>

</**xref**>

</**cellType**>

</**hostOrganism**>

</**hostOrganismList**>

<**interactionDetectionMethod**>

<**names**>

<**shortLabel**>ch-ip</**shortLabel**>

<**fullName**>chromatin immunoprecipitation assay</**fullName**>

</**names**>

<**xref**>

<**primaryRef db="psi-mi" dbAc="MI:0488" id="MI:0402" refType="identity" refTypeAc="MI:0356"**/>

</**xref**>

</**interactionDetectionMethod**>

<**participantIdentificationMethod**>

<**names**>

<**shortLabel**>primer specific pcr</**shortLabel**>

<**fullName**>primer specific pcr</**fullName**>

</**names**>

<**xref**>

<**primaryRef db="psi-mi" dbAc="MI:0488" id="MI:0088" refType="identity" refTypeAc="MI:0356"**/>

</**xref**>

</**participantIdentificationMethod**>

</**experimentDescription**>

</**experimentList**>

<**interactorList**>

<**interactor id="2"**>

<**names**>

<**shortLabel**>brca1_human_gene</**shortLabel**>

<**fullName**>breast cancer 1, early onset</**fullName**>

</**names**>

<**xref**>

<**primaryRef db="ensembl" dbAc="MI:0476" id="ENSG00000012048" refType="identity" refTypeAc="MI:0356"**/>

</**xref**>

<**interactorType**>

<**names**>

<**shortLabel**>gene</**shortLabel**>

<**fullName**>gene</**fullName**>

</**names**>

<**xref**>

<**primaryRef db="psi-mi" dbAc="MI:0488" id="MI:0250" refType="identity" refTypeAc="MI:0356"**/>

</**xref**>

</**interactorType**>

<**organism ncbiTaxId="9606"**>

<**names**>

<**shortLabel**>human</**shortLabel**>

<**fullName**>Homo sapiens</**fullName**>

<**alias type="synonym" typeAc="MI:1041"**>Human</**alias**>

</**names**>

</**organism**>

</**interactor**>

<**interactor id="3"**>

<**names**>

<**shortLabel**>myc_human</**shortLabel**>

<**fullName**>Myc proto-oncogene protein</**fullName**>

</**names**>

<**xref**>

<**primaryRef db="uniprotkb" dbAc="MI:0486" id="P01106" version="SP_118" refType="identity" refTypeAc="MI:0356"**/>

</**xref**>

<**interactorType**>

<**names**>

<**shortLabel**>protein</**shortLabel**>

<**fullName**>protein</**fullName**>

</**names**>

<**xref**>

<**primaryRef db="psi-mi" dbAc="MI:0488" id="MI:0326" refType="identity" refTypeAc="MI:0356"**/>

</**xref**>

</**interactorType**>

<**organism ncbiTaxId="9606"**>

<**names**>

<**shortLabel**>human</**shortLabel**>

<**fullName**>Homo sapiens</**fullName**>

<**alias type="synonym" typeAc="MI:1041"**>Human</**alias**>

</**names**>

</**organism**>

</**interactor**>

</**interactorList**>

<**interactionList**>

<**interaction id="4"**>

<**names**>

<**shortLabel**>myc-brca1-1</**shortLabel**>

</**names**>

<**xref**>

<**primaryRef db="intact" dbAc="MI:0469" id="EBI-6142659" refType="identity" refTypeAc="MI:0356"**/>

</**xref**>

<**experimentList**>

<**experimentRef**>1</**experimentRef**>

</**experimentList**>

<**participantList**>

<**participant id="5"**>

<**interactorRef**>3</**interactorRef**>

<**biologicalRole**>

<**names**>

<**shortLabel**>unspecified role</**shortLabel**>

<**fullName**>unspecified role</**fullName**>

</**names**>

<**xref**>

<**primaryRef db="psi-mi" dbAc="MI:0488" id="MI:0499" refType="identity" refTypeAc="MI:0356"**/>

</**xref**>

</**biologicalRole**>

<**experimentalRoleList**>

<**experimentalRole**>

<**names**>

<**shortLabel**>bait</**shortLabel**>

<**fullName**>bait</**fullName**>

</**names**>

<**xref**>

<**primaryRef db="psi-mi" dbAc="MI:0488" id="MI:0496" refType="identity" refTypeAc="MI:0356"**/>

</**xref**>

</**experimentalRole**>

</**experimentalRoleList**>

</**participant**>

<**participant id="6"**>

<**interactorRef**>2</**interactorRef**>

<**biologicalRole**>

<**names**>

<**shortLabel**>unspecified role</**shortLabel**>

<**fullName**>unspecified role</**fullName**>

</**names**>

<**xref**>

<**primaryRef db="psi-mi" dbAc="MI:0488" id="MI:0499" refType="identity" refTypeAc="MI:0356"**/>

</**xref**>

</**biologicalRole**>

<**experimentalRoleList**>

<**experimentalRole**>

<**names**>

<**shortLabel**>prey</**shortLabel**>

<**fullName**>prey</**fullName**>

</**names**>

<**xref**>

<**primaryRef db="psi-mi" dbAc="MI:0488" id="MI:0498" refType="identity" refTypeAc="MI:0356"**/>

</**xref**>

</**experimentalRole**>

</**experimentalRoleList**>

<**featureList**>

<**feature id="7"**>

<**names**>

<**shortLabel**>e box y</**shortLabel**>

</**names**>

<**xref**>

<**primaryRef db="intact" dbAc="MI:0469" id="EBI-6143256" refType="identity" refTypeAc="MI:0356"**/>

</**xref**>

<**featureType**>

<**names**>

<**shortLabel**>binding region</**shortLabel**>

<**fullName**>binding-associated region</**fullName**>

</**names**>

<**xref**>

<**primaryRef db="psi-mi" dbAc="MI:0488" id="MI:0117" refType="identity" refTypeAc="MI:0356"**/>

</**xref**>

</**featureType**>

<**featureRangeList**>

<**featureRange**>

<**startStatus**>

<**names**>

<**shortLabel**>certain</**shortLabel**>

<**fullName**>certain sequence position</**fullName**>

<**alias type="synonym" typeAc="MI:1041"**>certain</**alias**>

</**names**>

<**xref**>

<**primaryRef db="psi-mi" dbAc="MI:0488" id="MI:0335" refType="identity" refTypeAc="MI:0356"**/>

</**xref**>

</**startStatus**>

<**begin position="-912"**/>

<**endStatus**>

<**names**>

<**shortLabel**>certain</**shortLabel**>

<**fullName**>certain sequence position</**fullName**>

<**alias type="synonym" typeAc="MI:1041"**>certain</**alias**>

</**names**>

<**xref**>

<**primaryRef db="psi-mi" dbAc="MI:0488" id="MI:0335" refType="identity" refTypeAc="MI:0356"**/>

</**xref**>

</**endStatus**>

<**end position="-907"**/>

</**featureRange**>

</**featureRangeList**>

</**feature**>

<**feature id="8"**>

<**names**>

<**shortLabel**>e box x</**shortLabel**>

</**names**>

<**xref**>

<**primaryRef db="intact" dbAc="MI:0469" id="EBI-6143254" refType="identity" refTypeAc="MI:0356"**/>

</**xref**>

<**featureType**>

<**names**>

<**shortLabel**>binding region</**shortLabel**>

<**fullName**>binding-associated region</**fullName**>

</**names**>

<**xref**>

<**primaryRef db="psi-mi" dbAc="MI:0488" id="MI:0117" refType="identity" refTypeAc="MI:0356"**/>

</**xref**>

</**featureType**>

<**featureRangeList**>

<**featureRange**>

<**startStatus**>

<**names**>

<**shortLabel**>undetermined</**shortLabel**>

<**fullName**>undetermined sequence position</**fullName**>

</**names**>

<**xref**>

<**primaryRef db="psi-mi" dbAc="MI:0488" id="MI:0339" refType="identity" refTypeAc="MI:0356"**/>

</**xref**>

</**startStatus**>

<**endStatus**>

<**names**>

<**shortLabel**>undetermined</**shortLabel**>

<**fullName**>undetermined sequence position</**fullName**>

</**names**>

<**xref**>

<**primaryRef db="psi-mi" dbAc="MI:0488" id="MI:0339" refType="identity" refTypeAc="MI:0356"**/>

</**xref**>

</**endStatus**>

</**featureRange**>

</**featureRangeList**>

</**feature**>

</**featureList**>

</**participant**>

</**participantList**>

<**interactionType**>

<**names**>

<**shortLabel**>association</**shortLabel**>

<**fullName**>association</**fullName**>

</**names**>

<**xref**>

<**primaryRef db="psi-mi" dbAc="MI:0488" id="MI:0914" refType="identity" refTypeAc="MI:0356"**/>

</**xref**>

</**interactionType**>

<**attributeList**>

<**attribute name="figure legend" nameAc="MI:0599"**>Figure 4A</**attribute**>

</**attributeList**>

</**interaction**>

</**interactionList**>

</**entry**>

</**entrySet**>
