# Supplementary material for: Encompassing new use cases - level 3.0 of the HUPO-PSI format for molecular interactions
Source: BMC Bioinformatics. 2018 Apr 11;19:134. doi: 10.1186/s12859-018-2118-1 (PMC5896046; doi:10.1186/s12859-018-2118-1)
Supplement: Supplementary file 3 — Representation of the sequence change caused by introduction of a mutation (use case 1.3b). (https://github.com/HUPO-PSI/miXML/blob/master/3.0/pub/Appendix%204.docx). (DOCX 38 kb) [file 12859_2018_2118_MOESM3_ESM.docx]

**Representation of the sequence change caused by introduction of a mutation**

The actual sequence change of a mutation is systematically captured

PMID: **10481074**

A proline to arginine mutation at position 639 in a sequence

<**feature id="10"**>

<**names**>

<**shortLabel**>pro639arg</**shortLabel**>

</**names**>

<**xref**>

<**primaryRef db="intact" dbAc="MI:0469" id="EBI-12553596" refType="identity" refTypeAc="MI:0356"**/>

</**xref**>

<**featureType**>

<**names**>

<**shortLabel**>mutation</**shortLabel**>

<**fullName**>mutation</**fullName**>

</**names**>

<**xref**>

<**primaryRef db="psi-mi" dbAc="MI:0488" id="MI:0118" refType="identity" refTypeAc="MI:0356"**/>

<**secondaryRef db="intact" dbAc="MI:0469" id="EBI-456558" refType="identity" refTypeAc="MI:0356"**/>

<**secondaryRef db="pubmed" dbAc="MI:0446" id="14755292" refType="primary-reference" refTypeAc="MI:0358"**/>

</**xref**>

</**featureType**>

<**featureRangeList**>

<**featureRange**>

<**startStatus**>

<**names**>

<**shortLabel**>certain</**shortLabel**>

<**fullName**>certain sequence position</**fullName**>

<**alias type="synonym" typeAc="MI:1041"**>certain</**alias**>

</**names**>

<**xref**>

<**primaryRef db="psi-mi" dbAc="MI:0488" id="MI:0335" refType="identity" refTypeAc="MI:0356"**/>

<**secondaryRef db="intact" dbAc="MI:0469" id="EBI-540564" refType="identity" refTypeAc="MI:0356"**/>

<**secondaryRef db="pubmed" dbAc="MI:0446" id="14755292" refType="primary-reference" refTypeAc="MI:0358"**/>

</**xref**>

</**startStatus**>

<**begin position="639"**/>

<**endStatus**>

<**names**>

<**shortLabel**>certain</**shortLabel**>

<**fullName**>certain sequence position</**fullName**>

<**alias type="synonym" typeAc="MI:1041"**>certain</**alias**>

</**names**>

<**xref**>

<**primaryRef db="psi-mi" dbAc="MI:0488" id="MI:0335" refType="identity" refTypeAc="MI:0356"**/>

<**secondaryRef db="intact" dbAc="MI:0469" id="EBI-540564" refType="identity" refTypeAc="MI:0356"**/>

<**secondaryRef db="pubmed" dbAc="MI:0446" id="14755292" refType="primary-reference" refTypeAc="MI:0358"**/>

</**xref**>

</**endStatus**>

<**end position="639"**/>

<**resultingSequence**>

<**originalSequence**>P</**originalSequence**>

<**newSequence**>R</**newSequence**>

</**resultingSequence**>

</**featureRange**>

</**featureRangeList**>

</**feature**>

File:

*<?***xml version='1.0' encoding='UTF-8'***?>*

<**entrySet xmlns:xsi="http://www.w3.org/2001/XMLSchema-instance" xmlns="http://psi.hupo.org/mi/mif300"**

**xsi:schemaLocation="http://psi.hupo.org/mi/mif300 https://raw.githubusercontent.com/HUPO-PSI/miXML/master/3.0/src/MIF300.xsd"**

**level="3" version="0" minorVersion="0"**>

<**entry**>

<**source releaseDate="2017-05-18"**>

<**names**>

<**shortLabel**>IntAct</**shortLabel**>

<**fullName**>European Bioinformatics Institute</**fullName**>

<**alias type="synonym" typeAc="MI:1041"**>IntAct</**alias**>

</**names**>

<**bibref**>

<**xref**>

<**primaryRef db="pubmed" dbAc="MI:0446" id="14681455" refType="primary-reference" refTypeAc="MI:0358"**/>

</**xref**>

</**bibref**>

<**xref**>

<**primaryRef db="psi-mi" dbAc="MI:0488" id="MI:0469" refType="identity" refTypeAc="MI:0356"**/>

<**secondaryRef db="intact" dbAc="MI:0469" id="EBI-10" refType="identity" refTypeAc="MI:0356"**/>

<**secondaryRef db="pubmed" dbAc="MI:0446" id="14681455" refType="primary-reference" refTypeAc="MI:0358"**/>

<**secondaryRef db="pubmed" dbAc="MI:0446" id="22121220" refType="method reference" refTypeAc="MI:0357"**/>

<**secondaryRef db="pubmed" dbAc="MI:0446" id="19850723" refType="method reference" refTypeAc="MI:0357"**/>

</**xref**>

<**attributeList**>

<**attribute name="url" nameAc="MI:0614"**>http://www.ebi.ac.uk/</**attribute**>

<**attribute name="search-url" nameAc="MI:0615"**>http://www.ebi.ac.uk/intact/query/${ac}</**attribute**>

<**attribute name="id-validation-regexp" nameAc="MI:0628"**>EBI-[0-9]+|IA:[0-9]+</**attribute**>

<**attribute name="definition"**>INTerAction database (IntAct) provides an open source database and toolkit for the storage, presentation and analysis of molecular interactions.</**attribute**>

<**attribute name="url" nameAc="MI:0614"**>http://www.ebi.ac.uk/intact</**attribute**>

<**attribute name="postaladdress"**>European Bioinformatics Institute; Wellcome Trust Genome Campus; Hinxton, Cambridge; CB10 1SD; United Kingdom</**attribute**>

<**attribute name="url" nameAc="MI:0614"**>http://www.ebi.ac.uk/intact/</**attribute**>

</**attributeList**>

</**source**>

<**experimentList**>

<**experimentDescription id="1"**>

<**names**>

<**fullName**>A GSK3-binding peptide from FRAT1 selectively inhibits the GSK3-catalysed phosphorylation of axin and beta-catenin.</**fullName**>

</**names**>

<**bibref**>

<**xref**>

<**primaryRef db="pubmed" dbAc="MI:0446" id="10481074" refType="primary-reference" refTypeAc="MI:0358"**/>

<**secondaryRef db="intact" dbAc="MI:0469" id="EBI-11789085" refType="identity" refTypeAc="MI:0356"**/>

<**secondaryRef db="imex" dbAc="MI:0670" id="IM-25329" refType="imex-primary" refTypeAc="MI:0662"**/>

</**xref**>

<**attributeList**>

<**attribute name="publication title" nameAc="MI:1091"**>A GSK3-binding peptide from FRAT1 selectively inhibits the GSK3-catalysed phosphorylation of axin and beta-catenin.</**attribute**>

<**attribute name="journal" nameAc="MI:0885"**>FEBS letters</**attribute**>

<**attribute name="publication year" nameAc="MI:0886"**>1999</**attribute**>

<**attribute name="curation depth" nameAc="MI:0955"**>imex curation</**attribute**>

<**attribute name="imex curation" nameAc="MI:0959"**/>

<**attribute name="author-list" nameAc="MI:0636"**>Thomas GM., Frame S., Goedert M., Nathke I., Polakis P., Cohen P.</**attribute**>

<**attribute name="contact-email" nameAc="MI:0634"**>pcohen@bad.dundee.ac.uk</**attribute**>

<**attribute name="comment" nameAc="MI:0612"**>

Assays involving GS-1 were not added as the peptide did not match GS-1 of any species. The phosphorylation of Tau by GSK was not added as the exact identity and origins of GSK are not known.

</**attribute**>

<**attribute name="full coverage" nameAc="MI:0957"**>Only protein-protein interactions</**attribute**>

<**attribute name="imex curation" nameAc="MI:0959"**>imex curation</**attribute**>

<**attribute name="author-announcement"**>20-Dec-2016: Contacted by IntAct-Help.</**attribute**>

</**attributeList**>

</**bibref**>

<**xref**>

<**primaryRef db="pubmed" dbAc="MI:0446" id="10481074" refType="primary-reference" refTypeAc="MI:0358"**/>

<**secondaryRef db="imex" dbAc="MI:0670" id="IM-25329" refType="imex-primary" refTypeAc="MI:0662"**/>

</**xref**>

<**hostOrganismList**>

<**hostOrganism ncbiTaxId="-1"**>

<**names**>

<**shortLabel**>in vitro</**shortLabel**>

<**fullName**>In vitro</**fullName**>

</**names**>

</**hostOrganism**>

</**hostOrganismList**>

<**interactionDetectionMethod**>

<**names**>

<**shortLabel**>protein kinase assay</**shortLabel**>

<**fullName**>protein kinase assay</**fullName**>

</**names**>

<**xref**>

<**primaryRef db="psi-mi" dbAc="MI:0488" id="MI:0424" refType="identity" refTypeAc="MI:0356"**/>

<**secondaryRef db="intact" dbAc="MI:0469" id="EBI-608517" refType="identity" refTypeAc="MI:0356"**/>

<**secondaryRef db="pubmed" dbAc="MI:0446" id="14755292" refType="primary-reference" refTypeAc="MI:0358"**/>

</**xref**>

</**interactionDetectionMethod**>

<**participantIdentificationMethod**>

<**names**>

<**shortLabel**>weight autoradiogra</**shortLabel**>

<**fullName**>molecular weight estimation by autoradiography</**fullName**>

</**names**>

<**xref**>

<**primaryRef db="psi-mi" dbAc="MI:0488" id="MI:0821" refType="identity" refTypeAc="MI:0356"**/>

<**secondaryRef db="intact" dbAc="MI:0469" id="EBI-1218638" refType="identity" refTypeAc="MI:0356"**/>

<**secondaryRef db="pubmed" dbAc="MI:0446" id="14755292" refType="primary-reference" refTypeAc="MI:0358"**/>

</**xref**>

</**participantIdentificationMethod**>

<**attributeList**>

<**attribute name="contact-email" nameAc="MI:0634"**>pcohen@bad.dundee.ac.uk</**attribute**>

<**attribute name="journal" nameAc="MI:0885"**>FEBS letters</**attribute**>

<**attribute name="publication year" nameAc="MI:0886"**>1999</**attribute**>

<**attribute name="author-list" nameAc="MI:0636"**>Thomas GM., Frame S., Goedert M., Nathke I., Polakis P., Cohen P.</**attribute**>

<**attribute name="curation depth" nameAc="MI:0955"**>imex curation</**attribute**>

<**attribute name="accepted"**>Accepted 2016-OCT-20 AT 10:35 BST AT 10:35 BST by MGT</**attribute**>

<**attribute name="comment" nameAc="MI:0612"**>

Assays involving GS-1 were not added as the peptide did not match GS-1 of any species. The phosphorylation of Tau by GSK was not added as the exact identity and origins of GSK are not known.

</**attribute**>

<**attribute name="full coverage" nameAc="MI:0957"**>Only protein-protein interactions</**attribute**>

<**attribute name="imex curation" nameAc="MI:0959"**>imex curation</**attribute**>

<**attribute name="correction comment"**/>

</**attributeList**>

</**experimentDescription**>

</**experimentList**>

<**interactorList**>

<**interactor id="2"**>

<**names**>

<**shortLabel**>gys1_human</**shortLabel**>

<**fullName**>Glycogen [starch] synthase, muscle</**fullName**>

<**alias type="gene name" typeAc="MI:0301"**>GYS1</**alias**>

<**alias type="gene name synonym" typeAc="MI:0302"**>GYS</**alias**>

</**names**>

<**xref**>

<**primaryRef db="uniprotkb" dbAc="MI:0486" id="P13807" version="SP_123" refType="identity" refTypeAc="MI:0356"**/>

<**secondaryRef db="intact" dbAc="MI:0469" id="EBI-963094" refType="intact-secondary"**/>

<**secondaryRef db="intact" dbAc="MI:0469" id="EBI-2948518" refType="intact-secondary"**/>

<**secondaryRef db="uniprotkb" dbAc="MI:0486" id="Q9BTT9" version="SP_123" refType="secondary-ac" refTypeAc="MI:0360"**/>

<**secondaryRef db="intact" dbAc="MI:0469" id="EBI-740553" refType="identity" refTypeAc="MI:0356"**/>

<**secondaryRef db="ensembl" dbAc="MI:0476" id="ENST00000263276"**/>

<**secondaryRef db="ensembl" dbAc="MI:0476" id="ENST00000323798"**/>

<**secondaryRef db="go" dbAc="MI:0448" id="GO:0004373"**/>

<**secondaryRef db="go" dbAc="MI:0448" id="GO:0005978"**/>

<**secondaryRef db="refseq" dbAc="MI:0481" id="NP_001155059.1"**/>

<**secondaryRef db="interpro" dbAc="MI:0449" id="IPR008631"**/>

<**secondaryRef db="go" dbAc="MI:0448" id="GO:0005829"**/>

<**secondaryRef db="refseq" dbAc="MI:0481" id="NP_002094.2"**/>

<**secondaryRef db="go" dbAc="MI:0448" id="GO:0005536"**/>

<**secondaryRef db="go" dbAc="MI:0448" id="GO:0007507"**/>

<**secondaryRef db="go" dbAc="MI:0448" id="GO:0016234"**/>

<**secondaryRef db="go" dbAc="MI:0448" id="GO:0019901"**/>

<**secondaryRef db="go" dbAc="MI:0448" id="GO:0061547"**/>

<**secondaryRef db="ensembl" dbAc="MI:0476" id="ENSG00000104812"**/>

<**secondaryRef db="ensembl" dbAc="MI:0476" id="ENSP00000263276"**/>

<**secondaryRef db="ensembl" dbAc="MI:0476" id="ENSP00000317904"**/>

<**secondaryRef db="go" dbAc="MI:0448" id="GO:0016020"**/>

<**secondaryRef db="reactome" dbAc="MI:0467" id="R-HSA-3322077"**/>

<**secondaryRef db="reactome" dbAc="MI:0467" id="R-HSA-3785653"**/>

<**secondaryRef db="reactome" dbAc="MI:0467" id="R-HSA-3814836"**/>

<**secondaryRef db="reactome" dbAc="MI:0467" id="R-HSA-3828062"**/>

<**secondaryRef db="reactome" dbAc="MI:0467" id="R-HSA-3878781"**/>

</**xref**>

<**interactorType**>

<**names**>

<**shortLabel**>protein</**shortLabel**>

<**fullName**>protein</**fullName**>

</**names**>

<**xref**>

<**primaryRef db="psi-mi" dbAc="MI:0488" id="MI:0326" refType="identity" refTypeAc="MI:0356"**/>

<**secondaryRef db="intact" dbAc="MI:0469" id="EBI-619654" refType="identity" refTypeAc="MI:0356"**/>

<**secondaryRef db="pubmed" dbAc="MI:0446" id="14755292" refType="primary-reference" refTypeAc="MI:0358"**/>

<**secondaryRef db="so" dbAc="MI:0601" id="SO:0000358" refType="see-also" refTypeAc="MI:0361"**/>

</**xref**>

</**interactorType**>

<**organism ncbiTaxId="9606"**>

<**names**>

<**shortLabel**>human</**shortLabel**>

<**fullName**>Homo sapiens</**fullName**>

<**alias type="synonym" typeAc="MI:1041"**>Human</**alias**>

</**names**>

</**organism**>

<**sequence**>MPLNRTLSMSSLPGLEDWEDEFDLENAVLFEVAWEVANKVGGIYTVLQTKAKVTGDEWGDNYFLVGPYTEQGVRTQVELLEAPTPALKRTLDSMNSKGCKVYFGRWLIEGGPLVVLLDVGASAWALERWKGELWDTCNIGVPWYDREANDAVLFGFLTTWFLGEFLAQSEEKPHVVAHFHEWLAGVGLCLCRARRLPVATIFTTHATLLGRYLCAGAVDFYNNLENFNVDKEAGERQIYHRYCMERAAAHCAHVFTTVSQITAIEAQHLLKRKPDIVTPNGLNVKKFSAMHEFQNLHAQSKARIQEFVRGHFYGHLDFNLDKTLYFFIAGRYEFSNKGADVFLEALARLNYLLRVNGSEQTVVAFFIMPARTNNFNVETLKGQAVRKQLWDTANTVKEKFGRKLYESLLVGSLPDMNKMLDKEDFTMMKRAIFATQRQSFPPVCTHNMLDDSSDPILTTIRRIGLFNSSADRVKVIFHPEFLSSTSPLLPVDYEEFVRGCHLGVFPSYYEPWGYTPAECTVMGIPSISTNLSGFGCFMEEHIADPSAYGIYILDRRFRSLDDSCSQLTSFLYSFCQQSRRQRIIQRNRTERLSDLLDWKYLGRYYMSARHMALSKAFPEHFTYEPNEADAAQGYRYPRPASVPPSPSLSRHSSPHQSEDEEDPRNGPLEEDGERYDEDEEAAKDRRNIRAPEWPRRASCTSSTSGSKRNSVDTATSSSLSTPSEPLSPTSSLGEERN</**sequence**>

<**attributeList**>

<**attribute name="crc64"**>0E321BBFDEB0BD7F</**attribute**>

</**attributeList**>

</**interactor**>

<**interactor id="3"**>

<**names**>

<**shortLabel**>gsk3b_human</**shortLabel**>

<**fullName**>Glycogen synthase kinase-3 beta</**fullName**>

<**alias type="gene name synonym" typeAc="MI:0302"**>Serine/threonine-protein kinase GSK3B</**alias**>

<**alias type="gene name" typeAc="MI:0301"**>GSK3B</**alias**>

</**names**>

<**xref**>

<**primaryRef db="uniprotkb" dbAc="MI:0486" id="P49841" version="SP_99" refType="identity" refTypeAc="MI:0356"**/>

<**secondaryRef db="uniprotkb" dbAc="MI:0486" id="D3DN89" version="SP_142" refType="secondary-ac" refTypeAc="MI:0360"**/>

<**secondaryRef db="uniprotkb" dbAc="MI:0486" id="Q9BWH3" version="SP_99" refType="secondary-ac" refTypeAc="MI:0360"**/>

<**secondaryRef db="uniprotkb" dbAc="MI:0486" id="Q9UL47" version="SP_99" refType="secondary-ac" refTypeAc="MI:0360"**/>

<**secondaryRef db="intact" dbAc="MI:0469" id="EBI-373586" refType="identity" refTypeAc="MI:0356"**/>

<**secondaryRef db="rcsb pdb" dbAc="MI:0460" id="4NM7"**/>

<**secondaryRef db="go" dbAc="MI:0448" id="GO:0007623"**/>

<**secondaryRef db="go" dbAc="MI:0448" id="GO:0002039"**/>

<**secondaryRef db="rcsb pdb" dbAc="MI:0460" id="4ACC"**/>

<**secondaryRef db="rcsb pdb" dbAc="MI:0460" id="4ACD"**/>

<**secondaryRef db="rcsb pdb" dbAc="MI:0460" id="4ACG"**/>

<**secondaryRef db="rcsb pdb" dbAc="MI:0460" id="4ACH"**/>

<**secondaryRef db="rcsb pdb" dbAc="MI:0460" id="4EKK"**/>

<**secondaryRef db="rcsb pdb" dbAc="MI:0460" id="4J1R"**/>

<**secondaryRef db="rcsb pdb" dbAc="MI:0460" id="4J71"**/>

<**secondaryRef db="go" dbAc="MI:0448" id="GO:0010614"**/>

<**secondaryRef db="rcsb pdb" dbAc="MI:0460" id="3MV5"**/>

<**secondaryRef db="rcsb pdb" dbAc="MI:0460" id="4NM5"**/>

<**secondaryRef db="refseq" dbAc="MI:0481" id="NP_001139628.1"**/>

<**secondaryRef db="refseq" dbAc="MI:0481" id="NP_002084.2"**/>

<**secondaryRef db="interpro" dbAc="MI:0449" id="IPR011009"**/>

<**secondaryRef db="interpro" dbAc="MI:0449" id="IPR000719"**/>

<**secondaryRef db="interpro" dbAc="MI:0449" id="IPR017441"**/>

<**secondaryRef db="interpro" dbAc="MI:0449" id="IPR008271"**/>

<**secondaryRef db="go" dbAc="MI:0448" id="GO:0030877"**/>

<**secondaryRef db="go" dbAc="MI:0448" id="GO:0005813"**/>

<**secondaryRef db="go" dbAc="MI:0448" id="GO:0005829"**/>

<**secondaryRef db="go" dbAc="MI:0448" id="GO:0005634"**/>

<**secondaryRef db="go" dbAc="MI:0448" id="GO:0005886"**/>

<**secondaryRef db="go" dbAc="MI:0448" id="GO:0005524"**/>

<**secondaryRef db="go" dbAc="MI:0448" id="GO:0004674"**/>

<**secondaryRef db="go" dbAc="MI:0448" id="GO:0050321"**/>

<**secondaryRef db="go" dbAc="MI:0448" id="GO:0060070"**/>

<**secondaryRef db="go" dbAc="MI:0448" id="GO:0001837"**/>

<**secondaryRef db="go" dbAc="MI:0448" id="GO:0006983"**/>

<**secondaryRef db="go" dbAc="MI:0448" id="GO:0005977"**/>

<**secondaryRef db="go" dbAc="MI:0448" id="GO:0021766"**/>

<**secondaryRef db="go" dbAc="MI:0448" id="GO:0043066"**/>

<**secondaryRef db="go" dbAc="MI:0448" id="GO:0032091"**/>

<**secondaryRef db="go" dbAc="MI:0448" id="GO:0031333"**/>

<**secondaryRef db="go" dbAc="MI:0448" id="GO:0018105"**/>

<**secondaryRef db="go" dbAc="MI:0448" id="GO:0001954"**/>

<**secondaryRef db="go" dbAc="MI:0448" id="GO:0045732"**/>

<**secondaryRef db="go" dbAc="MI:0448" id="GO:0031334"**/>

<**secondaryRef db="go" dbAc="MI:0448" id="GO:0046827"**/>

<**secondaryRef db="go" dbAc="MI:0448" id="GO:0032886"**/>

<**secondaryRef db="go" dbAc="MI:0448" id="GO:0071109"**/>

<**secondaryRef db="rcsb pdb" dbAc="MI:0460" id="1GNG"**/>

<**secondaryRef db="rcsb pdb" dbAc="MI:0460" id="1H8F"**/>

<**secondaryRef db="rcsb pdb" dbAc="MI:0460" id="1I09"**/>

<**secondaryRef db="rcsb pdb" dbAc="MI:0460" id="1J1B"**/>

<**secondaryRef db="rcsb pdb" dbAc="MI:0460" id="1J1C"**/>

<**secondaryRef db="rcsb pdb" dbAc="MI:0460" id="1O9U"**/>

<**secondaryRef db="rcsb pdb" dbAc="MI:0460" id="1PYX"**/>

<**secondaryRef db="rcsb pdb" dbAc="MI:0460" id="1Q3D"**/>

<**secondaryRef db="rcsb pdb" dbAc="MI:0460" id="1Q3W"**/>

<**secondaryRef db="rcsb pdb" dbAc="MI:0460" id="1Q41"**/>

<**secondaryRef db="rcsb pdb" dbAc="MI:0460" id="1Q4L"**/>

<**secondaryRef db="rcsb pdb" dbAc="MI:0460" id="1Q5K"**/>

<**secondaryRef db="rcsb pdb" dbAc="MI:0460" id="1R0E"**/>

<**secondaryRef db="rcsb pdb" dbAc="MI:0460" id="1UV5"**/>

<**secondaryRef db="rcsb pdb" dbAc="MI:0460" id="2JDO"**/>

<**secondaryRef db="rcsb pdb" dbAc="MI:0460" id="2JDR"**/>

<**secondaryRef db="rcsb pdb" dbAc="MI:0460" id="2JLD"**/>

<**secondaryRef db="rcsb pdb" dbAc="MI:0460" id="2O5K"**/>

<**secondaryRef db="rcsb pdb" dbAc="MI:0460" id="2OW3"**/>

<**secondaryRef db="rcsb pdb" dbAc="MI:0460" id="2UW9"**/>

<**secondaryRef db="rcsb pdb" dbAc="MI:0460" id="2X39"**/>

<**secondaryRef db="rcsb pdb" dbAc="MI:0460" id="2XH5"**/>

<**secondaryRef db="rcsb pdb" dbAc="MI:0460" id="3CQU"**/>

<**secondaryRef db="rcsb pdb" dbAc="MI:0460" id="3CQW"**/>

<**secondaryRef db="rcsb pdb" dbAc="MI:0460" id="3DU8"**/>

<**secondaryRef db="rcsb pdb" dbAc="MI:0460" id="3E87"**/>

<**secondaryRef db="rcsb pdb" dbAc="MI:0460" id="3E88"**/>

<**secondaryRef db="rcsb pdb" dbAc="MI:0460" id="3E8D"**/>

<**secondaryRef db="rcsb pdb" dbAc="MI:0460" id="3F7Z"**/>

<**secondaryRef db="rcsb pdb" dbAc="MI:0460" id="3F88"**/>

<**secondaryRef db="rcsb pdb" dbAc="MI:0460" id="3GB2"**/>

<**secondaryRef db="rcsb pdb" dbAc="MI:0460" id="3I4B"**/>

<**secondaryRef db="rcsb pdb" dbAc="MI:0460" id="3L1S"**/>

<**secondaryRef db="rcsb pdb" dbAc="MI:0460" id="3M1S"**/>

<**secondaryRef db="rcsb pdb" dbAc="MI:0460" id="3PUP"**/>

<**secondaryRef db="rcsb pdb" dbAc="MI:0460" id="3Q3B"**/>

<**secondaryRef db="rcsb pdb" dbAc="MI:0460" id="3SD0"**/>

<**secondaryRef db="rcsb pdb" dbAc="MI:0460" id="3ZRK"**/>

<**secondaryRef db="rcsb pdb" dbAc="MI:0460" id="3ZRL"**/>

<**secondaryRef db="rcsb pdb" dbAc="MI:0460" id="3ZRM"**/>

<**secondaryRef db="go" dbAc="MI:0448" id="GO:0007409"**/>

<**secondaryRef db="go" dbAc="MI:0448" id="GO:0016055"**/>

<**secondaryRef db="go" dbAc="MI:0448" id="GO:0043161"**/>

<**secondaryRef db="go" dbAc="MI:0448" id="GO:1904885"**/>

<**secondaryRef db="go" dbAc="MI:0448" id="GO:1904886"**/>

<**secondaryRef db="interpro" dbAc="MI:0449" id="IPR033573"**/>

<**secondaryRef db="rcsb pdb" dbAc="MI:0460" id="5HLN"**/>

<**secondaryRef db="rcsb pdb" dbAc="MI:0460" id="5HLP"**/>

<**secondaryRef db="go" dbAc="MI:0448" id="GO:0090090"**/>

<**secondaryRef db="go" dbAc="MI:0448" id="GO:2000466"**/>

<**secondaryRef db="go" dbAc="MI:0448" id="GO:0045719"**/>

<**secondaryRef db="go" dbAc="MI:0448" id="GO:0051534"**/>

<**secondaryRef db="go" dbAc="MI:0448" id="GO:2000077"**/>

<**secondaryRef db="rcsb pdb" dbAc="MI:0460" id="4AFJ"**/>

<**secondaryRef db="rcsb pdb" dbAc="MI:0460" id="4DIT"**/>

<**secondaryRef db="rcsb pdb" dbAc="MI:0460" id="4IQ6"**/>

<**secondaryRef db="rcsb pdb" dbAc="MI:0460" id="3SAY"**/>

<**secondaryRef db="go" dbAc="MI:0448" id="GO:0000320"**/>

<**secondaryRef db="go" dbAc="MI:0448" id="GO:0006349"**/>

<**secondaryRef db="go" dbAc="MI:0448" id="GO:0006611"**/>

<**secondaryRef db="go" dbAc="MI:0448" id="GO:0007520"**/>

<**secondaryRef db="go" dbAc="MI:0448" id="GO:0009887"**/>

<**secondaryRef db="go" dbAc="MI:0448" id="GO:0010800"**/>

<**secondaryRef db="go" dbAc="MI:0448" id="GO:0016477"**/>

<**secondaryRef db="go" dbAc="MI:0448" id="GO:0030426"**/>

<**secondaryRef db="go" dbAc="MI:0448" id="GO:0030529"**/>

<**secondaryRef db="go" dbAc="MI:0448" id="GO:0032092"**/>

<**secondaryRef db="go" dbAc="MI:0448" id="GO:0033138"**/>

<**secondaryRef db="go" dbAc="MI:0448" id="GO:0035372"**/>

<**secondaryRef db="go" dbAc="MI:0448" id="GO:0043025"**/>

<**secondaryRef db="go" dbAc="MI:0448" id="GO:0043198"**/>

<**secondaryRef db="go" dbAc="MI:0448" id="GO:0044027"**/>

<**secondaryRef db="go" dbAc="MI:0448" id="GO:0044337"**/>

<**secondaryRef db="go" dbAc="MI:0448" id="GO:0045444"**/>

<**secondaryRef db="go" dbAc="MI:0448" id="GO:0045944"**/>

<**secondaryRef db="go" dbAc="MI:0448" id="GO:0048471"**/>

<**secondaryRef db="go" dbAc="MI:0448" id="GO:2000738"**/>

<**secondaryRef db="rcsb pdb" dbAc="MI:0460" id="4B7T"**/>

<**secondaryRef db="rcsb pdb" dbAc="MI:0460" id="3ZDI"**/>

<**secondaryRef db="go" dbAc="MI:0448" id="GO:0036016"**/>

<**secondaryRef db="go" dbAc="MI:0448" id="GO:0097192"**/>

<**secondaryRef db="go" dbAc="MI:0448" id="GO:1901030"**/>

<**secondaryRef db="go" dbAc="MI:0448" id="GO:0007212"**/>

<**secondaryRef db="go" dbAc="MI:0448" id="GO:0099565"**/>

<**secondaryRef db="go" dbAc="MI:0448" id="GO:1901216"**/>

<**secondaryRef db="rcsb pdb" dbAc="MI:0460" id="1O6K"**/>

<**secondaryRef db="rcsb pdb" dbAc="MI:0460" id="1O6L"**/>

<**secondaryRef db="go" dbAc="MI:0448" id="GO:0001085"**/>

<**secondaryRef db="go" dbAc="MI:0448" id="GO:0005737"**/>

<**secondaryRef db="go" dbAc="MI:0448" id="GO:0006468"**/>

<**secondaryRef db="go" dbAc="MI:0448" id="GO:0008013"**/>

<**secondaryRef db="go" dbAc="MI:0448" id="GO:0016301"**/>

<**secondaryRef db="go" dbAc="MI:0448" id="GO:0019901"**/>

<**secondaryRef db="go" dbAc="MI:0448" id="GO:0031625"**/>

<**secondaryRef db="go" dbAc="MI:0448" id="GO:0034236"**/>

<**secondaryRef db="go" dbAc="MI:0448" id="GO:0035556"**/>

<**secondaryRef db="go" dbAc="MI:0448" id="GO:0051059"**/>

<**secondaryRef db="rcsb pdb" dbAc="MI:0460" id="3QKK"**/>

<**secondaryRef db="rcsb pdb" dbAc="MI:0460" id="4NM0"**/>

<**secondaryRef db="rcsb pdb" dbAc="MI:0460" id="4NM3"**/>

<**secondaryRef db="rcsb pdb" dbAc="MI:0460" id="3OW4"**/>

<**secondaryRef db="ensembl" dbAc="MI:0476" id="ENST00000264235"**/>

<**secondaryRef db="ensembl" dbAc="MI:0476" id="ENST00000316626"**/>

<**secondaryRef db="ensembl" dbAc="MI:0476" id="ENSG00000082701"**/>

<**secondaryRef db="ensembl" dbAc="MI:0476" id="ENSP00000264235"**/>

<**secondaryRef db="ensembl" dbAc="MI:0476" id="ENSP00000324806"**/>

<**secondaryRef db="go" dbAc="MI:0448" id="GO:1900181"**/>

<**secondaryRef db="go" dbAc="MI:0448" id="GO:0043547"**/>

<**secondaryRef db="rcsb pdb" dbAc="MI:0460" id="4PTC"**/>

<**secondaryRef db="rcsb pdb" dbAc="MI:0460" id="4PTE"**/>

<**secondaryRef db="rcsb pdb" dbAc="MI:0460" id="4PTG"**/>

<**secondaryRef db="go" dbAc="MI:0448" id="GO:0014043"**/>

<**secondaryRef db="go" dbAc="MI:0448" id="GO:0032436"**/>

<**secondaryRef db="reactome" dbAc="MI:0467" id="R-HSA-195253"**/>

<**secondaryRef db="go" dbAc="MI:0448" id="GO:1904339"**/>

<**secondaryRef db="reactome" dbAc="MI:0467" id="R-HSA-5250924"**/>

<**secondaryRef db="go" dbAc="MI:0448" id="GO:0005739"**/>

<**secondaryRef db="go" dbAc="MI:0448" id="GO:0046777"**/>

<**secondaryRef db="reactome" dbAc="MI:0467" id="R-HSA-196299"**/>

<**secondaryRef db="reactome" dbAc="MI:0467" id="R-HSA-198323"**/>

<**secondaryRef db="reactome" dbAc="MI:0467" id="R-HSA-3371453"**/>

<**secondaryRef db="reactome" dbAc="MI:0467" id="R-HSA-399956"**/>

<**secondaryRef db="reactome" dbAc="MI:0467" id="R-HSA-4641262"**/>

<**secondaryRef db="reactome" dbAc="MI:0467" id="R-HSA-5339716"**/>

<**secondaryRef db="reactome" dbAc="MI:0467" id="R-HSA-5358747"**/>

<**secondaryRef db="reactome" dbAc="MI:0467" id="R-HSA-5358749"**/>

<**secondaryRef db="reactome" dbAc="MI:0467" id="R-HSA-5358751"**/>

<**secondaryRef db="reactome" dbAc="MI:0467" id="R-HSA-5358752"**/>

<**secondaryRef db="reactome" dbAc="MI:0467" id="R-HSA-5467337"**/>

<**secondaryRef db="reactome" dbAc="MI:0467" id="R-HSA-5467340"**/>

<**secondaryRef db="reactome" dbAc="MI:0467" id="R-HSA-5467348"**/>

<**secondaryRef db="reactome" dbAc="MI:0467" id="R-HSA-5610783"**/>

<**secondaryRef db="reactome" dbAc="MI:0467" id="R-HSA-5610785"**/>

<**secondaryRef db="reactome" dbAc="MI:0467" id="R-HSA-5674400"**/>

<**secondaryRef db="go" dbAc="MI:0448" id="GO:0045773"**/>

<**secondaryRef db="go" dbAc="MI:0448" id="GO:1900034"**/>

<**secondaryRef db="go" dbAc="MI:0448" id="GO:0035729"**/>

<**secondaryRef db="go" dbAc="MI:0448" id="GO:0070059"**/>

<**secondaryRef db="go" dbAc="MI:0448" id="GO:0004672"**/>

<**secondaryRef db="go" dbAc="MI:0448" id="GO:0018107"**/>

<**secondaryRef db="go" dbAc="MI:0448" id="GO:1990909"**/>

<**secondaryRef db="rcsb pdb" dbAc="MI:0460" id="5F94"**/>

<**secondaryRef db="rcsb pdb" dbAc="MI:0460" id="5F95"**/>

<**secondaryRef db="go" dbAc="MI:0448" id="GO:0010508"**/>

<**secondaryRef db="go" dbAc="MI:0448" id="GO:0032007"**/>

<**secondaryRef db="go" dbAc="MI:0448" id="GO:0010977"**/>

<**secondaryRef db="go" dbAc="MI:0448" id="GO:0014069"**/>

<**secondaryRef db="go" dbAc="MI:0448" id="GO:2000727"**/>

<**secondaryRef db="rcsb pdb" dbAc="MI:0460" id="5K5N"**/>

<**secondaryRef db="go" dbAc="MI:0448" id="GO:0002020"**/>

<**secondaryRef db="go" dbAc="MI:0448" id="GO:0010822"**/>

</**xref**>

<**interactorType**>

<**names**>

<**shortLabel**>protein</**shortLabel**>

<**fullName**>protein</**fullName**>

</**names**>

<**xref**>

<**primaryRef db="psi-mi" dbAc="MI:0488" id="MI:0326" refType="identity" refTypeAc="MI:0356"**/>

<**secondaryRef db="intact" dbAc="MI:0469" id="EBI-619654" refType="identity" refTypeAc="MI:0356"**/>

<**secondaryRef db="pubmed" dbAc="MI:0446" id="14755292" refType="primary-reference" refTypeAc="MI:0358"**/>

<**secondaryRef db="so" dbAc="MI:0601" id="SO:0000358" refType="see-also" refTypeAc="MI:0361"**/>

</**xref**>

</**interactorType**>

<**organism ncbiTaxId="9606"**>

<**names**>

<**shortLabel**>human</**shortLabel**>

<**fullName**>Homo sapiens</**fullName**>

<**alias type="synonym" typeAc="MI:1041"**>Human</**alias**>

</**names**>

</**organism**>

<**sequence**>

MSGRPRTTSFAESCKPVQQPSAFGSMKVSRDKDGSKVTTVVATPGQGPDRPQEVSYTDTKVIGNGSFGVVYQAKLCDSGELVAIKKVLQDKRFKNRELQIMRKLDHCNIVRLRYFFYSSGEKKDEVYLNLVLDYVPETVYRVARHYSRAKQTLPVIYVKLYMYQLFRSLAYIHSFGICHRDIKPQNLLLDPDTAVLKLCDFGSAKQLVRGEPNVSYICSRYYRAPELIFGATDYTSSIDVWSAGCVLAELLLGQPIFPGDSGVDQLVEIIKVLGTPTREQIREMNPNYTEFKFPQIKAHPWTKVFRPRTPPEAIALCSRLLEYTPTARLTPLEACAHSFFDELRDPNVKLPNGRDTPALFNFTTQELSSNPPLATILIPPHARIQAAASTPTNATAASDANTGDRGQTNNAASASASNST

</**sequence**>

<**attributeList**>

<**attribute name="crc64"**>4ACC24D00CDBB9C3</**attribute**>

</**attributeList**>

</**interactor**>

</**interactorList**>

<**interactionList**>

<**interaction id="4" imexId="IM-25329-17"**>

<**names**>

<**shortLabel**>gsk3b-gys1-1</**shortLabel**>

</**names**>

<**xref**>

<**primaryRef db="intact" dbAc="MI:0469" id="EBI-12553583" refType="identity" refTypeAc="MI:0356"**/>

<**secondaryRef db="go" dbAc="MI:0448" id="GO:0006468" refType="process" refTypeAc="MI:0359"**/>

<**secondaryRef db="go" dbAc="MI:0448" id="GO:0004672" refType="function" refTypeAc="MI:0355"**/>

<**secondaryRef db="imex" dbAc="MI:0670" id="IM-25329-17" refType="imex-primary" refTypeAc="MI:0662"**/>

</**xref**>

<**experimentList**>

<**experimentRef**>1</**experimentRef**>

</**experimentList**>

<**participantList**>

<**participant id="5"**>

<**interactorRef**>3</**interactorRef**>

<**biologicalRole**>

<**names**>

<**shortLabel**>enzyme</**shortLabel**>

<**fullName**>enzyme</**fullName**>

</**names**>

<**xref**>

<**primaryRef db="psi-mi" dbAc="MI:0488" id="MI:0501" refType="identity" refTypeAc="MI:0356"**/>

<**secondaryRef db="intact" dbAc="MI:0469" id="EBI-46" refType="identity" refTypeAc="MI:0356"**/>

<**secondaryRef db="pubmed" dbAc="MI:0446" id="14755292" refType="primary-reference" refTypeAc="MI:0358"**/>

</**xref**>

</**biologicalRole**>

<**experimentalRoleList**>

<**experimentalRole**>

<**names**>

<**shortLabel**>neutral component</**shortLabel**>

<**fullName**>neutral component</**fullName**>

</**names**>

<**xref**>

<**primaryRef db="psi-mi" dbAc="MI:0488" id="MI:0497" refType="identity" refTypeAc="MI:0356"**/>

<**secondaryRef db="intact" dbAc="MI:0469" id="EBI-55" refType="identity" refTypeAc="MI:0356"**/>

<**secondaryRef db="pubmed" dbAc="MI:0446" id="14755292" refType="primary-reference" refTypeAc="MI:0358"**/>

</**xref**>

</**experimentalRole**>

</**experimentalRoleList**>

<**featureList**>

<**feature id="6"**>

<**names**>

<**shortLabel**>region</**shortLabel**>

</**names**>

<**xref**>

<**primaryRef db="intact" dbAc="MI:0469" id="EBI-12553589" refType="identity" refTypeAc="MI:0356"**/>

</**xref**>

<**featureType**>

<**names**>

<**shortLabel**>his tag</**shortLabel**>

<**fullName**>his tag</**fullName**>

<**alias type="go synonym" typeAc="MI:0303"**>Hexa-His-tag</**alias**>

<**alias type="go synonym" typeAc="MI:0303"**>6-His-tag</**alias**>

<**alias type="go synonym" typeAc="MI:0303"**>Histidine-tag</**alias**>

</**names**>

<**xref**>

<**primaryRef db="psi-mi" dbAc="MI:0488" id="MI:0521" refType="identity" refTypeAc="MI:0356"**/>

<**secondaryRef db="intact" dbAc="MI:0469" id="EBI-456516" refType="identity" refTypeAc="MI:0356"**/>

<**secondaryRef db="pubmed" dbAc="MI:0446" id="14755292" refType="primary-reference" refTypeAc="MI:0358"**/>

</**xref**>

</**featureType**>

<**featureRangeList**>

<**featureRange**>

<**startStatus**>

<**names**>

<**shortLabel**>undetermined</**shortLabel**>

<**fullName**>undetermined sequence position</**fullName**>

</**names**>

<**xref**>

<**primaryRef db="psi-mi" dbAc="MI:0488" id="MI:0339" refType="identity" refTypeAc="MI:0356"**/>

<**secondaryRef db="intact" dbAc="MI:0469" id="EBI-448295" refType="identity" refTypeAc="MI:0356"**/>

<**secondaryRef db="pubmed" dbAc="MI:0446" id="14755292" refType="primary-reference" refTypeAc="MI:0358"**/>

</**xref**>

</**startStatus**>

<**endStatus**>

<**names**>

<**shortLabel**>undetermined</**shortLabel**>

<**fullName**>undetermined sequence position</**fullName**>

</**names**>

<**xref**>

<**primaryRef db="psi-mi" dbAc="MI:0488" id="MI:0339" refType="identity" refTypeAc="MI:0356"**/>

<**secondaryRef db="intact" dbAc="MI:0469" id="EBI-448295" refType="identity" refTypeAc="MI:0356"**/>

<**secondaryRef db="pubmed" dbAc="MI:0446" id="14755292" refType="primary-reference" refTypeAc="MI:0358"**/>

</**xref**>

</**endStatus**>

</**featureRange**>

</**featureRangeList**>

</**feature**>

</**featureList**>

<**hostOrganismList**>

<**hostOrganism ncbiTaxId="7108"**>

<**names**>

<**shortLabel**>spofr-sf_9</**shortLabel**>

<**fullName**>Spodoptera frugiperda insect cells</**fullName**>

</**names**>

<**cellType**>

<**names**>

<**shortLabel**>sf_9</**shortLabel**>

<**fullName**>insect cells</**fullName**>

</**names**>

<**xref**>

<**primaryRef db="cabri" dbAc="MI:0246" id="ACC 125" refType="identity" refTypeAc="MI:0356"**/>

<**secondaryRef db="intact" dbAc="MI:0469" id="IA:0078" refType="identity" refTypeAc="MI:0356"**/>

<**secondaryRef db="mint" dbAc="MI:0471" id="MINT-86941" refType="identity" refTypeAc="MI:0356"**/>

<**secondaryRef db="intact" dbAc="MI:0469" id="EBI-346407" refType="identity" refTypeAc="MI:0356"**/>

</**xref**>

<**attributeList**>

<**attribute name="comment" nameAc="MI:0612"**>Derived from pupal ovarian tissue of the fall armyworm</**attribute**>

<**attribute name="comment" nameAc="MI:0612"**>Cloned from the parent cell line, IPLB-SF-21-AE (SF-21)</**attribute**>

<**attribute name="comment" nameAc="MI:0612"**>

derived from immature ovaries of fall armyworm Spodoptera frugiperda pupae</**attribute**>

<**attribute name="comment" nameAc="MI:0612"**>cells are susceptible to Baculovirus infection</**attribute**>

</**attributeList**>

</**cellType**>

</**hostOrganism**>

</**hostOrganismList**>

</**participant**>

<**participant id="7"**>

<**interactorRef**>2</**interactorRef**>

<**biologicalRole**>

<**names**>

<**shortLabel**>enzyme target</**shortLabel**>

<**fullName**>enzyme target</**fullName**>

<**alias type="go synonym" typeAc="MI:0303"**>substrate</**alias**>

</**names**>

<**xref**>

<**primaryRef db="psi-mi" dbAc="MI:0488" id="MI:0502" refType="identity" refTypeAc="MI:0356"**/>

<**secondaryRef db="intact" dbAc="MI:0469" id="EBI-64" refType="identity" refTypeAc="MI:0356"**/>

<**secondaryRef db="pubmed" dbAc="MI:0446" id="14755292" refType="primary-reference" refTypeAc="MI:0358"**/>

</**xref**>

</**biologicalRole**>

<**experimentalRoleList**>

<**experimentalRole**>

<**names**>

<**shortLabel**>neutral component</**shortLabel**>

<**fullName**>neutral component</**fullName**>

</**names**>

<**xref**>

<**primaryRef db="psi-mi" dbAc="MI:0488" id="MI:0497" refType="identity" refTypeAc="MI:0356"**/>

<**secondaryRef db="intact" dbAc="MI:0469" id="EBI-55" refType="identity" refTypeAc="MI:0356"**/>

<**secondaryRef db="pubmed" dbAc="MI:0446" id="14755292" refType="primary-reference" refTypeAc="MI:0358"**/>

</**xref**>

</**experimentalRole**>

</**experimentalRoleList**>

<**featureList**>

<**feature id="8"**>

<**names**>

<**shortLabel**>region</**shortLabel**>

</**names**>

<**xref**>

<**primaryRef db="intact" dbAc="MI:0469" id="EBI-12553592" refType="identity" refTypeAc="MI:0356"**/>

</**xref**>

<**featureType**>

<**names**>

<**shortLabel**>ophosres</**shortLabel**>

<**fullName**>N-phosphorylated residue</**fullName**>

</**names**>

<**xref**>

<**primaryRef db="psi-mod" dbAc="MI:0897" id="MOD:01456" refType="identity" refTypeAc="MI:0356"**/>

<**secondaryRef db="intact" dbAc="MI:0469" id="EBI-5527781" refType="identity" refTypeAc="MI:0356"**/>

<**secondaryRef db="pubmed" dbAc="MI:0446" id="18688235" refType="primary-reference" refTypeAc="MI:0358"**/>

</**xref**>

</**featureType**>

<**featureRangeList**>

<**featureRange**>

<**startStatus**>

<**names**>

<**shortLabel**>undetermined</**shortLabel**>

<**fullName**>undetermined sequence position</**fullName**>

</**names**>

<**xref**>

<**primaryRef db="psi-mi" dbAc="MI:0488" id="MI:0339" refType="identity" refTypeAc="MI:0356"**/>

<**secondaryRef db="intact" dbAc="MI:0469" id="EBI-448295" refType="identity" refTypeAc="MI:0356"**/>

<**secondaryRef db="pubmed" dbAc="MI:0446" id="14755292" refType="primary-reference" refTypeAc="MI:0358"**/>

</**xref**>

</**startStatus**>

<**endStatus**>

<**names**>

<**shortLabel**>undetermined</**shortLabel**>

<**fullName**>undetermined sequence position</**fullName**>

</**names**>

<**xref**>

<**primaryRef db="psi-mi" dbAc="MI:0488" id="MI:0339" refType="identity" refTypeAc="MI:0356"**/>

<**secondaryRef db="intact" dbAc="MI:0469" id="EBI-448295" refType="identity" refTypeAc="MI:0356"**/>

<**secondaryRef db="pubmed" dbAc="MI:0446" id="14755292" refType="primary-reference" refTypeAc="MI:0358"**/>

</**xref**>

</**endStatus**>

</**featureRange**>

</**featureRangeList**>

<**featureRole**>

<**names**>

<**shortLabel**>resulting-ptm</**shortLabel**>

<**fullName**>resulting-ptm</**fullName**>

</**names**>

<**xref**>

<**primaryRef db="psi-mi" dbAc="MI:0488" id="MI:0639" refType="identity" refTypeAc="MI:0356"**/>

<**secondaryRef db="intact" dbAc="MI:0469" id="EBI-877" refType="identity" refTypeAc="MI:0356"**/>

<**secondaryRef db="pubmed" dbAc="MI:0446" id="14755292" refType="primary-reference" refTypeAc="MI:0358"**/>

</**xref**>

</**featureRole**>

</**feature**>

<**feature id="9"**>

<**names**>

<**shortLabel**>region</**shortLabel**>

</**names**>

<**xref**>

<**primaryRef db="intact" dbAc="MI:0469" id="EBI-12553594" refType="identity" refTypeAc="MI:0356"**/>

</**xref**>

<**featureType**>

<**names**>

<**shortLabel**>sufficient to bind</**shortLabel**>

<**fullName**>sufficient binding region</**fullName**>

<**alias type="synonym" typeAc="MI:1041"**>sufficient to bind</**alias**>

</**names**>

<**xref**>

<**primaryRef db="psi-mi" dbAc="MI:0488" id="MI:0442" refType="identity" refTypeAc="MI:0356"**/>

<**secondaryRef db="intact" dbAc="MI:0469" id="EBI-608899" refType="identity" refTypeAc="MI:0356"**/>

<**secondaryRef db="pubmed" dbAc="MI:0446" id="14755292" refType="primary-reference" refTypeAc="MI:0358"**/>

</**xref**>

</**featureType**>

<**featureRangeList**>

<**featureRange**>

<**startStatus**>

<**names**>

<**shortLabel**>certain</**shortLabel**>

<**fullName**>certain sequence position</**fullName**>

<**alias type="synonym" typeAc="MI:1041"**>certain</**alias**>

</**names**>

<**xref**>

<**primaryRef db="psi-mi" dbAc="MI:0488" id="MI:0335" refType="identity" refTypeAc="MI:0356"**/>

<**secondaryRef db="intact" dbAc="MI:0469" id="EBI-540564" refType="identity" refTypeAc="MI:0356"**/>

<**secondaryRef db="pubmed" dbAc="MI:0446" id="14755292" refType="primary-reference" refTypeAc="MI:0358"**/>

</**xref**>

</**startStatus**>

<**begin position="637"**/>

<**endStatus**>

<**names**>

<**shortLabel**>certain</**shortLabel**>

<**fullName**>certain sequence position</**fullName**>

<**alias type="synonym" typeAc="MI:1041"**>certain</**alias**>

</**names**>

<**xref**>

<**primaryRef db="psi-mi" dbAc="MI:0488" id="MI:0335" refType="identity" refTypeAc="MI:0356"**/>

<**secondaryRef db="intact" dbAc="MI:0469" id="EBI-540564" refType="identity" refTypeAc="MI:0356"**/>

<**secondaryRef db="pubmed" dbAc="MI:0446" id="14755292" refType="primary-reference" refTypeAc="MI:0358"**/>

</**xref**>

</**endStatus**>

<**end position="661"**/>

</**featureRange**>

</**featureRangeList**>

</**feature**>

<**feature id="10"**>

<**names**>

<**shortLabel**>pro639arg</**shortLabel**>

</**names**>

<**xref**>

<**primaryRef db="intact" dbAc="MI:0469" id="EBI-12553596" refType="identity" refTypeAc="MI:0356"**/>

</**xref**>

<**featureType**>

<**names**>

<**shortLabel**>mutation</**shortLabel**>

<**fullName**>mutation</**fullName**>

</**names**>

<**xref**>

<**primaryRef db="psi-mi" dbAc="MI:0488" id="MI:0118" refType="identity" refTypeAc="MI:0356"**/>

<**secondaryRef db="intact" dbAc="MI:0469" id="EBI-456558" refType="identity" refTypeAc="MI:0356"**/>

<**secondaryRef db="pubmed" dbAc="MI:0446" id="14755292" refType="primary-reference" refTypeAc="MI:0358"**/>

</**xref**>

</**featureType**>

<**featureRangeList**>

<**featureRange**>

<**startStatus**>

<**names**>

<**shortLabel**>certain</**shortLabel**>

<**fullName**>certain sequence position</**fullName**>

<**alias type="synonym" typeAc="MI:1041"**>certain</**alias**>

</**names**>

<**xref**>

<**primaryRef db="psi-mi" dbAc="MI:0488" id="MI:0335" refType="identity" refTypeAc="MI:0356"**/>

<**secondaryRef db="intact" dbAc="MI:0469" id="EBI-540564" refType="identity" refTypeAc="MI:0356"**/>

<**secondaryRef db="pubmed" dbAc="MI:0446" id="14755292" refType="primary-reference" refTypeAc="MI:0358"**/>

</**xref**>

</**startStatus**>

<**begin position="639"**/>

<**endStatus**>

<**names**>

<**shortLabel**>certain</**shortLabel**>

<**fullName**>certain sequence position</**fullName**>

<**alias type="synonym" typeAc="MI:1041"**>certain</**alias**>

</**names**>

<**xref**>

<**primaryRef db="psi-mi" dbAc="MI:0488" id="MI:0335" refType="identity" refTypeAc="MI:0356"**/>

<**secondaryRef db="intact" dbAc="MI:0469" id="EBI-540564" refType="identity" refTypeAc="MI:0356"**/>

<**secondaryRef db="pubmed" dbAc="MI:0446" id="14755292" refType="primary-reference" refTypeAc="MI:0358"**/>

</**xref**>

</**endStatus**>

<**end position="639"**/>

<**resultingSequence**>

<**originalSequence**>P</**originalSequence**>

<**newSequence**>R</**newSequence**>

</**resultingSequence**>

</**featureRange**>

</**featureRangeList**>

</**feature**>

<**feature id="11"**>

<**names**>

<**shortLabel**>ser-657</**shortLabel**>

</**names**>

<**xref**>

<**primaryRef db="intact" dbAc="MI:0469" id="EBI-12553602" refType="identity" refTypeAc="MI:0356"**/>

</**xref**>

<**featureType**>

<**names**>

<**shortLabel**>opser</**shortLabel**>

<**fullName**>O-phospho-L-serine</**fullName**>

<**alias type="synonym" typeAc="MI:1041"**>(2S)-2-amino-3-(phosphonooxy)propanoic acid</**alias**>

<**alias type="synonym" typeAc="MI:1041"**>2-amino-3-hydroxypropanoic acid 3-phosphate</**alias**>

<**alias type="synonym" typeAc="MI:1041"**>2-azanyl-3-(phosphonooxy)propanoic acid</**alias**>

<**alias type="synonym" typeAc="MI:1041"**>ACT_SITE Phosphoserine intermediate</**alias**>

<**alias type="synonym" typeAc="MI:1041"**>MOD_RES Phosphoserine</**alias**>

<**alias type="synonym" typeAc="MI:1041"**>O-phospho-L-serine</**alias**>

<**alias type="synonym" typeAc="MI:1041"**>O-phosphonoserine</**alias**>

<**alias type="synonym" typeAc="MI:1041"**>O-phosphorylated L-serine</**alias**>

<**alias type="synonym" typeAc="MI:1041"**>O3-phosphoserine</**alias**>

<**alias type="synonym" typeAc="MI:1041"**>serine phosphate ester</**alias**>

</**names**>

<**xref**>

<**primaryRef db="psi-mod" dbAc="MI:0897" id="MOD:00046" refType="identity" refTypeAc="MI:0356"**/>

<**secondaryRef db="intact" dbAc="MI:0469" id="EBI-456676" refType="identity" refTypeAc="MI:0356"**/>

<**secondaryRef db="pubmed" dbAc="MI:0446" id="12923550" refType="primary-reference" refTypeAc="MI:0358"**/>

<**secondaryRef db="pubmed" dbAc="MI:0446" id="4065410" refType="primary-reference" refTypeAc="MI:0358"**/>

<**secondaryRef db="pubmed" dbAc="MI:0446" id="8061611" refType="primary-reference" refTypeAc="MI:0358"**/>

<**secondaryRef db="resid" dbAc="MI:0248" id="AA0037" refType="see-also" refTypeAc="MI:0361"**/>

<**secondaryRef db="chebi" dbAc="MI:0474" id="15811" refType="see-also" refTypeAc="MI:0361"**/>

</**xref**>

</**featureType**>

<**featureRangeList**>

<**featureRange**>

<**startStatus**>

<**names**>

<**shortLabel**>certain</**shortLabel**>

<**fullName**>certain sequence position</**fullName**>

<**alias type="synonym" typeAc="MI:1041"**>certain</**alias**>

</**names**>

<**xref**>

<**primaryRef db="psi-mi" dbAc="MI:0488" id="MI:0335" refType="identity" refTypeAc="MI:0356"**/>

<**secondaryRef db="intact" dbAc="MI:0469" id="EBI-540564" refType="identity" refTypeAc="MI:0356"**/>

<**secondaryRef db="pubmed" dbAc="MI:0446" id="14755292" refType="primary-reference" refTypeAc="MI:0358"**/>

</**xref**>

</**startStatus**>

<**begin position="657"**/>

<**endStatus**>

<**names**>

<**shortLabel**>certain</**shortLabel**>

<**fullName**>certain sequence position</**fullName**>

<**alias type="synonym" typeAc="MI:1041"**>certain</**alias**>

</**names**>

<**xref**>

<**primaryRef db="psi-mi" dbAc="MI:0488" id="MI:0335" refType="identity" refTypeAc="MI:0356"**/>

<**secondaryRef db="intact" dbAc="MI:0469" id="EBI-540564" refType="identity" refTypeAc="MI:0356"**/>

<**secondaryRef db="pubmed" dbAc="MI:0446" id="14755292" refType="primary-reference" refTypeAc="MI:0358"**/>

</**xref**>

</**endStatus**>

<**end position="657"**/>

</**featureRange**>

</**featureRangeList**>

</**feature**>

<**feature id="12"**>

<**names**>

<**shortLabel**>pro637tyr</**shortLabel**>

</**names**>

<**xref**>

<**primaryRef db="intact" dbAc="MI:0469" id="EBI-12553604" refType="identity" refTypeAc="MI:0356"**/>

</**xref**>

<**featureType**>

<**names**>

<**shortLabel**>mutation</**shortLabel**>

<**fullName**>mutation</**fullName**>

</**names**>

<**xref**>

<**primaryRef db="psi-mi" dbAc="MI:0488" id="MI:0118" refType="identity" refTypeAc="MI:0356"**/>

<**secondaryRef db="intact" dbAc="MI:0469" id="EBI-456558" refType="identity" refTypeAc="MI:0356"**/>

<**secondaryRef db="pubmed" dbAc="MI:0446" id="14755292" refType="primary-reference" refTypeAc="MI:0358"**/>

</**xref**>

</**featureType**>

<**featureRangeList**>

<**featureRange**>

<**startStatus**>

<**names**>

<**shortLabel**>certain</**shortLabel**>

<**fullName**>certain sequence position</**fullName**>

<**alias type="synonym" typeAc="MI:1041"**>certain</**alias**>

</**names**>

<**xref**>

<**primaryRef db="psi-mi" dbAc="MI:0488" id="MI:0335" refType="identity" refTypeAc="MI:0356"**/>

<**secondaryRef db="intact" dbAc="MI:0469" id="EBI-540564" refType="identity" refTypeAc="MI:0356"**/>

<**secondaryRef db="pubmed" dbAc="MI:0446" id="14755292" refType="primary-reference" refTypeAc="MI:0358"**/>

</**xref**>

</**startStatus**>

<**begin position="637"**/>

<**endStatus**>

<**names**>

<**shortLabel**>certain</**shortLabel**>

<**fullName**>certain sequence position</**fullName**>

<**alias type="synonym" typeAc="MI:1041"**>certain</**alias**>

</**names**>

<**xref**>

<**primaryRef db="psi-mi" dbAc="MI:0488" id="MI:0335" refType="identity" refTypeAc="MI:0356"**/>

<**secondaryRef db="intact" dbAc="MI:0469" id="EBI-540564" refType="identity" refTypeAc="MI:0356"**/>

<**secondaryRef db="pubmed" dbAc="MI:0446" id="14755292" refType="primary-reference" refTypeAc="MI:0358"**/>

</**xref**>

</**endStatus**>

<**end position="637"**/>

<**resultingSequence**>

<**originalSequence**>P</**originalSequence**>

<**newSequence**>Y</**newSequence**>

</**resultingSequence**>

</**featureRange**>

</**featureRangeList**>

</**feature**>

<**feature id="13"**>

<**names**>

<**shortLabel**>ser641ala</**shortLabel**>

</**names**>

<**xref**>

<**primaryRef db="intact" dbAc="MI:0469" id="EBI-12553606" refType="identity" refTypeAc="MI:0356"**/>

</**xref**>

<**featureType**>

<**names**>

<**shortLabel**>mutation</**shortLabel**>

<**fullName**>mutation</**fullName**>

</**names**>

<**xref**>

<**primaryRef db="psi-mi" dbAc="MI:0488" id="MI:0118" refType="identity" refTypeAc="MI:0356"**/>

<**secondaryRef db="intact" dbAc="MI:0469" id="EBI-456558" refType="identity" refTypeAc="MI:0356"**/>

<**secondaryRef db="pubmed" dbAc="MI:0446" id="14755292" refType="primary-reference" refTypeAc="MI:0358"**/>

</**xref**>

</**featureType**>

<**featureRangeList**>

<**featureRange**>

<**startStatus**>

<**names**>

<**shortLabel**>certain</**shortLabel**>

<**fullName**>certain sequence position</**fullName**>

<**alias type="synonym" typeAc="MI:1041"**>certain</**alias**>

</**names**>

<**xref**>

<**primaryRef db="psi-mi" dbAc="MI:0488" id="MI:0335" refType="identity" refTypeAc="MI:0356"**/>

<**secondaryRef db="intact" dbAc="MI:0469" id="EBI-540564" refType="identity" refTypeAc="MI:0356"**/>

<**secondaryRef db="pubmed" dbAc="MI:0446" id="14755292" refType="primary-reference" refTypeAc="MI:0358"**/>

</**xref**>

</**startStatus**>

<**begin position="641"**/>

<**endStatus**>

<**names**>

<**shortLabel**>certain</**shortLabel**>

<**fullName**>certain sequence position</**fullName**>

<**alias type="synonym" typeAc="MI:1041"**>certain</**alias**>

</**names**>

<**xref**>

<**primaryRef db="psi-mi" dbAc="MI:0488" id="MI:0335" refType="identity" refTypeAc="MI:0356"**/>

<**secondaryRef db="intact" dbAc="MI:0469" id="EBI-540564" refType="identity" refTypeAc="MI:0356"**/>

<**secondaryRef db="pubmed" dbAc="MI:0446" id="14755292" refType="primary-reference" refTypeAc="MI:0358"**/>

</**xref**>

</**endStatus**>

<**end position="641"**/>

<**resultingSequence**>

<**originalSequence**>S</**originalSequence**>

<**newSequence**>A</**newSequence**>

</**resultingSequence**>

</**featureRange**>

</**featureRangeList**>

</**feature**>

</**featureList**>

<**hostOrganismList**>

<**hostOrganism ncbiTaxId="-2"**>

<**names**>

<**shortLabel**>chemical synthesis</**shortLabel**>

<**fullName**>Chemical synthesis (Chemical synthesis)</**fullName**>

</**names**>

</**hostOrganism**>

</**hostOrganismList**>

</**participant**>

</**participantList**>

<**interactionType**>

<**names**>

<**shortLabel**>phosphorylation</**shortLabel**>

<**fullName**>phosphorylation reaction</**fullName**>

</**names**>

<**xref**>

<**primaryRef db="psi-mi" dbAc="MI:0488" id="MI:0217" refType="identity" refTypeAc="MI:0356"**/>

<**secondaryRef db="intact" dbAc="MI:0469" id="EBI-49922" refType="identity" refTypeAc="MI:0356"**/>

<**secondaryRef db="resid" dbAc="MI:0248" id="AA0033" refType="see-also" refTypeAc="MI:0361"**/>

<**secondaryRef db="resid" dbAc="MI:0248" id="AA0034" refType="see-also" refTypeAc="MI:0361"**/>

<**secondaryRef db="resid" dbAc="MI:0248" id="AA0035" refType="see-also" refTypeAc="MI:0361"**/>

<**secondaryRef db="resid" dbAc="MI:0248" id="AA0036" refType="see-also" refTypeAc="MI:0361"**/>

<**secondaryRef db="resid" dbAc="MI:0248" id="AA0037" refType="see-also" refTypeAc="MI:0361"**/>

<**secondaryRef db="resid" dbAc="MI:0248" id="AA0038" refType="see-also" refTypeAc="MI:0361"**/>

<**secondaryRef db="resid" dbAc="MI:0248" id="AA0039" refType="see-also" refTypeAc="MI:0361"**/>

<**secondaryRef db="resid" dbAc="MI:0248" id="AA0222" refType="see-also" refTypeAc="MI:0361"**/>

<**secondaryRef db="pubmed" dbAc="MI:0446" id="14755292" refType="primary-reference" refTypeAc="MI:0358"**/>

<**secondaryRef db="go" dbAc="MI:0448" id="GO:0016310" refType="see-also" refTypeAc="MI:0361"**/>

</**xref**>

</**interactionType**>

<**attributeList**>

<**attribute name="figure legend" nameAc="MI:0599"**>Fig. 5</**attribute**>

</**attributeList**>

</**interaction**>

</**interactionList**>

</**entry**>

</**entrySet**>
