# Supplementary material for: Encompassing new use cases - level 3.0 of the HUPO-PSI format for molecular interactions
Source: BMC Bioinformatics. 2018 Apr 11;19:134. doi: 10.1186/s12859-018-2118-1 (PMC5896046; doi:10.1186/s12859-018-2118-1)
Supplement: Supplementary file 4 — Representation of multiple feature detection methods and feature roles (use case 1.3c, use case 1.3d). (https://github.com/HUPO-PSI/miXML/blob/master/3.0/pub/Appendix%205.docx). (DOCX 68 kb) [file 12859_2018_2118_MOESM4_ESM.docx]

# Multiple feature detection methods and feature roles

#### Publication id: 28054552

#### Interaction: EBI-14988426

#### Feature: EBI-14988463

*<?***xml version="1.0" encoding="UTF-8"***?>*

<**entrySet xmlns:xsi="http://www.w3.org/2001/XMLSchema-instance"**

**xmlns="http://psi.hupo.org/mi/mif300"**

**xsi:schemaLocation="http://psi.hupo.org/mi/mif300 https://raw.githubusercontent.com/HUPO-PSI/miXML/master/3.0/src/MIF300.xsd"**

**level="3" version="0" minorVersion="0"**>
 <**entry**>
 <**source releaseDate="2017-06-05"**>
 <**names**>
 <**shortLabel**>IntAct</**shortLabel**>
 <**fullName**>European Bioinformatics Institute</**fullName**>
 <**alias type="synonym" typeAc="MI:1041"**>IntAct</**alias**>
 </**names**>
 <**bibref**>
 <**xref**>
 <**primaryRef db="pubmed" dbAc="MI:0446" id="14681455" refType="primary-reference" refTypeAc="MI:0358"**/>
 </**xref**>
 </**bibref**>
 <**xref**>
 <**primaryRef db="psi-mi" dbAc="MI:0488" id="MI:0469" refType="identity" refTypeAc="MI:0356"**/>
 <**secondaryRef db="intact" dbAc="MI:0469" id="EBI-10" refType="identity" refTypeAc="MI:0356"**/>
 <**secondaryRef db="pubmed" dbAc="MI:0446" id="14681455" refType="primary-reference" refTypeAc="MI:0358"**/>
 <**secondaryRef db="pubmed" dbAc="MI:0446" id="22121220" refType="method reference" refTypeAc="MI:0357"**/>
 <**secondaryRef db="pubmed" dbAc="MI:0446" id="19850723" refType="method reference" refTypeAc="MI:0357"**/>
 </**xref**>
 <**attributeList**>
 <**attribute name="url" nameAc="MI:0614"**>http://www.ebi.ac.uk/</**attribute**>
 <**attribute name="search-url" nameAc="MI:0615"**>http://www.ebi.ac.uk/intact/query/${ac}</**attribute**>
 <**attribute name="id-validation-regexp" nameAc="MI:0628"**>EBI-[0-9]+|IA:[0-9]+</**attribute**>
 <**attribute name="definition"**>INTerAction database (IntAct) provides an open source database and toolkit
 for the storage, presentation and analysis of molecular interactions.
 </**attribute**>
 <**attribute name="url" nameAc="MI:0614"**>http://www.ebi.ac.uk/intact</**attribute**>
 <**attribute name="postaladdress"**>European Bioinformatics Institute; Wellcome Trust Genome Campus;
 Hinxton, Cambridge; CB10 1SD; United Kingdom
 </**attribute**>
 <**attribute name="url" nameAc="MI:0614"**>http://www.ebi.ac.uk/intact/</**attribute**>
 </**attributeList**>
 </**source**>
 <**interactionList**>
 <**interaction id="1"**>
 <**names**>
 <**shortLabel**>hk1-src-3</**shortLabel**>
 </**names**>
 <**xref**>
 <**primaryRef db="intact" dbAc="MI:0469" id="EBI-14988426" refType="identity" refTypeAc="MI:0356"**/>
 </**xref**>
 <**experimentList**>
 <**experimentDescription id="2"**>
 <**names**>
 <**fullName**>c-Src phosphorylation and activation of hexokinase promotes tumorigenesis and metastasis.</**fullName**>
 </**names**>
 <**bibref**>
 <**xref**>
 <**primaryRef db="pubmed" dbAc="MI:0446" id="28054552" refType="primary-reference" refTypeAc="MI:0358"**/>
 <**secondaryRef db="intact" dbAc="MI:0469" id="EBI-14988372" refType="identity" refTypeAc="MI:0356"**/>
 </**xref**>
 <**attributeList**>
 <**attribute name="publication title" nameAc="MI:1091"**>c-Src phosphorylation and
 activation of hexokinase promotes tumorigenesis and metastasis.
 </**attribute**>
 <**attribute name="journal" nameAc="MI:0885"**>Nature communications</**attribute**>
 <**attribute name="publication year" nameAc="MI:0886"**>2017</**attribute**>
 <**attribute name="curation depth" nameAc="MI:0955"**>imex curation</**attribute**>
 <**attribute name="imex curation" nameAc="MI:0959"**/>
 <**attribute name="author-list" nameAc="MI:0636"**>Zhang J., Wang S., Jiang B., Huang L., Ji
 Z., Li X., Zhou H., Han A., Chen A., Wu Y., Ma H., Zhao W., Zhao Q., Xie C., Sun X.,
 Zhou Y., Huang H., Suleman M., Lin F., Zhou L., Tian F., Jin M., Cai Y., Zhang N.,
 Li Q.
 </**attribute**>
 <**attribute name="contact-email" nameAc="MI:0634"**>liqinxi@xmu.edu.cn</**attribute**>
 </**attributeList**>
 </**bibref**>
 <**xref**>
 <**primaryRef db="pubmed" dbAc="MI:0446" id="28054552" refType="primary-reference" refTypeAc="MI:0358"**/>
 </**xref**>
 <**hostOrganismList**>
 <**hostOrganism ncbiTaxId="-1"**>
 <**names**>
 <**shortLabel**>in vitro</**shortLabel**>
 <**fullName**>In vitro</**fullName**>
 </**names**>
 </**hostOrganism**>
 </**hostOrganismList**>
 <**interactionDetectionMethod**>
 <**names**>
 <**shortLabel**>pull down</**shortLabel**>
 <**fullName**>pull down</**fullName**>
 </**names**>
 <**xref**>
 <**primaryRef db="psi-mi" dbAc="MI:0488" id="MI:0096" refType="identity" refTypeAc="MI:0356"**/>
 <**secondaryRef db="intact" dbAc="MI:0469" id="EBI-1223" refType="identity" refTypeAc="MI:0356"**/>
 <**secondaryRef db="pubmed" dbAc="MI:0446" id="14755292" refType="primary-reference" refTypeAc="MI:0358"**/>
 </**xref**>
 </**interactionDetectionMethod**>
 <**participantIdentificationMethod**>
 <**names**>
 <**shortLabel**>anti tag western</**shortLabel**>
 <**fullName**>anti tag western blot</**fullName**>
 </**names**>
 <**xref**>
 <**primaryRef db="psi-mi" dbAc="MI:0488" id="MI:0705" refType="identity" refTypeAc="MI:0356"**/>
 <**secondaryRef db="intact" dbAc="MI:0469" id="EBI-967832" refType="identity" refTypeAc="MI:0356"**/>
 <**secondaryRef db="pubmed" dbAc="MI:0446" id="14755292" refType="primary-reference" refTypeAc="MI:0358"**/>
 </**xref**>
 </**participantIdentificationMethod**>
 <**attributeList**>
 <**attribute name="contact-email" nameAc="MI:0634"**>liqinxi@xmu.edu.cn</**attribute**>
 <**attribute name="journal" nameAc="MI:0885"**>Nature communications</**attribute**>
 <**attribute name="publication year" nameAc="MI:0886"**>2017</**attribute**>
 <**attribute name="author-list" nameAc="MI:0636"**>Zhang J., Wang S., Jiang B., Huang L., Ji Z.,
 Li X., Zhou H., Han A., Chen A., Wu Y., Ma H., Zhao W., Zhao Q., Xie C., Sun X., Zhou
 Y., Huang H., Suleman M., Lin F., Zhou L., Tian F., Jin M., Cai Y., Zhang N., Li Q.
 </**attribute**>
 <**attribute name="curation depth" nameAc="MI:0955"**>IMEx</**attribute**>
 </**attributeList**>
 </**experimentDescription**>
 </**experimentList**>
 <**participantList**>
 <**participant id="3"**>
 <**interactor id="4"**>
 <**names**>
 <**shortLabel**>src_mouse</**shortLabel**>
 <**fullName**>Neuronal proto-oncogene tyrosine-protein kinase Src</**fullName**>
 <**alias type="gene name" typeAc="MI:0301"**>Src</**alias**>
 <**alias type="gene name synonym" typeAc="MI:0302"**>pp60c-src</**alias**>
 <**alias type="gene name synonym" typeAc="MI:0302"**>Proto-oncogene c-Src</**alias**>
 </**names**>
 <**xref**>
 <**primaryRef db="uniprotkb" dbAc="MI:0486" id="P05480" version="SP_88" refType="identity" refTypeAc="MI:0356"**/>
 <**secondaryRef db="uniprotkb" dbAc="MI:0486" id="Q2M4I4" version="SP_129" refType="secondary-ac" refTypeAc="MI:0360"**/>
 <**secondaryRef db="intact" dbAc="MI:0469" id="EBI-298680" refType="identity" refTypeAc="MI:0356"**/>
 <**secondaryRef db="go" dbAc="MI:0448" id="GO:0071803"**/>
 <**secondaryRef db="go" dbAc="MI:0448" id="GO:0005634"**/>
 <**secondaryRef db="go" dbAc="MI:0448" id="GO:0007049"**/>
 <**secondaryRef db="go" dbAc="MI:0448" id="GO:0005829"**/>
 <**secondaryRef db="go" dbAc="MI:0448" id="GO:0005743"**/>
 <**secondaryRef db="go" dbAc="MI:0448" id="GO:0005886"**/>
 <**secondaryRef db="go" dbAc="MI:0448" id="GO:0005524"**/>
 <**secondaryRef db="go" dbAc="MI:0448" id="GO:0020037"**/>
 <**secondaryRef db="go" dbAc="MI:0448" id="GO:0004715"**/>
 <**secondaryRef db="go" dbAc="MI:0448" id="GO:0045453"**/>
 <**secondaryRef db="go" dbAc="MI:0448" id="GO:0060444"**/>
 <**secondaryRef db="go" dbAc="MI:0448" id="GO:0016477"**/>
 <**secondaryRef db="go" dbAc="MI:0448" id="GO:0030900"**/>
 <**secondaryRef db="go" dbAc="MI:0448" id="GO:0048477"**/>
 <**secondaryRef db="go" dbAc="MI:0448" id="GO:0070374"**/>
 <**secondaryRef db="go" dbAc="MI:0448" id="GO:0033146"**/>
 <**secondaryRef db="go" dbAc="MI:0448" id="GO:0060065"**/>
 <**secondaryRef db="refseq" dbAc="MI:0481" id="NP_001020566.1"**/>
 <**secondaryRef db="refseq" dbAc="MI:0481" id="NP_033297.2"**/>
 <**secondaryRef db="interpro" dbAc="MI:0449" id="IPR011009"**/>
 <**secondaryRef db="interpro" dbAc="MI:0449" id="IPR000719"**/>
 <**secondaryRef db="interpro" dbAc="MI:0449" id="IPR017441"**/>
 <**secondaryRef db="interpro" dbAc="MI:0449" id="IPR001245"**/>
 <**secondaryRef db="interpro" dbAc="MI:0449" id="IPR000980"**/>
 <**secondaryRef db="interpro" dbAc="MI:0449" id="IPR001452"**/>
 <**secondaryRef db="interpro" dbAc="MI:0449" id="IPR008266"**/>
 <**secondaryRef db="interpro" dbAc="MI:0449" id="IPR020635"**/>
 <**secondaryRef db="go" dbAc="MI:0448" id="GO:0004713"**/>
 <**secondaryRef db="go" dbAc="MI:0448" id="GO:0090263"**/>
 <**secondaryRef db="go" dbAc="MI:0448" id="GO:0036035"**/>
 <**secondaryRef db="go" dbAc="MI:0448" id="GO:0043393"**/>
 <**secondaryRef db="go" dbAc="MI:0448" id="GO:0004672"**/>
 <**secondaryRef db="go" dbAc="MI:0448" id="GO:0006468"**/>
 <**secondaryRef db="go" dbAc="MI:0448" id="GO:0016301"**/>
 <**secondaryRef db="go" dbAc="MI:0448" id="GO:0018108"**/>
 <**secondaryRef db="go" dbAc="MI:0448" id="GO:0019904"**/>
 <**secondaryRef db="go" dbAc="MI:0448" id="GO:0046875"**/>
 <**secondaryRef db="go" dbAc="MI:0448" id="GO:0005884"**/>
 <**secondaryRef db="go" dbAc="MI:0448" id="GO:0032587"**/>
 <**secondaryRef db="go" dbAc="MI:0448" id="GO:0034446"**/>
 <**secondaryRef db="go" dbAc="MI:0448" id="GO:0036120"**/>
 <**secondaryRef db="go" dbAc="MI:0448" id="GO:0048471"**/>
 <**secondaryRef db="go" dbAc="MI:0448" id="GO:0060491"**/>
 <**secondaryRef db="go" dbAc="MI:0448" id="GO:0035556"**/>
 <**secondaryRef db="ensembl" dbAc="MI:0476" id="ENSMUST00000092576"**/>
 <**secondaryRef db="ensembl" dbAc="MI:0476" id="ENSMUST00000109529"**/>
 <**secondaryRef db="go" dbAc="MI:0448" id="GO:0048008"**/>
 <**secondaryRef db="go" dbAc="MI:0448" id="GO:0048010"**/>
 <**secondaryRef db="go" dbAc="MI:0448" id="GO:0048013"**/>
 <**secondaryRef db="go" dbAc="MI:0448" id="GO:0051427"**/>
 <**secondaryRef db="go" dbAc="MI:0448" id="GO:0051726"**/>
 <**secondaryRef db="reactome" dbAc="MI:0467" id="R-MMU-375165"**/>
 <**secondaryRef db="reactome" dbAc="MI:0467" id="R-MMU-418886"**/>
 <**secondaryRef db="reactome" dbAc="MI:0467" id="R-MMU-6811558"**/>
 <**secondaryRef db="go" dbAc="MI:0448" id="GO:0007411"**/>
 <**secondaryRef db="go" dbAc="MI:0448" id="GO:0031234"**/>
 <**secondaryRef db="go" dbAc="MI:0448" id="GO:0038083"**/>
 <**secondaryRef db="go" dbAc="MI:0448" id="GO:0042127"**/>
 <**secondaryRef db="go" dbAc="MI:0448" id="GO:0045087"**/>
 <**secondaryRef db="ensembl" dbAc="MI:0476" id="ENSMUSG00000027646"**/>
 <**secondaryRef db="ensembl" dbAc="MI:0476" id="ENSMUSP00000090237"**/>
 <**secondaryRef db="ensembl" dbAc="MI:0476" id="ENSMUSP00000105155"**/>
 <**secondaryRef db="reactome" dbAc="MI:0467" id="R-MMU-1227986"**/>
 <**secondaryRef db="reactome" dbAc="MI:0467" id="R-MMU-1295596"**/>
 <**secondaryRef db="reactome" dbAc="MI:0467" id="R-MMU-1433557"**/>
 <**secondaryRef db="reactome" dbAc="MI:0467" id="R-MMU-1433559"**/>
 <**secondaryRef db="go" dbAc="MI:0448" id="GO:0016337"**/>
 <**secondaryRef db="go" dbAc="MI:0448" id="GO:0018105"**/>
 <**secondaryRef db="go" dbAc="MI:0448" id="GO:0031667"**/>
 <**secondaryRef db="go" dbAc="MI:0448" id="GO:0031954"**/>
 <**secondaryRef db="go" dbAc="MI:0448" id="GO:0032148"**/>
 <**secondaryRef db="go" dbAc="MI:0448" id="GO:0032211"**/>
 <**secondaryRef db="go" dbAc="MI:0448" id="GO:0032869"**/>
 <**secondaryRef db="go" dbAc="MI:0448" id="GO:0034332"**/>
 <**secondaryRef db="go" dbAc="MI:0448" id="GO:0042493"**/>
 <**secondaryRef db="go" dbAc="MI:0448" id="GO:0005102"**/>
 <**secondaryRef db="go" dbAc="MI:0448" id="GO:0005737"**/>
 <**secondaryRef db="go" dbAc="MI:0448" id="GO:0005739"**/>
 <**secondaryRef db="go" dbAc="MI:0448" id="GO:0019899"**/>
 <**secondaryRef db="go" dbAc="MI:0448" id="GO:0031648"**/>
 <**secondaryRef db="go" dbAc="MI:0448" id="GO:0034614"**/>
 <**secondaryRef db="go" dbAc="MI:0448" id="GO:0042169"**/>
 <**secondaryRef db="go" dbAc="MI:0448" id="GO:0043066"**/>
 <**secondaryRef db="go" dbAc="MI:0448" id="GO:0044325"**/>
 <**secondaryRef db="go" dbAc="MI:0448" id="GO:0046777"**/>
 <**secondaryRef db="go" dbAc="MI:0448" id="GO:0051219"**/>
 <**secondaryRef db="go" dbAc="MI:0448" id="GO:0008283"**/>
 <**secondaryRef db="go" dbAc="MI:0448" id="GO:0009612"**/>
 <**secondaryRef db="go" dbAc="MI:0448" id="GO:0009615"**/>
 <**secondaryRef db="go" dbAc="MI:0448" id="GO:0010447"**/>
 <**secondaryRef db="go" dbAc="MI:0448" id="GO:0010641"**/>
 <**secondaryRef db="go" dbAc="MI:0448" id="GO:0010907"**/>
 <**secondaryRef db="go" dbAc="MI:0448" id="GO:0014069"**/>
 <**secondaryRef db="go" dbAc="MI:0448" id="GO:0014911"**/>
 <**secondaryRef db="go" dbAc="MI:0448" id="GO:0071560"**/>
 <**secondaryRef db="go" dbAc="MI:0448" id="GO:0042542"**/>
 <**secondaryRef db="go" dbAc="MI:0448" id="GO:0043005"**/>
 <**secondaryRef db="go" dbAc="MI:0448" id="GO:0043065"**/>
 <**secondaryRef db="go" dbAc="MI:0448" id="GO:0043406"**/>
 <**secondaryRef db="go" dbAc="MI:0448" id="GO:0043552"**/>
 <**secondaryRef db="go" dbAc="MI:0448" id="GO:0045056"**/>
 <**secondaryRef db="go" dbAc="MI:0448" id="GO:0045737"**/>
 <**secondaryRef db="go" dbAc="MI:0448" id="GO:0045785"**/>
 <**secondaryRef db="go" dbAc="MI:0448" id="GO:0045892"**/>
 <**secondaryRef db="go" dbAc="MI:0448" id="GO:0045893"**/>
 <**secondaryRef db="go" dbAc="MI:0448" id="GO:0046628"**/>
 <**secondaryRef db="go" dbAc="MI:0448" id="GO:0048011"**/>
 <**secondaryRef db="go" dbAc="MI:0448" id="GO:0050715"**/>
 <**secondaryRef db="go" dbAc="MI:0448" id="GO:0051385"**/>
 <**secondaryRef db="go" dbAc="MI:0448" id="GO:0051602"**/>
 <**secondaryRef db="go" dbAc="MI:0448" id="GO:0051895"**/>
 <**secondaryRef db="go" dbAc="MI:0448" id="GO:0051974"**/>
 <**secondaryRef db="go" dbAc="MI:0448" id="GO:0071222"**/>
 <**secondaryRef db="go" dbAc="MI:0448" id="GO:0071393"**/>
 <**secondaryRef db="go" dbAc="MI:0448" id="GO:0007173"**/>
 <**secondaryRef db="go" dbAc="MI:0448" id="GO:0050847"**/>
 <**secondaryRef db="go" dbAc="MI:0448" id="GO:0070851"**/>
 <**secondaryRef db="go" dbAc="MI:0448" id="GO:0071375"**/>
 <**secondaryRef db="go" dbAc="MI:0448" id="GO:0086098"**/>
 <**secondaryRef db="go" dbAc="MI:0448" id="GO:0097110"**/>
 <**secondaryRef db="go" dbAc="MI:0448" id="GO:2001237"**/>
 <**secondaryRef db="go" dbAc="MI:0448" id="GO:2001243"**/>
 <**secondaryRef db="reactome" dbAc="MI:0467" id="R-MMU-177929"**/>
 <**secondaryRef db="go" dbAc="MI:0448" id="GO:0005764"**/>
 <**secondaryRef db="go" dbAc="MI:0448" id="GO:0005770"**/>
 <**secondaryRef db="go" dbAc="MI:0448" id="GO:0005901"**/>
 <**secondaryRef db="go" dbAc="MI:0448" id="GO:0007179"**/>
 <**secondaryRef db="go" dbAc="MI:0448" id="GO:0007229"**/>
 <**secondaryRef db="go" dbAc="MI:0448" id="GO:0010632"**/>
 <**secondaryRef db="go" dbAc="MI:0448" id="GO:0016310"**/>
 <**secondaryRef db="go" dbAc="MI:0448" id="GO:0022407"**/>
 <**secondaryRef db="go" dbAc="MI:0448" id="GO:0032463"**/>
 <**secondaryRef db="go" dbAc="MI:0448" id="GO:0043149"**/>
 <**secondaryRef db="go" dbAc="MI:0448" id="GO:0043154"**/>
 <**secondaryRef db="go" dbAc="MI:0448" id="GO:0051897"**/>
 <**secondaryRef db="go" dbAc="MI:0448" id="GO:0051902"**/>
 <**secondaryRef db="go" dbAc="MI:0448" id="GO:0070062"**/>
 <**secondaryRef db="go" dbAc="MI:0448" id="GO:0070555"**/>
 <**secondaryRef db="go" dbAc="MI:0448" id="GO:2000641"**/>
 <**secondaryRef db="go" dbAc="MI:0448" id="GO:2000811"**/>
 <**secondaryRef db="go" dbAc="MI:0448" id="GO:2001286"**/>
 <**secondaryRef db="go" dbAc="MI:0448" id="GO:0071498"**/>
 <**secondaryRef db="reactome" dbAc="MI:0467" id="R-MMU-180292"**/>
 <**secondaryRef db="reactome" dbAc="MI:0467" id="R-MMU-186763"**/>
 <**secondaryRef db="reactome" dbAc="MI:0467" id="R-MMU-191647"**/>
 <**secondaryRef db="reactome" dbAc="MI:0467" id="R-MMU-354192"**/>
 <**secondaryRef db="reactome" dbAc="MI:0467" id="R-MMU-354194"**/>
 <**secondaryRef db="reactome" dbAc="MI:0467" id="R-MMU-372708"**/>
 <**secondaryRef db="reactome" dbAc="MI:0467" id="R-MMU-389356"**/>
 <**secondaryRef db="reactome" dbAc="MI:0467" id="R-MMU-389513"**/>
 <**secondaryRef db="reactome" dbAc="MI:0467" id="R-MMU-3928662"**/>
 <**secondaryRef db="reactome" dbAc="MI:0467" id="R-MMU-3928663"**/>
 <**secondaryRef db="reactome" dbAc="MI:0467" id="R-MMU-3928664"**/>
 <**secondaryRef db="reactome" dbAc="MI:0467" id="R-MMU-3928665"**/>
 <**secondaryRef db="reactome" dbAc="MI:0467" id="R-MMU-418592"**/>
 <**secondaryRef db="reactome" dbAc="MI:0467" id="R-MMU-418885"**/>
 <**secondaryRef db="reactome" dbAc="MI:0467" id="R-MMU-430116"**/>
 <**secondaryRef db="reactome" dbAc="MI:0467" id="R-MMU-437239"**/>
 <**secondaryRef db="reactome" dbAc="MI:0467" id="R-MMU-4420097"**/>
 <**secondaryRef db="reactome" dbAc="MI:0467" id="R-MMU-456926"**/>
 <**secondaryRef db="reactome" dbAc="MI:0467" id="R-MMU-5218921"**/>
 <**secondaryRef db="reactome" dbAc="MI:0467" id="R-MMU-5607764"**/>
 <**secondaryRef db="reactome" dbAc="MI:0467" id="R-MMU-5663220"**/>
 <**secondaryRef db="reactome" dbAc="MI:0467" id="R-MMU-5673000"**/>
 <**secondaryRef db="go" dbAc="MI:0448" id="GO:0010634"**/>
 <**secondaryRef db="go" dbAc="MI:0448" id="GO:2000394"**/>
 <**secondaryRef db="go" dbAc="MI:0448" id="GO:0019900"**/>
 <**secondaryRef db="go" dbAc="MI:0448" id="GO:0071902"**/>
 <**secondaryRef db="go" dbAc="MI:0448" id="GO:0071398"**/>
 <**secondaryRef db="go" dbAc="MI:0448" id="GO:0071456"**/>
 <**secondaryRef db="go" dbAc="MI:0448" id="GO:2000573"**/>
 <**secondaryRef db="go" dbAc="MI:0448" id="GO:0010954"**/>
 <**secondaryRef db="go" dbAc="MI:0448" id="GO:0050731"**/>
 <**secondaryRef db="go" dbAc="MI:0448" id="GO:1900182"**/>
 <**secondaryRef db="go" dbAc="MI:0448" id="GO:0005080"**/>
 <**secondaryRef db="go" dbAc="MI:0448" id="GO:0005158"**/>
 <**secondaryRef db="go" dbAc="MI:0448" id="GO:0008022"**/>
 <**secondaryRef db="go" dbAc="MI:0448" id="GO:0030331"**/>
 <**secondaryRef db="go" dbAc="MI:0448" id="GO:0031625"**/>
 <**secondaryRef db="go" dbAc="MI:0448" id="GO:0045296"**/>
 <**secondaryRef db="go" dbAc="MI:0448" id="GO:0071253"**/>
 <**secondaryRef db="go" dbAc="MI:0448" id="GO:0002102"**/>
 <**secondaryRef db="go" dbAc="MI:0448" id="GO:0051057"**/>
 <**secondaryRef db="reactome" dbAc="MI:0467" id="R-MMU-8853659"**/>
 <**secondaryRef db="reactome" dbAc="MI:0467" id="R-MMU-8874081"**/>
 </**xref**>
 <**interactorType**>
 <**names**>
 <**shortLabel**>protein</**shortLabel**>
 <**fullName**>protein</**fullName**>
 </**names**>
 <**xref**>
 <**primaryRef db="psi-mi" dbAc="MI:0488" id="MI:0326" refType="identity" refTypeAc="MI:0356"**/>
 <**secondaryRef db="intact" dbAc="MI:0469" id="EBI-619654" refType="identity" refTypeAc="MI:0356"**/>
 <**secondaryRef db="pubmed" dbAc="MI:0446" id="14755292" refType="primary-reference" refTypeAc="MI:0358"**/>
 <**secondaryRef db="so" dbAc="MI:0601" id="SO:0000358" refType="see-also" refTypeAc="MI:0361"**/>
 </**xref**>
 </**interactorType**>
 <**organism ncbiTaxId="10090"**>
 <**names**>
 <**shortLabel**>mouse</**shortLabel**>
 <**fullName**>Mus musculus</**fullName**>
 <**alias type="synonym" typeAc="MI:1041"**>Mouse</**alias**>
 </**names**>
 </**organism**>
 <**sequence**>
 MGSNKSKPKDASQRRRSLEPSENVHGAGGAFPASQTPSKPASADGHRGPSAAFVPPAAEPKLFGGFNSSDTVTSPQRAGPLAGGVTTFVALYDYESRTETDLSFKKGERLQIVNNTRKVDVREGDWWLAHSLSTGQTGYIPSNYVAPSDSIQAEEWYFGKITRRESERLLLNAENPRGTFLVRESETTKGAYCLSVSDFDNAKGLNVKHYKIRKLDSGGFYITSRTQFNSLQQLVAYYSKHADGLCHRLTTVCPTSKPQTQGLAKDAWEIPRESLRLEVKLGQGCFGEVWMGTWNGTTRVAIKTLKPGTMSPEAFLQEAQVMKKLRHEKLVQLYAVVSEEPIYIVTEYMNKGSLLDFLKGETGKYLRLPQLVDMSAQIASGMAYVERMNYVHRDLRAANILVGENLVCKVADFGLARLIEDNEYTARQGAKFPIKWTAPEAALYGRFTIKSDVWSFGILLTELTTKGRVPYPGMVNREVLDQVERGYRMPCPPECPESLHDLMCQCWRKEPEERPTFEYLQAFLEDYFTSTEPQYQPGENL
 </**sequence**>
 <**attributeList**>
 <**attribute name="crc64"**>0534AF027783BCCF</**attribute**>
 </**attributeList**>
 </**interactor**>
 <**biologicalRole**>
 <**names**>
 <**shortLabel**>unspecified role</**shortLabel**>
 <**fullName**>unspecified role</**fullName**>
 </**names**>
 <**xref**>
 <**primaryRef db="psi-mi" dbAc="MI:0488" id="MI:0499" refType="identity" refTypeAc="MI:0356"**/>
 <**secondaryRef db="intact" dbAc="MI:0469" id="EBI-77781" refType="identity" refTypeAc="MI:0356"**/>
 <**secondaryRef db="pubmed" dbAc="MI:0446" id="14755292" refType="primary-reference" refTypeAc="MI:0358"**/>
 </**xref**>
 </**biologicalRole**>
 <**experimentalRoleList**>
 <**experimentalRole**>
 <**names**>
 <**shortLabel**>bait</**shortLabel**>
 <**fullName**>bait</**fullName**>
 </**names**>
 <**xref**>
 <**primaryRef db="psi-mi" dbAc="MI:0488" id="MI:0496" refType="identity" refTypeAc="MI:0356"**/>
 <**secondaryRef db="intact" dbAc="MI:0469" id="EBI-49" refType="identity" refTypeAc="MI:0356"**/>
 <**secondaryRef db="pubmed" dbAc="MI:0446" id="14755292" refType="primary-reference" refTypeAc="MI:0358"**/>
 </**xref**>
 </**experimentalRole**>
 </**experimentalRoleList**>
 <**featureList**>
 <**feature id="5"**>
 <**names**>
 <**shortLabel**>n-terminal</**shortLabel**>
 </**names**>
 <**xref**>
 <**primaryRef db="intact" dbAc="MI:0469" id="EBI-14988430" refType="identity" refTypeAc="MI:0356"**/>
 </**xref**>
 <**featureType**>
 <**names**>
 <**shortLabel**>gst tag</**shortLabel**>
 <**fullName**>glutathione s tranferase tag</**fullName**>
 <**alias type="go synonym" typeAc="MI:0303"**>glutathione S-tranferase tag</**alias**>
 </**names**>
 <**xref**>
 <**primaryRef db="psi-mi" dbAc="MI:0488" id="MI:0519" refType="identity" refTypeAc="MI:0356"**/>
 <**secondaryRef db="intact" dbAc="MI:0469" id="EBI-456510" refType="identity" refTypeAc="MI:0356"**/>
 <**secondaryRef db="pubmed" dbAc="MI:0446" id="14755292" refType="primary-reference" refTypeAc="MI:0358"**/>
 </**xref**>
 </**featureType**>
 <**featureRangeList**>
 <**featureRange**>
 <**startStatus**>
 <**names**>
 <**shortLabel**>n-term range</**shortLabel**>
 <**fullName**>n-terminal range</**fullName**>
 <**alias type="synonym" typeAc="MI:1041"**>n-term range</**alias**>
 </**names**>
 <**xref**>
 <**primaryRef db="psi-mi" dbAc="MI:0488" id="MI:1040" refType="identity" refTypeAc="MI:0356"**/>
 <**secondaryRef db="intact" dbAc="MI:0469" id="EBI-2929795" refType="identity" refTypeAc="MI:0356"**/>
 <**secondaryRef db="pubmed" dbAc="MI:0446" id="14760721" refType="primary-reference" refTypeAc="MI:0358"**/>
 </**xref**>
 </**startStatus**>
 <**endStatus**>
 <**names**>
 <**shortLabel**>n-term range</**shortLabel**>
 <**fullName**>n-terminal range</**fullName**>
 <**alias type="synonym" typeAc="MI:1041"**>n-term range</**alias**>
 </**names**>
 <**xref**>
 <**primaryRef db="psi-mi" dbAc="MI:0488" id="MI:1040" refType="identity" refTypeAc="MI:0356"**/>
 <**secondaryRef db="intact" dbAc="MI:0469" id="EBI-2929795" refType="identity" refTypeAc="MI:0356"**/>
 <**secondaryRef db="pubmed" dbAc="MI:0446" id="14760721" refType="primary-reference" refTypeAc="MI:0358"**/>
 </**xref**>
 </**endStatus**>
 </**featureRange**>
 </**featureRangeList**>
 </**feature**>
 </**featureList**>
 <**hostOrganismList**>
 <**hostOrganism ncbiTaxId="83333"**>
 <**names**>
 <**shortLabel**>ecoli</**shortLabel**>
 <**fullName**>Escherichia coli (strain K12)</**fullName**>
 </**names**>
 </**hostOrganism**>
 </**hostOrganismList**>
 </**participant**>
 <**participant id="6"**>
 <**interactor id="7"**>
 <**names**>
 <**shortLabel**>hxk1_human</**shortLabel**>
 <**fullName**>Hexokinase-1</**fullName**>
 <**alias type="gene name synonym" typeAc="MI:0302"**>Hexokinase type I</**alias**>
 <**alias type="gene name synonym" typeAc="MI:0302"**>Brain form hexokinase</**alias**>
 <**alias type="gene name" typeAc="MI:0301"**>HK1</**alias**>
 </**names**>
 <**xref**>
 <**primaryRef db="uniprotkb" dbAc="MI:0486" id="P19367" version="SP_103" refType="identity" refTypeAc="MI:0356"**/>
 <**secondaryRef db="uniprotkb" dbAc="MI:0486" id="Q5VTC3" version="SP_103" refType="secondary-ac" refTypeAc="MI:0360"**/>
 <**secondaryRef db="uniprotkb" dbAc="MI:0486" id="Q96HC8" version="SP_103" refType="secondary-ac" refTypeAc="MI:0360"**/>
 <**secondaryRef db="uniprotkb" dbAc="MI:0486" id="Q9NNZ4" version="SP_103" refType="secondary-ac" refTypeAc="MI:0360"**/>
 <**secondaryRef db="uniprotkb" dbAc="MI:0486" id="O43443" version="SP_103" refType="secondary-ac" refTypeAc="MI:0360"**/>
 <**secondaryRef db="uniprotkb" dbAc="MI:0486" id="O43444" version="SP_103" refType="secondary-ac" refTypeAc="MI:0360"**/>
 <**secondaryRef db="uniprotkb" dbAc="MI:0486" id="O75574" version="SP_103" refType="secondary-ac" refTypeAc="MI:0360"**/>
 <**secondaryRef db="uniprotkb" dbAc="MI:0486" id="Q9NNZ5" version="SP_103" refType="secondary-ac" refTypeAc="MI:0360"**/>
 <**secondaryRef db="uniprotkb" dbAc="MI:0486" id="E9PCK0" version="SP_150" refType="secondary-ac" refTypeAc="MI:0360"**/>
 <**secondaryRef db="intact" dbAc="MI:0469" id="EBI-713162" refType="identity" refTypeAc="MI:0356"**/>
 <**secondaryRef db="rcsb pdb" dbAc="MI:0460" id="4F9O"**/>
 <**secondaryRef db="refseq" dbAc="MI:0481" id="NP_000179.2"**/>
 <**secondaryRef db="refseq" dbAc="MI:0481" id="NP_277031.1"**/>
 <**secondaryRef db="refseq" dbAc="MI:0481" id="NP_277032.1"**/>
 <**secondaryRef db="refseq" dbAc="MI:0481" id="NP_277033.1"**/>
 <**secondaryRef db="refseq" dbAc="MI:0481" id="NP_277035.2"**/>
 <**secondaryRef db="interpro" dbAc="MI:0449" id="IPR001312"**/>
 <**secondaryRef db="interpro" dbAc="MI:0449" id="IPR022673"**/>
 <**secondaryRef db="interpro" dbAc="MI:0449" id="IPR019807"**/>
 <**secondaryRef db="interpro" dbAc="MI:0449" id="IPR022672"**/>
 <**secondaryRef db="go" dbAc="MI:0448" id="GO:0005829"**/>
 <**secondaryRef db="go" dbAc="MI:0448" id="GO:0005741"**/>
 <**secondaryRef db="go" dbAc="MI:0448" id="GO:0005524"**/>
 <**secondaryRef db="go" dbAc="MI:0448" id="GO:0015758"**/>
 <**secondaryRef db="go" dbAc="MI:0448" id="GO:0006096"**/>
 <**secondaryRef db="rcsb pdb" dbAc="MI:0460" id="1CZA"**/>
 <**secondaryRef db="rcsb pdb" dbAc="MI:0460" id="1DGK"**/>
 <**secondaryRef db="rcsb pdb" dbAc="MI:0460" id="1HKB"**/>
 <**secondaryRef db="rcsb pdb" dbAc="MI:0460" id="1HKC"**/>
 <**secondaryRef db="rcsb pdb" dbAc="MI:0460" id="1QHA"**/>
 <**secondaryRef db="go" dbAc="MI:0448" id="GO:0001678"**/>
 <**secondaryRef db="go" dbAc="MI:0448" id="GO:0004340"**/>
 <**secondaryRef db="go" dbAc="MI:0448" id="GO:0008865"**/>
 <**secondaryRef db="go" dbAc="MI:0448" id="GO:0019158"**/>
 <**secondaryRef db="ensembl" dbAc="MI:0476" id="ENST00000298649"**/>
 <**secondaryRef db="ensembl" dbAc="MI:0476" id="ENST00000359426"**/>
 <**secondaryRef db="ensembl" dbAc="MI:0476" id="ENST00000360289"**/>
 <**secondaryRef db="go" dbAc="MI:0448" id="GO:0061621"**/>
 <**secondaryRef db="go" dbAc="MI:0448" id="GO:0005739"**/>
 <**secondaryRef db="rcsb pdb" dbAc="MI:0460" id="4FOE"**/>
 <**secondaryRef db="rcsb pdb" dbAc="MI:0460" id="4FOI"**/>
 <**secondaryRef db="rcsb pdb" dbAc="MI:0460" id="4FPA"**/>
 <**secondaryRef db="rcsb pdb" dbAc="MI:0460" id="4FPB"**/>
 <**secondaryRef db="go" dbAc="MI:0448" id="GO:0004396"**/>
 <**secondaryRef db="refseq" dbAc="MI:0481" id="NP_001309293.1"**/>
 <**secondaryRef db="ensembl" dbAc="MI:0476" id="ENSG00000156515"**/>
 <**secondaryRef db="ensembl" dbAc="MI:0476" id="ENSP00000298649"**/>
 <**secondaryRef db="ensembl" dbAc="MI:0476" id="ENSP00000352398"**/>
 <**secondaryRef db="ensembl" dbAc="MI:0476" id="ENSP00000353433"**/>
 <**secondaryRef db="refseq" dbAc="MI:0481" id="XP_011538034.1"**/>
 <**secondaryRef db="go" dbAc="MI:0448" id="GO:0005536"**/>
 <**secondaryRef db="reactome" dbAc="MI:0467" id="R-HSA-70153"**/>
 <**secondaryRef db="reactome" dbAc="MI:0467" id="R-HSA-70171"**/>
 <**secondaryRef db="ensembl" dbAc="MI:0476" id="ENSP00000402103"**/>
 <**secondaryRef db="ensembl" dbAc="MI:0476" id="ENST00000448642"**/>
 <**secondaryRef db="go" dbAc="MI:0448" id="GO:0072655"**/>
 <**secondaryRef db="go" dbAc="MI:0448" id="GO:0072656"**/>
 <**secondaryRef db="go" dbAc="MI:0448" id="GO:0045121"**/>
 <**secondaryRef db="go" dbAc="MI:0448" id="GO:0097228"**/>
 </**xref**>
 <**interactorType**>
 <**names**>
 <**shortLabel**>protein</**shortLabel**>
 <**fullName**>protein</**fullName**>
 </**names**>
 <**xref**>
 <**primaryRef db="psi-mi" dbAc="MI:0488" id="MI:0326" refType="identity" refTypeAc="MI:0356"**/>
 <**secondaryRef db="intact" dbAc="MI:0469" id="EBI-619654" refType="identity" refTypeAc="MI:0356"**/>
 <**secondaryRef db="pubmed" dbAc="MI:0446" id="14755292" refType="primary-reference" refTypeAc="MI:0358"**/>
 <**secondaryRef db="so" dbAc="MI:0601" id="SO:0000358" refType="see-also" refTypeAc="MI:0361"**/>
 </**xref**>
 </**interactorType**>
 <**organism ncbiTaxId="9606"**>
 <**names**>
 <**shortLabel**>human</**shortLabel**>
 <**fullName**>Homo sapiens</**fullName**>
 <**alias type="synonym" typeAc="MI:1041"**>Human</**alias**>
 </**names**>
 </**organism**>
 <**sequence**>
 MIAAQLLAYYFTELKDDQVKKIDKYLYAMRLSDETLIDIMTRFRKEMKNGLSRDFNPTATVKMLPTFVRSIPDGSEKGDFIALDLGGSSFRILRVQVNHEKNQNVHMESEVYDTPENIVHGSGSQLFDHVAECLGDFMEKRKIKDKKLPVGFTFSFPCQQSKIDEAILITWTKRFKASGVEGADVVKLLNKAIKKRGDYDANIVAVVNDTVGTMMTCGYDDQHCEVGLIIGTGTNACYMEELRHIDLVEGDEGRMCINTEWGAFGDDGSLEDIRTEFDREIDRGSLNPGKQLFEKMVSGMYLGELVRLILVKMAKEGLLFEGRITPELLTRGKFNTSDVSAIEKNKEGLHNAKEILTRLGVEPSDDDCVSVQHVCTIVSFRSANLVAATLGAILNRLRDNKGTPRLRTTVGVDGSLYKTHPQYSRRFHKTLRRLVPDSDVRFLLSESGSGKGAAMVTAVAYRLAEQHRQIEETLAHFHLTKDMLLEVKKRMRAEMELGLRKQTHNNAVVKMLPSFVRRTPDGTENGDFLALDLGGTNFRVLLVKIRSGKKRTVEMHNKIYAIPIEIMQGTGEELFDHIVSCISDFLDYMGIKGPRMPLGFTFSFPCQQTSLDAGILITWTKGFKATDCVGHDVVTLLRDAIKRREEFDLDVVAVVNDTVGTMMTCAYEEPTCEVGLIVGTGSNACYMEEMKNVEMVEGDQGQMCINMEWGAFGDNGCLDDIRTHYDRLVDEYSLNAGKQRYEKMISGMYLGEIVRNILIDFTKKGFLFRGQISETLKTRGIFETKFLSQIESDRLALLQVRAILQQLGLNSTCDDSILVKTVCGVVSRRAAQLCGAGMAAVVDKIRENRGLDRLNVTVGVDGTLYKLHPHFSRIMHQTVKELSPKCNVSFLLSEDGSGKGAALITAVGVRLRTEASS
 </**sequence**>
 <**attributeList**>
 <**attribute name="crc64"**>F29A6837531C0594</**attribute**>
 </**attributeList**>
 </**interactor**>
 <**biologicalRole**>
 <**names**>
 <**shortLabel**>unspecified role</**shortLabel**>
 <**fullName**>unspecified role</**fullName**>
 </**names**>
 <**xref**>
 <**primaryRef db="psi-mi" dbAc="MI:0488" id="MI:0499" refType="identity" refTypeAc="MI:0356"**/>
 <**secondaryRef db="intact" dbAc="MI:0469" id="EBI-77781" refType="identity" refTypeAc="MI:0356"**/>
 <**secondaryRef db="pubmed" dbAc="MI:0446" id="14755292" refType="primary-reference" refTypeAc="MI:0358"**/>
 </**xref**>
 </**biologicalRole**>
 <**experimentalRoleList**>
 <**experimentalRole**>
 <**names**>
 <**shortLabel**>prey</**shortLabel**>
 <**fullName**>prey</**fullName**>
 </**names**>
 <**xref**>
 <**primaryRef db="psi-mi" dbAc="MI:0488" id="MI:0498" refType="identity" refTypeAc="MI:0356"**/>
 <**secondaryRef db="intact" dbAc="MI:0469" id="EBI-58" refType="identity" refTypeAc="MI:0356"**/>
 <**secondaryRef db="pubmed" dbAc="MI:0446" id="14755292" refType="primary-reference" refTypeAc="MI:0358"**/>
 </**xref**>
 </**experimentalRole**>
 </**experimentalRoleList**>
 <**featureList**>
 <**feature id="8"**>
 <**names**>
 <**shortLabel**>n-terminal</**shortLabel**>
 </**names**>
 <**xref**>
 <**primaryRef db="intact" dbAc="MI:0469" id="EBI-14988433" refType="identity" refTypeAc="MI:0356"**/>
 </**xref**>
 <**featureType**>
 <**names**>
 <**shortLabel**>his tag</**shortLabel**>
 <**fullName**>his tag</**fullName**>
 <**alias type="go synonym" typeAc="MI:0303"**>Hexa-His-tag</**alias**>
 <**alias type="go synonym" typeAc="MI:0303"**>6-His-tag</**alias**>
 <**alias type="go synonym" typeAc="MI:0303"**>Histidine-tag</**alias**>
 </**names**>
 <**xref**>
 <**primaryRef db="psi-mi" dbAc="MI:0488" id="MI:0521" refType="identity" refTypeAc="MI:0356"**/>
 <**secondaryRef db="intact" dbAc="MI:0469" id="EBI-456516" refType="identity" refTypeAc="MI:0356"**/>
 <**secondaryRef db="pubmed" dbAc="MI:0446" id="14755292" refType="primary-reference" refTypeAc="MI:0358"**/>
 </**xref**>
 </**featureType**>
 <**featureRangeList**>
 <**featureRange**>
 <**startStatus**>
 <**names**>
 <**shortLabel**>undetermined</**shortLabel**>
 <**fullName**>undetermined sequence position</**fullName**>
 </**names**>
 <**xref**>
 <**primaryRef db="psi-mi" dbAc="MI:0488" id="MI:0339" refType="identity" refTypeAc="MI:0356"**/>
 <**secondaryRef db="intact" dbAc="MI:0469" id="EBI-448295" refType="identity" refTypeAc="MI:0356"**/>
 <**secondaryRef db="pubmed" dbAc="MI:0446" id="14755292" refType="primary-reference" refTypeAc="MI:0358"**/>
 </**xref**>
 </**startStatus**>
 <**endStatus**>
 <**names**>
 <**shortLabel**>undetermined</**shortLabel**>
 <**fullName**>undetermined sequence position</**fullName**>
 </**names**>
 <**xref**>
 <**primaryRef db="psi-mi" dbAc="MI:0488" id="MI:0339" refType="identity" refTypeAc="MI:0356"**/>
 <**secondaryRef db="intact" dbAc="MI:0469" id="EBI-448295" refType="identity" refTypeAc="MI:0356"**/>
 <**secondaryRef db="pubmed" dbAc="MI:0446" id="14755292" refType="primary-reference" refTypeAc="MI:0358"**/>
 </**xref**>
 </**endStatus**>
 </**featureRange**>
 </**featureRangeList**>
 </**feature**>
 <**feature id="9"**>
 <**names**>
 <**shortLabel**>c-terminal</**shortLabel**>
 </**names**>
 <**xref**>
 <**primaryRef db="intact" dbAc="MI:0469" id="EBI-14988435" refType="identity" refTypeAc="MI:0356"**/>
 </**xref**>
 <**featureType**>
 <**names**>
 <**shortLabel**>his tag</**shortLabel**>
 <**fullName**>his tag</**fullName**>
 <**alias type="go synonym" typeAc="MI:0303"**>Hexa-His-tag</**alias**>
 <**alias type="go synonym" typeAc="MI:0303"**>6-His-tag</**alias**>
 <**alias type="go synonym" typeAc="MI:0303"**>Histidine-tag</**alias**>
 </**names**>
 <**xref**>
 <**primaryRef db="psi-mi" dbAc="MI:0488" id="MI:0521" refType="identity" refTypeAc="MI:0356"**/>
 <**secondaryRef db="intact" dbAc="MI:0469" id="EBI-456516" refType="identity" refTypeAc="MI:0356"**/>
 <**secondaryRef db="pubmed" dbAc="MI:0446" id="14755292" refType="primary-reference" refTypeAc="MI:0358"**/>
 </**xref**>
 </**featureType**>
 <**featureRangeList**>
 <**featureRange**>
 <**startStatus**>
 <**names**>
 <**shortLabel**>c-term range</**shortLabel**>
 <**fullName**>C-terminal range</**fullName**>
 </**names**>
 <**xref**>
 <**primaryRef db="psi-mi" dbAc="MI:0488" id="MI:1039" refType="identity" refTypeAc="MI:0356"**/>
 <**secondaryRef db="intact" dbAc="MI:0469" id="EBI-2929799" refType="identity" refTypeAc="MI:0356"**/>
 <**secondaryRef db="pubmed" dbAc="MI:0446" id="14760721" refType="primary-reference" refTypeAc="MI:0358"**/>
 </**xref**>
 </**startStatus**>
 <**endStatus**>
 <**names**>
 <**shortLabel**>c-term range</**shortLabel**>
 <**fullName**>C-terminal range</**fullName**>
 </**names**>
 <**xref**>
 <**primaryRef db="psi-mi" dbAc="MI:0488" id="MI:1039" refType="identity" refTypeAc="MI:0356"**/>
 <**secondaryRef db="intact" dbAc="MI:0469" id="EBI-2929799" refType="identity" refTypeAc="MI:0356"**/>
 <**secondaryRef db="pubmed" dbAc="MI:0446" id="14760721" refType="primary-reference" refTypeAc="MI:0358"**/>
 </**xref**>
 </**endStatus**>
 </**featureRange**>
 </**featureRangeList**>
 </**feature**>
 <**feature id="10"**>
 <**names**>
 <**shortLabel**>region</**shortLabel**>
 </**names**>
 <**xref**>
 <**primaryRef db="intact" dbAc="MI:0469" id="EBI-14988459" refType="identity" refTypeAc="MI:0356"**/>
 </**xref**>
 <**featureType**>
 <**names**>
 <**shortLabel**>ophosres</**shortLabel**>
 <**fullName**>N-phosphorylated residue</**fullName**>
 </**names**>
 <**xref**>
 <**primaryRef db="psi-mod" dbAc="MI:0897" id="MOD:01456" refType="identity" refTypeAc="MI:0356"**/>
 <**secondaryRef db="intact" dbAc="MI:0469" id="EBI-5527781" refType="identity" refTypeAc="MI:0356"**/>
 <**secondaryRef db="pubmed" dbAc="MI:0446" id="18688235" refType="primary-reference" refTypeAc="MI:0358"**/>
 </**xref**>
 </**featureType**>
 <**featureDetectionMethod**>
 <**names**>
 <**shortLabel**>western blot</**shortLabel**>
 <**fullName**>western blot</**fullName**>
 <**alias type="go synonym" typeAc="MI:0303"**>Immuno blot</**alias**>
 </**names**>
 <**xref**>
 <**primaryRef db="psi-mi" dbAc="MI:0488" id="MI:0113" refType="identity" refTypeAc="MI:0356"**/>
 <**secondaryRef db="intact" dbAc="MI:0469" id="EBI-456832" refType="identity" refTypeAc="MI:0356"**/>
 <**secondaryRef db="pubmed" dbAc="MI:0446" id="14755292" refType="primary-reference" refTypeAc="MI:0358"**/>
 </**xref**>
 </**featureDetectionMethod**>
 <**featureRangeList**>
 <**featureRange**>
 <**startStatus**>
 <**names**>
 <**shortLabel**>undetermined</**shortLabel**>
 <**fullName**>undetermined sequence position</**fullName**>
 </**names**>
 <**xref**>
 <**primaryRef db="psi-mi" dbAc="MI:0488" id="MI:0339" refType="identity" refTypeAc="MI:0356"**/>
 <**secondaryRef db="intact" dbAc="MI:0469" id="EBI-448295" refType="identity" refTypeAc="MI:0356"**/>
 <**secondaryRef db="pubmed" dbAc="MI:0446" id="14755292" refType="primary-reference" refTypeAc="MI:0358"**/>
 </**xref**>
 </**startStatus**>
 <**endStatus**>
 <**names**>
 <**shortLabel**>undetermined</**shortLabel**>
 <**fullName**>undetermined sequence position</**fullName**>
 </**names**>
 <**xref**>
 <**primaryRef db="psi-mi" dbAc="MI:0488" id="MI:0339" refType="identity" refTypeAc="MI:0356"**/>
 <**secondaryRef db="intact" dbAc="MI:0469" id="EBI-448295" refType="identity" refTypeAc="MI:0356"**/>
 <**secondaryRef db="pubmed" dbAc="MI:0446" id="14755292" refType="primary-reference" refTypeAc="MI:0358"**/>
 </**xref**>
 </**endStatus**>
 </**featureRange**>
 </**featureRangeList**>
 </**feature**>
 <**feature id="11"**>
 <**names**>
 <**shortLabel**>tyr732phe</**shortLabel**>
 </**names**>
 <**xref**>
 <**primaryRef db="intact" dbAc="MI:0469" id="EBI-14988461" refType="identity" refTypeAc="MI:0356"**/>
 </**xref**>
 <**featureType**>
 <**names**>
 <**shortLabel**>mutation disrupting strength</**shortLabel**>
 <**fullName**>mutation disrupting interaction strength</**fullName**>
 </**names**>
 <**xref**>
 <**primaryRef db="psi-mi" dbAc="MI:0488" id="MI:1128" refType="identity" refTypeAc="MI:0356"**/>
 <**secondaryRef db="intact" dbAc="MI:0469" id="EBI-5528001" refType="identity" refTypeAc="MI:0356"**/>
 <**secondaryRef db="pubmed" dbAc="MI:0446" id="14755292" refType="primary-reference" refTypeAc="MI:0358"**/>
 </**xref**>
 </**featureType**>
 <**featureRangeList**>
 <**featureRange**>
 <**startStatus**>
 <**names**>
 <**shortLabel**>certain</**shortLabel**>
 <**fullName**>certain sequence position</**fullName**>
 <**alias type="synonym" typeAc="MI:1041"**>certain</**alias**>
 </**names**>
 <**xref**>
 <**primaryRef db="psi-mi" dbAc="MI:0488" id="MI:0335" refType="identity" refTypeAc="MI:0356"**/>
 <**secondaryRef db="intact" dbAc="MI:0469" id="EBI-540564" refType="identity" refTypeAc="MI:0356"**/>
 <**secondaryRef db="pubmed" dbAc="MI:0446" id="14755292" refType="primary-reference" refTypeAc="MI:0358"**/>
 </**xref**>
 </**startStatus**>
 <**begin position="732"**/>
 <**endStatus**>
 <**names**>
 <**shortLabel**>certain</**shortLabel**>
 <**fullName**>certain sequence position</**fullName**>
 <**alias type="synonym" typeAc="MI:1041"**>certain</**alias**>
 </**names**>
 <**xref**>
 <**primaryRef db="psi-mi" dbAc="MI:0488" id="MI:0335" refType="identity" refTypeAc="MI:0356"**/>
 <**secondaryRef db="intact" dbAc="MI:0469" id="EBI-540564" refType="identity" refTypeAc="MI:0356"**/>
 <**secondaryRef db="pubmed" dbAc="MI:0446" id="14755292" refType="primary-reference" refTypeAc="MI:0358"**/>
 </**xref**>
 </**endStatus**>
 <**end position="732"**/>
 <**resultingSequence**>
 <**originalSequence**>Y</**originalSequence**>
 <**newSequence**>F</**newSequence**>
 </**resultingSequence**>
 </**featureRange**>
 </**featureRangeList**>
 </**feature**>
 <**feature id="12"**>
 <**names**>
 <**shortLabel**>ser-732</**shortLabel**>
 </**names**>
 <**xref**>
 <**primaryRef db="intact" dbAc="MI:0469" id="EBI-14988463" refType="identity" refTypeAc="MI:0356"**/>
 </**xref**>
 <**featureType**>
 <**names**>
 <**shortLabel**>optyr</**shortLabel**>
 <**fullName**>O4'-phospho-L-tyrosine</**fullName**>
 <**alias type="synonym" typeAc="MI:1041"**>(2S)-2-amino-3-(4-phosphonooxyphenyl)propanoic acid</**alias**>
 <**alias type="synonym" typeAc="MI:1041"**>2-azanyl-3-(4-phosphonooxyphenyl)propanoic acid</**alias**>
 <**alias type="synonym" typeAc="MI:1041"**>MOD_RES Phosphotyrosine</**alias**>
 <**alias type="synonym" typeAc="MI:1041"**>O4-phosphotyrosine</**alias**>
 <**alias type="synonym" typeAc="MI:1041"**>tyrosine phosphate</**alias**>
 <**alias type="synonym" typeAc="MI:1041"**>2-amino-3-(4-hydroxyphenyl)propanoic acid 4'-phosphate</**alias**>
 <**alias type="synonym" typeAc="MI:1041"**>O4'-phospho-L-tyrosine</**alias**>
 <**alias type="synonym" typeAc="MI:1041"**>O4'-phosphorylated L-tyrosine</**alias**>
 </**names**>
 <**xref**>
 <**primaryRef db="psi-mod" dbAc="MI:0897" id="MOD:00048" refType="identity" refTypeAc="MI:0356"**/>
 <**secondaryRef db="intact" dbAc="MI:0469" id="EBI-456748" refType="identity" refTypeAc="MI:0356"**/>
 <**secondaryRef db="pubmed" dbAc="MI:0446" id="10226369" refType="primary-reference" refTypeAc="MI:0358"**/>
 <**secondaryRef db="pubmed" dbAc="MI:0446" id="1725475" refType="primary-reference" refTypeAc="MI:0358"**/>
 <**secondaryRef db="resid" dbAc="MI:0248" id="AA0039" refType="see-also" refTypeAc="MI:0361"**/>
 </**xref**>
 </**featureType**>
 <**featureDetectionMethod**>
 <**names**>
 <**shortLabel**>western blot</**shortLabel**>
 <**fullName**>western blot</**fullName**>
 <**alias type="go synonym" typeAc="MI:0303"**>Immuno blot</**alias**>
 </**names**>
 <**xref**>
 <**primaryRef db="psi-mi" dbAc="MI:0488" id="MI:0113" refType="identity" refTypeAc="MI:0356"**/>
 <**secondaryRef db="intact" dbAc="MI:0469" id="EBI-456832" refType="identity" refTypeAc="MI:0356"**/>
 <**secondaryRef db="pubmed" dbAc="MI:0446" id="14755292" refType="primary-reference" refTypeAc="MI:0358"**/>
 </**xref**>
 </**featureDetectionMethod**>
 <**featureDetectionMethod**>
 <**names**>
 <**shortLabel**>mutation analysis</**shortLabel**>
 <**fullName**>mutation analysis</**fullName**>
 </**names**>
 <**xref**>
 <**primaryRef db="psi-mi" dbAc="MI:0488" id="MI:0074" refType="identity" refTypeAc="MI:0356"**/>
 <**secondaryRef db="intact" dbAc="MI:0469" id="EBI-456810" refType="identity" refTypeAc="MI:0356"**/>
 <**secondaryRef db="pubmed" dbAc="MI:0446" id="14755292" refType="primary-reference" refTypeAc="MI:0358"**/>
 </**xref**>
 </**featureDetectionMethod**>
 <**featureRangeList**>
 <**featureRange**>
 <**startStatus**>
 <**names**>
 <**shortLabel**>certain</**shortLabel**>
 <**fullName**>certain sequence position</**fullName**>
 <**alias type="synonym" typeAc="MI:1041"**>certain</**alias**>
 </**names**>
 <**xref**>
 <**primaryRef db="psi-mi" dbAc="MI:0488" id="MI:0335" refType="identity" refTypeAc="MI:0356"**/>
 <**secondaryRef db="intact" dbAc="MI:0469" id="EBI-540564" refType="identity" refTypeAc="MI:0356"**/>
 <**secondaryRef db="pubmed" dbAc="MI:0446" id="14755292" refType="primary-reference" refTypeAc="MI:0358"**/>
 </**xref**>
 </**startStatus**>
 <**begin position="732"**/>
 <**endStatus**>
 <**names**>
 <**shortLabel**>certain</**shortLabel**>
 <**fullName**>certain sequence position</**fullName**>
 <**alias type="synonym" typeAc="MI:1041"**>certain</**alias**>
 </**names**>
 <**xref**>
 <**primaryRef db="psi-mi" dbAc="MI:0488" id="MI:0335" refType="identity" refTypeAc="MI:0356"**/>
 <**secondaryRef db="intact" dbAc="MI:0469" id="EBI-540564" refType="identity" refTypeAc="MI:0356"**/>
 <**secondaryRef db="pubmed" dbAc="MI:0446" id="14755292" refType="primary-reference" refTypeAc="MI:0358"**/>
 </**xref**>
 </**endStatus**>
 <**end position="732"**/>
 </**featureRange**>
 </**featureRangeList**>
 <**featureRole**>
 <**names**>
 <**shortLabel**>prerequisite-ptm</**shortLabel**>
 <**fullName**>prerequisite-ptm</**fullName**>
 </**names**>
 <**xref**>
 <**primaryRef db="psi-mi" dbAc="MI:0488" id="MI:0638" refType="identity" refTypeAc="MI:0356"**/>
 <**secondaryRef db="intact" dbAc="MI:0469" id="EBI-872" refType="identity" refTypeAc="MI:0356"**/>
 <**secondaryRef db="pubmed" dbAc="MI:0446" id="14755292" refType="primary-reference" refTypeAc="MI:0358"**/>
 </**xref**>
 </**featureRole**>
 </**feature**>
 </**featureList**>
 <**hostOrganismList**>
 <**hostOrganism ncbiTaxId="83333"**>
 <**names**>
 <**shortLabel**>ecoli</**shortLabel**>
 <**fullName**>Escherichia coli (strain K12)</**fullName**>
 </**names**>
 </**hostOrganism**>
 </**hostOrganismList**>
 </**participant**>
 </**participantList**>
 <**interactionType**>
 <**names**>
 <**shortLabel**>physical association</**shortLabel**>
 <**fullName**>physical association</**fullName**>
 </**names**>
 <**xref**>
 <**primaryRef db="psi-mi" dbAc="MI:0488" id="MI:0915" refType="identity" refTypeAc="MI:0356"**/>
 <**secondaryRef db="intact" dbAc="MI:0469" id="EBI-1813147" refType="identity" refTypeAc="MI:0356"**/>
 <**secondaryRef db="pubmed" dbAc="MI:0446" id="14755292" refType="primary-reference" refTypeAc="MI:0358"**/>
 </**xref**>
 </**interactionType**>
 <**attributeList**>
 <**attribute name="source-text"**>In vitro GST-pull down assay also confirmed the direct interaction
 between His-HK1 and GST-c-Src, as indicated by coomassie brilliant blue staining (Fig. 1e, left
 panel) and western blot (Fig. 1e, right panel)
 </**attribute**>
 <**attribute name="figure legend" nameAc="MI:0599"**>Fig. 1E</**attribute**>
 </**attributeList**>
 </**interaction**>
 </**interactionList**>
 </**entry**>
</**entrySet**>

#### Publication: 28054552

#### Interaction: EBI-14988396

#### Feature: EBI-14988456 and EBI-14988471

*<?***xml version='1.0' encoding='UTF-8'***?>*<**entrySet xmlns:xsi="http://www.w3.org/2001/XMLSchema-instance" xmlns="http://psi.hupo.org/mi/mif300"
 xsi:schemaLocation="http://psi.hupo.org/mi/mif300 http://psidev.cvs.sourceforge.net/viewvc/psidev/psi/mi/rel30/src/MIF300.xsd"
 level="3" version="0" minorVersion="0"**>
<**entry**>
 <**source releaseDate="2017-06-05"**>
 <**names**>
 <**shortLabel**>IntAct</**shortLabel**>
 <**fullName**>European Bioinformatics Institute</**fullName**>
 <**alias type="synonym" typeAc="MI:1041"**>IntAct</**alias**>
 </**names**>
 <**bibref**>
 <**xref**>
 <**primaryRef db="pubmed" dbAc="MI:0446" id="14681455" refType="primary-reference" refTypeAc="MI:0358"**/>
 </**xref**>
 </**bibref**>
 <**xref**>
 <**primaryRef db="psi-mi" dbAc="MI:0488" id="MI:0469" refType="identity" refTypeAc="MI:0356"**/>
 <**secondaryRef db="intact" dbAc="MI:0469" id="EBI-10" refType="identity" refTypeAc="MI:0356"**/>
 <**secondaryRef db="pubmed" dbAc="MI:0446" id="14681455" refType="primary-reference" refTypeAc="MI:0358"**/>
 <**secondaryRef db="pubmed" dbAc="MI:0446" id="22121220" refType="method reference" refTypeAc="MI:0357"**/>
 <**secondaryRef db="pubmed" dbAc="MI:0446" id="19850723" refType="method reference" refTypeAc="MI:0357"**/>
 </**xref**>
 <**attributeList**>
 <**attribute name="url" nameAc="MI:0614"**>http://www.ebi.ac.uk/</**attribute**>
 <**attribute name="search-url" nameAc="MI:0615"**>http://www.ebi.ac.uk/intact/query/${ac}</**attribute**>
 <**attribute name="id-validation-regexp" nameAc="MI:0628"**>EBI-[0-9]+|IA:[0-9]+</**attribute**>
 <**attribute name="definition"**>INTerAction database (IntAct) provides an open source database and toolkit
 for the storage, presentation and analysis of molecular interactions.
 </**attribute**>
 <**attribute name="url" nameAc="MI:0614"**>http://www.ebi.ac.uk/intact</**attribute**>
 <**attribute name="postaladdress"**>European Bioinformatics Institute; Wellcome Trust Genome Campus;
 Hinxton, Cambridge; CB10 1SD; United Kingdom
 </**attribute**>
 <**attribute name="url" nameAc="MI:0614"**>http://www.ebi.ac.uk/intact/</**attribute**>
 </**attributeList**>
 </**source**>
 <**experimentList**>
 <**experimentDescription id="1"**>
 <**names**>
 <**fullName**>c-Src phosphorylation and activation of hexokinase promotes tumorigenesis and metastasis.</**fullName**>
 </**names**>
 <**bibref**>
 <**xref**>
 <**primaryRef db="pubmed" dbAc="MI:0446" id="28054552" refType="primary-reference" refTypeAc="MI:0358"**/>
 <**secondaryRef db="intact" dbAc="MI:0469" id="EBI-14988372" refType="identity" refTypeAc="MI:0356"**/>
 </**xref**>
 <**attributeList**>
 <**attribute name="publication title" nameAc="MI:1091"**>c-Src phosphorylation and activation of
 hexokinase promotes tumorigenesis and metastasis.
 </**attribute**>
 <**attribute name="journal" nameAc="MI:0885"**>Nature communications</**attribute**>
 <**attribute name="publication year" nameAc="MI:0886"**>2017</**attribute**>
 <**attribute name="curation depth" nameAc="MI:0955"**>imex curation</**attribute**>
 <**attribute name="imex curation" nameAc="MI:0959"**/>
 <**attribute name="author-list" nameAc="MI:0636"**>Zhang J., Wang S., Jiang B., Huang L., Ji Z., Li
 X., Zhou H., Han A., Chen A., Wu Y., Ma H., Zhao W., Zhao Q., Xie C., Sun X., Zhou Y., Huang
 H., Suleman M., Lin F., Zhou L., Tian F., Jin M., Cai Y., Zhang N., Li Q.
 </**attribute**>
 <**attribute name="contact-email" nameAc="MI:0634"**>liqinxi@xmu.edu.cn</**attribute**>
 </**attributeList**>
 </**bibref**>
 <**xref**>
 <**primaryRef db="pubmed" dbAc="MI:0446" id="28054552" refType="primary-reference" refTypeAc="MI:0358"**/>
 </**xref**>
 <**hostOrganismList**>
 <**hostOrganism ncbiTaxId="9606"**>
 <**names**>
 <**shortLabel**>human-293t</**shortLabel**>
 <**fullName**>Homo sapiens 293 cells transformed with SV40 large T antigen</**fullName**>
 </**names**>
 <**cellType**>
 <**names**>
 <**shortLabel**>293t</**shortLabel**>
 <**fullName**>293 cells expressing SV40 large T antigen.</**fullName**>
 </**names**>
 <**xref**>
 <**primaryRef db="cabri" dbAc="MI:0246" id="ICLC HTL04001" refType="identity" refTypeAc="MI:0356"**/>
 <**secondaryRef db="intact" dbAc="MI:0469" id="IA:0074" refType="identity" refTypeAc="MI:0356"**/>
 <**secondaryRef db="cabri" dbAc="MI:0246" id="ACC 635" refType="identity" refTypeAc="MI:0356"**/>
 <**secondaryRef db="mint" dbAc="MI:0471" id="MINT-1891516" refType="identity" refTypeAc="MI:0356"**/>
 <**secondaryRef db="intact" dbAc="MI:0469" id="EBI-308000" refType="identity" refTypeAc="MI:0356"**/>
 <**secondaryRef db="pubmed" dbAc="MI:0446" id="3031469" refType="primary-reference" refTypeAc="MI:0358"**/>
 </**xref**>
 <**attributeList**>
 <**attribute name="comment" nameAc="MI:0612"**>The original designation of this cell line was 293tsA1609neo. It is not in Cabri,ATCC or HyperCLDB.</**attribute**>
 </**attributeList**>
 </**cellType**>
 </**hostOrganism**>
 </**hostOrganismList**>
 <**interactionDetectionMethod**>
 <**names**>
 <**shortLabel**>anti tag coip</**shortLabel**>
 <**fullName**>anti tag coimmunoprecipitation</**fullName**>
 </**names**>
 <**xref**>
 <**primaryRef db="psi-mi" dbAc="MI:0488" id="MI:0007" refType="identity" refTypeAc="MI:0356"**/>
 <**secondaryRef db="intact" dbAc="MI:0469" id="EBI-90" refType="identity" refTypeAc="MI:0356"**/>
 <**secondaryRef db="pubmed" dbAc="MI:0446" id="7708014" refType="primary-reference" refTypeAc="MI:0358"**/>
 </**xref**>
 </**interactionDetectionMethod**>
 <**participantIdentificationMethod**>
 <**names**>
 <**shortLabel**>anti tag western</**shortLabel**>
 <**fullName**>anti tag western blot</**fullName**>
 </**names**>
 <**xref**>
 <**primaryRef db="psi-mi" dbAc="MI:0488" id="MI:0705" refType="identity" refTypeAc="MI:0356"**/>
 <**secondaryRef db="intact" dbAc="MI:0469" id="EBI-967832" refType="identity" refTypeAc="MI:0356"**/>
 <**secondaryRef db="pubmed" dbAc="MI:0446" id="14755292" refType="primary-reference" refTypeAc="MI:0358"**/>
 </**xref**>
 </**participantIdentificationMethod**>
 <**attributeList**>
 <**attribute name="contact-email" nameAc="MI:0634"**>liqinxi@xmu.edu.cn</**attribute**>
 <**attribute name="journal" nameAc="MI:0885"**>Nature communications</**attribute**>
 <**attribute name="publication year" nameAc="MI:0886"**>2017</**attribute**>
 <**attribute name="author-list" nameAc="MI:0636"**>Zhang J., Wang S., Jiang B., Huang L., Ji Z., Li X., Zhou H., Han A., Chen A., Wu Y., Ma H., Zhao W., Zhao Q., Xie C., Sun X., Zhou Y., Huang H., Suleman M., Lin F., Zhou L., Tian F., Jin M., Cai Y., Zhang N., Li Q.</**attribute**>
 <**attribute name="curation depth" nameAc="MI:0955"**>IMEx</**attribute**>
 </**attributeList**>
 </**experimentDescription**>
 </**experimentList**>
 <**interactorList**>
 <**interactor id="2"**>
 <**names**>
 <**shortLabel**>src_mouse</**shortLabel**>
 <**fullName**>Neuronal proto-oncogene tyrosine-protein kinase Src</**fullName**>
 <**alias type="gene name" typeAc="MI:0301"**>Src</**alias**>
 <**alias type="gene name synonym" typeAc="MI:0302"**>pp60c-src</**alias**>
 <**alias type="gene name synonym" typeAc="MI:0302"**>Proto-oncogene c-Src</**alias**>
 </**names**>
 <**xref**>
 <**primaryRef db="uniprotkb" dbAc="MI:0486" id="P05480" version="SP_88" refType="identity" refTypeAc="MI:0356"**/>
 <**secondaryRef db="uniprotkb" dbAc="MI:0486" id="Q2M4I4" version="SP_129" refType="secondary-ac" refTypeAc="MI:0360"**/>
 <**secondaryRef db="intact" dbAc="MI:0469" id="EBI-298680" refType="identity" refTypeAc="MI:0356"**/>
 <**secondaryRef db="go" dbAc="MI:0448" id="GO:0071803"**/>
 <**secondaryRef db="go" dbAc="MI:0448" id="GO:0005634"**/>
 <**secondaryRef db="go" dbAc="MI:0448" id="GO:0007049"**/>
 <**secondaryRef db="go" dbAc="MI:0448" id="GO:0005829"**/>
 <**secondaryRef db="go" dbAc="MI:0448" id="GO:0005743"**/>
 <**secondaryRef db="go" dbAc="MI:0448" id="GO:0005886"**/>
 <**secondaryRef db="go" dbAc="MI:0448" id="GO:0005524"**/>
 <**secondaryRef db="go" dbAc="MI:0448" id="GO:0020037"**/>
 <**secondaryRef db="go" dbAc="MI:0448" id="GO:0004715"**/>
 <**secondaryRef db="go" dbAc="MI:0448" id="GO:0045453"**/>
 <**secondaryRef db="go" dbAc="MI:0448" id="GO:0060444"**/>
 <**secondaryRef db="go" dbAc="MI:0448" id="GO:0016477"**/>
 <**secondaryRef db="go" dbAc="MI:0448" id="GO:0030900"**/>
 <**secondaryRef db="go" dbAc="MI:0448" id="GO:0048477"**/>
 <**secondaryRef db="go" dbAc="MI:0448" id="GO:0070374"**/>
 <**secondaryRef db="go" dbAc="MI:0448" id="GO:0033146"**/>
 <**secondaryRef db="go" dbAc="MI:0448" id="GO:0060065"**/>
 <**secondaryRef db="refseq" dbAc="MI:0481" id="NP_001020566.1"**/>
 <**secondaryRef db="refseq" dbAc="MI:0481" id="NP_033297.2"**/>
 <**secondaryRef db="interpro" dbAc="MI:0449" id="IPR011009"**/>
 <**secondaryRef db="interpro" dbAc="MI:0449" id="IPR000719"**/>
 <**secondaryRef db="interpro" dbAc="MI:0449" id="IPR017441"**/>
 <**secondaryRef db="interpro" dbAc="MI:0449" id="IPR001245"**/>
 <**secondaryRef db="interpro" dbAc="MI:0449" id="IPR000980"**/>
 <**secondaryRef db="interpro" dbAc="MI:0449" id="IPR001452"**/>
 <**secondaryRef db="interpro" dbAc="MI:0449" id="IPR008266"**/>
 <**secondaryRef db="interpro" dbAc="MI:0449" id="IPR020635"**/>
 <**secondaryRef db="go" dbAc="MI:0448" id="GO:0004713"**/>
 <**secondaryRef db="go" dbAc="MI:0448" id="GO:0090263"**/>
 <**secondaryRef db="go" dbAc="MI:0448" id="GO:0036035"**/>
 <**secondaryRef db="go" dbAc="MI:0448" id="GO:0043393"**/>
 <**secondaryRef db="go" dbAc="MI:0448" id="GO:0004672"**/>
 <**secondaryRef db="go" dbAc="MI:0448" id="GO:0006468"**/>
 <**secondaryRef db="go" dbAc="MI:0448" id="GO:0016301"**/>
 <**secondaryRef db="go" dbAc="MI:0448" id="GO:0018108"**/>
 <**secondaryRef db="go" dbAc="MI:0448" id="GO:0019904"**/>
 <**secondaryRef db="go" dbAc="MI:0448" id="GO:0046875"**/>
 <**secondaryRef db="go" dbAc="MI:0448" id="GO:0005884"**/>
 <**secondaryRef db="go" dbAc="MI:0448" id="GO:0032587"**/>
 <**secondaryRef db="go" dbAc="MI:0448" id="GO:0034446"**/>
 <**secondaryRef db="go" dbAc="MI:0448" id="GO:0036120"**/>
 <**secondaryRef db="go" dbAc="MI:0448" id="GO:0048471"**/>
 <**secondaryRef db="go" dbAc="MI:0448" id="GO:0060491"**/>
 <**secondaryRef db="go" dbAc="MI:0448" id="GO:0035556"**/>
 <**secondaryRef db="ensembl" dbAc="MI:0476" id="ENSMUST00000092576"**/>
 <**secondaryRef db="ensembl" dbAc="MI:0476" id="ENSMUST00000109529"**/>
 <**secondaryRef db="go" dbAc="MI:0448" id="GO:0048008"**/>
 <**secondaryRef db="go" dbAc="MI:0448" id="GO:0048010"**/>
 <**secondaryRef db="go" dbAc="MI:0448" id="GO:0048013"**/>
 <**secondaryRef db="go" dbAc="MI:0448" id="GO:0051427"**/>
 <**secondaryRef db="go" dbAc="MI:0448" id="GO:0051726"**/>
 <**secondaryRef db="reactome" dbAc="MI:0467" id="R-MMU-375165"**/>
 <**secondaryRef db="reactome" dbAc="MI:0467" id="R-MMU-418886"**/>
 <**secondaryRef db="reactome" dbAc="MI:0467" id="R-MMU-6811558"**/>
 <**secondaryRef db="go" dbAc="MI:0448" id="GO:0007411"**/>
 <**secondaryRef db="go" dbAc="MI:0448" id="GO:0031234"**/>
 <**secondaryRef db="go" dbAc="MI:0448" id="GO:0038083"**/>
 <**secondaryRef db="go" dbAc="MI:0448" id="GO:0042127"**/>
 <**secondaryRef db="go" dbAc="MI:0448" id="GO:0045087"**/>
 <**secondaryRef db="ensembl" dbAc="MI:0476" id="ENSMUSG00000027646"**/>
 <**secondaryRef db="ensembl" dbAc="MI:0476" id="ENSMUSP00000090237"**/>
 <**secondaryRef db="ensembl" dbAc="MI:0476" id="ENSMUSP00000105155"**/>
 <**secondaryRef db="reactome" dbAc="MI:0467" id="R-MMU-1227986"**/>
 <**secondaryRef db="reactome" dbAc="MI:0467" id="R-MMU-1295596"**/>
 <**secondaryRef db="reactome" dbAc="MI:0467" id="R-MMU-1433557"**/>
 <**secondaryRef db="reactome" dbAc="MI:0467" id="R-MMU-1433559"**/>
 <**secondaryRef db="go" dbAc="MI:0448" id="GO:0016337"**/>
 <**secondaryRef db="go" dbAc="MI:0448" id="GO:0018105"**/>
 <**secondaryRef db="go" dbAc="MI:0448" id="GO:0031667"**/>
 <**secondaryRef db="go" dbAc="MI:0448" id="GO:0031954"**/>
 <**secondaryRef db="go" dbAc="MI:0448" id="GO:0032148"**/>
 <**secondaryRef db="go" dbAc="MI:0448" id="GO:0032211"**/>
 <**secondaryRef db="go" dbAc="MI:0448" id="GO:0032869"**/>
 <**secondaryRef db="go" dbAc="MI:0448" id="GO:0034332"**/>
 <**secondaryRef db="go" dbAc="MI:0448" id="GO:0042493"**/>
 <**secondaryRef db="go" dbAc="MI:0448" id="GO:0005102"**/>
 <**secondaryRef db="go" dbAc="MI:0448" id="GO:0005737"**/>
 <**secondaryRef db="go" dbAc="MI:0448" id="GO:0005739"**/>
 <**secondaryRef db="go" dbAc="MI:0448" id="GO:0019899"**/>
 <**secondaryRef db="go" dbAc="MI:0448" id="GO:0031648"**/>
 <**secondaryRef db="go" dbAc="MI:0448" id="GO:0034614"**/>
 <**secondaryRef db="go" dbAc="MI:0448" id="GO:0042169"**/>
 <**secondaryRef db="go" dbAc="MI:0448" id="GO:0043066"**/>
 <**secondaryRef db="go" dbAc="MI:0448" id="GO:0044325"**/>
 <**secondaryRef db="go" dbAc="MI:0448" id="GO:0046777"**/>
 <**secondaryRef db="go" dbAc="MI:0448" id="GO:0051219"**/>
 <**secondaryRef db="go" dbAc="MI:0448" id="GO:0008283"**/>
 <**secondaryRef db="go" dbAc="MI:0448" id="GO:0009612"**/>
 <**secondaryRef db="go" dbAc="MI:0448" id="GO:0009615"**/>
 <**secondaryRef db="go" dbAc="MI:0448" id="GO:0010447"**/>
 <**secondaryRef db="go" dbAc="MI:0448" id="GO:0010641"**/>
 <**secondaryRef db="go" dbAc="MI:0448" id="GO:0010907"**/>
 <**secondaryRef db="go" dbAc="MI:0448" id="GO:0014069"**/>
 <**secondaryRef db="go" dbAc="MI:0448" id="GO:0014911"**/>
 <**secondaryRef db="go" dbAc="MI:0448" id="GO:0071560"**/>
 <**secondaryRef db="go" dbAc="MI:0448" id="GO:0042542"**/>
 <**secondaryRef db="go" dbAc="MI:0448" id="GO:0043005"**/>
 <**secondaryRef db="go" dbAc="MI:0448" id="GO:0043065"**/>
 <**secondaryRef db="go" dbAc="MI:0448" id="GO:0043406"**/>
 <**secondaryRef db="go" dbAc="MI:0448" id="GO:0043552"**/>
 <**secondaryRef db="go" dbAc="MI:0448" id="GO:0045056"**/>
 <**secondaryRef db="go" dbAc="MI:0448" id="GO:0045737"**/>
 <**secondaryRef db="go" dbAc="MI:0448" id="GO:0045785"**/>
 <**secondaryRef db="go" dbAc="MI:0448" id="GO:0045892"**/>
 <**secondaryRef db="go" dbAc="MI:0448" id="GO:0045893"**/>
 <**secondaryRef db="go" dbAc="MI:0448" id="GO:0046628"**/>
 <**secondaryRef db="go" dbAc="MI:0448" id="GO:0048011"**/>
 <**secondaryRef db="go" dbAc="MI:0448" id="GO:0050715"**/>
 <**secondaryRef db="go" dbAc="MI:0448" id="GO:0051385"**/>
 <**secondaryRef db="go" dbAc="MI:0448" id="GO:0051602"**/>
 <**secondaryRef db="go" dbAc="MI:0448" id="GO:0051895"**/>
 <**secondaryRef db="go" dbAc="MI:0448" id="GO:0051974"**/>
 <**secondaryRef db="go" dbAc="MI:0448" id="GO:0071222"**/>
 <**secondaryRef db="go" dbAc="MI:0448" id="GO:0071393"**/>
 <**secondaryRef db="go" dbAc="MI:0448" id="GO:0007173"**/>
 <**secondaryRef db="go" dbAc="MI:0448" id="GO:0050847"**/>
 <**secondaryRef db="go" dbAc="MI:0448" id="GO:0070851"**/>
 <**secondaryRef db="go" dbAc="MI:0448" id="GO:0071375"**/>
 <**secondaryRef db="go" dbAc="MI:0448" id="GO:0086098"**/>
 <**secondaryRef db="go" dbAc="MI:0448" id="GO:0097110"**/>
 <**secondaryRef db="go" dbAc="MI:0448" id="GO:2001237"**/>
 <**secondaryRef db="go" dbAc="MI:0448" id="GO:2001243"**/>
 <**secondaryRef db="reactome" dbAc="MI:0467" id="R-MMU-177929"**/>
 <**secondaryRef db="go" dbAc="MI:0448" id="GO:0005764"**/>
 <**secondaryRef db="go" dbAc="MI:0448" id="GO:0005770"**/>
 <**secondaryRef db="go" dbAc="MI:0448" id="GO:0005901"**/>
 <**secondaryRef db="go" dbAc="MI:0448" id="GO:0007179"**/>
 <**secondaryRef db="go" dbAc="MI:0448" id="GO:0007229"**/>
 <**secondaryRef db="go" dbAc="MI:0448" id="GO:0010632"**/>
 <**secondaryRef db="go" dbAc="MI:0448" id="GO:0016310"**/>
 <**secondaryRef db="go" dbAc="MI:0448" id="GO:0022407"**/>
 <**secondaryRef db="go" dbAc="MI:0448" id="GO:0032463"**/>
 <**secondaryRef db="go" dbAc="MI:0448" id="GO:0043149"**/>
 <**secondaryRef db="go" dbAc="MI:0448" id="GO:0043154"**/>
 <**secondaryRef db="go" dbAc="MI:0448" id="GO:0051897"**/>
 <**secondaryRef db="go" dbAc="MI:0448" id="GO:0051902"**/>
 <**secondaryRef db="go" dbAc="MI:0448" id="GO:0070062"**/>
 <**secondaryRef db="go" dbAc="MI:0448" id="GO:0070555"**/>
 <**secondaryRef db="go" dbAc="MI:0448" id="GO:2000641"**/>
 <**secondaryRef db="go" dbAc="MI:0448" id="GO:2000811"**/>
 <**secondaryRef db="go" dbAc="MI:0448" id="GO:2001286"**/>
 <**secondaryRef db="go" dbAc="MI:0448" id="GO:0071498"**/>
 <**secondaryRef db="reactome" dbAc="MI:0467" id="R-MMU-180292"**/>
 <**secondaryRef db="reactome" dbAc="MI:0467" id="R-MMU-186763"**/>
 <**secondaryRef db="reactome" dbAc="MI:0467" id="R-MMU-191647"**/>
 <**secondaryRef db="reactome" dbAc="MI:0467" id="R-MMU-354192"**/>
 <**secondaryRef db="reactome" dbAc="MI:0467" id="R-MMU-354194"**/>
 <**secondaryRef db="reactome" dbAc="MI:0467" id="R-MMU-372708"**/>
 <**secondaryRef db="reactome" dbAc="MI:0467" id="R-MMU-389356"**/>
 <**secondaryRef db="reactome" dbAc="MI:0467" id="R-MMU-389513"**/>
 <**secondaryRef db="reactome" dbAc="MI:0467" id="R-MMU-3928662"**/>
 <**secondaryRef db="reactome" dbAc="MI:0467" id="R-MMU-3928663"**/>
 <**secondaryRef db="reactome" dbAc="MI:0467" id="R-MMU-3928664"**/>
 <**secondaryRef db="reactome" dbAc="MI:0467" id="R-MMU-3928665"**/>
 <**secondaryRef db="reactome" dbAc="MI:0467" id="R-MMU-418592"**/>
 <**secondaryRef db="reactome" dbAc="MI:0467" id="R-MMU-418885"**/>
 <**secondaryRef db="reactome" dbAc="MI:0467" id="R-MMU-430116"**/>
 <**secondaryRef db="reactome" dbAc="MI:0467" id="R-MMU-437239"**/>
 <**secondaryRef db="reactome" dbAc="MI:0467" id="R-MMU-4420097"**/>
 <**secondaryRef db="reactome" dbAc="MI:0467" id="R-MMU-456926"**/>
 <**secondaryRef db="reactome" dbAc="MI:0467" id="R-MMU-5218921"**/>
 <**secondaryRef db="reactome" dbAc="MI:0467" id="R-MMU-5607764"**/>
 <**secondaryRef db="reactome" dbAc="MI:0467" id="R-MMU-5663220"**/>
 <**secondaryRef db="reactome" dbAc="MI:0467" id="R-MMU-5673000"**/>
 <**secondaryRef db="go" dbAc="MI:0448" id="GO:0010634"**/>
 <**secondaryRef db="go" dbAc="MI:0448" id="GO:2000394"**/>
 <**secondaryRef db="go" dbAc="MI:0448" id="GO:0019900"**/>
 <**secondaryRef db="go" dbAc="MI:0448" id="GO:0071902"**/>
 <**secondaryRef db="go" dbAc="MI:0448" id="GO:0071398"**/>
 <**secondaryRef db="go" dbAc="MI:0448" id="GO:0071456"**/>
 <**secondaryRef db="go" dbAc="MI:0448" id="GO:2000573"**/>
 <**secondaryRef db="go" dbAc="MI:0448" id="GO:0010954"**/>
 <**secondaryRef db="go" dbAc="MI:0448" id="GO:0050731"**/>
 <**secondaryRef db="go" dbAc="MI:0448" id="GO:1900182"**/>
 <**secondaryRef db="go" dbAc="MI:0448" id="GO:0005080"**/>
 <**secondaryRef db="go" dbAc="MI:0448" id="GO:0005158"**/>
 <**secondaryRef db="go" dbAc="MI:0448" id="GO:0008022"**/>
 <**secondaryRef db="go" dbAc="MI:0448" id="GO:0030331"**/>
 <**secondaryRef db="go" dbAc="MI:0448" id="GO:0031625"**/>
 <**secondaryRef db="go" dbAc="MI:0448" id="GO:0045296"**/>
 <**secondaryRef db="go" dbAc="MI:0448" id="GO:0071253"**/>
 <**secondaryRef db="go" dbAc="MI:0448" id="GO:0002102"**/>
 <**secondaryRef db="go" dbAc="MI:0448" id="GO:0051057"**/>
 <**secondaryRef db="reactome" dbAc="MI:0467" id="R-MMU-8853659"**/>
 <**secondaryRef db="reactome" dbAc="MI:0467" id="R-MMU-8874081"**/>
 </**xref**>
 <**interactorType**>
 <**names**>
 <**shortLabel**>protein</**shortLabel**>
 <**fullName**>protein</**fullName**>
 </**names**>
 <**xref**>
 <**primaryRef db="psi-mi" dbAc="MI:0488" id="MI:0326" refType="identity" refTypeAc="MI:0356"**/>
 <**secondaryRef db="intact" dbAc="MI:0469" id="EBI-619654" refType="identity" refTypeAc="MI:0356"**/>
 <**secondaryRef db="pubmed" dbAc="MI:0446" id="14755292" refType="primary-reference" refTypeAc="MI:0358"**/>
 <**secondaryRef db="so" dbAc="MI:0601" id="SO:0000358" refType="see-also" refTypeAc="MI:0361"**/>
 </**xref**>
 </**interactorType**>
 <**organism ncbiTaxId="10090"**>
 <**names**>
 <**shortLabel**>mouse</**shortLabel**>
 <**fullName**>Mus musculus</**fullName**>
 <**alias type="synonym" typeAc="MI:1041"**>Mouse</**alias**>
 </**names**>
 </**organism**>
 <**sequence**>
 MGSNKSKPKDASQRRRSLEPSENVHGAGGAFPASQTPSKPASADGHRGPSAAFVPPAAEPKLFGGFNSSDTVTSPQRAGPLAGGVTTFVALYDYESRTETDLSFKKGERLQIVNNTRKVDVREGDWWLAHSLSTGQTGYIPSNYVAPSDSIQAEEWYFGKITRRESERLLLNAENPRGTFLVRESETTKGAYCLSVSDFDNAKGLNVKHYKIRKLDSGGFYITSRTQFNSLQQLVAYYSKHADGLCHRLTTVCPTSKPQTQGLAKDAWEIPRESLRLEVKLGQGCFGEVWMGTWNGTTRVAIKTLKPGTMSPEAFLQEAQVMKKLRHEKLVQLYAVVSEEPIYIVTEYMNKGSLLDFLKGETGKYLRLPQLVDMSAQIASGMAYVERMNYVHRDLRAANILVGENLVCKVADFGLARLIEDNEYTARQGAKFPIKWTAPEAALYGRFTIKSDVWSFGILLTELTTKGRVPYPGMVNREVLDQVERGYRMPCPPECPESLHDLMCQCWRKEPEERPTFEYLQAFLEDYFTSTEPQYQPGENL
 </**sequence**>
 <**attributeList**>
 <**attribute name="crc64"**>0534AF027783BCCF</**attribute**>
 </**attributeList**>
 </**interactor**>
 <**interactor id="3"**>
 <**names**>
 <**shortLabel**>hxk1_human</**shortLabel**>
 <**fullName**>Hexokinase-1</**fullName**>
 <**alias type="gene name synonym" typeAc="MI:0302"**>Hexokinase type I</**alias**>
 <**alias type="gene name synonym" typeAc="MI:0302"**>Brain form hexokinase</**alias**>
 <**alias type="gene name" typeAc="MI:0301"**>HK1</**alias**>
 </**names**>
 <**xref**>
 <**primaryRef db="uniprotkb" dbAc="MI:0486" id="P19367" version="SP_103" refType="identity" refTypeAc="MI:0356"**/>
 <**secondaryRef db="uniprotkb" dbAc="MI:0486" id="Q5VTC3" version="SP_103" refType="secondary-ac" refTypeAc="MI:0360"**/>
 <**secondaryRef db="uniprotkb" dbAc="MI:0486" id="Q96HC8" version="SP_103" refType="secondary-ac" refTypeAc="MI:0360"**/>
 <**secondaryRef db="uniprotkb" dbAc="MI:0486" id="Q9NNZ4" version="SP_103" refType="secondary-ac" refTypeAc="MI:0360"**/>
 <**secondaryRef db="uniprotkb" dbAc="MI:0486" id="O43443" version="SP_103" refType="secondary-ac" refTypeAc="MI:0360"**/>
 <**secondaryRef db="uniprotkb" dbAc="MI:0486" id="O43444" version="SP_103" refType="secondary-ac" refTypeAc="MI:0360"**/>
 <**secondaryRef db="uniprotkb" dbAc="MI:0486" id="O75574" version="SP_103" refType="secondary-ac" refTypeAc="MI:0360"**/>
 <**secondaryRef db="uniprotkb" dbAc="MI:0486" id="Q9NNZ5" version="SP_103" refType="secondary-ac" refTypeAc="MI:0360"**/>
 <**secondaryRef db="uniprotkb" dbAc="MI:0486" id="E9PCK0" version="SP_150" refType="secondary-ac" refTypeAc="MI:0360"**/>
 <**secondaryRef db="intact" dbAc="MI:0469" id="EBI-713162" refType="identity" refTypeAc="MI:0356"**/>
 <**secondaryRef db="rcsb pdb" dbAc="MI:0460" id="4F9O"**/>
 <**secondaryRef db="refseq" dbAc="MI:0481" id="NP_000179.2"**/>
 <**secondaryRef db="refseq" dbAc="MI:0481" id="NP_277031.1"**/>
 <**secondaryRef db="refseq" dbAc="MI:0481" id="NP_277032.1"**/>
 <**secondaryRef db="refseq" dbAc="MI:0481" id="NP_277033.1"**/>
 <**secondaryRef db="refseq" dbAc="MI:0481" id="NP_277035.2"**/>
 <**secondaryRef db="interpro" dbAc="MI:0449" id="IPR001312"**/>
 <**secondaryRef db="interpro" dbAc="MI:0449" id="IPR022673"**/>
 <**secondaryRef db="interpro" dbAc="MI:0449" id="IPR019807"**/>
 <**secondaryRef db="interpro" dbAc="MI:0449" id="IPR022672"**/>
 <**secondaryRef db="go" dbAc="MI:0448" id="GO:0005829"**/>
 <**secondaryRef db="go" dbAc="MI:0448" id="GO:0005741"**/>
 <**secondaryRef db="go" dbAc="MI:0448" id="GO:0005524"**/>
 <**secondaryRef db="go" dbAc="MI:0448" id="GO:0015758"**/>
 <**secondaryRef db="go" dbAc="MI:0448" id="GO:0006096"**/>
 <**secondaryRef db="rcsb pdb" dbAc="MI:0460" id="1CZA"**/>
 <**secondaryRef db="rcsb pdb" dbAc="MI:0460" id="1DGK"**/>
 <**secondaryRef db="rcsb pdb" dbAc="MI:0460" id="1HKB"**/>
 <**secondaryRef db="rcsb pdb" dbAc="MI:0460" id="1HKC"**/>
 <**secondaryRef db="rcsb pdb" dbAc="MI:0460" id="1QHA"**/>
 <**secondaryRef db="go" dbAc="MI:0448" id="GO:0001678"**/>
 <**secondaryRef db="go" dbAc="MI:0448" id="GO:0004340"**/>
 <**secondaryRef db="go" dbAc="MI:0448" id="GO:0008865"**/>
 <**secondaryRef db="go" dbAc="MI:0448" id="GO:0019158"**/>
 <**secondaryRef db="ensembl" dbAc="MI:0476" id="ENST00000298649"**/>
 <**secondaryRef db="ensembl" dbAc="MI:0476" id="ENST00000359426"**/>
 <**secondaryRef db="ensembl" dbAc="MI:0476" id="ENST00000360289"**/>
 <**secondaryRef db="go" dbAc="MI:0448" id="GO:0061621"**/>
 <**secondaryRef db="go" dbAc="MI:0448" id="GO:0005739"**/>
 <**secondaryRef db="rcsb pdb" dbAc="MI:0460" id="4FOE"**/>
 <**secondaryRef db="rcsb pdb" dbAc="MI:0460" id="4FOI"**/>
 <**secondaryRef db="rcsb pdb" dbAc="MI:0460" id="4FPA"**/>
 <**secondaryRef db="rcsb pdb" dbAc="MI:0460" id="4FPB"**/>
 <**secondaryRef db="go" dbAc="MI:0448" id="GO:0004396"**/>
 <**secondaryRef db="refseq" dbAc="MI:0481" id="NP_001309293.1"**/>
 <**secondaryRef db="ensembl" dbAc="MI:0476" id="ENSG00000156515"**/>
 <**secondaryRef db="ensembl" dbAc="MI:0476" id="ENSP00000298649"**/>
 <**secondaryRef db="ensembl" dbAc="MI:0476" id="ENSP00000352398"**/>
 <**secondaryRef db="ensembl" dbAc="MI:0476" id="ENSP00000353433"**/>
 <**secondaryRef db="refseq" dbAc="MI:0481" id="XP_011538034.1"**/>
 <**secondaryRef db="go" dbAc="MI:0448" id="GO:0005536"**/>
 <**secondaryRef db="reactome" dbAc="MI:0467" id="R-HSA-70153"**/>
 <**secondaryRef db="reactome" dbAc="MI:0467" id="R-HSA-70171"**/>
 <**secondaryRef db="ensembl" dbAc="MI:0476" id="ENSP00000402103"**/>
 <**secondaryRef db="ensembl" dbAc="MI:0476" id="ENST00000448642"**/>
 <**secondaryRef db="go" dbAc="MI:0448" id="GO:0072655"**/>
 <**secondaryRef db="go" dbAc="MI:0448" id="GO:0072656"**/>
 <**secondaryRef db="go" dbAc="MI:0448" id="GO:0045121"**/>
 <**secondaryRef db="go" dbAc="MI:0448" id="GO:0097228"**/>
 </**xref**>
 <**interactorType**>
 <**names**>
 <**shortLabel**>protein</**shortLabel**>
 <**fullName**>protein</**fullName**>
 </**names**>
 <**xref**>
 <**primaryRef db="psi-mi" dbAc="MI:0488" id="MI:0326" refType="identity" refTypeAc="MI:0356"**/>
 <**secondaryRef db="intact" dbAc="MI:0469" id="EBI-619654" refType="identity" refTypeAc="MI:0356"**/>
 <**secondaryRef db="pubmed" dbAc="MI:0446" id="14755292" refType="primary-reference" refTypeAc="MI:0358"**/>
 <**secondaryRef db="so" dbAc="MI:0601" id="SO:0000358" refType="see-also" refTypeAc="MI:0361"**/>
 </**xref**>
 </**interactorType**>
 <**organism ncbiTaxId="9606"**>
 <**names**>
 <**shortLabel**>human</**shortLabel**>
 <**fullName**>Homo sapiens</**fullName**>
 <**alias type="synonym" typeAc="MI:1041"**>Human</**alias**>
 </**names**>
 </**organism**>
 <**sequence**>
 MIAAQLLAYYFTELKDDQVKKIDKYLYAMRLSDETLIDIMTRFRKEMKNGLSRDFNPTATVKMLPTFVRSIPDGSEKGDFIALDLGGSSFRILRVQVNHEKNQNVHMESEVYDTPENIVHGSGSQLFDHVAECLGDFMEKRKIKDKKLPVGFTFSFPCQQSKIDEAILITWTKRFKASGVEGADVVKLLNKAIKKRGDYDANIVAVVNDTVGTMMTCGYDDQHCEVGLIIGTGTNACYMEELRHIDLVEGDEGRMCINTEWGAFGDDGSLEDIRTEFDREIDRGSLNPGKQLFEKMVSGMYLGELVRLILVKMAKEGLLFEGRITPELLTRGKFNTSDVSAIEKNKEGLHNAKEILTRLGVEPSDDDCVSVQHVCTIVSFRSANLVAATLGAILNRLRDNKGTPRLRTTVGVDGSLYKTHPQYSRRFHKTLRRLVPDSDVRFLLSESGSGKGAAMVTAVAYRLAEQHRQIEETLAHFHLTKDMLLEVKKRMRAEMELGLRKQTHNNAVVKMLPSFVRRTPDGTENGDFLALDLGGTNFRVLLVKIRSGKKRTVEMHNKIYAIPIEIMQGTGEELFDHIVSCISDFLDYMGIKGPRMPLGFTFSFPCQQTSLDAGILITWTKGFKATDCVGHDVVTLLRDAIKRREEFDLDVVAVVNDTVGTMMTCAYEEPTCEVGLIVGTGSNACYMEEMKNVEMVEGDQGQMCINMEWGAFGDNGCLDDIRTHYDRLVDEYSLNAGKQRYEKMISGMYLGEIVRNILIDFTKKGFLFRGQISETLKTRGIFETKFLSQIESDRLALLQVRAILQQLGLNSTCDDSILVKTVCGVVSRRAAQLCGAGMAAVVDKIRENRGLDRLNVTVGVDGTLYKLHPHFSRIMHQTVKELSPKCNVSFLLSEDGSGKGAALITAVGVRLRTEASS
 </**sequence**>
 <**attributeList**>
 <**attribute name="crc64"**>F29A6837531C0594</**attribute**>
 </**attributeList**>
 </**interactor**>
 </**interactorList**>
 <**interactionList**>
 <**interaction id="4"**>
 <**names**>
 <**shortLabel**>src-hk1-1</**shortLabel**>
 </**names**>
 <**xref**>
 <**primaryRef db="intact" dbAc="MI:0469" id="EBI-14988396" refType="identity" refTypeAc="MI:0356"**/>
 </**xref**>
 <**experimentList**>
 <**experimentRef**>1</**experimentRef**>
 </**experimentList**>
 <**participantList**>
 <**participant id="5"**>
 <**interactorRef**>2</**interactorRef**>
 <**biologicalRole**>
 <**names**>
 <**shortLabel**>unspecified role</**shortLabel**>
 <**fullName**>unspecified role</**fullName**>
 </**names**>
 <**xref**>
 <**primaryRef db="psi-mi" dbAc="MI:0488" id="MI:0499" refType="identity" refTypeAc="MI:0356"**/>
 <**secondaryRef db="intact" dbAc="MI:0469" id="EBI-77781" refType="identity" refTypeAc="MI:0356"**/>
 <**secondaryRef db="pubmed" dbAc="MI:0446" id="14755292" refType="primary-reference" refTypeAc="MI:0358"**/>
 </**xref**>
 </**biologicalRole**>
 <**experimentalRoleList**>
 <**experimentalRole**>
 <**names**>
 <**shortLabel**>prey</**shortLabel**>
 <**fullName**>prey</**fullName**>
 </**names**>
 <**xref**>
 <**primaryRef db="psi-mi" dbAc="MI:0488" id="MI:0498" refType="identity" refTypeAc="MI:0356"**/>
 <**secondaryRef db="intact" dbAc="MI:0469" id="EBI-58" refType="identity" refTypeAc="MI:0356"**/>
 <**secondaryRef db="pubmed" dbAc="MI:0446" id="14755292" refType="primary-reference" refTypeAc="MI:0358"**/>
 </**xref**>
 </**experimentalRole**>
 </**experimentalRoleList**>
 <**featureList**>
 <**feature id="6"**>
 <**names**>
 <**shortLabel**>region</**shortLabel**>
 </**names**>
 <**xref**>
 <**primaryRef db="intact" dbAc="MI:0469" id="EBI-14988400" refType="identity" refTypeAc="MI:0356"**/>
 </**xref**>
 <**featureType**>
 <**names**>
 <**shortLabel**>ha tag</**shortLabel**>
 <**fullName**>ha tag</**fullName**>
 <**alias type="go synonym" typeAc="MI:0303"**>YPYDVPDYA epitope tag</**alias**>
 </**names**>
 <**xref**>
 <**primaryRef db="psi-mi" dbAc="MI:0488" id="MI:0520" refType="identity" refTypeAc="MI:0356"**/>
 <**secondaryRef db="intact" dbAc="MI:0469" id="EBI-456513" refType="identity" refTypeAc="MI:0356"**/>
 <**secondaryRef db="pubmed" dbAc="MI:0446" id="14755292" refType="primary-reference" refTypeAc="MI:0358"**/>
 </**xref**>
 </**featureType**>
 <**featureRangeList**>
 <**featureRange**>
 <**startStatus**>
 <**names**>
 <**shortLabel**>undetermined</**shortLabel**>
 <**fullName**>undetermined sequence position</**fullName**>
 </**names**>
 <**xref**>
 <**primaryRef db="psi-mi" dbAc="MI:0488" id="MI:0339" refType="identity" refTypeAc="MI:0356"**/>
 <**secondaryRef db="intact" dbAc="MI:0469" id="EBI-448295" refType="identity" refTypeAc="MI:0356"**/>
 <**secondaryRef db="pubmed" dbAc="MI:0446" id="14755292" refType="primary-reference" refTypeAc="MI:0358"**/>
 </**xref**>
 </**startStatus**>
 <**endStatus**>
 <**names**>
 <**shortLabel**>undetermined</**shortLabel**>
 <**fullName**>undetermined sequence position</**fullName**>
 </**names**>
 <**xref**>
 <**primaryRef db="psi-mi" dbAc="MI:0488" id="MI:0339" refType="identity" refTypeAc="MI:0356"**/>
 <**secondaryRef db="intact" dbAc="MI:0469" id="EBI-448295" refType="identity" refTypeAc="MI:0356"**/>
 <**secondaryRef db="pubmed" dbAc="MI:0446" id="14755292" refType="primary-reference" refTypeAc="MI:0358"**/>
 </**xref**>
 </**endStatus**>
 </**featureRange**>
 </**featureRangeList**>
 </**feature**>
 </**featureList**>
 </**participant**>
 <**participant id="7"**>
 <**interactorRef**>3</**interactorRef**>
 <**biologicalRole**>
 <**names**>
 <**shortLabel**>unspecified role</**shortLabel**>
 <**fullName**>unspecified role</**fullName**>
 </**names**>
 <**xref**>
 <**primaryRef db="psi-mi" dbAc="MI:0488" id="MI:0499" refType="identity" refTypeAc="MI:0356"**/>
 <**secondaryRef db="intact" dbAc="MI:0469" id="EBI-77781" refType="identity" refTypeAc="MI:0356"**/>
 <**secondaryRef db="pubmed" dbAc="MI:0446" id="14755292" refType="primary-reference" refTypeAc="MI:0358"**/>
 </**xref**>
 </**biologicalRole**>
 <**experimentalRoleList**>
 <**experimentalRole**>
 <**names**>
 <**shortLabel**>bait</**shortLabel**>
 <**fullName**>bait</**fullName**>
 </**names**>
 <**xref**>
 <**primaryRef db="psi-mi" dbAc="MI:0488" id="MI:0496" refType="identity" refTypeAc="MI:0356"**/>
 <**secondaryRef db="intact" dbAc="MI:0469" id="EBI-49" refType="identity" refTypeAc="MI:0356"**/>
 <**secondaryRef db="pubmed" dbAc="MI:0446" id="14755292" refType="primary-reference" refTypeAc="MI:0358"**/>
 </**xref**>
 </**experimentalRole**>
 </**experimentalRoleList**>
 <**featureList**>
 <**feature id="8"**>
 <**names**>
 <**shortLabel**>region</**shortLabel**>
 </**names**>
 <**xref**>
 <**primaryRef db="intact" dbAc="MI:0469" id="EBI-14988403" refType="identity" refTypeAc="MI:0356"**/>
 </**xref**>
 <**featureType**>
 <**names**>
 <**shortLabel**>flag tag</**shortLabel**>
 <**fullName**>flag tag</**fullName**>
 <**alias type="go synonym" typeAc="MI:0303"**>FLAG-tagged</**alias**>
 <**alias type="go synonym" typeAc="MI:0303"**>FLAG</**alias**>
 <**alias type="go synonym" typeAc="MI:0303"**>DYKDDDDKV epitope tag</**alias**>
 </**names**>
 <**xref**>
 <**primaryRef db="psi-mi" dbAc="MI:0488" id="MI:0518" refType="identity" refTypeAc="MI:0356"**/>
 <**secondaryRef db="intact" dbAc="MI:0469" id="EBI-456503" refType="identity" refTypeAc="MI:0356"**/>
 <**secondaryRef db="pubmed" dbAc="MI:0446" id="14755292" refType="primary-reference" refTypeAc="MI:0358"**/>
 </**xref**>
 </**featureType**>
 <**featureRangeList**>
 <**featureRange**>
 <**startStatus**>
 <**names**>
 <**shortLabel**>undetermined</**shortLabel**>
 <**fullName**>undetermined sequence position</**fullName**>
 </**names**>
 <**xref**>
 <**primaryRef db="psi-mi" dbAc="MI:0488" id="MI:0339" refType="identity" refTypeAc="MI:0356"**/>
 <**secondaryRef db="intact" dbAc="MI:0469" id="EBI-448295" refType="identity" refTypeAc="MI:0356"**/>
 <**secondaryRef db="pubmed" dbAc="MI:0446" id="14755292" refType="primary-reference" refTypeAc="MI:0358"**/>
 </**xref**>
 </**startStatus**>
 <**endStatus**>
 <**names**>
 <**shortLabel**>undetermined</**shortLabel**>
 <**fullName**>undetermined sequence position</**fullName**>
 </**names**>
 <**xref**>
 <**primaryRef db="psi-mi" dbAc="MI:0488" id="MI:0339" refType="identity" refTypeAc="MI:0356"**/>
 <**secondaryRef db="intact" dbAc="MI:0469" id="EBI-448295" refType="identity" refTypeAc="MI:0356"**/>
 <**secondaryRef db="pubmed" dbAc="MI:0446" id="14755292" refType="primary-reference" refTypeAc="MI:0358"**/>
 </**xref**>
 </**endStatus**>
 </**featureRange**>
 </**featureRangeList**>
 </**feature**>
 <**feature id="9"**>
 <**names**>
 <**shortLabel**>region</**shortLabel**>
 </**names**>
 <**xref**>
 <**primaryRef db="intact" dbAc="MI:0469" id="EBI-14988456" refType="identity" refTypeAc="MI:0356"**/>
 </**xref**>
 <**featureType**>
 <**names**>
 <**shortLabel**>ophosres</**shortLabel**>
 <**fullName**>N-phosphorylated residue</**fullName**>
 </**names**>
 <**xref**>
 <**primaryRef db="psi-mod" dbAc="MI:0897" id="MOD:01456" refType="identity" refTypeAc="MI:0356"**/>
 <**secondaryRef db="intact" dbAc="MI:0469" id="EBI-5527781" refType="identity" refTypeAc="MI:0356"**/>
 <**secondaryRef db="pubmed" dbAc="MI:0446" id="18688235" refType="primary-reference" refTypeAc="MI:0358"**/>
 </**xref**>
 </**featureType**>
 <**featureRangeList**>
 <**featureRange**>
 <**startStatus**>
 <**names**>
 <**shortLabel**>undetermined</**shortLabel**>
 <**fullName**>undetermined sequence position</**fullName**>
 </**names**>
 <**xref**>
 <**primaryRef db="psi-mi" dbAc="MI:0488" id="MI:0339" refType="identity" refTypeAc="MI:0356"**/>
 <**secondaryRef db="intact" dbAc="MI:0469" id="EBI-448295" refType="identity" refTypeAc="MI:0356"**/>
 <**secondaryRef db="pubmed" dbAc="MI:0446" id="14755292" refType="primary-reference" refTypeAc="MI:0358"**/>
 </**xref**>
 </**startStatus**>
 <**endStatus**>
 <**names**>
 <**shortLabel**>undetermined</**shortLabel**>
 <**fullName**>undetermined sequence position</**fullName**>
 </**names**>
 <**xref**>
 <**primaryRef db="psi-mi" dbAc="MI:0488" id="MI:0339" refType="identity" refTypeAc="MI:0356"**/>
 <**secondaryRef db="intact" dbAc="MI:0469" id="EBI-448295" refType="identity" refTypeAc="MI:0356"**/>
 <**secondaryRef db="pubmed" dbAc="MI:0446" id="14755292" refType="primary-reference" refTypeAc="MI:0358"**/>
 </**xref**>
 </**endStatus**>
 </**featureRange**>
 </**featureRangeList**>
 <**featureRole**>
 <**names**>
 <**shortLabel**>observed ptm</**shortLabel**>
 <**fullName**>observed ptm</**fullName**>
 </**names**>
 <**xref**>
 <**primaryRef db="psi-mi" dbAc="MI:0488" id="MI:0925" refType="identity" refTypeAc="MI:0356"**/>
 <**secondaryRef db="intact" dbAc="MI:0469" id="EBI-1813092" refType="identity" refTypeAc="MI:0356"**/>
 <**secondaryRef db="pubmed" dbAc="MI:0446" id="14755292" refType="primary-reference" refTypeAc="MI:0358"**/>
 </**xref**>
 </**featureRole**>
 </**feature**>
 <**feature id="10"**>
 <**names**>
 <**shortLabel**>tyr27phe</**shortLabel**>
 </**names**>
 <**xref**>
 <**primaryRef db="intact" dbAc="MI:0469" id="EBI-14988465" refType="identity" refTypeAc="MI:0356"**/>
 </**xref**>
 <**featureType**>
 <**names**>
 <**shortLabel**>mutation with no effect</**shortLabel**>
 <**fullName**>mutation having no effect on an interaction</**fullName**>
 </**names**>
 <**xref**>
 <**primaryRef db="psi-mi" dbAc="MI:0488" id="MI:2226" refType="identity" refTypeAc="MI:0356"**/>
 <**secondaryRef db="intact" dbAc="MI:0469" id="IA:3038" refType="identity" refTypeAc="MI:0356"**/>
 <**secondaryRef db="intact" dbAc="MI:0469" id="EBI-11688027" refType="identity" refTypeAc="MI:0356"**/>
 </**xref**>
 </**featureType**>
 <**featureRangeList**>
 <**featureRange**>
 <**startStatus**>
 <**names**>
 <**shortLabel**>certain</**shortLabel**>
 <**fullName**>certain sequence position</**fullName**>
 <**alias type="synonym" typeAc="MI:1041"**>certain</**alias**>
 </**names**>
 <**xref**>
 <**primaryRef db="psi-mi" dbAc="MI:0488" id="MI:0335" refType="identity" refTypeAc="MI:0356"**/>
 <**secondaryRef db="intact" dbAc="MI:0469" id="EBI-540564" refType="identity" refTypeAc="MI:0356"**/>
 <**secondaryRef db="pubmed" dbAc="MI:0446" id="14755292" refType="primary-reference" refTypeAc="MI:0358"**/>
 </**xref**>
 </**startStatus**>
 <**begin position="27"**/>
 <**endStatus**>
 <**names**>
 <**shortLabel**>certain</**shortLabel**>
 <**fullName**>certain sequence position</**fullName**>
 <**alias type="synonym" typeAc="MI:1041"**>certain</**alias**>
 </**names**>
 <**xref**>
 <**primaryRef db="psi-mi" dbAc="MI:0488" id="MI:0335" refType="identity" refTypeAc="MI:0356"**/>
 <**secondaryRef db="intact" dbAc="MI:0469" id="EBI-540564" refType="identity" refTypeAc="MI:0356"**/>
 <**secondaryRef db="pubmed" dbAc="MI:0446" id="14755292" refType="primary-reference" refTypeAc="MI:0358"**/>
 </**xref**>
 </**endStatus**>
 <**end position="27"**/>
 <**resultingSequence**>
 <**originalSequence**>Y</**originalSequence**>
 <**newSequence**>F</**newSequence**>
 </**resultingSequence**>
 </**featureRange**>
 </**featureRangeList**>
 </**feature**>
 <**feature id="11"**>
 <**names**>
 <**shortLabel**>tyr417phe</**shortLabel**>
 </**names**>
 <**xref**>
 <**primaryRef db="intact" dbAc="MI:0469" id="EBI-14988467" refType="identity" refTypeAc="MI:0356"**/>
 </**xref**>
 <**featureType**>
 <**names**>
 <**shortLabel**>mutation with no effect</**shortLabel**>
 <**fullName**>mutation having no effect on an interaction</**fullName**>
 </**names**>
 <**xref**>
 <**primaryRef db="psi-mi" dbAc="MI:0488" id="MI:2226" refType="identity" refTypeAc="MI:0356"**/>
 <**secondaryRef db="intact" dbAc="MI:0469" id="IA:3038" refType="identity" refTypeAc="MI:0356"**/>
 <**secondaryRef db="intact" dbAc="MI:0469" id="EBI-11688027" refType="identity" refTypeAc="MI:0356"**/>
 </**xref**>
 </**featureType**>
 <**featureRangeList**>
 <**featureRange**>
 <**startStatus**>
 <**names**>
 <**shortLabel**>certain</**shortLabel**>
 <**fullName**>certain sequence position</**fullName**>
 <**alias type="synonym" typeAc="MI:1041"**>certain</**alias**>
 </**names**>
 <**xref**>
 <**primaryRef db="psi-mi" dbAc="MI:0488" id="MI:0335" refType="identity" refTypeAc="MI:0356"**/>
 <**secondaryRef db="intact" dbAc="MI:0469" id="EBI-540564" refType="identity" refTypeAc="MI:0356"**/>
 <**secondaryRef db="pubmed" dbAc="MI:0446" id="14755292" refType="primary-reference" refTypeAc="MI:0358"**/>
 </**xref**>
 </**startStatus**>
 <**begin position="417"**/>
 <**endStatus**>
 <**names**>
 <**shortLabel**>certain</**shortLabel**>
 <**fullName**>certain sequence position</**fullName**>
 <**alias type="synonym" typeAc="MI:1041"**>certain</**alias**>
 </**names**>
 <**xref**>
 <**primaryRef db="psi-mi" dbAc="MI:0488" id="MI:0335" refType="identity" refTypeAc="MI:0356"**/>
 <**secondaryRef db="intact" dbAc="MI:0469" id="EBI-540564" refType="identity" refTypeAc="MI:0356"**/>
 <**secondaryRef db="pubmed" dbAc="MI:0446" id="14755292" refType="primary-reference" refTypeAc="MI:0358"**/>
 </**xref**>
 </**endStatus**>
 <**end position="417"**/>
 <**resultingSequence**>
 <**originalSequence**>Y</**originalSequence**>
 <**newSequence**>F</**newSequence**>
 </**resultingSequence**>
 </**featureRange**>
 </**featureRangeList**>
 </**feature**>
 <**feature id="12"**>
 <**names**>
 <**shortLabel**>tyr732phe</**shortLabel**>
 </**names**>
 <**xref**>
 <**primaryRef db="intact" dbAc="MI:0469" id="EBI-14988469" refType="identity" refTypeAc="MI:0356"**/>
 </**xref**>
 <**featureType**>
 <**names**>
 <**shortLabel**>mutation disrupting strength</**shortLabel**>
 <**fullName**>mutation disrupting interaction strength</**fullName**>
 </**names**>
 <**xref**>
 <**primaryRef db="psi-mi" dbAc="MI:0488" id="MI:1128" refType="identity" refTypeAc="MI:0356"**/>
 <**secondaryRef db="intact" dbAc="MI:0469" id="EBI-5528001" refType="identity" refTypeAc="MI:0356"**/>
 <**secondaryRef db="pubmed" dbAc="MI:0446" id="14755292" refType="primary-reference" refTypeAc="MI:0358"**/>
 </**xref**>
 </**featureType**>
 <**featureDetectionMethod**>
 <**names**>
 <**shortLabel**>western blot</**shortLabel**>
 <**fullName**>western blot</**fullName**>
 <**alias type="go synonym" typeAc="MI:0303"**>Immuno blot</**alias**>
 </**names**>
 <**xref**>
 <**primaryRef db="psi-mi" dbAc="MI:0488" id="MI:0113" refType="identity" refTypeAc="MI:0356"**/>
 <**secondaryRef db="intact" dbAc="MI:0469" id="EBI-456832" refType="identity" refTypeAc="MI:0356"**/>
 <**secondaryRef db="pubmed" dbAc="MI:0446" id="14755292" refType="primary-reference" refTypeAc="MI:0358"**/>
 </**xref**>
 </**featureDetectionMethod**>
 <**featureRangeList**>
 <**featureRange**>
 <**startStatus**>
 <**names**>
 <**shortLabel**>certain</**shortLabel**>
 <**fullName**>certain sequence position</**fullName**>
 <**alias type="synonym" typeAc="MI:1041"**>certain</**alias**>
 </**names**>
 <**xref**>
 <**primaryRef db="psi-mi" dbAc="MI:0488" id="MI:0335" refType="identity" refTypeAc="MI:0356"**/>
 <**secondaryRef db="intact" dbAc="MI:0469" id="EBI-540564" refType="identity" refTypeAc="MI:0356"**/>
 <**secondaryRef db="pubmed" dbAc="MI:0446" id="14755292" refType="primary-reference" refTypeAc="MI:0358"**/>
 </**xref**>
 </**startStatus**>
 <**begin position="732"**/>
 <**endStatus**>
 <**names**>
 <**shortLabel**>certain</**shortLabel**>
 <**fullName**>certain sequence position</**fullName**>
 <**alias type="synonym" typeAc="MI:1041"**>certain</**alias**>
 </**names**>
 <**xref**>
 <**primaryRef db="psi-mi" dbAc="MI:0488" id="MI:0335" refType="identity" refTypeAc="MI:0356"**/>
 <**secondaryRef db="intact" dbAc="MI:0469" id="EBI-540564" refType="identity" refTypeAc="MI:0356"**/>
 <**secondaryRef db="pubmed" dbAc="MI:0446" id="14755292" refType="primary-reference" refTypeAc="MI:0358"**/>
 </**xref**>
 </**endStatus**>
 <**end position="732"**/>
 <**resultingSequence**>
 <**originalSequence**>Y</**originalSequence**>
 <**newSequence**>F</**newSequence**>
 </**resultingSequence**>
 </**featureRange**>
 </**featureRangeList**>
 </**feature**>
 <**feature id="13"**>
 <**names**>
 <**shortLabel**>ser-732</**shortLabel**>
 </**names**>
 <**xref**>
 <**primaryRef db="intact" dbAc="MI:0469" id="EBI-14988471" refType="identity" refTypeAc="MI:0356"**/>
 </**xref**>
 <**featureType**>
 <**names**>
 <**shortLabel**>optyr</**shortLabel**>
 <**fullName**>O4'-phospho-L-tyrosine</**fullName**>
 <**alias type="synonym" typeAc="MI:1041"**>(2S)-2-amino-3-(4-phosphonooxyphenyl)propanoic acid</**alias**>
 <**alias type="synonym" typeAc="MI:1041"**>2-azanyl-3-(4-phosphonooxyphenyl)propanoic acid</**alias**>
 <**alias type="synonym" typeAc="MI:1041"**>MOD_RES Phosphotyrosine</**alias**>
 <**alias type="synonym" typeAc="MI:1041"**>O4-phosphotyrosine</**alias**>
 <**alias type="synonym" typeAc="MI:1041"**>tyrosine phosphate</**alias**>
 <**alias type="synonym" typeAc="MI:1041"**>2-amino-3-(4-hydroxyphenyl)propanoic acid 4'-phosphate</**alias**>
 <**alias type="synonym" typeAc="MI:1041"**>O4'-phospho-L-tyrosine</**alias**>
 <**alias type="synonym" typeAc="MI:1041"**>O4'-phosphorylated L-tyrosine</**alias**>
 </**names**>
 <**xref**>
 <**primaryRef db="psi-mod" dbAc="MI:0897" id="MOD:00048" refType="identity" refTypeAc="MI:0356"**/>
 <**secondaryRef db="intact" dbAc="MI:0469" id="EBI-456748" refType="identity" refTypeAc="MI:0356"**/>
 <**secondaryRef db="pubmed" dbAc="MI:0446" id="10226369" refType="primary-reference" refTypeAc="MI:0358"**/>
 <**secondaryRef db="pubmed" dbAc="MI:0446" id="1725475" refType="primary-reference" refTypeAc="MI:0358"**/>
 <**secondaryRef db="resid" dbAc="MI:0248" id="AA0039" refType="see-also" refTypeAc="MI:0361"**/>
 </**xref**>
 </**featureType**>
 <**featureRangeList**>
 <**featureRange**>
 <**startStatus**>
 <**names**>
 <**shortLabel**>certain</**shortLabel**>
 <**fullName**>certain sequence position</**fullName**>
 <**alias type="synonym" typeAc="MI:1041"**>certain</**alias**>
 </**names**>
 <**xref**>
 <**primaryRef db="psi-mi" dbAc="MI:0488" id="MI:0335" refType="identity" refTypeAc="MI:0356"**/>
 <**secondaryRef db="intact" dbAc="MI:0469" id="EBI-540564" refType="identity" refTypeAc="MI:0356"**/>
 <**secondaryRef db="pubmed" dbAc="MI:0446" id="14755292" refType="primary-reference" refTypeAc="MI:0358"**/>
 </**xref**>
 </**startStatus**>
 <**begin position="732"**/>
 <**endStatus**>
 <**names**>
 <**shortLabel**>certain</**shortLabel**>
 <**fullName**>certain sequence position</**fullName**>
 <**alias type="synonym" typeAc="MI:1041"**>certain</**alias**>
 </**names**>
 <**xref**>
 <**primaryRef db="psi-mi" dbAc="MI:0488" id="MI:0335" refType="identity" refTypeAc="MI:0356"**/>
 <**secondaryRef db="intact" dbAc="MI:0469" id="EBI-540564" refType="identity" refTypeAc="MI:0356"**/>
 <**secondaryRef db="pubmed" dbAc="MI:0446" id="14755292" refType="primary-reference" refTypeAc="MI:0358"**/>
 </**xref**>
 </**endStatus**>
 <**end position="732"**/>
 <**resultingSequence**>
 <**originalSequence**>Y</**originalSequence**>
 <**newSequence**>F</**newSequence**>
 </**resultingSequence**>
 </**featureRange**>
 </**featureRangeList**>
 <**featureRole**>
 <**names**>
 <**shortLabel**>prerequisite-ptm</**shortLabel**>
 <**fullName**>prerequisite-ptm</**fullName**>
 </**names**>
 <**xref**>
 <**primaryRef db="psi-mi" dbAc="MI:0488" id="MI:0638" refType="identity" refTypeAc="MI:0356"**/>
 <**secondaryRef db="intact" dbAc="MI:0469" id="EBI-872" refType="identity" refTypeAc="MI:0356"**/>
 <**secondaryRef db="pubmed" dbAc="MI:0446" id="14755292" refType="primary-reference" refTypeAc="MI:0358"**/>
 </**xref**>
 </**featureRole**>
 </**feature**>
 </**featureList**>
 </**participant**>
 </**participantList**>
 <**interactionType**>
 <**names**>
 <**shortLabel**>physical association</**shortLabel**>
 <**fullName**>physical association</**fullName**>
 </**names**>
 <**xref**>
 <**primaryRef db="psi-mi" dbAc="MI:0488" id="MI:0915" refType="identity" refTypeAc="MI:0356"**/>
 <**secondaryRef db="intact" dbAc="MI:0469" id="EBI-1813147" refType="identity" refTypeAc="MI:0356"**/>
 <**secondaryRef db="pubmed" dbAc="MI:0446" id="14755292" refType="primary-reference" refTypeAc="MI:0358"**/>
 </**xref**>
 </**interactionType**>
 <**attributeList**>
 <**attribute name="source-text"**>Among ten human glycolytic enzymes co-expressed individually with
 HA-c-Src, HK1 was exclusively precipitated by HA-c-Src (Fig. 1a).
 </**attribute**>
 <**attribute name="figure legend" nameAc="MI:0599"**>Fig. 1B, 2A, 2B, 2C, 2G, 2I</**attribute**>
 <**attribute name="comment" nameAc="MI:0612"**>Kinase-dead SRC failed to phosphorylate HK1 and
 phosphorylation was inhibited by c-Src inhibitors PP2 and SU6656
 </**attribute**>
 </**attributeList**>
 </**interaction**>
 </**interactionList**>
 </**entry**>
</**entrySet**>
