# Supplementary material for: Encompassing new use cases - level 3.0 of the HUPO-PSI format for molecular interactions
Source: BMC Bioinformatics. 2018 Apr 11;19:134. doi: 10.1186/s12859-018-2118-1 (PMC5896046; doi:10.1186/s12859-018-2118-1)
Supplement: Supplementary file 5 — Representation of kinetic parameters added at feature level (use case 1.3e). (https://github.com/HUPO-PSI/miXML/blob/master/3.0/pub/Appendix%206.docx). (DOCX 39 kb) [file 12859_2018_2118_MOESM5_ESM.docx]

**Kinetic parameters added at feature level**

PMID:22087277

A causal single nucleotide polymorphism (SNP) in the MST1 gene encoding Macrophage Stimulating Protein (MSP), results in an R689C amino acid substitution within the β-chain of MSP (MSPβ). This variant binds to the RON receptor tyrosine kinase with a Kd=1.39x10-7M.

<**feature id="9"**>

<**names**>

<**shortLabel**>arg689cys</**shortLabel**>

<**fullName**>R689C</**fullName**>

</**names**>

<**xref**>

<**primaryRef db="intact" dbAc="MI:0469" id="EBI-11167329" refType="identity" refTypeAc="MI:0356"**/>

</**xref**>

<**featureType**>

<**names**>

<**shortLabel**>variant</**shortLabel**>

<**fullName**>variant</**fullName**>

</**names**>

<**xref**>

<**primaryRef db="psi-mi" dbAc="MI:0488" id="MI:1241" refType="identity" refTypeAc="MI:0356"**/>

<**secondaryRef db="intact" dbAc="MI:0469" id="EBI-6354006" refType="identity" refTypeAc="MI:0356"**/>

</**xref**>

</**featureType**>

<**featureRangeList**>

<**featureRange**>

<**startStatus**>

<**names**>

<**shortLabel**>certain</**shortLabel**>

<**fullName**>certain sequence position</**fullName**>

<**alias type="synonym" typeAc="MI:1041"**>certain</**alias**>

</**names**>

<**xref**>

<**primaryRef db="psi-mi" dbAc="MI:0488" id="MI:0335" refType="identity" refTypeAc="MI:0356"**/>

<**secondaryRef db="intact" dbAc="MI:0469" id="EBI-540564" refType="identity" refTypeAc="MI:0356"**/>

<**secondaryRef db="pubmed" dbAc="MI:0446" id="14755292" refType="primary-reference" refTypeAc="MI:0358"**/>

</**xref**>

</**startStatus**>

<**begin position="689"**/>

<**endStatus**>

<**names**>

<**shortLabel**>certain</**shortLabel**>

<**fullName**>certain sequence position</**fullName**>

<**alias type="synonym" typeAc="MI:1041"**>certain</**alias**>

</**names**>

<**xref**>

<**primaryRef db="psi-mi" dbAc="MI:0488" id="MI:0335" refType="identity" refTypeAc="MI:0356"**/>

<**secondaryRef db="intact" dbAc="MI:0469" id="EBI-540564" refType="identity" refTypeAc="MI:0356"**/>

<**secondaryRef db="pubmed" dbAc="MI:0446" id="14755292" refType="primary-reference" refTypeAc="MI:0358"**/>

</**xref**>

</**endStatus**>

<**end position="689"**/>

<**resultingSequence**>

<**originalSequence**>R</**originalSequence**>

<**newSequence**>C</**newSequence**>

</**resultingSequence**>

</**featureRange**>

</**featureRangeList**>

<**parameterList**>

<**parameter term="kd" termAc="MI:0646" unit="molar" unitAc="MI:0648" base="10" exponent="-7" factor="1.39"**/>

</**parameterList**>

</**feature**>

File:

*<?***xml version="1.0" encoding="UTF-8"***?>*

<**entrySet xmlns:xsi="http://www.w3.org/2001/XMLSchema-instance" xmlns="http://psi.hupo.org/mi/mif300"**

**xsi:schemaLocation="http://psi.hupo.org/mi/mif300 https://raw.githubusercontent.com/HUPO-PSI/miXML/master/3.0/src/MIF300.xsd"**

**level="3" version="0" minorVersion="0"**>

<**entry**>

<**source releaseDate="2017-05-17"**>

<**names**>

<**shortLabel**>IntAct</**shortLabel**>

<**fullName**>European Bioinformatics Institute</**fullName**>

<**alias type="synonym" typeAc="MI:1041"**>IntAct</**alias**>

</**names**>

<**bibref**>

<**xref**>

<**primaryRef db="pubmed" dbAc="MI:0446" id="14681455" refType="primary-reference" refTypeAc="MI:0358"**/>

</**xref**>

</**bibref**>

<**xref**>

<**primaryRef db="psi-mi" dbAc="MI:0488" id="MI:0469" refType="identity" refTypeAc="MI:0356"**/>

<**secondaryRef db="intact" dbAc="MI:0469" id="EBI-10" refType="identity" refTypeAc="MI:0356"**/>

<**secondaryRef db="pubmed" dbAc="MI:0446" id="14681455" refType="primary-reference" refTypeAc="MI:0358"**/>

<**secondaryRef db="pubmed" dbAc="MI:0446" id="22121220" refType="method reference" refTypeAc="MI:0357"**/>

<**secondaryRef db="pubmed" dbAc="MI:0446" id="19850723" refType="method reference" refTypeAc="MI:0357"**/>

</**xref**>

<**attributeList**>

<**attribute name="url" nameAc="MI:0614"**>http://www.ebi.ac.uk/</**attribute**>

<**attribute name="search-url" nameAc="MI:0615"**>http://www.ebi.ac.uk/intact/query/${ac}</**attribute**>

<**attribute name="id-validation-regexp" nameAc="MI:0628"**>EBI-[0-9]+|IA:[0-9]+</**attribute**>

<**attribute name="definition"**>

INTerAction database (IntAct) provides an open source database and toolkit for the storage, presentation and analysis of molecular interactions.

</**attribute**>

<**attribute name="url" nameAc="MI:0614"**>http://www.ebi.ac.uk/intact</**attribute**>

<**attribute name="postaladdress"**>

European Bioinformatics Institute; Wellcome Trust Genome Campus; Hinxton, Cambridge; CB10 1SD; United Kingdom

</**attribute**>

<**attribute name="url" nameAc="MI:0614"**>http://www.ebi.ac.uk/intact/</**attribute**>

</**attributeList**>

</**source**>

<**experimentList**>

<**experimentDescription id="1"**>

<**names**>

<**fullName**>

Protein characterization of a candidate mechanism SNP for Crohn's disease: the macrophage stimulating protein R689C substitution.

</**fullName**>

</**names**>

<**bibref**>

<**xref**>

<**primaryRef db="pubmed" dbAc="MI:0446" id="22087277" refType="primary-reference" refTypeAc="MI:0358"**/>

<**secondaryRef db="intact" dbAc="MI:0469" id="EBI-10902752" refType="identity" refTypeAc="MI:0356"**/>

<**secondaryRef db="imex" dbAc="MI:0670" id="IM-24508" refType="imex-primary" refTypeAc="MI:0662"**/>

<**secondaryRef db="pubmed" dbAc="MI:0446" id="25193665" refType="see-also" refTypeAc="MI:0361"**/>

</**xref**>

<**attributeList**>

<**attribute name="publication title" nameAc="MI:1091"**>

Protein characterization of a candidate mechanism SNP for Crohn's disease: the macrophage stimulating protein R689C substitution.

</**attribute**>

<**attribute name="journal" nameAc="MI:0885"**>PloS one</**attribute**>

<**attribute name="publication year" nameAc="MI:0886"**>2010</**attribute**>

<**attribute name="curation depth" nameAc="MI:0955"**>imex curation</**attribute**>

<**attribute name="imex curation" nameAc="MI:0959"**/>

<**attribute name="author-list" nameAc="MI:0636"**>

Gorlatova N., Chao K., Pal LR., Araj RH., Galkin A., Turko I., Moult J., Herzberg O.

</**attribute**>

<**attribute name="contact-email" nameAc="MI:0634"**>jmoult@umd.edu,osnat@umd.edu</**attribute**>

<**attribute name="dataset" nameAc="MI:0875"**>

Crohns disease - Interactions of proteins identified as having a link to Crohn's disease through GWAS studies

</**attribute**>

<**attribute name="full coverage" nameAc="MI:0957"**>Only protein-protein interactions</**attribute**>

<**attribute name="imex curation" nameAc="MI:0959"**/>

<**attribute name="author-announcement"**>21-10-2015: Contacted by IntAct-Help.</**attribute**>

</**attributeList**>

</**bibref**>

<**xref**>

<**primaryRef db="imex" dbAc="MI:0670" id="IM-24508" refType="imex-primary" refTypeAc="MI:0662"**/>

<**secondaryRef db="pubmed" dbAc="MI:0446" id="22087277" refType="primary-reference" refTypeAc="MI:0358"**/>

</**xref**>

<**hostOrganismList**>

<**hostOrganism ncbiTaxId="-1"**>

<**names**>

<**shortLabel**>in vitro</**shortLabel**>

<**fullName**>In vitro</**fullName**>

</**names**>

</**hostOrganism**>

</**hostOrganismList**>

<**interactionDetectionMethod**>

<**names**>

<**shortLabel**>spr</**shortLabel**>

<**fullName**>surface plasmon resonance</**fullName**>

<**alias type="go synonym" typeAc="MI:0303"**>Optical biosensor</**alias**>

<**alias type="go synonym" typeAc="MI:0303"**>BIAcore(r)</**alias**>

</**names**>

<**xref**>

<**primaryRef db="psi-mi" dbAc="MI:0488" id="MI:0107" refType="identity" refTypeAc="MI:0356"**/>

<**secondaryRef db="intact" dbAc="MI:0469" id="EBI-1252" refType="identity" refTypeAc="MI:0356"**/>

<**secondaryRef db="pubmed" dbAc="MI:0446" id="11896282" refType="primary-reference" refTypeAc="MI:0358"**/>

<**secondaryRef db="pubmed" dbAc="MI:0446" id="16338355" refType="primary-reference" refTypeAc="MI:0358"**/>

<**secondaryRef db="pubmed" dbAc="MI:0446" id="12120258" refType="primary-reference" refTypeAc="MI:0358"**/>

</**xref**>

</**interactionDetectionMethod**>

<**participantIdentificationMethod**>

<**names**>

<**shortLabel**>predetermined</**shortLabel**>

<**fullName**>predetermined participant</**fullName**>

<**alias type="synonym" typeAc="MI:1041"**>predetermined</**alias**>

</**names**>

<**xref**>

<**primaryRef db="psi-mi" dbAc="MI:0488" id="MI:0396" refType="identity" refTypeAc="MI:0356"**/>

<**secondaryRef db="intact" dbAc="MI:0469" id="EBI-1465" refType="identity" refTypeAc="MI:0356"**/>

<**secondaryRef db="pubmed" dbAc="MI:0446" id="14755292" refType="primary-reference" refTypeAc="MI:0358"**/>

</**xref**>

</**participantIdentificationMethod**>

<**attributeList**>

<**attribute name="journal" nameAc="MI:0885"**>PloS one</**attribute**>

<**attribute name="publication year" nameAc="MI:0886"**>2010</**attribute**>

<**attribute name="contact-email" nameAc="MI:0634"**>jmoult@umd.edu,osnat@umd.edu</**attribute**>

<**attribute name="author-list" nameAc="MI:0636"**>Gorlatova N., Chao K., Pal LR., Araj RH., Galkin A., Turko I., Moult J., Herzberg O.</**attribute**>

<**attribute name="curation depth" nameAc="MI:0955"**>imex curation</**attribute**>

<**attribute name="dataset" nameAc="MI:0875"**>

Crohns disease - Interactions of proteins identified as having a link to Crohn's disease through GWAS studies

</**attribute**>

<**attribute name="accepted"**>Accepted 2015-SEP-04 by ORCHARD</**attribute**>

<**attribute name="correction comment"**/>

</**attributeList**>

</**experimentDescription**>

</**experimentList**>

<**interactorList**>

<**interactor id="2"**>

<**names**>

<**shortLabel**>hgfl_human</**shortLabel**>

<**fullName**>Hepatocyte growth factor-like protein</**fullName**>

<**alias type="gene name synonym" typeAc="MI:0302"**>D3F15S2</**alias**>

<**alias type="gene name synonym" typeAc="MI:0302"**>DNF15S2</**alias**>

<**alias type="gene name synonym" typeAc="MI:0302"**>HGFL</**alias**>

<**alias type="gene name synonym" typeAc="MI:0302"**>Macrophage stimulatory protein</**alias**>

<**alias type="gene name synonym" typeAc="MI:0302"**>Macrophage-stimulating protein</**alias**>

<**alias type="gene name" typeAc="MI:0301"**>MST1</**alias**>

</**names**>

<**xref**>

<**primaryRef db="uniprotkb" dbAc="MI:0486" id="P26927" refType="identity" refTypeAc="MI:0356"**/>

<**secondaryRef db="uniprotkb" dbAc="MI:0486" id="Q6GTN4" refType="secondary-ac" refTypeAc="MI:0360"**/>

<**secondaryRef db="uniprotkb" dbAc="MI:0486" id="A8MSX3" refType="secondary-ac" refTypeAc="MI:0360"**/>

<**secondaryRef db="uniprotkb" dbAc="MI:0486" id="Q14870" refType="secondary-ac" refTypeAc="MI:0360"**/>

<**secondaryRef db="uniprotkb" dbAc="MI:0486" id="Q13350" refType="secondary-ac" refTypeAc="MI:0360"**/>

<**secondaryRef db="uniprotkb" dbAc="MI:0486" id="A6NLA3" refType="secondary-ac" refTypeAc="MI:0360"**/>

<**secondaryRef db="intact" dbAc="MI:0469" id="EBI-6929133" refType="identity" refTypeAc="MI:0356"**/>

<**secondaryRef db="go" dbAc="MI:0448" id="GO:0030971"**/>

<**secondaryRef db="go" dbAc="MI:0448" id="GO:0004252"**/>

<**secondaryRef db="interpro" dbAc="MI:0449" id="IPR000001"**/>

<**secondaryRef db="interpro" dbAc="MI:0449" id="IPR001254"**/>

<**secondaryRef db="interpro" dbAc="MI:0449" id="IPR001314"**/>

<**secondaryRef db="interpro" dbAc="MI:0449" id="IPR003609"**/>

<**secondaryRef db="interpro" dbAc="MI:0449" id="IPR009003"**/>

<**secondaryRef db="interpro" dbAc="MI:0449" id="IPR013806"**/>

<**secondaryRef db="interpro" dbAc="MI:0449" id="IPR018056"**/>

<**secondaryRef db="interpro" dbAc="MI:0449" id="IPR024174"**/>

<**secondaryRef db="rcsb pdb" dbAc="MI:0460" id="2ASU"**/>

<**secondaryRef db="refseq" dbAc="MI:0481" id="NP_066278.3"**/>

<**secondaryRef db="ensembl" dbAc="MI:0476" id="ENST00000383728"**/>

<**secondaryRef db="rcsb pdb" dbAc="MI:0460" id="4QT8"**/>

<**secondaryRef db="go" dbAc="MI:0448" id="GO:0070062"**/>

<**secondaryRef db="ensembl" dbAc="MI:0476" id="ENSG00000173531"**/>

<**secondaryRef db="ensembl" dbAc="MI:0476" id="ENSP00000373234"**/>

<**secondaryRef db="go" dbAc="MI:0448" id="GO:0005576"**/>

<**secondaryRef db="go" dbAc="MI:0448" id="GO:0005615"**/>

<**secondaryRef db="go" dbAc="MI:0448" id="GO:0045721"**/>

<**secondaryRef db="go" dbAc="MI:0448" id="GO:0048012"**/>

<**secondaryRef db="go" dbAc="MI:0448" id="GO:2000479"**/>

<**secondaryRef db="reactome" dbAc="MI:0467" id="R-HSA-8852405"**/>

</**xref**>

<**interactorType**>

<**names**>

<**shortLabel**>protein</**shortLabel**>

<**fullName**>protein</**fullName**>

</**names**>

<**xref**>

<**primaryRef db="psi-mi" dbAc="MI:0488" id="MI:0326" refType="identity" refTypeAc="MI:0356"**/>

<**secondaryRef db="intact" dbAc="MI:0469" id="EBI-619654" refType="identity" refTypeAc="MI:0356"**/>

<**secondaryRef db="pubmed" dbAc="MI:0446" id="14755292" refType="primary-reference" refTypeAc="MI:0358"**/>

<**secondaryRef db="so" dbAc="MI:0601" id="SO:0000358" refType="see-also" refTypeAc="MI:0361"**/>

</**xref**>

</**interactorType**>

<**organism ncbiTaxId="9606"**>

<**names**>

<**shortLabel**>human</**shortLabel**>

<**fullName**>Homo sapiens</**fullName**>

<**alias type="synonym" typeAc="MI:1041"**>Human</**alias**>

</**names**>

</**organism**>

<**sequence**>

MGWLPLLLLLTQCLGVPGQRSPLNDFQVLRGTELQHLLHAVVPGPWQEDVADAEECAGRCGPLMDCRAFHYNVSSHGCQLLPWTQHSPHTRLRRSGRCDLFQKKDYVRTCIMNNGVGYRGTMATTVGGLPCQAWSHKFPNDHKYTPTLRNGLEENFCRNPDGDPGGPWCYTTDPAVRFQSCGIKSCREAACVWCNGEEYRGAVDRTESGRECQRWDLQHPHQHPFEPGKFLDQGLDDNYCRNPDGSERPWCYTTDPQIEREFCDLPRCGSEAQPRQEATTVSCFRGKGEGYRGTANTTTAGVPCQRWDAQIPHQHRFTPEKYACKDLRENFCRNPDGSEAPWCFTLRPGMRAAFCYQIRRCTDDVRPQDCYHGAGEQYRGTVSKTRKGVQCQRWSAETPHKPQFTFTSEPHAQLEENFCRNPDGDSHGPWCYTMDPRTPFDYCALRRCADDQPPSILDPPDQVQFEKCGKRVDRLDQRRSKLRVVGGHPGNSPWTVSLRNRQGQHFCGGSLVKEQWILTARQCFSSCHMPLTGYEVWLGTLFQNPQHGEPSLQRVPVAKMVCGPSGSQLVLLKLERSVTLNQRVALICLPPEWYVVPPGTKCEIAGWGETKGTGNDTVLNVALLNVISNQECNIKHRGRVRESEMCTEGLLAPVGACEGDYGGPLACFTHNCWVLEGIIIPNRVCARSRWPAVFTRVSVFVDWIHKVMRLG

</**sequence**>

<**attributeList**>

<**attribute name="crc64"**>2E4B3C7D4AA9B566</**attribute**>

</**attributeList**>

</**interactor**>

<**interactor id="3"**>

<**names**>

<**shortLabel**>ron_human</**shortLabel**>

<**fullName**>Macrophage-stimulating protein receptor</**fullName**>

<**alias type="gene name" typeAc="MI:0301"**>MST1R</**alias**>

<**alias type="gene name synonym" typeAc="MI:0302"**>PTK8</**alias**>

<**alias type="gene name synonym" typeAc="MI:0302"**>RON</**alias**>

<**alias type="gene name synonym" typeAc="MI:0302"**>p185-Ron</**alias**>

<**alias type="gene name synonym" typeAc="MI:0302"**>Protein-tyrosine kinase 8</**alias**>

<**alias type="gene name synonym" typeAc="MI:0302"**>CDw136</**alias**>

</**names**>

<**xref**>

<**primaryRef db="uniprotkb" dbAc="MI:0486" id="Q04912" version="SP_113" refType="identity" refTypeAc="MI:0356"**/>

<**secondaryRef db="uniprotkb" dbAc="MI:0486" id="B5A944" version="SP_120" refType="secondary-ac" refTypeAc="MI:0360"**/>

<**secondaryRef db="uniprotkb" dbAc="MI:0486" id="B5A945" version="SP_120" refType="secondary-ac" refTypeAc="MI:0360"**/>

<**secondaryRef db="uniprotkb" dbAc="MI:0486" id="B5A946" version="SP_120" refType="secondary-ac" refTypeAc="MI:0360"**/>

<**secondaryRef db="uniprotkb" dbAc="MI:0486" id="B5A947" version="SP_120" refType="secondary-ac" refTypeAc="MI:0360"**/>

<**secondaryRef db="intact" dbAc="MI:0469" id="EBI-2637518" refType="identity" refTypeAc="MI:0356"**/>

<**secondaryRef db="go" dbAc="MI:0448" id="GO:0045087"**/>

<**secondaryRef db="ensembl" dbAc="MI:0476" id="ENSP00000296474"**/>

<**secondaryRef db="ensembl" dbAc="MI:0476" id="ENSP00000341325"**/>

<**secondaryRef db="go" dbAc="MI:0448" id="GO:0005887"**/>

<**secondaryRef db="go" dbAc="MI:0448" id="GO:0005524"**/>

<**secondaryRef db="go" dbAc="MI:0448" id="GO:0005011"**/>

<**secondaryRef db="go" dbAc="MI:0448" id="GO:0006928"**/>

<**secondaryRef db="go" dbAc="MI:0448" id="GO:0008284"**/>

<**secondaryRef db="go" dbAc="MI:0448" id="GO:0007338"**/>

<**secondaryRef db="ensembl" dbAc="MI:0476" id="ENSG00000164078"**/>

<**secondaryRef db="interpro" dbAc="MI:0449" id="IPR013783"**/>

<**secondaryRef db="interpro" dbAc="MI:0449" id="IPR014756"**/>

<**secondaryRef db="interpro" dbAc="MI:0449" id="IPR002909"**/>

<**secondaryRef db="interpro" dbAc="MI:0449" id="IPR011009"**/>

<**secondaryRef db="interpro" dbAc="MI:0449" id="IPR016201"**/>

<**secondaryRef db="interpro" dbAc="MI:0449" id="IPR000719"**/>

<**secondaryRef db="interpro" dbAc="MI:0449" id="IPR017441"**/>

<**secondaryRef db="interpro" dbAc="MI:0449" id="IPR001627"**/>

<**secondaryRef db="interpro" dbAc="MI:0449" id="IPR001245"**/>

<**secondaryRef db="interpro" dbAc="MI:0449" id="IPR008266"**/>

<**secondaryRef db="interpro" dbAc="MI:0449" id="IPR020635"**/>

<**secondaryRef db="interpro" dbAc="MI:0449" id="IPR016244"**/>

<**secondaryRef db="interpro" dbAc="MI:0449" id="IPR015943"**/>

<**secondaryRef db="rcsb pdb" dbAc="MI:0460" id="3PLS"**/>

<**secondaryRef db="go" dbAc="MI:0448" id="GO:0009615"**/>

<**secondaryRef db="go" dbAc="MI:0448" id="GO:0043406"**/>

<**secondaryRef db="go" dbAc="MI:0448" id="GO:0051897"**/>

<**secondaryRef db="go" dbAc="MI:0448" id="GO:0006952"**/>

<**secondaryRef db="rcsb pdb" dbAc="MI:0460" id="4FWW"**/>

<**secondaryRef db="go" dbAc="MI:0448" id="GO:0001725"**/>

<**secondaryRef db="go" dbAc="MI:0448" id="GO:0007165"**/>

<**secondaryRef db="go" dbAc="MI:0448" id="GO:0019899"**/>

<**secondaryRef db="ensembl" dbAc="MI:0476" id="ENST00000296474"**/>

<**secondaryRef db="ensembl" dbAc="MI:0476" id="ENST00000344206"**/>

<**secondaryRef db="go" dbAc="MI:0448" id="GO:0009986"**/>

<**secondaryRef db="rcsb pdb" dbAc="MI:0460" id="4QT8"**/>

<**secondaryRef db="go" dbAc="MI:0448" id="GO:0005886"**/>

<**secondaryRef db="go" dbAc="MI:0448" id="GO:0048012"**/>

<**secondaryRef db="reactome" dbAc="MI:0467" id="R-HSA-8852405"**/>

<**secondaryRef db="refseq" dbAc="MI:0481" id="NP_001231866.1"**/>

<**secondaryRef db="refseq" dbAc="MI:0481" id="NP_002438.2"**/>

<**secondaryRef db="go" dbAc="MI:0448" id="GO:0005773"**/>

</**xref**>

<**interactorType**>

<**names**>

<**shortLabel**>protein</**shortLabel**>

<**fullName**>protein</**fullName**>

</**names**>

<**xref**>

<**primaryRef db="psi-mi" dbAc="MI:0488" id="MI:0326" refType="identity" refTypeAc="MI:0356"**/>

<**secondaryRef db="intact" dbAc="MI:0469" id="EBI-619654" refType="identity" refTypeAc="MI:0356"**/>

<**secondaryRef db="pubmed" dbAc="MI:0446" id="14755292" refType="primary-reference" refTypeAc="MI:0358"**/>

<**secondaryRef db="so" dbAc="MI:0601" id="SO:0000358" refType="see-also" refTypeAc="MI:0361"**/>

</**xref**>

</**interactorType**>

<**organism ncbiTaxId="9606"**>

<**names**>

<**shortLabel**>human</**shortLabel**>

<**fullName**>Homo sapiens</**fullName**>

<**alias type="synonym" typeAc="MI:1041"**>Human</**alias**>

</**names**>

</**organism**>

<**sequence**>

MELLPPLPQSFLLLLLLPAKPAAGEDWQCPRTPYAASRDFDVKYVVPSFSAGGLVQAMVTYEGDRNESAVFVAIRNRLHVLGPDLKSVQSLATGPAGDPGCQTCAACGPGPHGPPGDTDTKVLVLDPALPALVSCGSSLQGRCFLHDLEPQGTAVHLAAPACLFSAHHNRPDDCPDCVASPLGTRVTVVEQGQASYFYVASSLDAAVAASFSPRSVSIRRLKADASGFAPGFVALSVLPKHLVSYSIEYVHSFHTGAFVYFLTVQPASVTDDPSALHTRLARLSATEPELGDYRELVLDCRFAPKRRRRGAPEGGQPYPVLRVAHSAPVGAQLATELSIAEGQEVLFGVFVTGKDGGPGVGPNSVVCAFPIDLLDTLIDEGVERCCESPVHPGLRRGLDFFQSPSFCPNPPGLEALSPNTSCRHFPLLVSSSFSRVDLFNGLLGPVQVTALYVTRLDNVTVAHMGTMDGRILQVELVRSLNYLLYVSNFSLGDSGQPVQRDVSRLGDHLLFASGDQVFQVPIQGPGCRHFLTCGRCLRAWHFMGCGWCGNMCGQQKECPGSWQQDHCPPKLTEFHPHSGPLRGSTRLTLCGSNFYLHPSGLVPEGTHQVTVGQSPCRPLPKDSSKLRPVPRKDFVEEFECELEPLGTQAVGPTNVSLTVTNMPPGKHFRVDGTSVLRGFSFMEPVLIAVQPLFGPRAGGTCLTLEGQSLSVGTSRAVLVNGTECLLARVSEGQLLCATPPGATVASVPLSLQVGGAQVPGSWTFQYREDPVVLSISPNCGYINSHITICGQHLTSAWHLVLSFHDGLRAVESRCERQLPEQQLCRLPEYVVRDPQGWVAGNLSARGDGAAGFTLPGFRFLPPPHPPSANLVPLKPEEHAIKFEYIGLGAVADCVGINVTVGGESCQHEFRGDMVVCPLPPSLQLGQDGAPLQVCVDGECHILGRVVRPGPDGVPQSTLLGILLPLLLLVAALATALVFSYWWRRKQLVLPPNLNDLASLDQTAGATPLPILYSGSDYRSGLALPAIDGLDSTTCVHGASFSDSEDESCVPLLRKESIQLRDLDSALLAEVKDVLIPHERVVTHSDRVIGKGHFGVVYHGEYIDQAQNRIQCAIKSLSRITEMQQVEAFLREGLLMRGLNHPNVLALIGIMLPPEGLPHVLLPYMCHGDLLQFIRSPQRNPTVKDLISFGLQVARSMEYLAEQKFVHRDLAARNCMLDESFTVKVADFGLARDILDREYYSVQQHRHARLPVKWMALESLQTYRFTTKSDVWSFGVLLWELLTRGAPPYRHIDPFDLTHFLAQGRRLPQPEYCPDSLYQVMQQCWEADPAVRPTFRVLVGEVEQIVSALLGDHYVQLPATYMNLGPSTSHEMNVRPEQPQFSPMPGNVRRPRPLSEPPRPT

</**sequence**>

<**attributeList**>

<**attribute name="crc64"**>EB5CA79ABC69A882</**attribute**>

</**attributeList**>

</**interactor**>

</**interactorList**>

<**interactionList**>

<**interaction id="4" imexId="IM-24508-1"**>

<**names**>

<**shortLabel**>mst1r-mst1-1</**shortLabel**>

</**names**>

<**xref**>

<**primaryRef db="intact" dbAc="MI:0469" id="EBI-10956041" refType="identity" refTypeAc="MI:0356"**/>

<**secondaryRef db="imex" dbAc="MI:0670" id="IM-24508-1" refType="imex-primary" refTypeAc="MI:0662"**/>

</**xref**>

<**experimentList**>

<**experimentRef**>1</**experimentRef**>

</**experimentList**>

<**participantList**>

<**participant id="5"**>

<**interactorRef**>3</**interactorRef**>

<**biologicalRole**>

<**names**>

<**shortLabel**>unspecified role</**shortLabel**>

<**fullName**>unspecified role</**fullName**>

</**names**>

<**xref**>

<**primaryRef db="psi-mi" dbAc="MI:0488" id="MI:0499" refType="identity" refTypeAc="MI:0356"**/>

<**secondaryRef db="intact" dbAc="MI:0469" id="EBI-77781" refType="identity" refTypeAc="MI:0356"**/>

<**secondaryRef db="pubmed" dbAc="MI:0446" id="14755292" refType="primary-reference" refTypeAc="MI:0358"**/>

</**xref**>

</**biologicalRole**>

<**experimentalRoleList**>

<**experimentalRole**>

<**names**>

<**shortLabel**>bait</**shortLabel**>

<**fullName**>bait</**fullName**>

</**names**>

<**xref**>

<**primaryRef db="psi-mi" dbAc="MI:0488" id="MI:0496" refType="identity" refTypeAc="MI:0356"**/>

<**secondaryRef db="intact" dbAc="MI:0469" id="EBI-49" refType="identity" refTypeAc="MI:0356"**/>

<**secondaryRef db="pubmed" dbAc="MI:0446" id="14755292" refType="primary-reference" refTypeAc="MI:0358"**/>

</**xref**>

</**experimentalRole**>

</**experimentalRoleList**>

<**featureList**>

<**feature id="6"**>

<**names**>

<**shortLabel**>c-terminus</**shortLabel**>

<**fullName**>RONSema</**fullName**>

</**names**>

<**xref**>

<**primaryRef db="intact" dbAc="MI:0469" id="EBI-10957304" refType="identity" refTypeAc="MI:0356"**/>

</**xref**>

<**featureType**>

<**names**>

<**shortLabel**>his tag</**shortLabel**>

<**fullName**>his tag</**fullName**>

<**alias type="go synonym" typeAc="MI:0303"**>Hexa-His-tag</**alias**>

<**alias type="go synonym" typeAc="MI:0303"**>6-His-tag</**alias**>

<**alias type="go synonym" typeAc="MI:0303"**>Histidine-tag</**alias**>

</**names**>

<**xref**>

<**primaryRef db="psi-mi" dbAc="MI:0488" id="MI:0521" refType="identity" refTypeAc="MI:0356"**/>

<**secondaryRef db="intact" dbAc="MI:0469" id="EBI-456516" refType="identity" refTypeAc="MI:0356"**/>

<**secondaryRef db="pubmed" dbAc="MI:0446" id="14755292" refType="primary-reference" refTypeAc="MI:0358"**/>

</**xref**>

</**featureType**>

<**featureRangeList**>

<**featureRange**>

<**startStatus**>

<**names**>

<**shortLabel**>c-term range</**shortLabel**>

<**fullName**>C-terminal range</**fullName**>

</**names**>

<**xref**>

<**primaryRef db="psi-mi" dbAc="MI:0488" id="MI:1039" refType="identity" refTypeAc="MI:0356"**/>

<**secondaryRef db="intact" dbAc="MI:0469" id="EBI-2929799" refType="identity" refTypeAc="MI:0356"**/>

<**secondaryRef db="pubmed" dbAc="MI:0446" id="14760721" refType="primary-reference" refTypeAc="MI:0358"**/>

</**xref**>

</**startStatus**>

<**endStatus**>

<**names**>

<**shortLabel**>c-term range</**shortLabel**>

<**fullName**>C-terminal range</**fullName**>

</**names**>

<**xref**>

<**primaryRef db="psi-mi" dbAc="MI:0488" id="MI:1039" refType="identity" refTypeAc="MI:0356"**/>

<**secondaryRef db="intact" dbAc="MI:0469" id="EBI-2929799" refType="identity" refTypeAc="MI:0356"**/>

<**secondaryRef db="pubmed" dbAc="MI:0446" id="14760721" refType="primary-reference" refTypeAc="MI:0358"**/>

</**xref**>

</**endStatus**>

</**featureRange**>

</**featureRangeList**>

</**feature**>

<**feature id="7"**>

<**names**>

<**shortLabel**>region</**shortLabel**>

<**fullName**>RONSema</**fullName**>

</**names**>

<**xref**>

<**primaryRef db="intact" dbAc="MI:0469" id="EBI-10957051" refType="identity" refTypeAc="MI:0356"**/>

</**xref**>

<**featureType**>

<**names**>

<**shortLabel**>sufficient to bind</**shortLabel**>

<**fullName**>sufficient binding region</**fullName**>

<**alias type="synonym" typeAc="MI:1041"**>sufficient to bind</**alias**>

</**names**>

<**xref**>

<**primaryRef db="psi-mi" dbAc="MI:0488" id="MI:0442" refType="identity" refTypeAc="MI:0356"**/>

<**secondaryRef db="intact" dbAc="MI:0469" id="EBI-608899" refType="identity" refTypeAc="MI:0356"**/>

<**secondaryRef db="pubmed" dbAc="MI:0446" id="14755292" refType="primary-reference" refTypeAc="MI:0358"**/>

</**xref**>

</**featureType**>

<**featureRangeList**>

<**featureRange**>

<**startStatus**>

<**names**>

<**shortLabel**>certain</**shortLabel**>

<**fullName**>certain sequence position</**fullName**>

<**alias type="synonym" typeAc="MI:1041"**>certain</**alias**>

</**names**>

<**xref**>

<**primaryRef db="psi-mi" dbAc="MI:0488" id="MI:0335" refType="identity" refTypeAc="MI:0356"**/>

<**secondaryRef db="intact" dbAc="MI:0469" id="EBI-540564" refType="identity" refTypeAc="MI:0356"**/>

<**secondaryRef db="pubmed" dbAc="MI:0446" id="14755292" refType="primary-reference" refTypeAc="MI:0358"**/>

</**xref**>

</**startStatus**>

<**begin position="25"**/>

<**endStatus**>

<**names**>

<**shortLabel**>certain</**shortLabel**>

<**fullName**>certain sequence position</**fullName**>

<**alias type="synonym" typeAc="MI:1041"**>certain</**alias**>

</**names**>

<**xref**>

<**primaryRef db="psi-mi" dbAc="MI:0488" id="MI:0335" refType="identity" refTypeAc="MI:0356"**/>

<**secondaryRef db="intact" dbAc="MI:0469" id="EBI-540564" refType="identity" refTypeAc="MI:0356"**/>

<**secondaryRef db="pubmed" dbAc="MI:0446" id="14755292" refType="primary-reference" refTypeAc="MI:0358"**/>

</**xref**>

</**endStatus**>

<**end position="524"**/>

</**featureRange**>

</**featureRangeList**>

</**feature**>

</**featureList**>

<**hostOrganismList**>

<**hostOrganism ncbiTaxId="7227"**>

<**names**>

<**shortLabel**>drome-schneider_2</**shortLabel**>

<**fullName**>Drosophila melanogaster late embryo cells</**fullName**>

</**names**>

<**cellType**>

<**names**>

<**shortLabel**>schneider_2</**shortLabel**>

<**fullName**>Established from the late embryo of Drosophila melanogaster</**fullName**>

</**names**>

<**xref**>

<**primaryRef db="cabri" dbAc="MI:0246" id="ACC 130" refType="identity" refTypeAc="MI:0356"**/>

<**secondaryRef db="intact" dbAc="MI:0469" id="IA:0067" refType="identity" refTypeAc="MI:0356"**/>

<**secondaryRef db="mint" dbAc="MI:0471" id="MINT-2569257" refType="identity" refTypeAc="MI:0356"**/>

<**secondaryRef db="intact" dbAc="MI:0469" id="EBI-307758" refType="identity" refTypeAc="MI:0356"**/>

</**xref**>

<**attributeList**>

<**attribute name="comment" nameAc="MI:0612"**>

Mixture of diploid and tetraploid. Originally had some XY cells now predominantly XX.

</**attribute**>

</**attributeList**>

</**cellType**>

</**hostOrganism**>

</**hostOrganismList**>

<**parameterList**>

<**parameter term="kd" termAc="MI:0646" unit="molar" unitAc="MI:0648" base="10" exponent="-9" factor="16.40"**/>

</**parameterList**>

</**participant**>

<**participant id="8"**>

<**interactorRef**>2</**interactorRef**>

<**biologicalRole**>

<**names**>

<**shortLabel**>unspecified role</**shortLabel**>

<**fullName**>unspecified role</**fullName**>

</**names**>

<**xref**>

<**primaryRef db="psi-mi" dbAc="MI:0488" id="MI:0499" refType="identity" refTypeAc="MI:0356"**/>

<**secondaryRef db="intact" dbAc="MI:0469" id="EBI-77781" refType="identity" refTypeAc="MI:0356"**/>

<**secondaryRef db="pubmed" dbAc="MI:0446" id="14755292" refType="primary-reference" refTypeAc="MI:0358"**/>

</**xref**>

</**biologicalRole**>

<**experimentalRoleList**>

<**experimentalRole**>

<**names**>

<**shortLabel**>prey</**shortLabel**>

<**fullName**>prey</**fullName**>

</**names**>

<**xref**>

<**primaryRef db="psi-mi" dbAc="MI:0488" id="MI:0498" refType="identity" refTypeAc="MI:0356"**/>

<**secondaryRef db="intact" dbAc="MI:0469" id="EBI-58" refType="identity" refTypeAc="MI:0356"**/>

<**secondaryRef db="pubmed" dbAc="MI:0446" id="14755292" refType="primary-reference" refTypeAc="MI:0358"**/>

</**xref**>

</**experimentalRole**>

</**experimentalRoleList**>

<**featureList**>

<**feature id="9"**>

<**names**>

<**shortLabel**>arg689cys</**shortLabel**>

<**fullName**>R689C</**fullName**>

</**names**>

<**xref**>

<**primaryRef db="intact" dbAc="MI:0469" id="EBI-11167329" refType="identity" refTypeAc="MI:0356"**/>

</**xref**>

<**featureType**>

<**names**>

<**shortLabel**>variant</**shortLabel**>

<**fullName**>variant</**fullName**>

</**names**>

<**xref**>

<**primaryRef db="psi-mi" dbAc="MI:0488" id="MI:1241" refType="identity" refTypeAc="MI:0356"**/>

<**secondaryRef db="intact" dbAc="MI:0469" id="EBI-6354006" refType="identity" refTypeAc="MI:0356"**/>

</**xref**>

</**featureType**>

<**featureRangeList**>

<**featureRange**>

<**startStatus**>

<**names**>

<**shortLabel**>certain</**shortLabel**>

<**fullName**>certain sequence position</**fullName**>

<**alias type="synonym" typeAc="MI:1041"**>certain</**alias**>

</**names**>

<**xref**>

<**primaryRef db="psi-mi" dbAc="MI:0488" id="MI:0335" refType="identity" refTypeAc="MI:0356"**/>

<**secondaryRef db="intact" dbAc="MI:0469" id="EBI-540564" refType="identity" refTypeAc="MI:0356"**/>

<**secondaryRef db="pubmed" dbAc="MI:0446" id="14755292" refType="primary-reference" refTypeAc="MI:0358"**/>

</**xref**>

</**startStatus**>

<**begin position="689"**/>

<**endStatus**>

<**names**>

<**shortLabel**>certain</**shortLabel**>

<**fullName**>certain sequence position</**fullName**>

<**alias type="synonym" typeAc="MI:1041"**>certain</**alias**>

</**names**>

<**xref**>

<**primaryRef db="psi-mi" dbAc="MI:0488" id="MI:0335" refType="identity" refTypeAc="MI:0356"**/>

<**secondaryRef db="intact" dbAc="MI:0469" id="EBI-540564" refType="identity" refTypeAc="MI:0356"**/>

<**secondaryRef db="pubmed" dbAc="MI:0446" id="14755292" refType="primary-reference" refTypeAc="MI:0358"**/>

</**xref**>

</**endStatus**>

<**end position="689"**/>

<**resultingSequence**>

<**originalSequence**>R</**originalSequence**>

<**newSequence**>C</**newSequence**>

</**resultingSequence**>

</**featureRange**>

</**featureRangeList**>

<**parameterList**>

<**parameter term="kd" termAc="MI:0646" unit="molar" unitAc="MI:0648" base="10" exponent="-7" factor="1.39"**/>

</**parameterList**>

</**feature**>

<**feature id="10"**>

<**names**>

<**shortLabel**>cys672ser</**shortLabel**>

<**fullName**>MSP1wt</**fullName**>

</**names**>

<**xref**>

<**primaryRef db="intact" dbAc="MI:0469" id="EBI-11167299" refType="identity" refTypeAc="MI:0356"**/>

</**xref**>

<**featureType**>

<**names**>

<**shortLabel**>mutation</**shortLabel**>

<**fullName**>mutation</**fullName**>

</**names**>

<**xref**>

<**primaryRef db="psi-mi" dbAc="MI:0488" id="MI:0118" refType="identity" refTypeAc="MI:0356"**/>

<**secondaryRef db="intact" dbAc="MI:0469" id="EBI-456558" refType="identity" refTypeAc="MI:0356"**/>

<**secondaryRef db="pubmed" dbAc="MI:0446" id="14755292" refType="primary-reference" refTypeAc="MI:0358"**/>

</**xref**>

</**featureType**>

<**featureRangeList**>

<**featureRange**>

<**startStatus**>

<**names**>

<**shortLabel**>certain</**shortLabel**>

<**fullName**>certain sequence position</**fullName**>

<**alias type="synonym" typeAc="MI:1041"**>certain</**alias**>

</**names**>

<**xref**>

<**primaryRef db="psi-mi" dbAc="MI:0488" id="MI:0335" refType="identity" refTypeAc="MI:0356"**/>

<**secondaryRef db="intact" dbAc="MI:0469" id="EBI-540564" refType="identity" refTypeAc="MI:0356"**/>

<**secondaryRef db="pubmed" dbAc="MI:0446" id="14755292" refType="primary-reference" refTypeAc="MI:0358"**/>

</**xref**>

</**startStatus**>

<**begin position="672"**/>

<**endStatus**>

<**names**>

<**shortLabel**>certain</**shortLabel**>

<**fullName**>certain sequence position</**fullName**>

<**alias type="synonym" typeAc="MI:1041"**>certain</**alias**>

</**names**>

<**xref**>

<**primaryRef db="psi-mi" dbAc="MI:0488" id="MI:0335" refType="identity" refTypeAc="MI:0356"**/>

<**secondaryRef db="intact" dbAc="MI:0469" id="EBI-540564" refType="identity" refTypeAc="MI:0356"**/>

<**secondaryRef db="pubmed" dbAc="MI:0446" id="14755292" refType="primary-reference" refTypeAc="MI:0358"**/>

</**xref**>

</**endStatus**>

<**end position="672"**/>

<**resultingSequence**>

<**originalSequence**>C</**originalSequence**>

<**newSequence**>S</**newSequence**>

</**resultingSequence**>

</**featureRange**>

</**featureRangeList**>

<**attributeList**>

<**attribute name="remark-internal"**>

Wed Jul 06 16:17:27 BST 2016 Sequence change details about this feature cannot be ascertained or do not fit with the current version of the referenced protein, so they have been deleted as a result of our quality control procedures. The original label was 'cys672ser'</**attribute**>

<**attribute name="no-mutation-export"**/>

</**attributeList**>

</**feature**>

<**feature id="11"**>

<**names**>

<**shortLabel**>region</**shortLabel**>

<**fullName**>MSP1wt</**fullName**>

</**names**>

<**xref**>

<**primaryRef db="intact" dbAc="MI:0469" id="EBI-11167301" refType="identity" refTypeAc="MI:0356"**/>

</**xref**>

<**featureType**>

<**names**>

<**shortLabel**>sufficient to bind</**shortLabel**>

<**fullName**>sufficient binding region</**fullName**>

<**alias type="synonym" typeAc="MI:1041"**>sufficient to bind</**alias**>

</**names**>

<**xref**>

<**primaryRef db="psi-mi" dbAc="MI:0488" id="MI:0442" refType="identity" refTypeAc="MI:0356"**/>

<**secondaryRef db="intact" dbAc="MI:0469" id="EBI-608899" refType="identity" refTypeAc="MI:0356"**/>

<**secondaryRef db="pubmed" dbAc="MI:0446" id="14755292" refType="primary-reference" refTypeAc="MI:0358"**/>

</**xref**>

</**featureType**>

<**featureRangeList**>

<**featureRange**>

<**startStatus**>

<**names**>

<**shortLabel**>certain</**shortLabel**>

<**fullName**>certain sequence position</**fullName**>

<**alias type="synonym" typeAc="MI:1041"**>certain</**alias**>

</**names**>

<**xref**>

<**primaryRef db="psi-mi" dbAc="MI:0488" id="MI:0335" refType="identity" refTypeAc="MI:0356"**/>

<**secondaryRef db="intact" dbAc="MI:0469" id="EBI-540564" refType="identity" refTypeAc="MI:0356"**/>

<**secondaryRef db="pubmed" dbAc="MI:0446" id="14755292" refType="primary-reference" refTypeAc="MI:0358"**/>

</**xref**>

</**startStatus**>

<**begin position="465"**/>

<**endStatus**>

<**names**>

<**shortLabel**>certain</**shortLabel**>

<**fullName**>certain sequence position</**fullName**>

<**alias type="synonym" typeAc="MI:1041"**>certain</**alias**>

</**names**>

<**xref**>

<**primaryRef db="psi-mi" dbAc="MI:0488" id="MI:0335" refType="identity" refTypeAc="MI:0356"**/>

<**secondaryRef db="intact" dbAc="MI:0469" id="EBI-540564" refType="identity" refTypeAc="MI:0356"**/>

<**secondaryRef db="pubmed" dbAc="MI:0446" id="14755292" refType="primary-reference" refTypeAc="MI:0358"**/>

</**xref**>

</**endStatus**>

<**end position="711"**/>

</**featureRange**>

</**featureRangeList**>

</**feature**>

<**feature id="12"**>

<**names**>

<**shortLabel**>region</**shortLabel**>

<**fullName**>MSP</**fullName**>

</**names**>

<**xref**>

<**primaryRef db="intact" dbAc="MI:0469" id="EBI-10958329" refType="identity" refTypeAc="MI:0356"**/>

</**xref**>

<**featureType**>

<**names**>

<**shortLabel**>his tag</**shortLabel**>

<**fullName**>his tag</**fullName**>

<**alias type="go synonym" typeAc="MI:0303"**>Hexa-His-tag</**alias**>

<**alias type="go synonym" typeAc="MI:0303"**>6-His-tag</**alias**>

<**alias type="go synonym" typeAc="MI:0303"**>Histidine-tag</**alias**>

</**names**>

<**xref**>

<**primaryRef db="psi-mi" dbAc="MI:0488" id="MI:0521" refType="identity" refTypeAc="MI:0356"**/>

<**secondaryRef db="intact" dbAc="MI:0469" id="EBI-456516" refType="identity" refTypeAc="MI:0356"**/>

<**secondaryRef db="pubmed" dbAc="MI:0446" id="14755292" refType="primary-reference" refTypeAc="MI:0358"**/>

</**xref**>

</**featureType**>

<**featureRangeList**>

<**featureRange**>

<**startStatus**>

<**names**>

<**shortLabel**>c-term range</**shortLabel**>

<**fullName**>C-terminal range</**fullName**>

</**names**>

<**xref**>

<**primaryRef db="psi-mi" dbAc="MI:0488" id="MI:1039" refType="identity" refTypeAc="MI:0356"**/>

<**secondaryRef db="intact" dbAc="MI:0469" id="EBI-2929799" refType="identity" refTypeAc="MI:0356"**/>

<**secondaryRef db="pubmed" dbAc="MI:0446" id="14760721" refType="primary-reference" refTypeAc="MI:0358"**/>

</**xref**>

</**startStatus**>

<**endStatus**>

<**names**>

<**shortLabel**>c-term range</**shortLabel**>

<**fullName**>C-terminal range</**fullName**>

</**names**>

<**xref**>

<**primaryRef db="psi-mi" dbAc="MI:0488" id="MI:1039" refType="identity" refTypeAc="MI:0356"**/>

<**secondaryRef db="intact" dbAc="MI:0469" id="EBI-2929799" refType="identity" refTypeAc="MI:0356"**/>

<**secondaryRef db="pubmed" dbAc="MI:0446" id="14760721" refType="primary-reference" refTypeAc="MI:0358"**/>

</**xref**>

</**endStatus**>

</**featureRange**>

</**featureRangeList**>

</**feature**>

</**featureList**>

<**hostOrganismList**>

<**hostOrganism ncbiTaxId="7227"**>

<**names**>

<**shortLabel**>drome-schneider_2</**shortLabel**>

<**fullName**>Drosophila melanogaster late embryo cells</**fullName**>

</**names**>

<**cellType**>

<**names**>

<**shortLabel**>schneider_2</**shortLabel**>

<**fullName**>Established from the late embryo of Drosophila melanogaster</**fullName**>

</**names**>

<**xref**>

<**primaryRef db="cabri" dbAc="MI:0246" id="ACC 130" refType="identity" refTypeAc="MI:0356"**/>

<**secondaryRef db="intact" dbAc="MI:0469" id="IA:0067" refType="identity" refTypeAc="MI:0356"**/>

<**secondaryRef db="mint" dbAc="MI:0471" id="MINT-2569257" refType="identity" refTypeAc="MI:0356"**/>

<**secondaryRef db="intact" dbAc="MI:0469" id="EBI-307758" refType="identity" refTypeAc="MI:0356"**/>

</**xref**>

<**attributeList**>

<**attribute name="comment" nameAc="MI:0612"**>

Mixture of diploid and tetraploid. Originally had some XY cells now predominantly XX.

</**attribute**>

</**attributeList**>

</**cellType**>

</**hostOrganism**>

</**hostOrganismList**>

</**participant**>

</**participantList**>

<**inferredInteractionList**>

<**inferredInteraction**>

<**participant**>

<**participantFeatureRef**>7</**participantFeatureRef**>

</**participant**>

<**participant**>

<**participantFeatureRef**>11</**participantFeatureRef**>

</**participant**>

</**inferredInteraction**>

</**inferredInteractionList**>

<**interactionType**>

<**names**>

<**shortLabel**>direct interaction</**shortLabel**>

<**fullName**>direct interaction</**fullName**>

</**names**>

<**xref**>

<**primaryRef db="psi-mi" dbAc="MI:0488" id="MI:0407" refType="identity" refTypeAc="MI:0356"**/>

<**secondaryRef db="intact" dbAc="MI:0469" id="EBI-608833" refType="identity" refTypeAc="MI:0356"**/>

<**secondaryRef db="pubmed" dbAc="MI:0446" id="14755292" refType="primary-reference" refTypeAc="MI:0358"**/>

</**xref**>

</**interactionType**>

<**parameterList**>

<**parameter term="kd" termAc="MI:0646" unit="molar" unitAc="MI:0648" base="10" exponent="-8" factor="1.64"**/>

</**parameterList**>

<**attributeList**>

<**attribute name="figure legend" nameAc="MI:0599"**>Fig. 2, Fig.3, Table 3</**attribute**>

<**attribute name="comment" nameAc="MI:0612"**>Kd reported are based on the equilibrium model</**attribute**>

<**attribute name="comment" nameAc="MI:0612"**>arg689cys_ S-RON, Kd: 139 (10e-9)</**attribute**>

</**attributeList**>

</**interaction**>

</**interactionList**>

</**entry**>

</**entrySet**>
