# Supplementary material for: Encompassing new use cases - level 3.0 of the HUPO-PSI format for molecular interactions
Source: BMC Bioinformatics. 2018 Apr 11;19:134. doi: 10.1186/s12859-018-2118-1 (PMC5896046; doi:10.1186/s12859-018-2118-1)
Supplement: Supplementary file 6 — Representation of variable conditions (dynamic interactions) in an experiment (use case 1.3f). (https://github.com/HUPO-PSI/miXML/blob/master/3.0/pub/Appendix%207.docx). (DOCX 43 kb) [file 12859_2018_2118_MOESM6_ESM.docx]

**Variable conditions**

The ability to describe dynamic interactions, i.e. interactions measured under identical experimental conditions other than the change of one or more specific variables

PMID:22000020

A time course protein interaction patterns following sendai virus infection of a cell line

Expt EBI-5237718

</**participantIdentificationMethod**>

<**variableParameterList**>

<**variableParameter**>

<**description**>Time after Sendai viral infection (hours).</**description**>

<**variableValueList**>

<**variableValue id="2" order="1"**>

<**value**>2</**value**>

</**variableValue**>

<**variableValue id="3" order="2"**>

<**value**>6</**value**>

</**variableValue**>

<**variableValue id="4" order="3"**>

<**value**>12</**value**>

</**variableValue**>

</**variableValueList**>

</**variableParameter**>

</**variableParameterList**>

File:

*<?***xml version='1.0' encoding='UTF-8'***?>*

<**entrySet xmlns:xsi="http://www.w3.org/2001/XMLSchema-instance" xmlns="http://psi.hupo.org/mi/mif300"**

**xsi:schemaLocation="http://psi.hupo.org/mi/mif300 https://raw.githubusercontent.com/HUPO-PSI/miXML/master/3.0/src/MIF300.xsd"**

**level="3" version="0" minorVersion="0"**>

<**entry**>

<**source releaseDate="2017-05-18"**>

<**names**>

<**shortLabel**>IntAct</**shortLabel**>

<**fullName**>European Bioinformatics Institute</**fullName**>

<**alias type="synonym" typeAc="MI:1041"**>IntAct</**alias**>

</**names**>

<**bibref**>

<**xref**>

<**primaryRef db="pubmed" dbAc="MI:0446" id="14681455" refType="primary-reference" refTypeAc="MI:0358"**/>

</**xref**>

</**bibref**>

<**xref**>

<**primaryRef db="psi-mi" dbAc="MI:0488" id="MI:0469" refType="identity" refTypeAc="MI:0356"**/>

<**secondaryRef db="intact" dbAc="MI:0469" id="EBI-10" refType="identity" refTypeAc="MI:0356"**/>

<**secondaryRef db="pubmed" dbAc="MI:0446" id="14681455" refType="primary-reference" refTypeAc="MI:0358"**/>

<**secondaryRef db="pubmed" dbAc="MI:0446" id="22121220" refType="method reference" refTypeAc="MI:0357"**/>

<**secondaryRef db="pubmed" dbAc="MI:0446" id="19850723" refType="method reference" refTypeAc="MI:0357"**/>

</**xref**>

<**attributeList**>

<**attribute name="url" nameAc="MI:0614"**>http://www.ebi.ac.uk/</**attribute**>

<**attribute name="search-url" nameAc="MI:0615"**>http://www.ebi.ac.uk/intact/query/${ac}</**attribute**>

<**attribute name="id-validation-regexp" nameAc="MI:0628"**>EBI-[0-9]+|IA:[0-9]+</**attribute**>

<**attribute name="definition"**>

INTerAction database (IntAct) provides an open source database and toolkit for the storage, presentation and analysis of molecular interactions.

</**attribute**>

<**attribute name="url" nameAc="MI:0614"**>http://www.ebi.ac.uk/intact</**attribute**>

<**attribute name="postaladdress"**>

European Bioinformatics Institute; Wellcome Trust Genome Campus; Hinxton, Cambridge; CB10 1SD; United Kingdom

</**attribute**>

<**attribute name="url" nameAc="MI:0614"**>http://www.ebi.ac.uk/intact/</**attribute**>

</**attributeList**>

</**source**>

<**experimentList**>

<**experimentDescription id="1"**>

<**names**>

<**fullName**>Activation of STAT6 by STING Is Critical for Antiviral Innate Immunity.</**fullName**>

</**names**>

<**bibref**>

<**xref**>

<**primaryRef db="pubmed" dbAc="MI:0446" id="22000020" refType="primary-reference" refTypeAc="MI:0358"**/>

<**secondaryRef db="intact" dbAc="MI:0469" id="EBI-5237558" refType="identity" refTypeAc="MI:0356"**/>

<**secondaryRef db="imex" dbAc="MI:0670" id="IM-16625" refType="imex-primary" refTypeAc="MI:0662"**/>

</**xref**>

<**attributeList**>

<**attribute name="publication title" nameAc="MI:1091"**>Activation of STAT6 by STING Is Critical for Antiviral Innate Immunity.</**attribute**>

<**attribute name="journal" nameAc="MI:0885"**>Cell (0092-8674)</**attribute**>

<**attribute name="publication year" nameAc="MI:0886"**>2011</**attribute**>

<**attribute name="curation depth" nameAc="MI:0955"**>imex curation</**attribute**>

<**attribute name="imex curation" nameAc="MI:0959"**/>

<**attribute name="author-list" nameAc="MI:0636"**>

Chen H., Sun H., You F., Sun W., Zhou X., Chen L., Yang J., Wang Y., Tang H., Guan Y., Xia W., Gu J., Ishikawa H., Gutman D., Barber G., Qin Z., Jiang Z.

</**attribute**>

<**attribute name="contact-email" nameAc="MI:0634"**>jiangzf@pku.edu.cn</**attribute**>

<**attribute name="dataset" nameAc="MI:0875"**>Apoptosis - Interactions involving proteins with a function related to apoptosis</**attribute**>

<**attribute name="full coverage" nameAc="MI:0957"**>Only protein-protein interactions</**attribute**>

<**attribute name="imex curation" nameAc="MI:0959"**/>

<**attribute name="author-announcement"**>14-Mar-2012: Contacted by JYOTI.</**attribute**>

<**attribute name="library-used" nameAc="MI:0672"**>Human leukocyte cDNA</**attribute**>

</**attributeList**>

</**bibref**>

<**xref**>

<**primaryRef db="pubmed" dbAc="MI:0446" id="22000020" refType="primary-reference" refTypeAc="MI:0358"**/>

<**secondaryRef db="imex" dbAc="MI:0670" id="IM-16625" refType="imex-primary" refTypeAc="MI:0662"**/>

</**xref**>

<**hostOrganismList**>

<**hostOrganism ncbiTaxId="9606"**>

<**names**>

<**shortLabel**>human-293</**shortLabel**>

<**fullName**>Homo sapiens transformed primary embryonal kidney cells</**fullName**>

</**names**>

<**cellType**>

<**names**>

<**shortLabel**>293</**shortLabel**>

<**fullName**>Transformed human primary embryonal kidney cells.</**fullName**>

</**names**>

<**xref**>

<**primaryRef db="cabri" dbAc="MI:0246" id="ACC 305" refType="identity" refTypeAc="MI:0356"**/>

<**secondaryRef db="intact" dbAc="MI:0469" id="IA:0058" refType="identity" refTypeAc="MI:0356"**/>

<**secondaryRef db="mint" dbAc="MI:0471" id="MINT-1782570" refType="identity" refTypeAc="MI:0356"**/>

<**secondaryRef db="intact" dbAc="MI:0469" id="EBI-307626" refType="identity" refTypeAc="MI:0356"**/>

</**xref**>

<**attributeList**>

<**attribute name="comment" nameAc="MI:0612"**>The Cell type named 293 is synonymous with HEK-293.</**attribute**>

</**attributeList**>

</**cellType**>

</**hostOrganism**>

</**hostOrganismList**>

<**interactionDetectionMethod**>

<**names**>

<**shortLabel**>anti tag coip</**shortLabel**>

<**fullName**>anti tag coimmunoprecipitation</**fullName**>

</**names**>

<**xref**>

<**primaryRef db="psi-mi" dbAc="MI:0488" id="MI:0007" refType="identity" refTypeAc="MI:0356"**/>

<**secondaryRef db="intact" dbAc="MI:0469" id="EBI-90" refType="identity" refTypeAc="MI:0356"**/>

<**secondaryRef db="pubmed" dbAc="MI:0446" id="7708014" refType="primary-reference" refTypeAc="MI:0358"**/>

</**xref**>

</**interactionDetectionMethod**>

<**participantIdentificationMethod**>

<**names**>

<**shortLabel**>western blot</**shortLabel**>

<**fullName**>western blot</**fullName**>

<**alias type="go synonym" typeAc="MI:0303"**>Immuno blot</**alias**>

</**names**>

<**xref**>

<**primaryRef db="psi-mi" dbAc="MI:0488" id="MI:0113" refType="identity" refTypeAc="MI:0356"**/>

<**secondaryRef db="intact" dbAc="MI:0469" id="EBI-973" refType="identity" refTypeAc="MI:0356"**/>

<**secondaryRef db="pubmed" dbAc="MI:0446" id="14755292" refType="primary-reference" refTypeAc="MI:0358"**/>

</**xref**>

</**participantIdentificationMethod**>

<**variableParameterList**>

<**variableParameter**>

<**description**>Time after Sendai viral infection (hours).</**description**>

<**variableValueList**>

<**variableValue id="2" order="1"**>

<**value**>2</**value**>

</**variableValue**>

<**variableValue id="3" order="2"**>

<**value**>6</**value**>

</**variableValue**>

<**variableValue id="4" order="3"**>

<**value**>12</**value**>

</**variableValue**>

</**variableValueList**>

</**variableParameter**>

</**variableParameterList**>

<**attributeList**>

<**attribute name="contact-email" nameAc="MI:0634"**>jiangzf@pku.edu.cn</**attribute**>

<**attribute name="author-list" nameAc="MI:0636"**>

Chen H., Sun H., You F., Sun W., Zhou X., Chen L., Yang J., Wang Y., Tang H., Guan Y., Xia W., Gu J., Ishikawa H., Gutman D., Barber G., Qin Z., Jiang Z.

</**attribute**>

<**attribute name="journal" nameAc="MI:0885"**>Cell (0092-8674)</**attribute**>

<**attribute name="publication year" nameAc="MI:0886"**>2011</**attribute**>

<**attribute name="curation depth" nameAc="MI:0955"**>imex curation</**attribute**>

<**attribute name="variable"**>Time after Sendai viral infection (hours).</**attribute**>

<**attribute name="accepted"**>Accepted 2011-DEC-15 by JYOTI</**attribute**>

<**attribute name="dataset" nameAc="MI:0875"**>Apoptosis - Interactions involving proteins with a function related to apoptosis</**attribute**>

<**attribute name="full coverage" nameAc="MI:0957"**>Only protein-protein interactions</**attribute**>

<**attribute name="imex curation" nameAc="MI:0959"**/>

<**attribute name="author-announcement"**>14-Mar-2012: Contacted by JYOTI.</**attribute**>

</**attributeList**>

</**experimentDescription**>

</**experimentList**>

<**interactorList**>

<**interactor id="5"**>

<**names**>

<**shortLabel**>mavs_human</**shortLabel**>

<**fullName**>Mitochondrial antiviral-signaling protein</**fullName**>

<**alias type="gene name synonym" typeAc="MI:0302"**>Interferon beta promoter stimulator protein 1</**alias**>

<**alias type="gene name synonym" typeAc="MI:0302"**>Virus-induced-signaling adapter</**alias**>

<**alias type="gene name synonym" typeAc="MI:0302"**>CARD adapter inducing interferon beta</**alias**>

<**alias type="gene name synonym" typeAc="MI:0302"**>Putative NF-kappa-B-activating protein 031N</**alias**>

<**alias type="gene name" typeAc="MI:0301"**>MAVS</**alias**>

<**alias type="gene name synonym" typeAc="MI:0302"**>IPS1</**alias**>

<**alias type="gene name synonym" typeAc="MI:0302"**>KIAA1271</**alias**>

<**alias type="gene name synonym" typeAc="MI:0302"**>VISA</**alias**>

</**names**>

<**xref**>

<**primaryRef db="uniprotkb" dbAc="MI:0486" id="Q7Z434" version="SP_42" refType="identity" refTypeAc="MI:0356"**/>

<**secondaryRef db="uniprotkb" dbAc="MI:0486" id="Q3I0Y2" version="SP_42" refType="secondary-ac" refTypeAc="MI:0360"**/>

<**secondaryRef db="uniprotkb" dbAc="MI:0486" id="Q5T7I6" version="SP_42" refType="secondary-ac" refTypeAc="MI:0360"**/>

<**secondaryRef db="uniprotkb" dbAc="MI:0486" id="Q86VY7" version="SP_42" refType="secondary-ac" refTypeAc="MI:0360"**/>

<**secondaryRef db="uniprotkb" dbAc="MI:0486" id="Q9H1H3" version="SP_42" refType="secondary-ac" refTypeAc="MI:0360"**/>

<**secondaryRef db="uniprotkb" dbAc="MI:0486" id="Q9H4Y1" version="SP_42" refType="secondary-ac" refTypeAc="MI:0360"**/>

<**secondaryRef db="uniprotkb" dbAc="MI:0486" id="Q9H8D3" version="SP_42" refType="secondary-ac" refTypeAc="MI:0360"**/>

<**secondaryRef db="uniprotkb" dbAc="MI:0486" id="Q9ULE9" version="SP_42" refType="secondary-ac" refTypeAc="MI:0360"**/>

<**secondaryRef db="uniprotkb" dbAc="MI:0486" id="Q2HWT5" version="SP_56" refType="secondary-ac" refTypeAc="MI:0360"**/>

<**secondaryRef db="uniprotkb" dbAc="MI:0486" id="A8K6X0" version="SP_99" refType="secondary-ac" refTypeAc="MI:0360"**/>

<**secondaryRef db="uniprotkb" dbAc="MI:0486" id="F5H6C8" version="SP_99" refType="secondary-ac" refTypeAc="MI:0360"**/>

<**secondaryRef db="uniprotkb" dbAc="MI:0486" id="B2BD33" version="SP_104" refType="secondary-ac" refTypeAc="MI:0360"**/>

<**secondaryRef db="uniprotkb" dbAc="MI:0486" id="B2BD34" version="SP_104" refType="secondary-ac" refTypeAc="MI:0360"**/>

<**secondaryRef db="uniprotkb" dbAc="MI:0486" id="M1P2Z0" version="SP_119" refType="secondary-ac" refTypeAc="MI:0360"**/>

<**secondaryRef db="intact" dbAc="MI:0469" id="EBI-995373" refType="identity" refTypeAc="MI:0356"**/>

<**secondaryRef db="go" dbAc="MI:0448" id="GO:0005739"**/>

<**secondaryRef db="refseq" dbAc="MI:0481" id="NP_001193420.1"**/>

<**secondaryRef db="rcsb pdb" dbAc="MI:0460" id="3J6J"**/>

<**secondaryRef db="ensembl" dbAc="MI:0476" id="ENST00000416600"**/>

<**secondaryRef db="ensembl" dbAc="MI:0476" id="ENST00000428216"**/>

<**secondaryRef db="go" dbAc="MI:0448" id="GO:0016021"**/>

<**secondaryRef db="go" dbAc="MI:0448" id="GO:0005741"**/>

<**secondaryRef db="go" dbAc="MI:0448" id="GO:0004871"**/>

<**secondaryRef db="go" dbAc="MI:0448" id="GO:0002218"**/>

<**secondaryRef db="go" dbAc="MI:0448" id="GO:0071360"**/>

<**secondaryRef db="go" dbAc="MI:0448" id="GO:0042742"**/>

<**secondaryRef db="go" dbAc="MI:0448" id="GO:0045087"**/>

<**secondaryRef db="go" dbAc="MI:0448" id="GO:0032480"**/>

<**secondaryRef db="go" dbAc="MI:0448" id="GO:0071651"**/>

<**secondaryRef db="go" dbAc="MI:0448" id="GO:0002230"**/>

<**secondaryRef db="go" dbAc="MI:0448" id="GO:0043123"**/>

<**secondaryRef db="go" dbAc="MI:0448" id="GO:0032727"**/>

<**secondaryRef db="go" dbAc="MI:0448" id="GO:0032728"**/>

<**secondaryRef db="go" dbAc="MI:0448" id="GO:0032757"**/>

<**secondaryRef db="go" dbAc="MI:0448" id="GO:0071660"**/>

<**secondaryRef db="go" dbAc="MI:0448" id="GO:0033160"**/>

<**secondaryRef db="go" dbAc="MI:0448" id="GO:0001934"**/>

<**secondaryRef db="go" dbAc="MI:0448" id="GO:0051091"**/>

<**secondaryRef db="go" dbAc="MI:0448" id="GO:0042993"**/>

<**secondaryRef db="go" dbAc="MI:0448" id="GO:0045944"**/>

<**secondaryRef db="go" dbAc="MI:0448" id="GO:0032760"**/>

<**secondaryRef db="go" dbAc="MI:0448" id="GO:0060340"**/>

<**secondaryRef db="refseq" dbAc="MI:0481" id="NP_065797.2"**/>

<**secondaryRef db="rcsb pdb" dbAc="MI:0460" id="2VGQ"**/>

<**secondaryRef db="go" dbAc="MI:0448" id="GO:0005778"**/>

<**secondaryRef db="go" dbAc="MI:0448" id="GO:0045071"**/>

<**secondaryRef db="go" dbAc="MI:0448" id="GO:0051607"**/>

<**secondaryRef db="go" dbAc="MI:0448" id="GO:1900063"**/>

<**secondaryRef db="interpro" dbAc="MI:0449" id="IPR026148"**/>

<**secondaryRef db="go" dbAc="MI:0448" id="GO:0039529"**/>

<**secondaryRef db="go" dbAc="MI:0448" id="GO:0007165"**/>

<**secondaryRef db="go" dbAc="MI:0448" id="GO:0019901"**/>

<**secondaryRef db="go" dbAc="MI:0448" id="GO:0031966"**/>

<**secondaryRef db="go" dbAc="MI:0448" id="GO:0050700"**/>

<**secondaryRef db="rcsb pdb" dbAc="MI:0460" id="3J6C"**/>

<**secondaryRef db="rcsb pdb" dbAc="MI:0460" id="4P4H"**/>

<**secondaryRef db="ensembl" dbAc="MI:0476" id="ENSG00000088888"**/>

<**secondaryRef db="ensembl" dbAc="MI:0476" id="ENSP00000401980"**/>

<**secondaryRef db="ensembl" dbAc="MI:0476" id="ENSP00000413749"**/>

<**secondaryRef db="rcsb pdb" dbAc="MI:0460" id="5JEK"**/>

<**secondaryRef db="reactome" dbAc="MI:0467" id="R-HSA-168928"**/>

<**secondaryRef db="reactome" dbAc="MI:0467" id="R-HSA-918233"**/>

<**secondaryRef db="reactome" dbAc="MI:0467" id="R-HSA-933541"**/>

<**secondaryRef db="reactome" dbAc="MI:0467" id="R-HSA-933542"**/>

<**secondaryRef db="reactome" dbAc="MI:0467" id="R-HSA-933543"**/>

<**secondaryRef db="reactome" dbAc="MI:0467" id="R-HSA-936440"**/>

<**secondaryRef db="rcsb pdb" dbAc="MI:0460" id="3RC5"**/>

<**secondaryRef db="interpro" dbAc="MI:0449" id="IPR031964"**/>

<**secondaryRef db="rcsb pdb" dbAc="MI:0460" id="2MS7"**/>

<**secondaryRef db="rcsb pdb" dbAc="MI:0460" id="2MS8"**/>

<**secondaryRef db="rcsb pdb" dbAc="MI:0460" id="4Z8M"**/>

<**secondaryRef db="go" dbAc="MI:0448" id="GO:0016579"**/>

<**secondaryRef db="interpro" dbAc="MI:0449" id="IPR011029"**/>

<**secondaryRef db="reactome" dbAc="MI:0467" id="R-HSA-5689896"**/>

</**xref**>

<**interactorType**>

<**names**>

<**shortLabel**>protein</**shortLabel**>

<**fullName**>protein</**fullName**>

</**names**>

<**xref**>

<**primaryRef db="psi-mi" dbAc="MI:0488" id="MI:0326" refType="identity" refTypeAc="MI:0356"**/>

<**secondaryRef db="intact" dbAc="MI:0469" id="EBI-619654" refType="identity" refTypeAc="MI:0356"**/>

<**secondaryRef db="pubmed" dbAc="MI:0446" id="14755292" refType="primary-reference" refTypeAc="MI:0358"**/>

<**secondaryRef db="so" dbAc="MI:0601" id="SO:0000358" refType="see-also" refTypeAc="MI:0361"**/>

</**xref**>

</**interactorType**>

<**organism ncbiTaxId="9606"**>

<**names**>

<**shortLabel**>human</**shortLabel**>

<**fullName**>Homo sapiens</**fullName**>

<**alias type="synonym" typeAc="MI:1041"**>Human</**alias**>

</**names**>

</**organism**>

<**sequence**>

MPFAEDKTYKYICRNFSNFCNVDVVEILPYLPCLTARDQDRLRATCTLSGNRDTLWHLFNTLQRRPGWVEYFIAALRGCELVDLADEVASVYQSYQPRTSDRPPDPLEPPSLPAERPGPPTPAAAHSIPYNSCREKEPSYPMPVQETQAPESPGENSEQALQTLSPRAIPRNPDGGPLESSSDLAALSPLTSSGHQEQDTELGSTHTAGATSSLTPSRGPVSPSVSFQPLARSTPRASRLPGPTGSVVSTGTSFSSSSPGLASAGAAEGKQGAESDQAEPIICSSGAEAPANSLPSKVPTTLMPVNTVALKVPANPASVSTVPSKLPTSSKPPGAVPSNALTNPAPSKLPINSTRAGMVPSKVPTSMVLTKVSASTVPTDGSSRNEETPAAPTPAGATGGSSAWLDSSSENRGLGSELSKPGVLASQVDSPFSGCFEDLAISASTSLGMGPCHGPEENEYKSEGTFGIHVAENPSIQLLEGNPGPPADPDGGPRPQADRKFQEREVPCHRPSPGALWLQVAVTGVLVVTLLVVLYRRRLH

</**sequence**>

<**attributeList**>

<**attribute name="crc64"**>0E23E3E115941EE8</**attribute**>

</**attributeList**>

</**interactor**>

<**interactor id="6"**>

<**names**>

<**shortLabel**>stat6_human</**shortLabel**>

<**fullName**>Signal transducer and activator of transcription 6</**fullName**>

<**alias type="gene name synonym" typeAc="MI:0302"**>IL-4 Stat</**alias**>

<**alias type="gene name" typeAc="MI:0301"**>STAT6</**alias**>

</**names**>

<**xref**>

<**primaryRef db="uniprotkb" dbAc="MI:0486" id="P42226" version="SP_74" refType="identity" refTypeAc="MI:0356"**/>

<**secondaryRef db="uniprotkb" dbAc="MI:0486" id="Q5FBW5" version="SP_83" refType="secondary-ac" refTypeAc="MI:0360"**/>

<**secondaryRef db="uniprotkb" dbAc="MI:0486" id="Q71UP4" version="SP_83" refType="secondary-ac" refTypeAc="MI:0360"**/>

<**secondaryRef db="uniprotkb" dbAc="MI:0486" id="A8K316" version="SP_86" refType="secondary-ac" refTypeAc="MI:0360"**/>

<**secondaryRef db="uniprotkb" dbAc="MI:0486" id="B7ZA27" version="SP_131" refType="secondary-ac" refTypeAc="MI:0360"**/>

<**secondaryRef db="uniprotkb" dbAc="MI:0486" id="F5GXI9" version="SP_131" refType="secondary-ac" refTypeAc="MI:0360"**/>

<**secondaryRef db="intact" dbAc="MI:0469" id="EBI-1186478" refType="identity" refTypeAc="MI:0356"**/>

<**secondaryRef db="ensembl" dbAc="MI:0476" id="ENST00000300134"**/>

<**secondaryRef db="ensembl" dbAc="MI:0476" id="ENST00000454075"**/>

<**secondaryRef db="ensembl" dbAc="MI:0476" id="ENST00000537215"**/>

<**secondaryRef db="ensembl" dbAc="MI:0476" id="ENST00000538913"**/>

<**secondaryRef db="ensembl" dbAc="MI:0476" id="ENST00000543873"**/>

<**secondaryRef db="ensembl" dbAc="MI:0476" id="ENST00000556155"**/>

<**secondaryRef db="go" dbAc="MI:0448" id="GO:0003700"**/>

<**secondaryRef db="go" dbAc="MI:0448" id="GO:0004871"**/>

<**secondaryRef db="refseq" dbAc="MI:0481" id="NP_001171549.1"**/>

<**secondaryRef db="refseq" dbAc="MI:0481" id="NP_001171550.1"**/>

<**secondaryRef db="refseq" dbAc="MI:0481" id="NP_003144.3"**/>

<**secondaryRef db="interpro" dbAc="MI:0449" id="IPR011992"**/>

<**secondaryRef db="interpro" dbAc="MI:0449" id="IPR008967"**/>

<**secondaryRef db="interpro" dbAc="MI:0449" id="IPR000980"**/>

<**secondaryRef db="interpro" dbAc="MI:0449" id="IPR013800"**/>

<**secondaryRef db="interpro" dbAc="MI:0449" id="IPR015988"**/>

<**secondaryRef db="interpro" dbAc="MI:0449" id="IPR001217"**/>

<**secondaryRef db="interpro" dbAc="MI:0449" id="IPR013801"**/>

<**secondaryRef db="interpro" dbAc="MI:0449" id="IPR012345"**/>

<**secondaryRef db="interpro" dbAc="MI:0449" id="IPR013799"**/>

<**secondaryRef db="rcsb pdb" dbAc="MI:0460" id="1OJ5"**/>

<**secondaryRef db="go" dbAc="MI:0448" id="GO:0005829"**/>

<**secondaryRef db="go" dbAc="MI:0448" id="GO:0005654"**/>

<**secondaryRef db="go" dbAc="MI:0448" id="GO:0032481"**/>

<**secondaryRef db="go" dbAc="MI:0448" id="GO:0035771"**/>

<**secondaryRef db="refseq" dbAc="MI:0481" id="XP_006719638.1"**/>

<**secondaryRef db="go" dbAc="MI:0448" id="GO:0006357"**/>

<**secondaryRef db="refseq" dbAc="MI:0481" id="NP_001171551.1"**/>

<**secondaryRef db="go" dbAc="MI:0448" id="GO:0000979"**/>

<**secondaryRef db="go" dbAc="MI:0448" id="GO:0033598"**/>

<**secondaryRef db="go" dbAc="MI:0448" id="GO:0060443"**/>

<**secondaryRef db="go" dbAc="MI:0448" id="GO:0000122"**/>

<**secondaryRef db="go" dbAc="MI:0448" id="GO:0000790"**/>

<**secondaryRef db="go" dbAc="MI:0448" id="GO:0002296"**/>

<**secondaryRef db="go" dbAc="MI:0448" id="GO:0002829"**/>

<**secondaryRef db="go" dbAc="MI:0448" id="GO:0042127"**/>

<**secondaryRef db="go" dbAc="MI:0448" id="GO:0045944"**/>

<**secondaryRef db="go" dbAc="MI:0448" id="GO:0048295"**/>

<**secondaryRef db="interpro" dbAc="MI:0449" id="IPR028187"**/>

<**secondaryRef db="go" dbAc="MI:0448" id="GO:0007165"**/>

<**secondaryRef db="go" dbAc="MI:0448" id="GO:0019903"**/>

<**secondaryRef db="reactome" dbAc="MI:0467" id="R-HSA-186763"**/>

<**secondaryRef db="reactome" dbAc="MI:0467" id="R-HSA-3249367"**/>

<**secondaryRef db="ensembl" dbAc="MI:0476" id="ENSG00000166888"**/>

<**secondaryRef db="ensembl" dbAc="MI:0476" id="ENSP00000300134"**/>

<**secondaryRef db="ensembl" dbAc="MI:0476" id="ENSP00000401486"**/>

<**secondaryRef db="ensembl" dbAc="MI:0476" id="ENSP00000438451"**/>

<**secondaryRef db="ensembl" dbAc="MI:0476" id="ENSP00000444530"**/>

<**secondaryRef db="ensembl" dbAc="MI:0476" id="ENSP00000445409"**/>

<**secondaryRef db="ensembl" dbAc="MI:0476" id="ENSP00000451742"**/>

<**secondaryRef db="rcsb pdb" dbAc="MI:0460" id="4Y5U"**/>

<**secondaryRef db="rcsb pdb" dbAc="MI:0460" id="4Y5W"**/>

<**secondaryRef db="go" dbAc="MI:0448" id="GO:0045121"**/>

<**secondaryRef db="go" dbAc="MI:0448" id="GO:0070301"**/>

<**secondaryRef db="go" dbAc="MI:0448" id="GO:1902170"**/>

<**secondaryRef db="go" dbAc="MI:0448" id="GO:0001228"**/>

<**secondaryRef db="rcsb pdb" dbAc="MI:0460" id="5D39"**/>

<**secondaryRef db="go" dbAc="MI:0448" id="GO:0042802"**/>

<**secondaryRef db="go" dbAc="MI:0448" id="GO:0000977"**/>

<**secondaryRef db="go" dbAc="MI:0448" id="GO:0031965"**/>

<**secondaryRef db="reactome" dbAc="MI:0467" id="R-HSA-6785807"**/>

</**xref**>

<**interactorType**>

<**names**>

<**shortLabel**>protein</**shortLabel**>

<**fullName**>protein</**fullName**>

</**names**>

<**xref**>

<**primaryRef db="psi-mi" dbAc="MI:0488" id="MI:0326" refType="identity" refTypeAc="MI:0356"**/>

<**secondaryRef db="intact" dbAc="MI:0469" id="EBI-619654" refType="identity" refTypeAc="MI:0356"**/>

<**secondaryRef db="pubmed" dbAc="MI:0446" id="14755292" refType="primary-reference" refTypeAc="MI:0358"**/>

<**secondaryRef db="so" dbAc="MI:0601" id="SO:0000358" refType="see-also" refTypeAc="MI:0361"**/>

</**xref**>

</**interactorType**>

<**organism ncbiTaxId="9606"**>

<**names**>

<**shortLabel**>human</**shortLabel**>

<**fullName**>Homo sapiens</**fullName**>

<**alias type="synonym" typeAc="MI:1041"**>Human</**alias**>

</**names**>

</**organism**>

<**sequence**>

MSLWGLVSKMPPEKVQRLYVDFPQHLRHLLGDWLESQPWEFLVGSDAFCCNLASALLSDTVQHLQASVGEQGEGSTILQHISTLESIYQRDPLKLVATFRQILQGEKKAVMEQFRHLPMPFHWKQEELKFKTGLRRLQHRVGEIHLLREALQKGAEAGQVSLHSLIETPANGTGPSEALAMLLQETTGELEAAKALVLKRIQIWKRQQQLAGNGAPFEESLAPLQERCESLVDIYSQLQQEVGAAGGELEPKTRASLTGRLDEVLRTLVTSCFLVEKQPPQVLKTQTKFQAGVRFLLGLRFLGAPAKPPLVRADMVTEKQARELSVPQGPGAGAESTGEIINNTVPLENSIPGNCCSALFKNLLLKKIKRCERKGTESVTEEKCAVLFSASFTLGPGKLPIQLQALSLPLVVIVHGNQDNNAKATILWDNAFSEMDRVPFVVAERVPWEKMCETLNLKFMAEVGTNRGLLPEHFLFLAQKIFNDNSLSMEAFQHRSVSWSQFNKEILLGRGFTFWQWFDGVLDLTKRCLRSYWSDRLIIGFISKQYVTSLLLNEPDGTFLLRFSDSEIGGITIAHVIRGQDGSPQIENIQPFSAKDLSIRSLGDRIRDLAQLKNLYPKKPKDEAFRSHYKPEQMGKDGRGYVPATIKMTVERDQPLPTPELQMPTMVPSYDLGMAPDSSMSMQLGPDMVPQVYPPHSHSIPPYQGLSPEESVNVLSAFQEPHLQMPPSLGQMSLPFDQPHPQGLLPCQPQEHAVSSPDPLLCSDVTMVEDSCLSQPVTAFPQGTWIGEDIFPPLLPPTEQDLTKLLLEGQGESGGGSLGAQPLLQPSHYGQSGISMSHMDLRANPSW

</**sequence**>

<**attributeList**>

<**attribute name="crc64"**>F35075F1C1F2A677</**attribute**>

</**attributeList**>

</**interactor**>

<**interactor id="7"**>

<**names**>

<**shortLabel**>sting_human</**shortLabel**>

<**fullName**>Stimulator of interferon genes protein</**fullName**>

<**alias type="gene name synonym" typeAc="MI:0302"**>ERIS</**alias**>

<**alias type="gene name synonym" typeAc="MI:0302"**>Endoplasmic reticulum interferon stimulator</**alias**>

<**alias type="gene name synonym" typeAc="MI:0302"**>MITA</**alias**>

<**alias type="gene name synonym" typeAc="MI:0302"**>STING</**alias**>

<**alias type="gene name synonym" typeAc="MI:0302"**>Mediator of IRF3 activation</**alias**>

<**alias type="gene name" typeAc="MI:0301"**>TMEM173</**alias**>

<**alias type="gene name synonym" typeAc="MI:0302"**>Transmembrane protein 173</**alias**>

</**names**>

<**xref**>

<**primaryRef db="uniprotkb" dbAc="MI:0486" id="Q86WV6" refType="identity" refTypeAc="MI:0356"**/>

<**secondaryRef db="uniprotkb" dbAc="MI:0486" id="B6EB35" version="SP_53" refType="secondary-ac" refTypeAc="MI:0360"**/>

<**secondaryRef db="uniprotkb" dbAc="MI:0486" id="D6RBX0" version="SP_53" refType="secondary-ac" refTypeAc="MI:0360"**/>

<**secondaryRef db="uniprotkb" dbAc="MI:0486" id="D6RE01" version="SP_53" refType="secondary-ac" refTypeAc="MI:0360"**/>

<**secondaryRef db="uniprotkb" dbAc="MI:0486" id="D6RID9" version="SP_53" refType="secondary-ac" refTypeAc="MI:0360"**/>

<**secondaryRef db="uniprotkb" dbAc="MI:0486" id="A8K3P6" refType="secondary-ac" refTypeAc="MI:0360"**/>

<**secondaryRef db="intact" dbAc="MI:0469" id="EBI-2800345" refType="identity" refTypeAc="MI:0356"**/>

<**secondaryRef db="rcsb pdb" dbAc="MI:0460" id="4KSY"**/>

<**secondaryRef db="go" dbAc="MI:0448" id="GO:0061507"**/>

<**secondaryRef db="go" dbAc="MI:0448" id="GO:0071407"**/>

<**secondaryRef db="interpro" dbAc="MI:0449" id="IPR033952"**/>

<**secondaryRef db="reactome" dbAc="MI:0467" id="R-HSA-6798695"**/>

<**secondaryRef db="go" dbAc="MI:0448" id="GO:0005789"**/>

<**secondaryRef db="go" dbAc="MI:0448" id="GO:0016021"**/>

<**secondaryRef db="go" dbAc="MI:0448" id="GO:0005741"**/>

<**secondaryRef db="go" dbAc="MI:0448" id="GO:0048471"**/>

<**secondaryRef db="go" dbAc="MI:0448" id="GO:0005886"**/>

<**secondaryRef db="go" dbAc="MI:0448" id="GO:0002218"**/>

<**secondaryRef db="go" dbAc="MI:0448" id="GO:0006915"**/>

<**secondaryRef db="go" dbAc="MI:0448" id="GO:0071360"**/>

<**secondaryRef db="go" dbAc="MI:0448" id="GO:0051607"**/>

<**secondaryRef db="go" dbAc="MI:0448" id="GO:0045087"**/>

<**secondaryRef db="go" dbAc="MI:0448" id="GO:0032608"**/>

<**secondaryRef db="go" dbAc="MI:0448" id="GO:0002230"**/>

<**secondaryRef db="go" dbAc="MI:0448" id="GO:0032092"**/>

<**secondaryRef db="go" dbAc="MI:0448" id="GO:0033160"**/>

<**secondaryRef db="go" dbAc="MI:0448" id="GO:0042993"**/>

<**secondaryRef db="go" dbAc="MI:0448" id="GO:0045944"**/>

<**secondaryRef db="refseq" dbAc="MI:0481" id="NP_938023.1"**/>

<**secondaryRef db="ensembl" dbAc="MI:0476" id="ENSG00000184584"**/>

<**secondaryRef db="go" dbAc="MI:0448" id="GO:0032481"**/>

<**secondaryRef db="rcsb pdb" dbAc="MI:0460" id="4EF4"**/>

<**secondaryRef db="rcsb pdb" dbAc="MI:0460" id="4EF5"**/>

<**secondaryRef db="go" dbAc="MI:0448" id="GO:0035458"**/>

<**secondaryRef db="rcsb pdb" dbAc="MI:0460" id="4F5D"**/>

<**secondaryRef db="rcsb pdb" dbAc="MI:0460" id="4F5E"**/>

<**secondaryRef db="rcsb pdb" dbAc="MI:0460" id="4F5W"**/>

<**secondaryRef db="rcsb pdb" dbAc="MI:0460" id="4F5Y"**/>

<**secondaryRef db="rcsb pdb" dbAc="MI:0460" id="4F9E"**/>

<**secondaryRef db="rcsb pdb" dbAc="MI:0460" id="4F9G"**/>

<**secondaryRef db="rcsb pdb" dbAc="MI:0460" id="5JEJ"**/>

<**secondaryRef db="rcsb pdb" dbAc="MI:0460" id="4EMT"**/>

<**secondaryRef db="rcsb pdb" dbAc="MI:0460" id="4EMU"**/>

<**secondaryRef db="go" dbAc="MI:0448" id="GO:0005794"**/>

<**secondaryRef db="go" dbAc="MI:0448" id="GO:0035438"**/>

<**secondaryRef db="go" dbAc="MI:0448" id="GO:0008134"**/>

<**secondaryRef db="go" dbAc="MI:0448" id="GO:0019901"**/>

<**secondaryRef db="go" dbAc="MI:0448" id="GO:0042803"**/>

<**secondaryRef db="go" dbAc="MI:0448" id="GO:0005777"**/>

<**secondaryRef db="go" dbAc="MI:0448" id="GO:0030659"**/>

<**secondaryRef db="rcsb pdb" dbAc="MI:0460" id="4LOH"**/>

<**secondaryRef db="rcsb pdb" dbAc="MI:0460" id="4LOI"**/>

<**secondaryRef db="go" dbAc="MI:0448" id="GO:0032479"**/>

<**secondaryRef db="interpro" dbAc="MI:0449" id="IPR029158"**/>

<**secondaryRef db="ensembl" dbAc="MI:0476" id="ENST00000330794"**/>

<**secondaryRef db="ensembl" dbAc="MI:0476" id="ENSP00000331288"**/>

<**secondaryRef db="rcsb pdb" dbAc="MI:0460" id="5BQX"**/>

<**secondaryRef db="reactome" dbAc="MI:0467" id="R-HSA-1834941"**/>

<**secondaryRef db="reactome" dbAc="MI:0467" id="R-HSA-3134975"**/>

<**secondaryRef db="reactome" dbAc="MI:0467" id="R-HSA-3249367"**/>

<**secondaryRef db="reactome" dbAc="MI:0467" id="R-HSA-3270619"**/>

<**secondaryRef db="go" dbAc="MI:0448" id="GO:0005654"**/>

<**secondaryRef db="go" dbAc="MI:0448" id="GO:0005829"**/>

<**secondaryRef db="go" dbAc="MI:0448" id="GO:0030667"**/>

<**secondaryRef db="go" dbAc="MI:0448" id="GO:0043312"**/>

<**secondaryRef db="rcsb pdb" dbAc="MI:0460" id="4QXO"**/>

<**secondaryRef db="rcsb pdb" dbAc="MI:0460" id="4QXP"**/>

<**secondaryRef db="rcsb pdb" dbAc="MI:0460" id="4QXQ"**/>

<**secondaryRef db="rcsb pdb" dbAc="MI:0460" id="4QXR"**/>

<**secondaryRef db="go" dbAc="MI:0448" id="GO:0042802"**/>

</**xref**>

<**interactorType**>

<**names**>

<**shortLabel**>protein</**shortLabel**>

<**fullName**>protein</**fullName**>

</**names**>

<**xref**>

<**primaryRef db="psi-mi" dbAc="MI:0488" id="MI:0326" refType="identity" refTypeAc="MI:0356"**/>

<**secondaryRef db="intact" dbAc="MI:0469" id="EBI-619654" refType="identity" refTypeAc="MI:0356"**/>

<**secondaryRef db="pubmed" dbAc="MI:0446" id="14755292" refType="primary-reference" refTypeAc="MI:0358"**/>

<**secondaryRef db="so" dbAc="MI:0601" id="SO:0000358" refType="see-also" refTypeAc="MI:0361"**/>

</**xref**>

</**interactorType**>

<**organism ncbiTaxId="9606"**>

<**names**>

<**shortLabel**>human</**shortLabel**>

<**fullName**>Homo sapiens</**fullName**>

<**alias type="synonym" typeAc="MI:1041"**>Human</**alias**>

</**names**>

</**organism**>

<**sequence**>

MPHSSLHPSIPCPRGHGAQKAALVLLSACLVTLWGLGEPPEHTLRYLVLHLASLQLGLLLNGVCSLAEELRHIHSRYRGSYWRTVRACLGCPLRRGALLLLSIYFYYSLPNAVGPPFTWMLALLGLSQALNILLGLKGLAPAEISAVCEKGNFNVAHGLAWSYYIGYLRLILPELQARIRTYNQHYNNLLRGAVSQRLYILLPLDCGVPDNLSMADPNIRFLDKLPQQTGDHAGIKDRVYSNSIYELLENGQRAGTCVLEYATPLQTLFAMSQYSQAGFSREDRLEQAKLFCRTLEDILADAPESQNNCRLIAYQEPADDSSFSLSQEVLRHLRQEEKEEVTVGSLKTSAVPSTSTMSQEPELLISGMEKPLPLRTDFS

</**sequence**>

<**attributeList**>

<**attribute name="crc64"**>CB54D6A4D4D8E7C0</**attribute**>

</**attributeList**>

</**interactor**>

<**interactor id="8"**>

<**names**>

<**shortLabel**>tbk1_human</**shortLabel**>

<**fullName**>Serine/threonine-protein kinase TBK1</**fullName**>

<**alias type="gene name" typeAc="MI:0301"**>TBK1</**alias**>

<**alias type="gene name synonym" typeAc="MI:0302"**>NAK</**alias**>

<**alias type="gene name synonym" typeAc="MI:0302"**>TANK-binding kinase 1</**alias**>

<**alias type="gene name synonym" typeAc="MI:0302"**>T2K</**alias**>

<**alias type="gene name synonym" typeAc="MI:0302"**>NF-kappa-B-activating kinase</**alias**>

</**names**>

<**xref**>

<**primaryRef db="uniprotkb" dbAc="MI:0486" id="Q9UHD2" version="TrEMBL_29" refType="identity" refTypeAc="MI:0356"**/>

<**secondaryRef db="intact" dbAc="MI:0469" id="EBI-5773956" refType="intact-secondary"**/>

<**secondaryRef db="intact" dbAc="MI:0469" id="EBI-1383470" refType="intact-secondary"**/>

<**secondaryRef db="uniprotkb" dbAc="MI:0486" id="Q8IYV3" version="SP_59" refType="secondary-ac" refTypeAc="MI:0360"**/>

<**secondaryRef db="uniprotkb" dbAc="MI:0486" id="Q9NUJ5" version="SP_59" refType="secondary-ac" refTypeAc="MI:0360"**/>

<**secondaryRef db="uniprotkb" dbAc="MI:0486" id="A8K4S4" version="SP_71" refType="secondary-ac" refTypeAc="MI:0360"**/>

<**secondaryRef db="intact" dbAc="MI:0469" id="EBI-356402" refType="identity" refTypeAc="MI:0356"**/>

<**secondaryRef db="rcsb pdb" dbAc="MI:0460" id="4IM3"**/>

<**secondaryRef db="rcsb pdb" dbAc="MI:0460" id="4IW0"**/>

<**secondaryRef db="rcsb pdb" dbAc="MI:0460" id="4IWO"**/>

<**secondaryRef db="rcsb pdb" dbAc="MI:0460" id="4IWP"**/>

<**secondaryRef db="rcsb pdb" dbAc="MI:0460" id="4IWQ"**/>

<**secondaryRef db="rcsb pdb" dbAc="MI:0460" id="4IM0"**/>

<**secondaryRef db="rcsb pdb" dbAc="MI:0460" id="4IM2"**/>

<**secondaryRef db="go" dbAc="MI:0448" id="GO:0003676"**/>

<**secondaryRef db="go" dbAc="MI:0448" id="GO:0010629"**/>

<**secondaryRef db="go" dbAc="MI:0448" id="GO:0045359"**/>

<**secondaryRef db="go" dbAc="MI:0448" id="GO:0050830"**/>

<**secondaryRef db="rcsb pdb" dbAc="MI:0460" id="4EUT"**/>

<**secondaryRef db="rcsb pdb" dbAc="MI:0460" id="4EUU"**/>

<**secondaryRef db="go" dbAc="MI:0448" id="GO:0032480"**/>

<**secondaryRef db="go" dbAc="MI:0448" id="GO:0043123"**/>

<**secondaryRef db="go" dbAc="MI:0448" id="GO:0032727"**/>

<**secondaryRef db="go" dbAc="MI:0448" id="GO:0032728"**/>

<**secondaryRef db="go" dbAc="MI:0448" id="GO:0045944"**/>

<**secondaryRef db="go" dbAc="MI:0448" id="GO:0009615"**/>

<**secondaryRef db="refseq" dbAc="MI:0481" id="NP_037386.1"**/>

<**secondaryRef db="interpro" dbAc="MI:0449" id="IPR011009"**/>

<**secondaryRef db="interpro" dbAc="MI:0449" id="IPR000719"**/>

<**secondaryRef db="interpro" dbAc="MI:0449" id="IPR017441"**/>

<**secondaryRef db="go" dbAc="MI:0448" id="GO:0005829"**/>

<**secondaryRef db="go" dbAc="MI:0448" id="GO:0010008"**/>

<**secondaryRef db="go" dbAc="MI:0448" id="GO:0005524"**/>

<**secondaryRef db="go" dbAc="MI:0448" id="GO:0004674"**/>

<**secondaryRef db="go" dbAc="MI:0448" id="GO:0007249"**/>

<**secondaryRef db="go" dbAc="MI:0448" id="GO:0045087"**/>

<**secondaryRef db="rcsb pdb" dbAc="MI:0460" id="4EFO"**/>

<**secondaryRef db="go" dbAc="MI:0448" id="GO:0035666"**/>

<**secondaryRef db="go" dbAc="MI:0448" id="GO:0006954"**/>

<**secondaryRef db="go" dbAc="MI:0448" id="GO:0032606"**/>

<**secondaryRef db="ensembl" dbAc="MI:0476" id="ENSG00000183735"**/>

<**secondaryRef db="ensembl" dbAc="MI:0476" id="ENSP00000329967"**/>

<**secondaryRef db="go" dbAc="MI:0448" id="GO:0033138"**/>

<**secondaryRef db="go" dbAc="MI:0448" id="GO:0051607"**/>

<**secondaryRef db="refseq" dbAc="MI:0481" id="XP_005268866.1"**/>

<**secondaryRef db="refseq" dbAc="MI:0481" id="XP_005268867.1"**/>

<**secondaryRef db="go" dbAc="MI:0448" id="GO:0004672"**/>

<**secondaryRef db="go" dbAc="MI:0448" id="GO:0005737"**/>

<**secondaryRef db="go" dbAc="MI:0448" id="GO:0032481"**/>

<**secondaryRef db="go" dbAc="MI:0448" id="GO:0051219"**/>

<**secondaryRef db="ensembl" dbAc="MI:0476" id="ENST00000331710"**/>

<**secondaryRef db="go" dbAc="MI:0448" id="GO:0018105"**/>

<**secondaryRef db="go" dbAc="MI:0448" id="GO:1901214"**/>

<**secondaryRef db="reactome" dbAc="MI:0467" id="R-HSA-1606341"**/>

<**secondaryRef db="reactome" dbAc="MI:0467" id="R-HSA-3134975"**/>

<**secondaryRef db="reactome" dbAc="MI:0467" id="R-HSA-3249367"**/>

<**secondaryRef db="reactome" dbAc="MI:0467" id="R-HSA-3270619"**/>

<**secondaryRef db="reactome" dbAc="MI:0467" id="R-HSA-918233"**/>

<**secondaryRef db="reactome" dbAc="MI:0467" id="R-HSA-933541"**/>

<**secondaryRef db="reactome" dbAc="MI:0467" id="R-HSA-936440"**/>

<**secondaryRef db="reactome" dbAc="MI:0467" id="R-HSA-936964"**/>

<**secondaryRef db="go" dbAc="MI:0448" id="GO:0044565"**/>

<**secondaryRef db="go" dbAc="MI:0448" id="GO:1904417"**/>

<**secondaryRef db="go" dbAc="MI:0448" id="GO:0016239"**/>

<**secondaryRef db="go" dbAc="MI:0448" id="GO:0018107"**/>

<**secondaryRef db="rcsb pdb" dbAc="MI:0460" id="5EOA"**/>

<**secondaryRef db="rcsb pdb" dbAc="MI:0460" id="5EOF"**/>

<**secondaryRef db="rcsb pdb" dbAc="MI:0460" id="5EP6"**/>

<**secondaryRef db="go" dbAc="MI:0448" id="GO:0032479"**/>

</**xref**>

<**interactorType**>

<**names**>

<**shortLabel**>protein</**shortLabel**>

<**fullName**>protein</**fullName**>

</**names**>

<**xref**>

<**primaryRef db="psi-mi" dbAc="MI:0488" id="MI:0326" refType="identity" refTypeAc="MI:0356"**/>

<**secondaryRef db="intact" dbAc="MI:0469" id="EBI-619654" refType="identity" refTypeAc="MI:0356"**/>

<**secondaryRef db="pubmed" dbAc="MI:0446" id="14755292" refType="primary-reference" refTypeAc="MI:0358"**/>

<**secondaryRef db="so" dbAc="MI:0601" id="SO:0000358" refType="see-also" refTypeAc="MI:0361"**/>

</**xref**>

</**interactorType**>

<**organism ncbiTaxId="9606"**>

<**names**>

<**shortLabel**>human</**shortLabel**>

<**fullName**>Homo sapiens</**fullName**>

<**alias type="synonym" typeAc="MI:1041"**>Human</**alias**>

</**names**>

</**organism**>

<**sequence**>

MQSTSNHLWLLSDILGQGATANVFRGRHKKTGDLFAIKVFNNISFLRPVDVQMREFEVLKKLNHKNIVKLFAIEEETTTRHKVLIMEFCPCGSLYTVLEEPSNAYGLPESEFLIVLRDVVGGMNHLRENGIVHRDIKPGNIMRVIGEDGQSVYKLTDFGAARELEDDEQFVSLYGTEEYLHPDMYERAVLRKDHQKKYGATVDLWSIGVTFYHAATGSLPFRPFEGPRRNKEVMYKIITGKPSGAISGVQKAENGPIDWSGDMPVSCSLSRGLQVLLTPVLANILEADQEKCWGFDQFFAETSDILHRMVIHVFSLQQMTAHKIYIHSYNTATIFHELVYKQTKIISSNQELIYEGRRLVLEPGRLAQHFPKTTEENPIFVVSREPLNTIGLIYEKISLPKVHPRYDLDGDASMAKAITGVVCYACRIASTLLLYQELMRKGIRWLIELIKDDYNETVHKKTEVVITLDFCIRNIEKTVKVYEKLMKINLEAAELGEISDIHTKLLRLSSSQGTIETSLQDIDSRLSPGGSLADAWAHQEGTHPKDRNVEKLQVLLNCMTEIYYQFKKDKAERRLAYNEEQIHKFDKQKLYYHATKAMTHFTDECVKKYEAFLNKSEEWIRKMLHLRKQLLSLTNQCFDIEEEVSKYQEYTNELQETLPQKMFTASSGIKHTMTPIYPSSNTLVEMTLGMKKLKEEMEGVVKELAENNHILERFGSLTMDGGLRNVDCL

</**sequence**>

<**attributeList**>

<**attribute name="crc64"**>B58E4FE1B502276D</**attribute**>

</**attributeList**>

</**interactor**>

</**interactorList**>

<**interactionList**>

<**interaction id="9" imexId="IM-16625-3"**>

<**names**>

<**shortLabel**>stat6-tmem173-2</**shortLabel**>

</**names**>

<**xref**>

<**primaryRef db="intact" dbAc="MI:0469" id="EBI-5237739" refType="identity" refTypeAc="MI:0356"**/>

<**secondaryRef db="psi-mi" dbAc="MI:0488" id="MI:0469" refType="imex source"**/>

<**secondaryRef db="imex" dbAc="MI:0670" id="IM-16625-3" refType="imex-primary" refTypeAc="MI:0662"**/>

</**xref**>

<**experimentList**>

<**experimentRef**>1</**experimentRef**>

</**experimentList**>

<**participantList**>

<**participant id="10"**>

<**interactorRef**>6</**interactorRef**>

<**biologicalRole**>

<**names**>

<**shortLabel**>unspecified role</**shortLabel**>

<**fullName**>unspecified role</**fullName**>

</**names**>

<**xref**>

<**primaryRef db="psi-mi" dbAc="MI:0488" id="MI:0499" refType="identity" refTypeAc="MI:0356"**/>

<**secondaryRef db="intact" dbAc="MI:0469" id="EBI-77781" refType="identity" refTypeAc="MI:0356"**/>

<**secondaryRef db="pubmed" dbAc="MI:0446" id="14755292" refType="primary-reference" refTypeAc="MI:0358"**/>

</**xref**>

</**biologicalRole**>

<**experimentalRoleList**>

<**experimentalRole**>

<**names**>

<**shortLabel**>bait</**shortLabel**>

<**fullName**>bait</**fullName**>

</**names**>

<**xref**>

<**primaryRef db="psi-mi" dbAc="MI:0488" id="MI:0496" refType="identity" refTypeAc="MI:0356"**/>

<**secondaryRef db="intact" dbAc="MI:0469" id="EBI-49" refType="identity" refTypeAc="MI:0356"**/>

<**secondaryRef db="pubmed" dbAc="MI:0446" id="14755292" refType="primary-reference" refTypeAc="MI:0358"**/>

</**xref**>

</**experimentalRole**>

</**experimentalRoleList**>

<**featureList**>

<**feature id="11"**>

<**names**>

<**shortLabel**>region</**shortLabel**>

</**names**>

<**xref**>

<**primaryRef db="intact" dbAc="MI:0469" id="EBI-5237745" refType="identity" refTypeAc="MI:0356"**/>

</**xref**>

<**featureType**>

<**names**>

<**shortLabel**>flag tag</**shortLabel**>

<**fullName**>flag tag</**fullName**>

<**alias type="go synonym" typeAc="MI:0303"**>FLAG-tagged</**alias**>

<**alias type="go synonym" typeAc="MI:0303"**>FLAG</**alias**>

<**alias type="go synonym" typeAc="MI:0303"**>DYKDDDDKV epitope tag</**alias**>

</**names**>

<**xref**>

<**primaryRef db="psi-mi" dbAc="MI:0488" id="MI:0518" refType="identity" refTypeAc="MI:0356"**/>

<**secondaryRef db="intact" dbAc="MI:0469" id="EBI-456503" refType="identity" refTypeAc="MI:0356"**/>

<**secondaryRef db="pubmed" dbAc="MI:0446" id="14755292" refType="primary-reference" refTypeAc="MI:0358"**/>

</**xref**>

</**featureType**>

<**featureRangeList**>

<**featureRange**>

<**startStatus**>

<**names**>

<**shortLabel**>undetermined</**shortLabel**>

<**fullName**>undetermined sequence position</**fullName**>

</**names**>

<**xref**>

<**primaryRef db="psi-mi" dbAc="MI:0488" id="MI:0339" refType="identity" refTypeAc="MI:0356"**/>

<**secondaryRef db="intact" dbAc="MI:0469" id="EBI-448295" refType="identity" refTypeAc="MI:0356"**/>

<**secondaryRef db="pubmed" dbAc="MI:0446" id="14755292" refType="primary-reference" refTypeAc="MI:0358"**/>

</**xref**>

</**startStatus**>

<**endStatus**>

<**names**>

<**shortLabel**>undetermined</**shortLabel**>

<**fullName**>undetermined sequence position</**fullName**>

</**names**>

<**xref**>

<**primaryRef db="psi-mi" dbAc="MI:0488" id="MI:0339" refType="identity" refTypeAc="MI:0356"**/>

<**secondaryRef db="intact" dbAc="MI:0469" id="EBI-448295" refType="identity" refTypeAc="MI:0356"**/>

<**secondaryRef db="pubmed" dbAc="MI:0446" id="14755292" refType="primary-reference" refTypeAc="MI:0358"**/>

</**xref**>

</**endStatus**>

</**featureRange**>

</**featureRangeList**>

</**feature**>

</**featureList**>

</**participant**>

<**participant id="12"**>

<**interactorRef**>7</**interactorRef**>

<**biologicalRole**>

<**names**>

<**shortLabel**>unspecified role</**shortLabel**>

<**fullName**>unspecified role</**fullName**>

</**names**>

<**xref**>

<**primaryRef db="psi-mi" dbAc="MI:0488" id="MI:0499" refType="identity" refTypeAc="MI:0356"**/>

<**secondaryRef db="intact" dbAc="MI:0469" id="EBI-77781" refType="identity" refTypeAc="MI:0356"**/>

<**secondaryRef db="pubmed" dbAc="MI:0446" id="14755292" refType="primary-reference" refTypeAc="MI:0358"**/>

</**xref**>

</**biologicalRole**>

<**experimentalRoleList**>

<**experimentalRole**>

<**names**>

<**shortLabel**>prey</**shortLabel**>

<**fullName**>prey</**fullName**>

</**names**>

<**xref**>

<**primaryRef db="psi-mi" dbAc="MI:0488" id="MI:0498" refType="identity" refTypeAc="MI:0356"**/>

<**secondaryRef db="intact" dbAc="MI:0469" id="EBI-58" refType="identity" refTypeAc="MI:0356"**/>

<**secondaryRef db="pubmed" dbAc="MI:0446" id="14755292" refType="primary-reference" refTypeAc="MI:0358"**/>

</**xref**>

</**experimentalRole**>

</**experimentalRoleList**>

</**participant**>

</**participantList**>

<**interactionType**>

<**names**>

<**shortLabel**>physical association</**shortLabel**>

<**fullName**>physical association</**fullName**>

</**names**>

<**xref**>

<**primaryRef db="psi-mi" dbAc="MI:0488" id="MI:0915" refType="identity" refTypeAc="MI:0356"**/>

<**secondaryRef db="intact" dbAc="MI:0469" id="EBI-1813147" refType="identity" refTypeAc="MI:0356"**/>

<**secondaryRef db="pubmed" dbAc="MI:0446" id="14755292" refType="primary-reference" refTypeAc="MI:0358"**/>

</**xref**>

</**interactionType**>

<**experimentalVariableValueList**>

<**experimentalVariableValues**>

<**variableValueRef**>2</**variableValueRef**>

</**experimentalVariableValues**>

</**experimentalVariableValueList**>

<**attributeList**>

<**attribute name="figure legend" nameAc="MI:0599"**>Supp. Fig. 1E</**attribute**>

</**attributeList**>

</**interaction**>

<**interaction id="13" imexId="IM-16625-1"**>

<**names**>

<**shortLabel**>stat6-mavs-4</**shortLabel**>

</**names**>

<**xref**>

<**primaryRef db="intact" dbAc="MI:0469" id="EBI-5237770" refType="identity" refTypeAc="MI:0356"**/>

<**secondaryRef db="psi-mi" dbAc="MI:0488" id="MI:0469" refType="imex source"**/>

<**secondaryRef db="imex" dbAc="MI:0670" id="IM-16625-1" refType="imex-primary" refTypeAc="MI:0662"**/>

</**xref**>

<**experimentList**>

<**experimentRef**>1</**experimentRef**>

</**experimentList**>

<**participantList**>

<**participant id="14"**>

<**interactorRef**>8</**interactorRef**>

<**biologicalRole**>

<**names**>

<**shortLabel**>unspecified role</**shortLabel**>

<**fullName**>unspecified role</**fullName**>

</**names**>

<**xref**>

<**primaryRef db="psi-mi" dbAc="MI:0488" id="MI:0499" refType="identity" refTypeAc="MI:0356"**/>

<**secondaryRef db="intact" dbAc="MI:0469" id="EBI-77781" refType="identity" refTypeAc="MI:0356"**/>

<**secondaryRef db="pubmed" dbAc="MI:0446" id="14755292" refType="primary-reference" refTypeAc="MI:0358"**/>

</**xref**>

</**biologicalRole**>

<**experimentalRoleList**>

<**experimentalRole**>

<**names**>

<**shortLabel**>prey</**shortLabel**>

<**fullName**>prey</**fullName**>

</**names**>

<**xref**>

<**primaryRef db="psi-mi" dbAc="MI:0488" id="MI:0498" refType="identity" refTypeAc="MI:0356"**/>

<**secondaryRef db="intact" dbAc="MI:0469" id="EBI-58" refType="identity" refTypeAc="MI:0356"**/>

<**secondaryRef db="pubmed" dbAc="MI:0446" id="14755292" refType="primary-reference" refTypeAc="MI:0358"**/>

</**xref**>

</**experimentalRole**>

</**experimentalRoleList**>

</**participant**>

<**participant id="15"**>

<**interactorRef**>5</**interactorRef**>

<**biologicalRole**>

<**names**>

<**shortLabel**>unspecified role</**shortLabel**>

<**fullName**>unspecified role</**fullName**>

</**names**>

<**xref**>

<**primaryRef db="psi-mi" dbAc="MI:0488" id="MI:0499" refType="identity" refTypeAc="MI:0356"**/>

<**secondaryRef db="intact" dbAc="MI:0469" id="EBI-77781" refType="identity" refTypeAc="MI:0356"**/>

<**secondaryRef db="pubmed" dbAc="MI:0446" id="14755292" refType="primary-reference" refTypeAc="MI:0358"**/>

</**xref**>

</**biologicalRole**>

<**experimentalRoleList**>

<**experimentalRole**>

<**names**>

<**shortLabel**>prey</**shortLabel**>

<**fullName**>prey</**fullName**>

</**names**>

<**xref**>

<**primaryRef db="psi-mi" dbAc="MI:0488" id="MI:0498" refType="identity" refTypeAc="MI:0356"**/>

<**secondaryRef db="intact" dbAc="MI:0469" id="EBI-58" refType="identity" refTypeAc="MI:0356"**/>

<**secondaryRef db="pubmed" dbAc="MI:0446" id="14755292" refType="primary-reference" refTypeAc="MI:0358"**/>

</**xref**>

</**experimentalRole**>

</**experimentalRoleList**>

</**participant**>

<**participant id="16"**>

<**interactorRef**>6</**interactorRef**>

<**biologicalRole**>

<**names**>

<**shortLabel**>unspecified role</**shortLabel**>

<**fullName**>unspecified role</**fullName**>

</**names**>

<**xref**>

<**primaryRef db="psi-mi" dbAc="MI:0488" id="MI:0499" refType="identity" refTypeAc="MI:0356"**/>

<**secondaryRef db="intact" dbAc="MI:0469" id="EBI-77781" refType="identity" refTypeAc="MI:0356"**/>

<**secondaryRef db="pubmed" dbAc="MI:0446" id="14755292" refType="primary-reference" refTypeAc="MI:0358"**/>

</**xref**>

</**biologicalRole**>

<**experimentalRoleList**>

<**experimentalRole**>

<**names**>

<**shortLabel**>bait</**shortLabel**>

<**fullName**>bait</**fullName**>

</**names**>

<**xref**>

<**primaryRef db="psi-mi" dbAc="MI:0488" id="MI:0496" refType="identity" refTypeAc="MI:0356"**/>

<**secondaryRef db="intact" dbAc="MI:0469" id="EBI-49" refType="identity" refTypeAc="MI:0356"**/>

<**secondaryRef db="pubmed" dbAc="MI:0446" id="14755292" refType="primary-reference" refTypeAc="MI:0358"**/>

</**xref**>

</**experimentalRole**>

</**experimentalRoleList**>

<**featureList**>

<**feature id="17"**>

<**names**>

<**shortLabel**>region</**shortLabel**>

</**names**>

<**xref**>

<**primaryRef db="intact" dbAc="MI:0469" id="EBI-5237775" refType="identity" refTypeAc="MI:0356"**/>

</**xref**>

<**featureType**>

<**names**>

<**shortLabel**>flag tag</**shortLabel**>

<**fullName**>flag tag</**fullName**>

<**alias type="go synonym" typeAc="MI:0303"**>FLAG-tagged</**alias**>

<**alias type="go synonym" typeAc="MI:0303"**>FLAG</**alias**>

<**alias type="go synonym" typeAc="MI:0303"**>DYKDDDDKV epitope tag</**alias**>

</**names**>

<**xref**>

<**primaryRef db="psi-mi" dbAc="MI:0488" id="MI:0518" refType="identity" refTypeAc="MI:0356"**/>

<**secondaryRef db="intact" dbAc="MI:0469" id="EBI-456503" refType="identity" refTypeAc="MI:0356"**/>

<**secondaryRef db="pubmed" dbAc="MI:0446" id="14755292" refType="primary-reference" refTypeAc="MI:0358"**/>

</**xref**>

</**featureType**>

<**featureRangeList**>

<**featureRange**>

<**startStatus**>

<**names**>

<**shortLabel**>undetermined</**shortLabel**>

<**fullName**>undetermined sequence position</**fullName**>

</**names**>

<**xref**>

<**primaryRef db="psi-mi" dbAc="MI:0488" id="MI:0339" refType="identity" refTypeAc="MI:0356"**/>

<**secondaryRef db="intact" dbAc="MI:0469" id="EBI-448295" refType="identity" refTypeAc="MI:0356"**/>

<**secondaryRef db="pubmed" dbAc="MI:0446" id="14755292" refType="primary-reference" refTypeAc="MI:0358"**/>

</**xref**>

</**startStatus**>

<**endStatus**>

<**names**>

<**shortLabel**>undetermined</**shortLabel**>

<**fullName**>undetermined sequence position</**fullName**>

</**names**>

<**xref**>

<**primaryRef db="psi-mi" dbAc="MI:0488" id="MI:0339" refType="identity" refTypeAc="MI:0356"**/>

<**secondaryRef db="intact" dbAc="MI:0469" id="EBI-448295" refType="identity" refTypeAc="MI:0356"**/>

<**secondaryRef db="pubmed" dbAc="MI:0446" id="14755292" refType="primary-reference" refTypeAc="MI:0358"**/>

</**xref**>

</**endStatus**>

</**featureRange**>

</**featureRangeList**>

</**feature**>

</**featureList**>

</**participant**>

<**participant id="18"**>

<**interactorRef**>7</**interactorRef**>

<**biologicalRole**>

<**names**>

<**shortLabel**>unspecified role</**shortLabel**>

<**fullName**>unspecified role</**fullName**>

</**names**>

<**xref**>

<**primaryRef db="psi-mi" dbAc="MI:0488" id="MI:0499" refType="identity" refTypeAc="MI:0356"**/>

<**secondaryRef db="intact" dbAc="MI:0469" id="EBI-77781" refType="identity" refTypeAc="MI:0356"**/>

<**secondaryRef db="pubmed" dbAc="MI:0446" id="14755292" refType="primary-reference" refTypeAc="MI:0358"**/>

</**xref**>

</**biologicalRole**>

<**experimentalRoleList**>

<**experimentalRole**>

<**names**>

<**shortLabel**>prey</**shortLabel**>

<**fullName**>prey</**fullName**>

</**names**>

<**xref**>

<**primaryRef db="psi-mi" dbAc="MI:0488" id="MI:0498" refType="identity" refTypeAc="MI:0356"**/>

<**secondaryRef db="intact" dbAc="MI:0469" id="EBI-58" refType="identity" refTypeAc="MI:0356"**/>

<**secondaryRef db="pubmed" dbAc="MI:0446" id="14755292" refType="primary-reference" refTypeAc="MI:0358"**/>

</**xref**>

</**experimentalRole**>

</**experimentalRoleList**>

</**participant**>

</**participantList**>

<**interactionType**>

<**names**>

<**shortLabel**>association</**shortLabel**>

<**fullName**>association</**fullName**>

</**names**>

<**xref**>

<**primaryRef db="psi-mi" dbAc="MI:0488" id="MI:0914" refType="identity" refTypeAc="MI:0356"**/>

<**secondaryRef db="intact" dbAc="MI:0469" id="EBI-1813191" refType="identity" refTypeAc="MI:0356"**/>

<**secondaryRef db="pubmed" dbAc="MI:0446" id="14755292" refType="primary-reference" refTypeAc="MI:0358"**/>

</**xref**>

</**interactionType**>

<**experimentalVariableValueList**>

<**experimentalVariableValues**>

<**variableValueRef**>3</**variableValueRef**>

</**experimentalVariableValues**>

</**experimentalVariableValueList**>

<**attributeList**>

<**attribute name="figure legend" nameAc="MI:0599"**>Supp. Fig. 1E</**attribute**>

</**attributeList**>

</**interaction**>

<**interaction id="19" imexId="IM-16625-2"**>

<**names**>

<**shortLabel**>stat6-mavs-1</**shortLabel**>

</**names**>

<**xref**>

<**primaryRef db="intact" dbAc="MI:0469" id="EBI-5237802" refType="identity" refTypeAc="MI:0356"**/>

<**secondaryRef db="psi-mi" dbAc="MI:0488" id="MI:0469" refType="imex source"**/>

<**secondaryRef db="imex" dbAc="MI:0670" id="IM-16625-2" refType="imex-primary" refTypeAc="MI:0662"**/>

</**xref**>

<**experimentList**>

<**experimentRef**>1</**experimentRef**>

</**experimentList**>

<**participantList**>

<**participant id="20"**>

<**interactorRef**>7</**interactorRef**>

<**biologicalRole**>

<**names**>

<**shortLabel**>unspecified role</**shortLabel**>

<**fullName**>unspecified role</**fullName**>

</**names**>

<**xref**>

<**primaryRef db="psi-mi" dbAc="MI:0488" id="MI:0499" refType="identity" refTypeAc="MI:0356"**/>

<**secondaryRef db="intact" dbAc="MI:0469" id="EBI-77781" refType="identity" refTypeAc="MI:0356"**/>

<**secondaryRef db="pubmed" dbAc="MI:0446" id="14755292" refType="primary-reference" refTypeAc="MI:0358"**/>

</**xref**>

</**biologicalRole**>

<**experimentalRoleList**>

<**experimentalRole**>

<**names**>

<**shortLabel**>prey</**shortLabel**>

<**fullName**>prey</**fullName**>

</**names**>

<**xref**>

<**primaryRef db="psi-mi" dbAc="MI:0488" id="MI:0498" refType="identity" refTypeAc="MI:0356"**/>

<**secondaryRef db="intact" dbAc="MI:0469" id="EBI-58" refType="identity" refTypeAc="MI:0356"**/>

<**secondaryRef db="pubmed" dbAc="MI:0446" id="14755292" refType="primary-reference" refTypeAc="MI:0358"**/>

</**xref**>

</**experimentalRole**>

</**experimentalRoleList**>

</**participant**>

<**participant id="21"**>

<**interactorRef**>8</**interactorRef**>

<**biologicalRole**>

<**names**>

<**shortLabel**>unspecified role</**shortLabel**>

<**fullName**>unspecified role</**fullName**>

</**names**>

<**xref**>

<**primaryRef db="psi-mi" dbAc="MI:0488" id="MI:0499" refType="identity" refTypeAc="MI:0356"**/>

<**secondaryRef db="intact" dbAc="MI:0469" id="EBI-77781" refType="identity" refTypeAc="MI:0356"**/>

<**secondaryRef db="pubmed" dbAc="MI:0446" id="14755292" refType="primary-reference" refTypeAc="MI:0358"**/>

</**xref**>

</**biologicalRole**>

<**experimentalRoleList**>

<**experimentalRole**>

<**names**>

<**shortLabel**>prey</**shortLabel**>

<**fullName**>prey</**fullName**>

</**names**>

<**xref**>

<**primaryRef db="psi-mi" dbAc="MI:0488" id="MI:0498" refType="identity" refTypeAc="MI:0356"**/>

<**secondaryRef db="intact" dbAc="MI:0469" id="EBI-58" refType="identity" refTypeAc="MI:0356"**/>

<**secondaryRef db="pubmed" dbAc="MI:0446" id="14755292" refType="primary-reference" refTypeAc="MI:0358"**/>

</**xref**>

</**experimentalRole**>

</**experimentalRoleList**>

</**participant**>

<**participant id="22"**>

<**interactorRef**>6</**interactorRef**>

<**biologicalRole**>

<**names**>

<**shortLabel**>unspecified role</**shortLabel**>

<**fullName**>unspecified role</**fullName**>

</**names**>

<**xref**>

<**primaryRef db="psi-mi" dbAc="MI:0488" id="MI:0499" refType="identity" refTypeAc="MI:0356"**/>

<**secondaryRef db="intact" dbAc="MI:0469" id="EBI-77781" refType="identity" refTypeAc="MI:0356"**/>

<**secondaryRef db="pubmed" dbAc="MI:0446" id="14755292" refType="primary-reference" refTypeAc="MI:0358"**/>

</**xref**>

</**biologicalRole**>

<**experimentalRoleList**>

<**experimentalRole**>

<**names**>

<**shortLabel**>bait</**shortLabel**>

<**fullName**>bait</**fullName**>

</**names**>

<**xref**>

<**primaryRef db="psi-mi" dbAc="MI:0488" id="MI:0496" refType="identity" refTypeAc="MI:0356"**/>

<**secondaryRef db="intact" dbAc="MI:0469" id="EBI-49" refType="identity" refTypeAc="MI:0356"**/>

<**secondaryRef db="pubmed" dbAc="MI:0446" id="14755292" refType="primary-reference" refTypeAc="MI:0358"**/>

</**xref**>

</**experimentalRole**>

</**experimentalRoleList**>

<**featureList**>

<**feature id="23"**>

<**names**>

<**shortLabel**>region</**shortLabel**>

</**names**>

<**xref**>

<**primaryRef db="intact" dbAc="MI:0469" id="EBI-5237809" refType="identity" refTypeAc="MI:0356"**/>

</**xref**>

<**featureType**>

<**names**>

<**shortLabel**>flag tag</**shortLabel**>

<**fullName**>flag tag</**fullName**>

<**alias type="go synonym" typeAc="MI:0303"**>FLAG-tagged</**alias**>

<**alias type="go synonym" typeAc="MI:0303"**>FLAG</**alias**>

<**alias type="go synonym" typeAc="MI:0303"**>DYKDDDDKV epitope tag</**alias**>

</**names**>

<**xref**>

<**primaryRef db="psi-mi" dbAc="MI:0488" id="MI:0518" refType="identity" refTypeAc="MI:0356"**/>

<**secondaryRef db="intact" dbAc="MI:0469" id="EBI-456503" refType="identity" refTypeAc="MI:0356"**/>

<**secondaryRef db="pubmed" dbAc="MI:0446" id="14755292" refType="primary-reference" refTypeAc="MI:0358"**/>

</**xref**>

</**featureType**>

<**featureRangeList**>

<**featureRange**>

<**startStatus**>

<**names**>

<**shortLabel**>undetermined</**shortLabel**>

<**fullName**>undetermined sequence position</**fullName**>

</**names**>

<**xref**>

<**primaryRef db="psi-mi" dbAc="MI:0488" id="MI:0339" refType="identity" refTypeAc="MI:0356"**/>

<**secondaryRef db="intact" dbAc="MI:0469" id="EBI-448295" refType="identity" refTypeAc="MI:0356"**/>

<**secondaryRef db="pubmed" dbAc="MI:0446" id="14755292" refType="primary-reference" refTypeAc="MI:0358"**/>

</**xref**>

</**startStatus**>

<**endStatus**>

<**names**>

<**shortLabel**>undetermined</**shortLabel**>

<**fullName**>undetermined sequence position</**fullName**>

</**names**>

<**xref**>

<**primaryRef db="psi-mi" dbAc="MI:0488" id="MI:0339" refType="identity" refTypeAc="MI:0356"**/>

<**secondaryRef db="intact" dbAc="MI:0469" id="EBI-448295" refType="identity" refTypeAc="MI:0356"**/>

<**secondaryRef db="pubmed" dbAc="MI:0446" id="14755292" refType="primary-reference" refTypeAc="MI:0358"**/>

</**xref**>

</**endStatus**>

</**featureRange**>

</**featureRangeList**>

</**feature**>

</**featureList**>

</**participant**>

<**participant id="24"**>

<**interactorRef**>5</**interactorRef**>

<**biologicalRole**>

<**names**>

<**shortLabel**>unspecified role</**shortLabel**>

<**fullName**>unspecified role</**fullName**>

</**names**>

<**xref**>

<**primaryRef db="psi-mi" dbAc="MI:0488" id="MI:0499" refType="identity" refTypeAc="MI:0356"**/>

<**secondaryRef db="intact" dbAc="MI:0469" id="EBI-77781" refType="identity" refTypeAc="MI:0356"**/>

<**secondaryRef db="pubmed" dbAc="MI:0446" id="14755292" refType="primary-reference" refTypeAc="MI:0358"**/>

</**xref**>

</**biologicalRole**>

<**experimentalRoleList**>

<**experimentalRole**>

<**names**>

<**shortLabel**>prey</**shortLabel**>

<**fullName**>prey</**fullName**>

</**names**>

<**xref**>

<**primaryRef db="psi-mi" dbAc="MI:0488" id="MI:0498" refType="identity" refTypeAc="MI:0356"**/>

<**secondaryRef db="intact" dbAc="MI:0469" id="EBI-58" refType="identity" refTypeAc="MI:0356"**/>

<**secondaryRef db="pubmed" dbAc="MI:0446" id="14755292" refType="primary-reference" refTypeAc="MI:0358"**/>

</**xref**>

</**experimentalRole**>

</**experimentalRoleList**>

</**participant**>

</**participantList**>

<**interactionType**>

<**names**>

<**shortLabel**>association</**shortLabel**>

<**fullName**>association</**fullName**>

</**names**>

<**xref**>

<**primaryRef db="psi-mi" dbAc="MI:0488" id="MI:0914" refType="identity" refTypeAc="MI:0356"**/>

<**secondaryRef db="intact" dbAc="MI:0469" id="EBI-1813191" refType="identity" refTypeAc="MI:0356"**/>

<**secondaryRef db="pubmed" dbAc="MI:0446" id="14755292" refType="primary-reference" refTypeAc="MI:0358"**/>

</**xref**>

</**interactionType**>

<**experimentalVariableValueList**>

<**experimentalVariableValues**>

<**variableValueRef**>4</**variableValueRef**>

</**experimentalVariableValues**>

</**experimentalVariableValueList**>

<**attributeList**>

<**attribute name="figure legend" nameAc="MI:0599"**>Supp. Fig. 1E</**attribute**>

</**attributeList**>

</**interaction**>

</**interactionList**>

</**entry**>

</**entrySet**>
