# Supplementary material for: Encompassing new use cases - level 3.0 of the HUPO-PSI format for molecular interactions
Source: BMC Bioinformatics. 2018 Apr 11;19:134. doi: 10.1186/s12859-018-2118-1 (PMC5896046; doi:10.1186/s12859-018-2118-1)
Supplement: Supplementary file 7 — Representation of an abstracted interaction, a manually curated protein complex, in PSI-MI XML3.0.0 (use case 1.3 g). (https://github.com/HUPO-PSI/miXML/blob/master/3.0/pub/Appendix%208.docx). (DOCX 48 kb) [file 12859_2018_2118_MOESM7_ESM.docx]

**Representation of an abstracted interaction, a manually curated protein complex, in PSI-MI XML3.0**

The ability to add data which is not directly linked to a single publication but has been abstracted from multiple sources

Example taken from the Complex Portal (www.ebi.ac.uk/complexportal/complex/EBI-10887677)

*<?***xml version="1.0" encoding="UTF-8"***?>*

<**entrySet xmlns:xsi="http://www.w3.org/2001/XMLSchema-instance"**

**xmlns="http://psi.hupo.org/mi/mif300"**

**xsi:schemaLocation="http://psi.hupo.org/mi/mif300 https://raw.githubusercontent.com/HUPO-PSI/miXML/master/3.0/src/MIF300.xsd"**

**level="3" version="0" minorVersion="0"**>

<**entry**>

<**source releaseDate="2017-01-30"**>

<**names**>

<**shortLabel**>bhf-ucl</**shortLabel**>

</**names**>

<**xref**>

<**primaryRef db="psi-mi" dbAc="MI:0488" id="MI:1332" refType="identity" refTypeAc="MI:0356"**/>

<**secondaryRef db="unknown" id="EBI-6693962" refType="identity" refTypeAc="MI:0356"**/>

</**xref**>

</**source**>

<**experimentList**>

<**experimentDescription id="1"**>

<**names**>

<**fullName**>

Mock publication and experiment for abstract interactions that are not interaction evidences.

</**fullName**>

</**names**>

<**bibref**>

<**attributeList**>

<**attribute name="publication title" nameAc="MI:1091"**>

Mock publication and experiment for abstract interactions that are not interaction evidences.

</**attribute**>

</**attributeList**>

</**bibref**>

<**interactionDetectionMethod**>

<**names**>

<**shortLabel**>unspecified method</**shortLabel**>

</**names**>

<**xref**>

<**primaryRef db="psi-mi" dbAc="MI:0488" id="MI:0686" refType="identity" refTypeAc="MI:0356"**/>

</**xref**>

</**interactionDetectionMethod**>

</**experimentDescription**>

</**experimentList**>

<**interactorList**>

<**interactor id="2"**>

<**names**>

<**shortLabel**>te2ip_human</**shortLabel**>

<**fullName**>Telomeric repeat-binding factor 2-interacting protein 1</**fullName**>

<**alias type="gene name synonym" typeAc="MI:0302"**>DRIP5</**alias**>

<**alias type="gene name" typeAc="MI:0301"**>TERF2IP</**alias**>

<**alias type="gene name synonym" typeAc="MI:0302"**>RAP1</**alias**>

<**alias type="orf name" typeAc="MI:0306"**>PP8000</**alias**>

<**alias type="gene name synonym" typeAc="MI:0302"**>Dopamine receptor-interacting protein 5</**alias**>

<**alias type="gene name synonym" typeAc="MI:0302"**>Repressor/activator protein 1 homolog</**alias**>

</**names**>

<**xref**>

<**primaryRef db="uniprotkb" dbAc="MI:0486" id="Q9NYB0" version="SP_70" refType="identity" refTypeAc="MI:0356"**/>

<**secondaryRef db="uniprotkb" dbAc="MI:0486" id="B4DQN4" version="SP_106" refType="secondary-ac" refTypeAc="MI:0360"**/>

<**secondaryRef db="uniprotkb" dbAc="MI:0486" id="Q4W4Y2" version="SP_106" refType="secondary-ac" refTypeAc="MI:0360"**/>

<**secondaryRef db="uniprotkb" dbAc="MI:0486" id="Q8WYZ3" version="SP_70" refType="secondary-ac" refTypeAc="MI:0360"**/>

<**secondaryRef db="uniprotkb" dbAc="MI:0486" id="Q9NWR2" version="SP_70" refType="secondary-ac" refTypeAc="MI:0360"**/>

<**secondaryRef db="unknown" id="EBI-750109" refType="identity" refTypeAc="MI:0356"**/>

<**secondaryRef db="go" dbAc="MI:0448" id="GO:0005737"**/>

<**secondaryRef db="go" dbAc="MI:0448" id="GO:0000783"**/>

<**secondaryRef db="go" dbAc="MI:0448" id="GO:0005654"**/>

<**secondaryRef db="go" dbAc="MI:0448" id="GO:0048239"**/>

<**secondaryRef db="go" dbAc="MI:0448" id="GO:0032205"**/>

<**secondaryRef db="go" dbAc="MI:0448" id="GO:0043123"**/>

<**secondaryRef db="go" dbAc="MI:0448" id="GO:0051092"**/>

<**secondaryRef db="go" dbAc="MI:0448" id="GO:0031848"**/>

<**secondaryRef db="go" dbAc="MI:0448" id="GO:0070198"**/>

<**secondaryRef db="go" dbAc="MI:0448" id="GO:0010569"**/>

<**secondaryRef db="go" dbAc="MI:0448" id="GO:0007004"**/>

<**secondaryRef db="go" dbAc="MI:0448" id="GO:0006351"**/>

<**secondaryRef db="refseq" dbAc="MI:0481" id="NP_061848.2"**/>

<**secondaryRef db="interpro" dbAc="MI:0449" id="IPR009057"**/>

<**secondaryRef db="interpro" dbAc="MI:0449" id="IPR015010"**/>

<**secondaryRef db="rcsb pdb" dbAc="MI:0460" id="1FEX"**/>

<**secondaryRef db="rcsb pdb" dbAc="MI:0460" id="3K6G"**/>

<**secondaryRef db="go" dbAc="MI:0448" id="GO:0016233"**/>

<**secondaryRef db="go" dbAc="MI:0448" id="GO:0005635"**/>

<**secondaryRef db="go" dbAc="MI:0448" id="GO:0033138"**/>

<**secondaryRef db="go" dbAc="MI:0448" id="GO:1901985"**/>

<**secondaryRef db="interpro" dbAc="MI:0449" id="IPR021661"**/>

<**secondaryRef db="go" dbAc="MI:0448" id="GO:0000228"**/>

<**secondaryRef db="go" dbAc="MI:0448" id="GO:0000723"**/>

<**secondaryRef db="go" dbAc="MI:0448" id="GO:0000781"**/>

<**secondaryRef db="go" dbAc="MI:0448" id="GO:0000784"**/>

<**secondaryRef db="go" dbAc="MI:0448" id="GO:0005634"**/>

<**secondaryRef db="go" dbAc="MI:0448" id="GO:0006355"**/>

<**secondaryRef db="go" dbAc="MI:0448" id="GO:0010833"**/>

<**secondaryRef db="ensembl" dbAc="MI:0476" id="ENST00000300086"**/>

<**secondaryRef db="ensembl" dbAc="MI:0476" id="ENSG00000166848"**/>

<**secondaryRef db="ensembl" dbAc="MI:0476" id="ENSP00000300086"**/>

<**secondaryRef db="go" dbAc="MI:0448" id="GO:0070187"**/>

<**secondaryRef db="reactome" dbAc="MI:0467" id="R-HSA-1221632"**/>

<**secondaryRef db="reactome" dbAc="MI:0467" id="R-HSA-171306"**/>

<**secondaryRef db="reactome" dbAc="MI:0467" id="R-HSA-2559586"**/>

<**secondaryRef db="go" dbAc="MI:0448" id="GO:0042162"**/>

<**secondaryRef db="interpro" dbAc="MI:0449" id="IPR001357"**/>

<**secondaryRef db="go" dbAc="MI:0448" id="GO:0098505"**/>

<**secondaryRef db="rcsb pdb" dbAc="MI:0460" id="4RQI"**/>

</**xref**>

<**interactorType**>

<**names**>

<**shortLabel**>protein</**shortLabel**>

<**fullName**>protein</**fullName**>

</**names**>

<**xref**>

<**primaryRef db="psi-mi" dbAc="MI:0488" id="MI:0326" refType="identity" refTypeAc="MI:0356"**/>

<**secondaryRef db="unknown" id="EBI-619654" refType="identity" refTypeAc="MI:0356"**/>

<**secondaryRef db="pubmed" dbAc="MI:0446" id="14755292" refType="primary-reference" refTypeAc="MI:0358"**/>

<**secondaryRef db="so" dbAc="MI:0601" id="SO:0000358" refType="see-also" refTypeAc="MI:0361"**/>

</**xref**>

</**interactorType**>

<**organism ncbiTaxId="9606"**>

<**names**>

<**shortLabel**>human</**shortLabel**>

<**fullName**>Homo sapiens</**fullName**>

<**alias type="synonym" typeAc="MI:1041"**>Human</**alias**>

</**names**>

</**organism**>

<**sequence**> MAEAMDLGKDPNGPTHSSTLFVRDDGSSMSFYVRPSPAKRRLSTLILHGGGTVCRVQEPGAVLLAQPGEALAEASGDFISTQYILDCVERNERLELEAYRLGPASAADTGSEAKPGALAEGAAEPEPQRHAGRIAFTDADDVAILTYVKENARSPSSVTGNALWKAMEKSSLTQHSWQSLKDRYLKHLRGQEHKYLLGDAPVSPSSQKLKRKAEEDPEAADSGEPQNKRTPDLPEEEYVKEEIQENEEAVKKMLVEATREFEEVVVDESPPDFEIHITMCDDDPPTPEEDSETQPDEEEEEEEEKVSQPEVGAAIKIIRQLMEKFNLDLSTVTQAFLKNSGELEATSAFLASGQRADGYPIWSRQDDIDLQKDDEDTREALVKKFGAQNVARRIEFRKK

</**sequence**>

<**attributeList**>

<**attribute name="crc64"**>EAA615777F9D3D3D</**attribute**>

</**attributeList**>

</**interactor**>

<**interactor id="3"**>

<**names**>

<**shortLabel**>terf1_human</**shortLabel**>

<**fullName**>Telomeric repeat-binding factor 1</**fullName**>

<**alias type="gene name synonym" typeAc="MI:0302"**>TTAGGG repeat-binding factor 1</**alias**>

<**alias type="gene name synonym" typeAc="MI:0302"**>NIMA-interacting protein 2</**alias**>

<**alias type="gene name synonym" typeAc="MI:0302"**>Telomeric protein Pin2/TRF1</**alias**>

<**alias type="gene name" typeAc="MI:0301"**>TERF1</**alias**>

<**alias type="gene name synonym" typeAc="MI:0302"**>PIN2</**alias**>

<**alias type="gene name synonym" typeAc="MI:0302"**>TRF</**alias**>

<**alias type="gene name synonym" typeAc="MI:0302"**>TRF1</**alias**>

<**alias type="gene name synonym" typeAc="MI:0302"**>TRBF1</**alias**>

</**names**>

<**xref**>

<**primaryRef db="uniprotkb" dbAc="MI:0486" id="P54274" version="SP_84" refType="identity" refTypeAc="MI:0356"**/>

<**secondaryRef db="uniprotkb" dbAc="MI:0486" id="Q8NHT6" version="SP_103" refType="secondary-ac" refTypeAc="MI:0360"**/>

<**secondaryRef db="uniprotkb" dbAc="MI:0486" id="A7XP29" version="SP_100" refType="secondary-ac" refTypeAc="MI:0360"**/>

<**secondaryRef db="uniprotkb" dbAc="MI:0486" id="Q15553" version="SP_84" refType="secondary-ac" refTypeAc="MI:0360"**/>

<**secondaryRef db="uniprotkb" dbAc="MI:0486" id="Q93029" version="SP_84" refType="secondary-ac" refTypeAc="MI:0360"**/>

<**secondaryRef db="unknown" id="EBI-710997" refType="identity" refTypeAc="MI:0356"**/>

<**secondaryRef db="rcsb pdb" dbAc="MI:0460" id="5HKP"**/>

<**secondaryRef db="go" dbAc="MI:0448" id="GO:0043065"**/>

<**secondaryRef db="go" dbAc="MI:0448" id="GO:0051974"**/>

<**secondaryRef db="go" dbAc="MI:0448" id="GO:0042493"**/>

<**secondaryRef db="go" dbAc="MI:0448" id="GO:0008017"**/>

<**secondaryRef db="go" dbAc="MI:0448" id="GO:0046982"**/>

<**secondaryRef db="go" dbAc="MI:0448" id="GO:0016233"**/>

<**secondaryRef db="go" dbAc="MI:0448" id="GO:0031627"**/>

<**secondaryRef db="go" dbAc="MI:0448" id="GO:0000723"**/>

<**secondaryRef db="go" dbAc="MI:0448" id="GO:0003677"**/>

<**secondaryRef db="go" dbAc="MI:0448" id="GO:0008156"**/>

<**secondaryRef db="go" dbAc="MI:0448" id="GO:0042803"**/>

<**secondaryRef db="ensembl" dbAc="MI:0476" id="ENST00000276602"**/>

<**secondaryRef db="ensembl" dbAc="MI:0476" id="ENST00000276603"**/>

<**secondaryRef db="go" dbAc="MI:0448" id="GO:0000784"**/>

<**secondaryRef db="go" dbAc="MI:0448" id="GO:0051301"**/>

<**secondaryRef db="go" dbAc="MI:0448" id="GO:0005819"**/>

<**secondaryRef db="go" dbAc="MI:0448" id="GO:0070187"**/>

<**secondaryRef db="reactome" dbAc="MI:0467" id="R-HSA-1221632"**/>

<**secondaryRef db="reactome" dbAc="MI:0467" id="R-HSA-171306"**/>

<**secondaryRef db="reactome" dbAc="MI:0467" id="R-HSA-2559586"**/>

<**secondaryRef db="go" dbAc="MI:0448" id="GO:0045141"**/>

<**secondaryRef db="go" dbAc="MI:0448" id="GO:0043130"**/>

<**secondaryRef db="go" dbAc="MI:0448" id="GO:1904792"**/>

<**secondaryRef db="go" dbAc="MI:0448" id="GO:0005730"**/>

<**secondaryRef db="ensembl" dbAc="MI:0476" id="ENSG00000147601"**/>

<**secondaryRef db="ensembl" dbAc="MI:0476" id="ENSP00000276602"**/>

<**secondaryRef db="ensembl" dbAc="MI:0476" id="ENSP00000276603"**/>

<**secondaryRef db="go" dbAc="MI:0448" id="GO:0045931"**/>

<**secondaryRef db="go" dbAc="MI:0448" id="GO:0051260"**/>

<**secondaryRef db="go" dbAc="MI:0448" id="GO:0007004"**/>

<**secondaryRef db="refseq" dbAc="MI:0481" id="NP_003209.2"**/>

<**secondaryRef db="refseq" dbAc="MI:0481" id="NP_059523.2"**/>

<**secondaryRef db="interpro" dbAc="MI:0449" id="IPR009057"**/>

<**secondaryRef db="interpro" dbAc="MI:0449" id="IPR001005"**/>

<**secondaryRef db="interpro" dbAc="MI:0449" id="IPR017357"**/>

<**secondaryRef db="interpro" dbAc="MI:0449" id="IPR013867"**/>

<**secondaryRef db="interpro" dbAc="MI:0449" id="IPR017930"**/>

<**secondaryRef db="rcsb pdb" dbAc="MI:0460" id="1BA5"**/>

<**secondaryRef db="rcsb pdb" dbAc="MI:0460" id="1H6O"**/>

<**secondaryRef db="rcsb pdb" dbAc="MI:0460" id="1ITY"**/>

<**secondaryRef db="rcsb pdb" dbAc="MI:0460" id="1IV6"**/>

<**secondaryRef db="rcsb pdb" dbAc="MI:0460" id="1W0T"**/>

<**secondaryRef db="rcsb pdb" dbAc="MI:0460" id="3BQO"**/>

<**secondaryRef db="rcsb pdb" dbAc="MI:0460" id="3L82"**/>

<**secondaryRef db="go" dbAc="MI:0448" id="GO:0098505"**/>

<**secondaryRef db="go" dbAc="MI:0448" id="GO:1904850"**/>

<**secondaryRef db="go" dbAc="MI:0448" id="GO:1904911"**/>

<**secondaryRef db="go" dbAc="MI:0448" id="GO:1904914"**/>

<**secondaryRef db="go" dbAc="MI:0448" id="GO:0007094"**/>

<**secondaryRef db="go" dbAc="MI:0448" id="GO:0000781"**/>

<**secondaryRef db="go" dbAc="MI:0448" id="GO:0032214"**/>

<**secondaryRef db="go" dbAc="MI:0448" id="GO:0005737"**/>

<**secondaryRef db="go" dbAc="MI:0448" id="GO:0000783"**/>

<**secondaryRef db="go" dbAc="MI:0448" id="GO:0005654"**/>

<**secondaryRef db="go" dbAc="MI:0448" id="GO:0005634"**/>

<**secondaryRef db="go" dbAc="MI:0448" id="GO:0008301"**/>

<**secondaryRef db="go" dbAc="MI:0448" id="GO:0003691"**/>

<**secondaryRef db="go" dbAc="MI:0448" id="GO:0042162"**/>

<**secondaryRef db="go" dbAc="MI:0448" id="GO:0000086"**/>

<**secondaryRef db="go" dbAc="MI:0448" id="GO:0032211"**/>

<**secondaryRef db="go" dbAc="MI:0448" id="GO:0007067"**/>

<**secondaryRef db="go" dbAc="MI:0448" id="GO:0031116"**/>

<**secondaryRef db="go" dbAc="MI:0448" id="GO:0045840"**/>

</**xref**>

<**interactorType**>

<**names**>

<**shortLabel**>protein</**shortLabel**>

<**fullName**>protein</**fullName**>

</**names**>

<**xref**>

<**primaryRef db="psi-mi" dbAc="MI:0488" id="MI:0326" refType="identity" refTypeAc="MI:0356"**/>

<**secondaryRef db="unknown" id="EBI-619654" refType="identity" refTypeAc="MI:0356"**/>

<**secondaryRef db="pubmed" dbAc="MI:0446" id="14755292" refType="primary-reference" refTypeAc="MI:0358"**/>

<**secondaryRef db="so" dbAc="MI:0601" id="SO:0000358" refType="see-also" refTypeAc="MI:0361"**/>

</**xref**>

</**interactorType**>

<**organism ncbiTaxId="9606"**>

<**names**>

<**shortLabel**>human</**shortLabel**>

<**fullName**>Homo sapiens</**fullName**>

<**alias type="synonym" typeAc="MI:1041"**>Human</**alias**>

</**names**>

</**organism**>

<**sequence**> MAEDVSSAAPSPRGCADGRDADPTEEQMAETERNDEEQFECQELLECQVQVGAPEEEEEEEEDAGLVAEAEAVAAGWMLDFLCLSLCRAFRDGRSEDFRRTRNSAEAIIHGLSSLTACQLRTIYICQFLTRIAAGKTLDAQFENDERITPLESALMIWGSIEKEHDKLHEEIQNLIKIQAIAVCMENGNFKEAEEVFERIFGDPNSHMPFKSKLLMIISQKDTFHSFFQHFSYNHMMEKIKSYVNYVLSEKSSTFLMKAAAKVVESKRTRTITSQDKPSGNDVEMETEANLDTRKSVSDKQSAVTESSEGTVSLLRSHKNLFLSKLQHGTQQQDLNKKERRVGTPQSTKKKKESRRATESRIPVSKSQPVTPEKHRARKRQAWLWEEDKNLRSGVRKYGEGNWSKILLHYKFNNRTSVMLKDRWRTMKKLKLISSDSED

</**sequence**>

<**attributeList**>

<**attribute name="crc64"**>AB548E7D3124A211</**attribute**>

</**attributeList**>

</**interactor**>

<**interactor id="4"**>

<**names**>

<**shortLabel**>acd_human</**shortLabel**>

<**fullName**>Adrenocortical dysplasia protein homolog</**fullName**>

<**alias type="gene name synonym" typeAc="MI:0302"**>POT1 and TIN2-interacting protein</**alias**>

<**alias type="gene name" typeAc="MI:0301"**>ACD</**alias**>

<**alias type="gene name synonym" typeAc="MI:0302"**>PIP1</**alias**>

<**alias type="gene name synonym" typeAc="MI:0302"**>PTOP</**alias**>

<**alias type="gene name synonym" typeAc="MI:0302"**>TINT1</**alias**>

<**alias type="gene name synonym" typeAc="MI:0302"**>TPP1</**alias**>

</**names**>

<**xref**>

<**primaryRef db="uniprotkb" dbAc="MI:0486" id="Q96AP0" version="SP_31" refType="identity" refTypeAc="MI:0356"**/>

<**secondaryRef db="uniprotkb" dbAc="MI:0486" id="Q562H5" version="SP_31" refType="secondary-ac" refTypeAc="MI:0360"**/>

<**secondaryRef db="uniprotkb" dbAc="MI:0486" id="Q9H8F9" version="SP_31" refType="secondary-ac" refTypeAc="MI:0360"**/>

<**secondaryRef db="unknown" id="EBI-717666" refType="identity" refTypeAc="MI:0356"**/>

<**secondaryRef db="go" dbAc="MI:0448" id="GO:0007004"**/>

<**secondaryRef db="ensembl" dbAc="MI:0476" id="ENSG00000102977"**/>

<**secondaryRef db="ensembl" dbAc="MI:0476" id="ENSP00000219251"**/>

<**secondaryRef db="ensembl" dbAc="MI:0476" id="ENSP00000377496"**/>

<**secondaryRef db="ensembl" dbAc="MI:0476" id="ENSP00000483117"**/>

<**secondaryRef db="ensembl" dbAc="MI:0476" id="ENST00000620338"**/>

<**secondaryRef db="go" dbAc="MI:0448" id="GO:0032212"**/>

<**secondaryRef db="go" dbAc="MI:0448" id="GO:0042162"**/>

<**secondaryRef db="go" dbAc="MI:0448" id="GO:0001501"**/>

<**secondaryRef db="go" dbAc="MI:0448" id="GO:0070200"**/>

<**secondaryRef db="go" dbAc="MI:0448" id="GO:0005634"**/>

<**secondaryRef db="go" dbAc="MI:0448" id="GO:0005737"**/>

<**secondaryRef db="go" dbAc="MI:0448" id="GO:0070187"**/>

<**secondaryRef db="reactome" dbAc="MI:0467" id="R-HSA-1221632"**/>

<**secondaryRef db="reactome" dbAc="MI:0467" id="R-HSA-171306"**/>

<**secondaryRef db="reactome" dbAc="MI:0467" id="R-HSA-2559586"**/>

<**secondaryRef db="go" dbAc="MI:0448" id="GO:0000784"**/>

<**secondaryRef db="go" dbAc="MI:0448" id="GO:0044877"**/>

<**secondaryRef db="go" dbAc="MI:0448" id="GO:0000723"**/>

<**secondaryRef db="go" dbAc="MI:0448" id="GO:0016233"**/>

<**secondaryRef db="go" dbAc="MI:0448" id="GO:0070182"**/>

<**secondaryRef db="interpro" dbAc="MI:0449" id="IPR028631"**/>

<**secondaryRef db="ensembl" dbAc="MI:0476" id="ENST00000219251"**/>

<**secondaryRef db="ensembl" dbAc="MI:0476" id="ENST00000393919"**/>

<**secondaryRef db="go" dbAc="MI:0448" id="GO:0000783"**/>

<**secondaryRef db="go" dbAc="MI:0448" id="GO:0005654"**/>

<**secondaryRef db="go" dbAc="MI:0448" id="GO:0006886"**/>

<**secondaryRef db="go" dbAc="MI:0448" id="GO:0032211"**/>

<**secondaryRef db="go" dbAc="MI:0448" id="GO:0060381"**/>

<**secondaryRef db="go" dbAc="MI:0448" id="GO:0051973"**/>

<**secondaryRef db="go" dbAc="MI:0448" id="GO:0031848"**/>

<**secondaryRef db="go" dbAc="MI:0448" id="GO:0070198"**/>

<**secondaryRef db="go" dbAc="MI:0448" id="GO:0032202"**/>

<**secondaryRef db="refseq" dbAc="MI:0481" id="NP_001075955.1"**/>

<**secondaryRef db="refseq" dbAc="MI:0481" id="NP_001075956.1"**/>

<**secondaryRef db="refseq" dbAc="MI:0481" id="NP_075065.2"**/>

<**secondaryRef db="rcsb pdb" dbAc="MI:0460" id="2I46"**/>

<**secondaryRef db="go" dbAc="MI:0448" id="GO:0001655"**/>

<**secondaryRef db="go" dbAc="MI:0448" id="GO:0030326"**/>

<**secondaryRef db="go" dbAc="MI:0448" id="GO:0035282"**/>

<**secondaryRef db="rcsb pdb" dbAc="MI:0460" id="5I2X"**/>

<**secondaryRef db="rcsb pdb" dbAc="MI:0460" id="5I2Y"**/>

</**xref**>

<**interactorType**>

<**names**>

<**shortLabel**>protein</**shortLabel**>

<**fullName**>protein</**fullName**>

</**names**>

<**xref**>

<**primaryRef db="psi-mi" dbAc="MI:0488" id="MI:0326" refType="identity" refTypeAc="MI:0356"**/>

<**secondaryRef db="unknown" id="EBI-619654" refType="identity" refTypeAc="MI:0356"**/>

<**secondaryRef db="pubmed" dbAc="MI:0446" id="14755292" refType="primary-reference" refTypeAc="MI:0358"**/>

<**secondaryRef db="so" dbAc="MI:0601" id="SO:0000358" refType="see-also" refTypeAc="MI:0361"**/>

</**xref**>

</**interactorType**>

<**organism ncbiTaxId="9606"**>

<**names**>

<**shortLabel**>human</**shortLabel**>

<**fullName**>Homo sapiens</**fullName**>

<**alias type="synonym" typeAc="MI:1041"**>Human</**alias**>

</**names**>

</**organism**>

<**sequence**> MPGRCQSDAAMRVNGPASRAPAGWTSGSLHTGPRAGRPRAQARGVRGRGLLLRPRPAKELPLPRKGGAWAPAGNPGPLHPLGVAVGMAGSGRLVLRPWIRELILGSETPSSPRAGQLLEVLQDAEAAVAGPSHAPDTSDVGATLLVSDGTHSVRCLVTREALDTSDWEEKEFGFRGTEGRLLLLQDCGVHVQVAEGGAPAEFYLQVDRFSLLPTEQPRLRVPGCNQDLDVQKKLYDCLEEHLSESTSSNAGLSLSQLLDEMREDQEHQGALVCLAESCLTLEGPCTAPPVTHWAASRCKATGEAVYTVPSSMLCISENDQLILSSLGPCQRTQGPELPPPDPALQDLSLTLIASPPSSPSSSGTPALPGHMSSEESGTSISLLPALSLAAPDPGQRSSSQPSPAICSAPATLTPRSPHASRTPSSPLQSCTPSLSPRSHVPSPHQALVTRPQKPSLEFKEFVGLPCKNRPPFPRTGATRGAQEPCSVWEPPKRHRDGSAFQYEYEPPCTSLCARVQAVRLPPQLMAWALHFLMDAQPGSEPTPM

</**sequence**>

<**attributeList**>

<**attribute name="crc64"**>D2FDF242DA98C483</**attribute**>

</**attributeList**>

</**interactor**>

<**interactor id="5"**>

<**names**>

<**shortLabel**>pote1_human</**shortLabel**>

<**fullName**>Protection of telomeres protein 1</**fullName**>

<**alias type="gene name synonym" typeAc="MI:0302"**>POT1-like telomere end-binding protein</**alias**>

<**alias type="gene name" typeAc="MI:0301"**>POT1</**alias**>

</**names**>

<**xref**>

<**primaryRef db="uniprotkb" dbAc="MI:0486" id="Q9NUX5" version="SP_57" refType="identity" refTypeAc="MI:0356"**/>

<**secondaryRef db="uniprotkb" dbAc="MI:0486" id="Q5MJ36" version="SP_75" refType="secondary-ac" refTypeAc="MI:0360"**/>

<**secondaryRef db="uniprotkb" dbAc="MI:0486" id="O95018" version="SP_57" refType="secondary-ac" refTypeAc="MI:0360"**/>

<**secondaryRef db="uniprotkb" dbAc="MI:0486" id="Q9H662" version="SP_57" refType="secondary-ac" refTypeAc="MI:0360"**/>

<**secondaryRef db="uniprotkb" dbAc="MI:0486" id="Q9NW19" version="SP_57" refType="secondary-ac" refTypeAc="MI:0360"**/>

<**secondaryRef db="uniprotkb" dbAc="MI:0486" id="Q9UG95" version="SP_57" refType="secondary-ac" refTypeAc="MI:0360"**/>

<**secondaryRef db="unknown" id="EBI-752420" refType="identity" refTypeAc="MI:0356"**/>

<**secondaryRef db="go" dbAc="MI:0448" id="GO:0005634"**/>

<**secondaryRef db="refseq" dbAc="MI:0481" id="XP_006715980.1"**/>

<**secondaryRef db="go" dbAc="MI:0448" id="GO:0032202"**/>

<**secondaryRef db="go" dbAc="MI:0448" id="GO:0000781"**/>

<**secondaryRef db="go" dbAc="MI:0448" id="GO:0017151"**/>

<**secondaryRef db="go" dbAc="MI:0448" id="GO:0051974"**/>

<**secondaryRef db="ensembl" dbAc="MI:0476" id="ENST00000357628"**/>

<**secondaryRef db="ensembl" dbAc="MI:0476" id="ENSG00000128513"**/>

<**secondaryRef db="ensembl" dbAc="MI:0476" id="ENSP00000350249"**/>

<**secondaryRef db="go" dbAc="MI:0448" id="GO:0070187"**/>

<**secondaryRef db="go" dbAc="MI:0448" id="GO:0098505"**/>

<**secondaryRef db="reactome" dbAc="MI:0467" id="R-HSA-1221632"**/>

<**secondaryRef db="reactome" dbAc="MI:0467" id="R-HSA-171306"**/>

<**secondaryRef db="reactome" dbAc="MI:0467" id="R-HSA-2559586"**/>

<**secondaryRef db="go" dbAc="MI:0448" id="GO:0000784"**/>

<**secondaryRef db="go" dbAc="MI:0448" id="GO:0042162"**/>

<**secondaryRef db="interpro" dbAc="MI:0449" id="IPR032042"**/>

<**secondaryRef db="go" dbAc="MI:0448" id="GO:0070200"**/>

<**secondaryRef db="go" dbAc="MI:0448" id="GO:0000783"**/>

<**secondaryRef db="go" dbAc="MI:0448" id="GO:0005654"**/>

<**secondaryRef db="go" dbAc="MI:0448" id="GO:0043047"**/>

<**secondaryRef db="go" dbAc="MI:0448" id="GO:0010521"**/>

<**secondaryRef db="go" dbAc="MI:0448" id="GO:0032508"**/>

<**secondaryRef db="go" dbAc="MI:0448" id="GO:0060383"**/>

<**secondaryRef db="go" dbAc="MI:0448" id="GO:0051096"**/>

<**secondaryRef db="go" dbAc="MI:0448" id="GO:0051973"**/>

<**secondaryRef db="go" dbAc="MI:0448" id="GO:0032212"**/>

<**secondaryRef db="go" dbAc="MI:0448" id="GO:0016233"**/>

<**secondaryRef db="go" dbAc="MI:0448" id="GO:0007004"**/>

<**secondaryRef db="refseq" dbAc="MI:0481" id="NP_001036059.1"**/>

<**secondaryRef db="refseq" dbAc="MI:0481" id="NP_056265.2"**/>

<**secondaryRef db="interpro" dbAc="MI:0449" id="IPR012340"**/>

<**secondaryRef db="interpro" dbAc="MI:0449" id="IPR011564"**/>

<**secondaryRef db="rcsb pdb" dbAc="MI:0460" id="1XJV"**/>

<**secondaryRef db="rcsb pdb" dbAc="MI:0460" id="3KJO"**/>

<**secondaryRef db="rcsb pdb" dbAc="MI:0460" id="3KJP"**/>

<**secondaryRef db="interpro" dbAc="MI:0449" id="IPR028389"**/>

</**xref**>

<**interactorType**>

<**names**>

<**shortLabel**>protein</**shortLabel**>

<**fullName**>protein</**fullName**>

</**names**>

<**xref**>

<**primaryRef db="psi-mi" dbAc="MI:0488" id="MI:0326" refType="identity" refTypeAc="MI:0356"**/>

<**secondaryRef db="unknown" id="EBI-619654" refType="identity" refTypeAc="MI:0356"**/>

<**secondaryRef db="pubmed" dbAc="MI:0446" id="14755292" refType="primary-reference" refTypeAc="MI:0358"**/>

<**secondaryRef db="so" dbAc="MI:0601" id="SO:0000358" refType="see-also" refTypeAc="MI:0361"**/>

</**xref**>

</**interactorType**>

<**organism ncbiTaxId="9606"**>

<**names**>

<**shortLabel**>human</**shortLabel**>

<**fullName**>Homo sapiens</**fullName**>

<**alias type="synonym" typeAc="MI:1041"**>Human</**alias**>

</**names**>

</**organism**>

<**sequence**> MSLVPATNYIYTPLNQLKGGTIVNVYGVVKFFKPPYLSKGTDYCSVVTIVDQTNVKLTCLLFSGNYEALPIIYKNGDIVRFHRLKIQVYKKETQGITSSGFASLTFEGTLGAPIIPRTSSKYFNFTTEDHKMVEALRVWASTHMSPSWTLLKLCDVQPMQYFDLTCQLLGKAEVDGASFLLKVWDGTRTPFPSWRVLIQDLVLEGDLSHIHRLQNLTIDILVYDNHVHVARSLKVGSFLRIYSLHTKLQSMNSENQTMLSLEFHLHGGTSYGRGIRVLPESNSDVDQLKKDLESANLTANQHSDVICQSEPDDSFPSSGSVSLYEVERCQQLSATILTDHQYLERTPLCAILKQKAPQQYRIRAKLRSYKPRRLFQSVKLHCPKCHLLQEVPHEGDLDIIFQDGATKTPDVKLQNTSLYDSKIWTTKNQKGRKVAVHFVKNNGILPLSNECLLLIEGGTLSEICKLSNKFNSVIPVRSGHEDLELLDLSAPFLIQGTIHHYGCKQCSSLRSIQNLNSLVDKTSWIPSSVAEALGIVPLQYVFVMTFTLDDGTGVLEAYLMDSDKFFQIPASEVLMDDDLQKSVDMIMDMFCPPGIKIDAYPWLECFIKSYNVTNGTDNQICYQIFDTTVAEDVI

</**sequence**>

<**attributeList**>

<**attribute name="crc64"**>123A12CABE708C91</**attribute**>

</**attributeList**>

</**interactor**>

<**interactor id="6"**>

<**names**>

<**shortLabel**>terf2_human</**shortLabel**>

<**fullName**>Telomeric repeat-binding factor 2</**fullName**>

<**alias type="gene name synonym" typeAc="MI:0302"**>TTAGGG repeat-binding factor 2</**alias**>

<**alias type="gene name synonym" typeAc="MI:0302"**>Telomeric DNA-binding protein</**alias**>

<**alias type="gene name" typeAc="MI:0301"**>TERF2</**alias**>

<**alias type="gene name synonym" typeAc="MI:0302"**>TRF2</**alias**>

<**alias type="gene name synonym" typeAc="MI:0302"**>TRBF2</**alias**>

</**names**>

<**xref**>

<**primaryRef db="uniprotkb" dbAc="MI:0486" id="Q15554" version="SP_76" refType="identity" refTypeAc="MI:0356"**/>

<**secondaryRef db="intact" dbAc="MI:0469" id="EBI-706651" refType="intact-secondary"**/>

<**secondaryRef db="unknown" id="EBI-706637" refType="identity" refTypeAc="MI:0356"**/>

<**secondaryRef db="go" dbAc="MI:0448" id="GO:0032208"**/>

<**secondaryRef db="go" dbAc="MI:0448" id="GO:0032210"**/>

<**secondaryRef db="go" dbAc="MI:0448" id="GO:0098505"**/>

<**secondaryRef db="go" dbAc="MI:0448" id="GO:0000783"**/>

<**secondaryRef db="go" dbAc="MI:0448" id="GO:0005654"**/>

<**secondaryRef db="go" dbAc="MI:0448" id="GO:0003691"**/>

<**secondaryRef db="go" dbAc="MI:0448" id="GO:0007049"**/>

<**secondaryRef db="go" dbAc="MI:0448" id="GO:0090398"**/>

<**secondaryRef db="go" dbAc="MI:0448" id="GO:0032214"**/>

<**secondaryRef db="go" dbAc="MI:0448" id="GO:0031848"**/>

<**secondaryRef db="go" dbAc="MI:0448" id="GO:0070198"**/>

<**secondaryRef db="go" dbAc="MI:0448" id="GO:0007004"**/>

<**secondaryRef db="go" dbAc="MI:0448" id="GO:0031627"**/>

<**secondaryRef db="interpro" dbAc="MI:0449" id="IPR009057"**/>

<**secondaryRef db="interpro" dbAc="MI:0449" id="IPR001005"**/>

<**secondaryRef db="interpro" dbAc="MI:0449" id="IPR013867"**/>

<**secondaryRef db="interpro" dbAc="MI:0449" id="IPR017930"**/>

<**secondaryRef db="rcsb pdb" dbAc="MI:0460" id="1H6P"**/>

<**secondaryRef db="rcsb pdb" dbAc="MI:0460" id="1VF9"**/>

<**secondaryRef db="rcsb pdb" dbAc="MI:0460" id="1VFC"**/>

<**secondaryRef db="rcsb pdb" dbAc="MI:0460" id="1W0U"**/>

<**secondaryRef db="rcsb pdb" dbAc="MI:0460" id="1XG1"**/>

<**secondaryRef db="rcsb pdb" dbAc="MI:0460" id="3BU8"**/>

<**secondaryRef db="rcsb pdb" dbAc="MI:0460" id="3BUA"**/>

<**secondaryRef db="rcsb pdb" dbAc="MI:0460" id="3K6G"**/>

<**secondaryRef db="go" dbAc="MI:0448" id="GO:0005737"**/>

<**secondaryRef db="go" dbAc="MI:0448" id="GO:0005794"**/>

<**secondaryRef db="go" dbAc="MI:0448" id="GO:0010628"**/>

<**secondaryRef db="go" dbAc="MI:0448" id="GO:0010629"**/>

<**secondaryRef db="go" dbAc="MI:0448" id="GO:0051000"**/>

<**secondaryRef db="go" dbAc="MI:0448" id="GO:2000773"**/>

<**secondaryRef db="rcsb pdb" dbAc="MI:0460" id="4M7C"**/>

<**secondaryRef db="go" dbAc="MI:0448" id="GO:0044877"**/>

<**secondaryRef db="go" dbAc="MI:0448" id="GO:1904430"**/>

<**secondaryRef db="go" dbAc="MI:0448" id="GO:0032204"**/>

<**secondaryRef db="interpro" dbAc="MI:0449" id="IPR030657"**/>

<**secondaryRef db="rcsb pdb" dbAc="MI:0460" id="3SJM"**/>

<**secondaryRef db="refseq" dbAc="MI:0481" id="NP_005643.2"**/>

<**secondaryRef db="go" dbAc="MI:0448" id="GO:0070187"**/>

<**secondaryRef db="reactome" dbAc="MI:0467" id="R-HSA-1221632"**/>

<**secondaryRef db="reactome" dbAc="MI:0467" id="R-HSA-171306"**/>

<**secondaryRef db="reactome" dbAc="MI:0467" id="R-HSA-2559586"**/>

<**secondaryRef db="go" dbAc="MI:0448" id="GO:0000723"**/>

<**secondaryRef db="go" dbAc="MI:0448" id="GO:0000781"**/>

<**secondaryRef db="go" dbAc="MI:0448" id="GO:0005634"**/>

<**secondaryRef db="go" dbAc="MI:0448" id="GO:0008022"**/>

<**secondaryRef db="go" dbAc="MI:0448" id="GO:0016233"**/>

<**secondaryRef db="go" dbAc="MI:0448" id="GO:0032205"**/>

<**secondaryRef db="go" dbAc="MI:0448" id="GO:0042162"**/>

<**secondaryRef db="go" dbAc="MI:0448" id="GO:0042803"**/>

<**secondaryRef db="ensembl" dbAc="MI:0476" id="ENST00000254942"**/>

<**secondaryRef db="ensembl" dbAc="MI:0476" id="ENSP00000454955"**/>

<**secondaryRef db="go" dbAc="MI:0448" id="GO:0032206"**/>

<**secondaryRef db="ensembl" dbAc="MI:0476" id="ENST00000567296"**/>

<**secondaryRef db="go" dbAc="MI:0448" id="GO:0000784"**/>

<**secondaryRef db="go" dbAc="MI:0448" id="GO:1903770"**/>

<**secondaryRef db="go" dbAc="MI:0448" id="GO:1903824"**/>

<**secondaryRef db="go" dbAc="MI:0448" id="GO:1904354"**/>

<**secondaryRef db="interpro" dbAc="MI:0449" id="IPR031902"**/>

<**secondaryRef db="rcsb pdb" dbAc="MI:0460" id="4RQI"**/>

<**secondaryRef db="ensembl" dbAc="MI:0476" id="ENSG00000132604"**/>

<**secondaryRef db="ensembl" dbAc="MI:0476" id="ENSP00000254942"**/>

</**xref**>

<**interactorType**>

<**names**>

<**shortLabel**>protein</**shortLabel**>

<**fullName**>protein</**fullName**>

</**names**>

<**xref**>

<**primaryRef db="psi-mi" dbAc="MI:0488" id="MI:0326" refType="identity" refTypeAc="MI:0356"**/>

<**secondaryRef db="unknown" id="EBI-619654" refType="identity" refTypeAc="MI:0356"**/>

<**secondaryRef db="pubmed" dbAc="MI:0446" id="14755292" refType="primary-reference" refTypeAc="MI:0358"**/>

<**secondaryRef db="so" dbAc="MI:0601" id="SO:0000358" refType="see-also" refTypeAc="MI:0361"**/>

</**xref**>

</**interactorType**>

<**organism ncbiTaxId="9606"**>

<**names**>

<**shortLabel**>human</**shortLabel**>

<**fullName**>Homo sapiens</**fullName**>

<**alias type="synonym" typeAc="MI:1041"**>Human</**alias**>

</**names**>

</**organism**>

<**sequence**> MAAGAGTAGPASGPGVVRDPAASQPRKRPGREGGEGARRSDTMAGGGGSSDGSGRAAGRRASRSSGRARRGRHEPGLGGPAERGAGEARLEEAVNRWVLKFYFHEALRAFRGSRYGDFRQIRDIMQALLVRPLGKEHTVSRLLRVMQCLSRIEEGENLDCSFDMEAELTPLESAINVLEMIKTEFTLTEAVVESSRKLVKEAAVIICIKNKEFEKASKILKKHMSKDPTTQKLRNDLLNIIREKNLAHPVIQNFSYETFQQKMLRFLESHLDDAEPYLLTMAKKALKSESAASSTGKEDKQPAPGPVEKPPREPARQLRNPPTTIGMMTLKAAFKTLSGAQDSEAAFAKLDQKDLVLPTQALPASPALKNKRPRKDENESSAPADGEGGSELQPKNKRMTISRLVLEEDSQSTEPSAGLNSSQEAASAPPSKPTVLNQPLPGEKNPKVPKGKWNSSNGVEEKETWVEEDELFQVQAAPDEDSTTNITKKQKWTVEESEWVKAGVQKYGEGNWAAISKNYPFVNRTAVMIKDRWRTMKRLGMN

</**sequence**>

<**attributeList**>

<**attribute name="crc64"**>3A278AC6B594C43A</**attribute**>

</**attributeList**>

</**interactor**>

<**interactor id="7"**>

<**names**>

<**shortLabel**>tinf2_human</**shortLabel**>

<**fullName**>TERF1-interacting nuclear factor 2</**fullName**>

<**alias type="gene name synonym" typeAc="MI:0302"**>TRF1-interacting nuclear protein 2</**alias**>

<**alias type="gene name" typeAc="MI:0301"**>TINF2</**alias**>

<**alias type="gene name synonym" typeAc="MI:0302"**>TIN2</**alias**>

</**names**>

<**xref**>

<**primaryRef db="uniprotkb" dbAc="MI:0486" id="Q9BSI4" version="SP_54" refType="identity" refTypeAc="MI:0356"**/>

<**secondaryRef db="uniprotkb" dbAc="MI:0486" id="B3W5Q7" version="SP_118" refType="secondary-ac" refTypeAc="MI:0360"**/>

<**secondaryRef db="uniprotkb" dbAc="MI:0486" id="Q9H904" version="SP_54" refType="secondary-ac" refTypeAc="MI:0360"**/>

<**secondaryRef db="uniprotkb" dbAc="MI:0486" id="Q9UHC2" version="SP_54" refType="secondary-ac" refTypeAc="MI:0360"**/>

<**secondaryRef db="unknown" id="EBI-717399" refType="identity" refTypeAc="MI:0356"**/>

<**secondaryRef db="go" dbAc="MI:0448" id="GO:0016233"**/>

<**secondaryRef db="go" dbAc="MI:0448" id="GO:0000781"**/>

<**secondaryRef db="go" dbAc="MI:0448" id="GO:0034502"**/>

<**secondaryRef db="interpro" dbAc="MI:0449" id="IPR029400"**/>

<**secondaryRef db="ensembl" dbAc="MI:0476" id="ENST00000267415"**/>

<**secondaryRef db="ensembl" dbAc="MI:0476" id="ENST00000399423"**/>

<**secondaryRef db="ensembl" dbAc="MI:0476" id="ENSG00000092330"**/>

<**secondaryRef db="ensembl" dbAc="MI:0476" id="ENSP00000267415"**/>

<**secondaryRef db="ensembl" dbAc="MI:0476" id="ENSP00000382350"**/>

<**secondaryRef db="go" dbAc="MI:0448" id="GO:0005634"**/>

<**secondaryRef db="go" dbAc="MI:0448" id="GO:0010521"**/>

<**secondaryRef db="go" dbAc="MI:0448" id="GO:0051974"**/>

<**secondaryRef db="go" dbAc="MI:0448" id="GO:0016363"**/>

<**secondaryRef db="go" dbAc="MI:0448" id="GO:0070187"**/>

<**secondaryRef db="reactome" dbAc="MI:0467" id="R-HSA-1221632"**/>

<**secondaryRef db="reactome" dbAc="MI:0467" id="R-HSA-171306"**/>

<**secondaryRef db="reactome" dbAc="MI:0467" id="R-HSA-2559586"**/>

<**secondaryRef db="go" dbAc="MI:0448" id="GO:0000784"**/>

<**secondaryRef db="go" dbAc="MI:0448" id="GO:0000783"**/>

<**secondaryRef db="go" dbAc="MI:0448" id="GO:0005654"**/>

<**secondaryRef db="go" dbAc="MI:0448" id="GO:0010370"**/>

<**secondaryRef db="go" dbAc="MI:0448" id="GO:0042162"**/>

<**secondaryRef db="go" dbAc="MI:0448" id="GO:0050680"**/>

<**secondaryRef db="go" dbAc="MI:0448" id="GO:0010836"**/>

<**secondaryRef db="go" dbAc="MI:0448" id="GO:0032211"**/>

<**secondaryRef db="go" dbAc="MI:0448" id="GO:0032206"**/>

<**secondaryRef db="go" dbAc="MI:0448" id="GO:0070198"**/>

<**secondaryRef db="go" dbAc="MI:0448" id="GO:0032202"**/>

<**secondaryRef db="go" dbAc="MI:0448" id="GO:0010833"**/>

<**secondaryRef db="refseq" dbAc="MI:0481" id="NP_001092744.1"**/>

<**secondaryRef db="refseq" dbAc="MI:0481" id="NP_036593.2"**/>

<**secondaryRef db="rcsb pdb" dbAc="MI:0460" id="3BQO"**/>

<**secondaryRef db="rcsb pdb" dbAc="MI:0460" id="3BU8"**/>

</**xref**>

<**interactorType**>

<**names**>

<**shortLabel**>protein</**shortLabel**>

<**fullName**>protein</**fullName**>

</**names**>

<**xref**>

<**primaryRef db="psi-mi" dbAc="MI:0488" id="MI:0326" refType="identity" refTypeAc="MI:0356"**/>

<**secondaryRef db="unknown" id="EBI-619654" refType="identity" refTypeAc="MI:0356"**/>

<**secondaryRef db="pubmed" dbAc="MI:0446" id="14755292" refType="primary-reference" refTypeAc="MI:0358"**/>

<**secondaryRef db="so" dbAc="MI:0601" id="SO:0000358" refType="see-also" refTypeAc="MI:0361"**/>

</**xref**>

</**interactorType**>

<**organism ncbiTaxId="9606"**>

<**names**>

<**shortLabel**>human</**shortLabel**>

<**fullName**>Homo sapiens</**fullName**>

<**alias type="synonym" typeAc="MI:1041"**>Human</**alias**>

</**names**>

</**organism**>

<**sequence**> MATPLVAGPAALRFAAAASWQVVRGRCVEHFPRVLEFLRSLRAVAPGLVRYRHHERLCMGLKAKVVVELILQGRPWAQVLKALNHHFPESGPIVRDPKATKQDLRKILEAQETFYQQVKQLSEAPVDLASKLQELEQEYGEPFLAAMEKLLFEYLCQLEKALPTPQAQQLQDVLSWMQPGVSITSSLAWRQYGVDMGWLLPECSVTDSVNLAEPMEQNPPQQQRLALHNPLPKAKPGTHLPQGPSSRTHPEPLAGRHFNLAPLGRRRVQSQWASTRGGHKERPTVMLFPFRNLGSPTQVISKPESKEEHAIYTADLAMGTRAASTGKSKSPCQTLGGRALKENPVDLPATEQKENCLDCYMDPLRLSLLPPRARKPVCPPSLCSSVITIGDLVLDSDEEENGQGEGKESLENYQKTKFDTLIPTLCEYLPPSGHGAIPVSSCDCRDSSRPL

</**sequence**>

<**attributeList**>

<**attribute name="crc64"**>E5A7FD11CE523979</**attribute**>

</**attributeList**>

</**interactor**>

</**interactorList**>

<**interactionList**>

<**abstractInteraction id="8"**>

<**names**>

<**shortLabel**>shelterin_human</**shortLabel**>

<**alias type="synonym" typeAc="MI:1041"**>TPP1:POT1:RAP1:TRF1:TRF2:TIN2 complex</**alias**>

<**alias type="synonym" typeAc="MI:1041"**>Telosome complex</**alias**>

<**alias type="complex recommended name" typeAc="MI:1315"**>Shelterin complex</**alias**>

<**alias type="complex systematic name" typeAc="MI:1316"**>ACD:POT1:TERF2IP:2xTERF1:2xTERF2:TINF2</**alias**>

<**alias type="synonym" typeAc="MI:1041"**>TPP1:POTE1:TE2IP:TRF1:TRF2:TIN2 complex</**alias**>

</**names**><**xref**><**primaryRef db="reactome" dbAc="MI:0467" id="R-HSA-174898" refType="identity" refTypeAc="MI:0356"**/>

<**secondaryRef db="protein ontology" dbAc="MI:1347" id="PR:000028433" refType="identity" refTypeAc="MI:0356"**/>

<**secondaryRef db="unknown" id="EBI-10887677" refType="identity" refTypeAc="MI:0356"**/>

<**secondaryRef db="pubmed" dbAc="MI:0446" id="15383534" refType="see-also" refTypeAc="MI:0361"**/>

<**secondaryRef db="intact" dbAc="MI:0469" id="EBI-765004" refType="exp-evidence"**/>

<**secondaryRef db="wwpdb" dbAc="MI:0805" id="3BQO" refType="subset" refTypeAc="MI:2179"**/>

<**secondaryRef db="wwpdb" dbAc="MI:0805" id="3K6G" refType="subset" refTypeAc="MI:2179"**/>

<**secondaryRef db="wwpdb" dbAc="MI:0805" id="3BU8" refType="subset" refTypeAc="MI:2179"**/>

<**secondaryRef db="pubmed" dbAc="MI:0446" id="21852327" refType="see-also" refTypeAc="MI:0361"**/>

<**secondaryRef db="go" dbAc="MI:0448" id="GO:0016233" refType="process" refTypeAc="MI:0359"**/>

<**secondaryRef db="go" dbAc="MI:0448" id="GO:0070187" refType="component" refTypeAc="MI:0354"**/>

<**secondaryRef db="pubmed" dbAc="MI:0446" id="23299958" refType="see-also" refTypeAc="MI:0361"**/>

<**secondaryRef db="evidence ontology" dbAc="MI:1331" id="ECO:0000353"**/>

<**secondaryRef db="pubmed" dbAc="MI:0446" id="22965356" refType="see-also" refTypeAc="MI:0361"**/>

<**secondaryRef db="go" dbAc="MI:0448" id="GO:0090655" refType="function" refTypeAc="MI:0355"**/>

<**secondaryRef db="go" dbAc="MI:0448" id="GO:0032206" refType="process" refTypeAc="MI:0359"**/>

<**secondaryRef db="pubmed" dbAc="MI:0446" id="21346783" refType="see-also" refTypeAc="MI:0361"**/>

<**secondaryRef db="pubmed" dbAc="MI:0446" id="20125188" refType="see-also" refTypeAc="MI:0361"**/>

<**secondaryRef db="wwpdb" dbAc="MI:0805" id="1h6p" refType="subset" refTypeAc="MI:2179"**/>

<**secondaryRef db="wwpdb" dbAc="MI:0805" id="1H6O" refType="subset" refTypeAc="MI:2179"**/>

</**xref**>

<**participantList**><**participant id="9"**>

<**interactorRef**>6</**interactorRef**>

<**biologicalRole**>

<**names**>

<**shortLabel**>unspecified role</**shortLabel**>

<**fullName**>unspecified role</**fullName**>

</**names**>

<**xref**>

<**primaryRef db="psi-mi" dbAc="MI:0488" id="MI:0499" refType="identity" refTypeAc="MI:0356"**/>

<**secondaryRef db="unknown" id="EBI-77781" refType="identity" refTypeAc="MI:0356"**/>

<**secondaryRef db="pubmed" dbAc="MI:0446" id="14755292" refType="primary-reference" refTypeAc="MI:0358"**/>

</**xref**>

</**biologicalRole**>

<**featureList**>

<**feature id="10"**>

<**names**>

<**shortLabel**>terf2ip binding region</**shortLabel**>

</**names**>

<**xref**>

<**primaryRef db="unknown" id="EBI-10888364" refType="identity" refTypeAc="MI:0356"**/>

</**xref**>

<**featureType**>

<**names**>

<**shortLabel**>binding region</**shortLabel**>

<**fullName**>binding-associated region</**fullName**>

</**names**>

<**xref**>

<**primaryRef db="psi-mi" dbAc="MI:0488" id="MI:0117" refType="identity" refTypeAc="MI:0356"**/>

<**secondaryRef db="unknown" id="EBI-456493" refType="identity" refTypeAc="MI:0356"**/>

<**secondaryRef db="pubmed" dbAc="MI:0446" id="14755292" refType="primary-reference" refTypeAc="MI:0358"**/>

</**xref**>

</**featureType**>

<**featureRangeList**>

<**featureRange**>

<**startStatus**>

<**names**>

<**shortLabel**>certain</**shortLabel**>

<**fullName**>certain sequence position</**fullName**>

<**alias type="synonym" typeAc="MI:1041"**>certain</**alias**>

</**names**>

<**xref**>

<**primaryRef db="psi-mi" dbAc="MI:0488" id="MI:0335" refType="identity" refTypeAc="MI:0356"**/>

<**secondaryRef db="unknown" id="EBI-540564" refType="identity" refTypeAc="MI:0356"**/>

<**secondaryRef db="pubmed" dbAc="MI:0446" id="14755292" refType="primary-reference" refTypeAc="MI:0358"**/>

</**xref**>

</**startStatus**>

<**begin position="323"**/>

<**endStatus**>

<**names**>

<**shortLabel**>certain</**shortLabel**>

<**fullName**>certain sequence position</**fullName**>

<**alias type="synonym" typeAc="MI:1041"**>certain</**alias**>

</**names**>

<**xref**>

<**primaryRef db="psi-mi" dbAc="MI:0488" id="MI:0335" refType="identity" refTypeAc="MI:0356"**/>

<**secondaryRef db="unknown" id="EBI-540564" refType="identity" refTypeAc="MI:0356"**/>

<**secondaryRef db="pubmed" dbAc="MI:0446" id="14755292" refType="primary-reference" refTypeAc="MI:0358"**/>

</**xref**>

</**endStatus**>

<**end position="355"**/>

</**featureRange**>

</**featureRangeList**>

</**feature**>

<**feature id="11"**>

<**names**>

<**shortLabel**>tinf2 binding region</**shortLabel**>

</**names**>

<**xref**>

<**primaryRef db="unknown" id="EBI-10888358" refType="identity" refTypeAc="MI:0356"**/>

</**xref**>

<**featureType**>

<**names**>

<**shortLabel**>binding region</**shortLabel**>

<**fullName**>binding-associated region</**fullName**>

</**names**>

<**xref**>

<**primaryRef db="psi-mi" dbAc="MI:0488" id="MI:0117" refType="identity" refTypeAc="MI:0356"**/>

<**secondaryRef db="unknown" id="EBI-456493" refType="identity" refTypeAc="MI:0356"**/>

<**secondaryRef db="pubmed" dbAc="MI:0446" id="14755292" refType="primary-reference" refTypeAc="MI:0358"**/>

</**xref**>

</**featureType**>

<**featureRangeList**>

<**featureRange**>

<**startStatus**>

<**names**>

<**shortLabel**>certain</**shortLabel**>

<**fullName**>certain sequence position</**fullName**>

<**alias type="synonym" typeAc="MI:1041"**>certain</**alias**>

</**names**>

<**xref**>

<**primaryRef db="psi-mi" dbAc="MI:0488" id="MI:0335" refType="identity" refTypeAc="MI:0356"**/>

<**secondaryRef db="unknown" id="EBI-540564" refType="identity" refTypeAc="MI:0356"**/>

<**secondaryRef db="pubmed" dbAc="MI:0446" id="14755292" refType="primary-reference" refTypeAc="MI:0358"**/>

</**xref**>

</**startStatus**>

<**begin position="86"**/>

<**endStatus**>

<**names**>

<**shortLabel**>certain</**shortLabel**>

<**fullName**>certain sequence position</**fullName**>

<**alias type="synonym" typeAc="MI:1041"**>certain</**alias**>

</**names**>

<**xref**>

<**primaryRef db="psi-mi" dbAc="MI:0488" id="MI:0335" refType="identity" refTypeAc="MI:0356"**/>

<**secondaryRef db="unknown" id="EBI-540564" refType="identity" refTypeAc="MI:0356"**/>

<**secondaryRef db="pubmed" dbAc="MI:0446" id="14755292" refType="primary-reference" refTypeAc="MI:0358"**/>

</**xref**>

</**endStatus**>

<**end position="289"**/>

</**featureRange**>

</**featureRangeList**>

</**feature**>

<**feature id="12"**>

<**names**>

<**shortLabel**>terf2 binding region</**shortLabel**>

</**names**>

<**xref**>

<**primaryRef db="unknown" id="EBI-10897642" refType="identity" refTypeAc="MI:0356"**/>

</**xref**>

<**featureType**>

<**names**>

<**shortLabel**>binding region</**shortLabel**>

<**fullName**>binding-associated region</**fullName**>

</**names**>

<**xref**>

<**primaryRef db="psi-mi" dbAc="MI:0488" id="MI:0117" refType="identity" refTypeAc="MI:0356"**/>

<**secondaryRef db="unknown" id="EBI-456493" refType="identity" refTypeAc="MI:0356"**/>

<**secondaryRef db="pubmed" dbAc="MI:0446" id="14755292" refType="primary-reference" refTypeAc="MI:0358"**/>

</**xref**>

</**featureType**>

<**featureRangeList**>

<**featureRange**>

<**startStatus**>

<**names**>

<**shortLabel**>certain</**shortLabel**>

<**fullName**>certain sequence position</**fullName**>

<**alias type="synonym" typeAc="MI:1041"**>certain</**alias**>

</**names**>

<**xref**>

<**primaryRef db="psi-mi" dbAc="MI:0488" id="MI:0335" refType="identity" refTypeAc="MI:0356"**/>

<**secondaryRef db="unknown" id="EBI-540564" refType="identity" refTypeAc="MI:0356"**/>

<**secondaryRef db="pubmed" dbAc="MI:0446" id="14755292" refType="primary-reference" refTypeAc="MI:0358"**/>

</**xref**>

</**startStatus**>

<**begin position="85"**/>

<**endStatus**>

<**names**>

<**shortLabel**>certain</**shortLabel**>

<**fullName**>certain sequence position</**fullName**>

<**alias type="synonym" typeAc="MI:1041"**>certain</**alias**>

</**names**>

<**xref**>

<**primaryRef db="psi-mi" dbAc="MI:0488" id="MI:0335" refType="identity" refTypeAc="MI:0356"**/>

<**secondaryRef db="unknown" id="EBI-540564" refType="identity" refTypeAc="MI:0356"**/>

<**secondaryRef db="pubmed" dbAc="MI:0446" id="14755292" refType="primary-reference" refTypeAc="MI:0358"**/>

</**xref**>

</**endStatus**>

<**end position="287"**/>

</**featureRange**>

</**featureRangeList**>

</**feature**>

</**featureList**>

<**stoichiometry value="1"**/>

</**participant**>

<**participant id="13"**>

<**interactorRef**>5</**interactorRef**>

<**biologicalRole**>

<**names**>

<**shortLabel**>unspecified role</**shortLabel**>

<**fullName**>unspecified role</**fullName**>

</**names**>

<**xref**>

<**primaryRef db="psi-mi" dbAc="MI:0488" id="MI:0499" refType="identity" refTypeAc="MI:0356"**/>

<**secondaryRef db="unknown" id="EBI-77781" refType="identity" refTypeAc="MI:0356"**/>

<**secondaryRef db="pubmed" dbAc="MI:0446" id="14755292" refType="primary-reference" refTypeAc="MI:0358"**/>

</**xref**>

</**biologicalRole**>

<**featureList**>

<**feature id="14"**>

<**names**>

<**shortLabel**>acd binding region</**shortLabel**>

</**names**>

<**xref**>

<**primaryRef db="unknown" id="EBI-10897580" refType="identity" refTypeAc="MI:0356"**/>

</**xref**>

<**featureType**>

<**names**>

<**shortLabel**>binding region</**shortLabel**>

<**fullName**>binding-associated region</**fullName**>

</**names**>

<**xref**>

<**primaryRef db="psi-mi" dbAc="MI:0488" id="MI:0117" refType="identity" refTypeAc="MI:0356"**/>

<**secondaryRef db="unknown" id="EBI-456493" refType="identity" refTypeAc="MI:0356"**/>

<**secondaryRef db="pubmed" dbAc="MI:0446" id="14755292" refType="primary-reference" refTypeAc="MI:0358"**/>

</**xref**>

</**featureType**>

<**featureRangeList**>

<**featureRange**>

<**startStatus**>

<**names**>

<**shortLabel**>undetermined</**shortLabel**>

<**fullName**>undetermined sequence position</**fullName**>

</**names**>

<**xref**>

<**primaryRef db="psi-mi" dbAc="MI:0488" id="MI:0339" refType="identity" refTypeAc="MI:0356"**/>

<**secondaryRef db="unknown" id="EBI-448295" refType="identity" refTypeAc="MI:0356"**/>

<**secondaryRef db="pubmed" dbAc="MI:0446" id="14755292" refType="primary-reference" refTypeAc="MI:0358"**/>

</**xref**>

</**startStatus**>

<**endStatus**>

<**names**>

<**shortLabel**>undetermined</**shortLabel**>

<**fullName**>undetermined sequence position</**fullName**>

</**names**>

<**xref**>

<**primaryRef db="psi-mi" dbAc="MI:0488" id="MI:0339" refType="identity" refTypeAc="MI:0356"**/>

<**secondaryRef db="unknown" id="EBI-448295" refType="identity" refTypeAc="MI:0356"**/>

<**secondaryRef db="pubmed" dbAc="MI:0446" id="14755292" refType="primary-reference" refTypeAc="MI:0358"**/>

</**xref**>

</**endStatus**>

</**featureRange**>

</**featureRangeList**>

</**feature**>

</**featureList**>

<**stoichiometry value="1"**/>

</**participant**>

<**participant id="15"**>

<**interactorRef**>2</**interactorRef**>

<**biologicalRole**>

<**names**>

<**shortLabel**>unspecified role</**shortLabel**>

<**fullName**>unspecified role</**fullName**>

</**names**>

<**xref**>

<**primaryRef db="psi-mi" dbAc="MI:0488" id="MI:0499" refType="identity" refTypeAc="MI:0356"**/>

<**secondaryRef db="unknown" id="EBI-77781" refType="identity" refTypeAc="MI:0356"**/>

<**secondaryRef db="pubmed" dbAc="MI:0446" id="14755292" refType="primary-reference" refTypeAc="MI:0358"**/>

</**xref**>

</**biologicalRole**>

<**featureList**>

<**feature id="16"**>

<**names**>

<**shortLabel**>terf2 binding region</**shortLabel**>

</**names**>

<**xref**>

<**primaryRef db="unknown" id="EBI-10888362" refType="identity" refTypeAc="MI:0356"**/>

</**xref**>

<**featureType**>

<**names**>

<**shortLabel**>binding region</**shortLabel**>

<**fullName**>binding-associated region</**fullName**>

</**names**>

<**xref**>

<**primaryRef db="psi-mi" dbAc="MI:0488" id="MI:0117" refType="identity" refTypeAc="MI:0356"**/>

<**secondaryRef db="unknown" id="EBI-456493" refType="identity" refTypeAc="MI:0356"**/>

<**secondaryRef db="pubmed" dbAc="MI:0446" id="14755292" refType="primary-reference" refTypeAc="MI:0358"**/>

</**xref**>

</**featureType**>

<**featureRangeList**>

<**featureRange**>

<**startStatus**>

<**names**>

<**shortLabel**>certain</**shortLabel**>

<**fullName**>certain sequence position</**fullName**>

<**alias type="synonym" typeAc="MI:1041"**>certain</**alias**>

</**names**>

<**xref**>

<**primaryRef db="psi-mi" dbAc="MI:0488" id="MI:0335" refType="identity" refTypeAc="MI:0356"**/>

<**secondaryRef db="unknown" id="EBI-540564" refType="identity" refTypeAc="MI:0356"**/>

<**secondaryRef db="pubmed" dbAc="MI:0446" id="14755292" refType="primary-reference" refTypeAc="MI:0358"**/>

</**xref**>

</**startStatus**>

<**begin position="307"**/>

<**endStatus**>

<**names**>

<**shortLabel**>certain</**shortLabel**>

<**fullName**>certain sequence position</**fullName**>

<**alias type="synonym" typeAc="MI:1041"**>certain</**alias**>

</**names**>

<**xref**>

<**primaryRef db="psi-mi" dbAc="MI:0488" id="MI:0335" refType="identity" refTypeAc="MI:0356"**/>

<**secondaryRef db="unknown" id="EBI-540564" refType="identity" refTypeAc="MI:0356"**/>

<**secondaryRef db="pubmed" dbAc="MI:0446" id="14755292" refType="primary-reference" refTypeAc="MI:0358"**/>

</**xref**>

</**endStatus**>

<**end position="398"**/>

</**featureRange**>

</**featureRangeList**>

</**feature**>

</**featureList**>

<**stoichiometry value="1"**/>

</**participant**>

<**participant id="17"**>

<**interactorRef**>3</**interactorRef**>

<**biologicalRole**>

<**names**>

<**shortLabel**>unspecified role</**shortLabel**>

<**fullName**>unspecified role</**fullName**>

</**names**>

<**xref**>

<**primaryRef db="psi-mi" dbAc="MI:0488" id="MI:0499" refType="identity" refTypeAc="MI:0356"**/>

<**secondaryRef db="unknown" id="EBI-77781" refType="identity" refTypeAc="MI:0356"**/>

<**secondaryRef db="pubmed" dbAc="MI:0446" id="14755292" refType="primary-reference" refTypeAc="MI:0358"**/>

</**xref**>

</**biologicalRole**>

<**featureList**>

<**feature id="18"**>

<**names**>

<**shortLabel**>terf1 binding region</**shortLabel**>

</**names**>

<**xref**>

<**primaryRef db="unknown" id="EBI-10897115" refType="identity" refTypeAc="MI:0356"**/>

</**xref**>

<**featureType**>

<**names**>

<**shortLabel**>binding region</**shortLabel**>

<**fullName**>binding-associated region</**fullName**>

</**names**>

<**xref**>

<**primaryRef db="psi-mi" dbAc="MI:0488" id="MI:0117" refType="identity" refTypeAc="MI:0356"**/>

<**secondaryRef db="unknown" id="EBI-456493" refType="identity" refTypeAc="MI:0356"**/>

<**secondaryRef db="pubmed" dbAc="MI:0446" id="14755292" refType="primary-reference" refTypeAc="MI:0358"**/>

</**xref**>

</**featureType**>

<**featureRangeList**>

<**featureRange**>

<**startStatus**>

<**names**>

<**shortLabel**>certain</**shortLabel**>

<**fullName**>certain sequence position</**fullName**>

<**alias type="synonym" typeAc="MI:1041"**>certain</**alias**>

</**names**>

<**xref**>

<**primaryRef db="psi-mi" dbAc="MI:0488" id="MI:0335" refType="identity" refTypeAc="MI:0356"**/>

<**secondaryRef db="unknown" id="EBI-540564" refType="identity" refTypeAc="MI:0356"**/>

<**secondaryRef db="pubmed" dbAc="MI:0446" id="14755292" refType="primary-reference" refTypeAc="MI:0358"**/>

</**xref**>

</**startStatus**>

<**begin position="62"**/>

<**endStatus**>

<**names**>

<**shortLabel**>certain</**shortLabel**>

<**fullName**>certain sequence position</**fullName**>

<**alias type="synonym" typeAc="MI:1041"**>certain</**alias**>

</**names**>

<**xref**>

<**primaryRef db="psi-mi" dbAc="MI:0488" id="MI:0335" refType="identity" refTypeAc="MI:0356"**/>

<**secondaryRef db="unknown" id="EBI-540564" refType="identity" refTypeAc="MI:0356"**/>

<**secondaryRef db="pubmed" dbAc="MI:0446" id="14755292" refType="primary-reference" refTypeAc="MI:0358"**/>

</**xref**>

</**endStatus**>

<**end position="265"**/>

</**featureRange**>

</**featureRangeList**>

</**feature**>

<**feature id="19"**>

<**names**>

<**shortLabel**>tinf2 binding region</**shortLabel**>

</**names**>

<**xref**>

<**primaryRef db="unknown" id="EBI-10888354" refType="identity" refTypeAc="MI:0356"**/>

</**xref**>

<**featureType**>

<**names**>

<**shortLabel**>binding region</**shortLabel**>

<**fullName**>binding-associated region</**fullName**>

</**names**>

<**xref**>

<**primaryRef db="psi-mi" dbAc="MI:0488" id="MI:0117" refType="identity" refTypeAc="MI:0356"**/>

<**secondaryRef db="unknown" id="EBI-456493" refType="identity" refTypeAc="MI:0356"**/>

<**secondaryRef db="pubmed" dbAc="MI:0446" id="14755292" refType="primary-reference" refTypeAc="MI:0358"**/>

</**xref**>

</**featureType**>

<**featureRangeList**>

<**featureRange**>

<**startStatus**>

<**names**>

<**shortLabel**>certain</**shortLabel**>

<**fullName**>certain sequence position</**fullName**>

<**alias type="synonym" typeAc="MI:1041"**>certain</**alias**>

</**names**>

<**xref**>

<**primaryRef db="psi-mi" dbAc="MI:0488" id="MI:0335" refType="identity" refTypeAc="MI:0356"**/>

<**secondaryRef db="unknown" id="EBI-540564" refType="identity" refTypeAc="MI:0356"**/>

<**secondaryRef db="pubmed" dbAc="MI:0446" id="14755292" refType="primary-reference" refTypeAc="MI:0358"**/>

</**xref**>

</**startStatus**>

<**begin position="62"**/>

<**endStatus**>

<**names**>

<**shortLabel**>certain</**shortLabel**>

<**fullName**>certain sequence position</**fullName**>

<**alias type="synonym" typeAc="MI:1041"**>certain</**alias**>

</**names**>

<**xref**>

<**primaryRef db="psi-mi" dbAc="MI:0488" id="MI:0335" refType="identity" refTypeAc="MI:0356"**/>

<**secondaryRef db="unknown" id="EBI-540564" refType="identity" refTypeAc="MI:0356"**/>

<**secondaryRef db="pubmed" dbAc="MI:0446" id="14755292" refType="primary-reference" refTypeAc="MI:0358"**/>

</**xref**>

</**endStatus**>

<**end position="201"**/>

</**featureRange**>

<**featureRange**>

<**startStatus**>

<**names**>

<**shortLabel**>certain</**shortLabel**>

<**fullName**>certain sequence position</**fullName**>

<**alias type="synonym" typeAc="MI:1041"**>certain</**alias**>

</**names**>

<**xref**>

<**primaryRef db="psi-mi" dbAc="MI:0488" id="MI:0335" refType="identity" refTypeAc="MI:0356"**/>

<**secondaryRef db="unknown" id="EBI-540564" refType="identity" refTypeAc="MI:0356"**/>

<**secondaryRef db="pubmed" dbAc="MI:0446" id="14755292" refType="primary-reference" refTypeAc="MI:0358"**/>

</**xref**>

</**startStatus**>

<**begin position="207"**/>

<**endStatus**>

<**names**>

<**shortLabel**>certain</**shortLabel**>

<**fullName**>certain sequence position</**fullName**>

<**alias type="synonym" typeAc="MI:1041"**>certain</**alias**>

</**names**>

<**xref**>

<**primaryRef db="psi-mi" dbAc="MI:0488" id="MI:0335" refType="identity" refTypeAc="MI:0356"**/>

<**secondaryRef db="unknown" id="EBI-540564" refType="identity" refTypeAc="MI:0356"**/>

<**secondaryRef db="pubmed" dbAc="MI:0446" id="14755292" refType="primary-reference" refTypeAc="MI:0358"**/>

</**xref**>

</**endStatus**>

<**end position="268"**/>

</**featureRange**>

</**featureRangeList**>

</**feature**>

</**featureList**>

<**stoichiometry value="1"**/>

</**participant**>

<**participant id="20"**>

<**interactorRef**>4</**interactorRef**>

<**biologicalRole**>

<**names**>

<**shortLabel**>unspecified role</**shortLabel**>

<**fullName**>unspecified role</**fullName**>

</**names**>

<**xref**>

<**primaryRef db="psi-mi" dbAc="MI:0488" id="MI:0499" refType="identity" refTypeAc="MI:0356"**/>

<**secondaryRef db="unknown" id="EBI-77781" refType="identity" refTypeAc="MI:0356"**/>

<**secondaryRef db="pubmed" dbAc="MI:0446" id="14755292" refType="primary-reference" refTypeAc="MI:0358"**/>

</**xref**>

</**biologicalRole**>

<**featureList**>

<**feature id="21"**>

<**names**>

<**shortLabel**>pot1 binding region</**shortLabel**>

</**names**>

<**xref**>

<**primaryRef db="unknown" id="EBI-10897571" refType="identity" refTypeAc="MI:0356"**/>

</**xref**>

<**featureType**>

<**names**>

<**shortLabel**>binding region</**shortLabel**>

<**fullName**>binding-associated region</**fullName**>

</**names**>

<**xref**>

<**primaryRef db="psi-mi" dbAc="MI:0488" id="MI:0117" refType="identity" refTypeAc="MI:0356"**/>

<**secondaryRef db="unknown" id="EBI-456493" refType="identity" refTypeAc="MI:0356"**/>

<**secondaryRef db="pubmed" dbAc="MI:0446" id="14755292" refType="primary-reference" refTypeAc="MI:0358"**/>

</**xref**>

</**featureType**>

<**featureRangeList**>

<**featureRange**>

<**startStatus**>

<**names**>

<**shortLabel**>undetermined</**shortLabel**>

<**fullName**>undetermined sequence position</**fullName**>

</**names**>

<**xref**>

<**primaryRef db="psi-mi" dbAc="MI:0488" id="MI:0339" refType="identity" refTypeAc="MI:0356"**/>

<**secondaryRef db="unknown" id="EBI-448295" refType="identity" refTypeAc="MI:0356"**/>

<**secondaryRef db="pubmed" dbAc="MI:0446" id="14755292" refType="primary-reference" refTypeAc="MI:0358"**/>

</**xref**>

</**startStatus**>

<**endStatus**>

<**names**>

<**shortLabel**>undetermined</**shortLabel**>

<**fullName**>undetermined sequence position</**fullName**>

</**names**>

<**xref**>

<**primaryRef db="psi-mi" dbAc="MI:0488" id="MI:0339" refType="identity" refTypeAc="MI:0356"**/>

<**secondaryRef db="unknown" id="EBI-448295" refType="identity" refTypeAc="MI:0356"**/>

<**secondaryRef db="pubmed" dbAc="MI:0446" id="14755292" refType="primary-reference" refTypeAc="MI:0358"**/>

</**xref**>

</**endStatus**>

</**featureRange**>

</**featureRangeList**>

</**feature**>

<**feature id="22"**>

<**names**>

<**shortLabel**>tinf2 binding region</**shortLabel**>

</**names**>

<**xref**>

<**primaryRef db="unknown" id="EBI-10897567" refType="identity" refTypeAc="MI:0356"**/>

</**xref**>

<**featureType**>

<**names**>

<**shortLabel**>binding region</**shortLabel**>

<**fullName**>binding-associated region</**fullName**>

</**names**>

<**xref**>

<**primaryRef db="psi-mi" dbAc="MI:0488" id="MI:0117" refType="identity" refTypeAc="MI:0356"**/>

<**secondaryRef db="unknown" id="EBI-456493" refType="identity" refTypeAc="MI:0356"**/>

<**secondaryRef db="pubmed" dbAc="MI:0446" id="14755292" refType="primary-reference" refTypeAc="MI:0358"**/>

</**xref**>

</**featureType**>

<**featureRangeList**>

<**featureRange**>

<**startStatus**>

<**names**>

<**shortLabel**>undetermined</**shortLabel**>

<**fullName**>undetermined sequence position</**fullName**>

</**names**>

<**xref**>

<**primaryRef db="psi-mi" dbAc="MI:0488" id="MI:0339" refType="identity" refTypeAc="MI:0356"**/>

<**secondaryRef db="unknown" id="EBI-448295" refType="identity" refTypeAc="MI:0356"**/>

<**secondaryRef db="pubmed" dbAc="MI:0446" id="14755292" refType="primary-reference" refTypeAc="MI:0358"**/>

</**xref**>

</**startStatus**>

<**endStatus**>

<**names**>

<**shortLabel**>undetermined</**shortLabel**>

<**fullName**>undetermined sequence position</**fullName**>

</**names**>

<**xref**>

<**primaryRef db="psi-mi" dbAc="MI:0488" id="MI:0339" refType="identity" refTypeAc="MI:0356"**/>

<**secondaryRef db="unknown" id="EBI-448295" refType="identity" refTypeAc="MI:0356"**/>

<**secondaryRef db="pubmed" dbAc="MI:0446" id="14755292" refType="primary-reference" refTypeAc="MI:0358"**/>

</**xref**>

</**endStatus**>

</**featureRange**>

</**featureRangeList**>

</**feature**>

</**featureList**>

<**stoichiometry value="1"**/>

</**participant**>

<**participant id="23"**>

<**interactorRef**>7</**interactorRef**>

<**biologicalRole**>

<**names**>

<**shortLabel**>unspecified role</**shortLabel**>

<**fullName**>unspecified role</**fullName**>

</**names**>

<**xref**>

<**primaryRef db="psi-mi" dbAc="MI:0488" id="MI:0499" refType="identity" refTypeAc="MI:0356"**/>

<**secondaryRef db="unknown" id="EBI-77781" refType="identity" refTypeAc="MI:0356"**/>

<**secondaryRef db="pubmed" dbAc="MI:0446" id="14755292" refType="primary-reference" refTypeAc="MI:0358"**/>

</**xref**>

</**biologicalRole**>

<**featureList**>

<**feature id="24"**>

<**names**>

<**shortLabel**>terf1 binding region</**shortLabel**>

</**names**>

<**xref**>

<**primaryRef db="unknown" id="EBI-10888352" refType="identity" refTypeAc="MI:0356"**/>

</**xref**>

<**featureType**>

<**names**>

<**shortLabel**>binding region</**shortLabel**>

<**fullName**>binding-associated region</**fullName**>

</**names**>

<**xref**>

<**primaryRef db="psi-mi" dbAc="MI:0488" id="MI:0117" refType="identity" refTypeAc="MI:0356"**/>

<**secondaryRef db="unknown" id="EBI-456493" refType="identity" refTypeAc="MI:0356"**/>

<**secondaryRef db="pubmed" dbAc="MI:0446" id="14755292" refType="primary-reference" refTypeAc="MI:0358"**/>

</**xref**>

</**featureType**>

<**featureRangeList**>

<**featureRange**>

<**startStatus**>

<**names**>

<**shortLabel**>certain</**shortLabel**>

<**fullName**>certain sequence position</**fullName**>

<**alias type="synonym" typeAc="MI:1041"**>certain</**alias**>

</**names**>

<**xref**>

<**primaryRef db="psi-mi" dbAc="MI:0488" id="MI:0335" refType="identity" refTypeAc="MI:0356"**/>

<**secondaryRef db="unknown" id="EBI-540564" refType="identity" refTypeAc="MI:0356"**/>

<**secondaryRef db="pubmed" dbAc="MI:0446" id="14755292" refType="primary-reference" refTypeAc="MI:0358"**/>

</**xref**>

</**startStatus**>

<**begin position="257"**/>

<**endStatus**>

<**names**>

<**shortLabel**>certain</**shortLabel**>

<**fullName**>certain sequence position</**fullName**>

<**alias type="synonym" typeAc="MI:1041"**>certain</**alias**>

</**names**>

<**xref**>

<**primaryRef db="psi-mi" dbAc="MI:0488" id="MI:0335" refType="identity" refTypeAc="MI:0356"**/>

<**secondaryRef db="unknown" id="EBI-540564" refType="identity" refTypeAc="MI:0356"**/>

<**secondaryRef db="pubmed" dbAc="MI:0446" id="14755292" refType="primary-reference" refTypeAc="MI:0358"**/>

</**xref**>

</**endStatus**>

<**end position="268"**/>

</**featureRange**>

</**featureRangeList**>

</**feature**>

<**feature id="25"**>

<**names**>

<**shortLabel**>terf2 binding region</**shortLabel**>

</**names**>

<**xref**>

<**primaryRef db="unknown" id="EBI-10888360" refType="identity" refTypeAc="MI:0356"**/>

</**xref**>

<**featureType**>

<**names**>

<**shortLabel**>binding region</**shortLabel**>

<**fullName**>binding-associated region</**fullName**>

</**names**>

<**xref**>

<**primaryRef db="psi-mi" dbAc="MI:0488" id="MI:0117" refType="identity" refTypeAc="MI:0356"**/>

<**secondaryRef db="unknown" id="EBI-456493" refType="identity" refTypeAc="MI:0356"**/>

<**secondaryRef db="pubmed" dbAc="MI:0446" id="14755292" refType="primary-reference" refTypeAc="MI:0358"**/>

</**xref**>

</**featureType**>

<**featureRangeList**>

<**featureRange**>

<**startStatus**>

<**names**>

<**shortLabel**>certain</**shortLabel**>

<**fullName**>certain sequence position</**fullName**>

<**alias type="synonym" typeAc="MI:1041"**>certain</**alias**>

</**names**>

<**xref**>

<**primaryRef db="psi-mi" dbAc="MI:0488" id="MI:0335" refType="identity" refTypeAc="MI:0356"**/>

<**secondaryRef db="unknown" id="EBI-540564" refType="identity" refTypeAc="MI:0356"**/>

<**secondaryRef db="pubmed" dbAc="MI:0446" id="14755292" refType="primary-reference" refTypeAc="MI:0358"**/>

</**xref**>

</**startStatus**>

<**begin position="258"**/>

<**endStatus**>

<**names**>

<**shortLabel**>certain</**shortLabel**>

<**fullName**>certain sequence position</**fullName**>

<**alias type="synonym" typeAc="MI:1041"**>certain</**alias**>

</**names**>

<**xref**>

<**primaryRef db="psi-mi" dbAc="MI:0488" id="MI:0335" refType="identity" refTypeAc="MI:0356"**/>

<**secondaryRef db="unknown" id="EBI-540564" refType="identity" refTypeAc="MI:0356"**/>

<**secondaryRef db="pubmed" dbAc="MI:0446" id="14755292" refType="primary-reference" refTypeAc="MI:0358"**/>

</**xref**>

</**endStatus**>

<**end position="266"**/>

</**featureRange**>

</**featureRangeList**>

</**feature**>

<**feature id="26"**>

<**names**>

<**shortLabel**>acd binding region</**shortLabel**>

</**names**>

<**xref**>

<**primaryRef db="unknown" id="EBI-10897565" refType="identity" refTypeAc="MI:0356"**/>

</**xref**>

<**featureType**>

<**names**>

<**shortLabel**>binding region</**shortLabel**>

<**fullName**>binding-associated region</**fullName**>

</**names**>

<**xref**>

<**primaryRef db="psi-mi" dbAc="MI:0488" id="MI:0117" refType="identity" refTypeAc="MI:0356"**/>

<**secondaryRef db="unknown" id="EBI-456493" refType="identity" refTypeAc="MI:0356"**/>

<**secondaryRef db="pubmed" dbAc="MI:0446" id="14755292" refType="primary-reference" refTypeAc="MI:0358"**/>

</**xref**>

</**featureType**>

<**featureRangeList**>

<**featureRange**>

<**startStatus**>

<**names**>

<**shortLabel**>undetermined</**shortLabel**>

<**fullName**>undetermined sequence position</**fullName**>

</**names**>

<**xref**>

<**primaryRef db="psi-mi" dbAc="MI:0488" id="MI:0339" refType="identity" refTypeAc="MI:0356"**/>

<**secondaryRef db="unknown" id="EBI-448295" refType="identity" refTypeAc="MI:0356"**/>

<**secondaryRef db="pubmed" dbAc="MI:0446" id="14755292" refType="primary-reference" refTypeAc="MI:0358"**/>

</**xref**>

</**startStatus**>

<**endStatus**>

<**names**>

<**shortLabel**>undetermined</**shortLabel**>

<**fullName**>undetermined sequence position</**fullName**>

</**names**>

<**xref**>

<**primaryRef db="psi-mi" dbAc="MI:0488" id="MI:0339" refType="identity" refTypeAc="MI:0356"**/>

<**secondaryRef db="unknown" id="EBI-448295" refType="identity" refTypeAc="MI:0356"**/>

<**secondaryRef db="pubmed" dbAc="MI:0446" id="14755292" refType="primary-reference" refTypeAc="MI:0358"**/>

</**xref**>

</**endStatus**>

</**featureRange**>

</**featureRangeList**>

</**feature**>

</**featureList**>

<**stoichiometry value="1"**/>

</**participant**>

<**participant id="27"**>

<**interactorRef**>6</**interactorRef**>

<**biologicalRole**>

<**names**>

<**shortLabel**>unspecified role</**shortLabel**>

<**fullName**>unspecified role</**fullName**>

</**names**>

<**xref**>

<**primaryRef db="psi-mi" dbAc="MI:0488" id="MI:0499" refType="identity" refTypeAc="MI:0356"**/>

<**secondaryRef db="unknown" id="EBI-77781" refType="identity" refTypeAc="MI:0356"**/>

<**secondaryRef db="pubmed" dbAc="MI:0446" id="14755292" refType="primary-reference" refTypeAc="MI:0358"**/>

</**xref**>

</**biologicalRole**>

<**featureList**>

<**feature id="28"**>

<**names**>

<**shortLabel**>terf2 binding region</**shortLabel**>

</**names**>

<**xref**>

<**primaryRef db="unknown" id="EBI-10897640" refType="identity" refTypeAc="MI:0356"**/>

</**xref**>

<**featureType**>

<**names**>

<**shortLabel**>binding region</**shortLabel**>

<**fullName**>binding-associated region</**fullName**>

</**names**>

<**xref**>

<**primaryRef db="psi-mi" dbAc="MI:0488" id="MI:0117" refType="identity" refTypeAc="MI:0356"**/>

<**secondaryRef db="unknown" id="EBI-456493" refType="identity" refTypeAc="MI:0356"**/>

<**secondaryRef db="pubmed" dbAc="MI:0446" id="14755292" refType="primary-reference" refTypeAc="MI:0358"**/>

</**xref**>

</**featureType**>

<**featureRangeList**>

<**featureRange**>

<**startStatus**>

<**names**>

<**shortLabel**>certain</**shortLabel**>

<**fullName**>certain sequence position</**fullName**>

<**alias type="synonym" typeAc="MI:1041"**>certain</**alias**>

</**names**>

<**xref**>

<**primaryRef db="psi-mi" dbAc="MI:0488" id="MI:0335" refType="identity" refTypeAc="MI:0356"**/>

<**secondaryRef db="unknown" id="EBI-540564" refType="identity" refTypeAc="MI:0356"**/>

<**secondaryRef db="pubmed" dbAc="MI:0446" id="14755292" refType="primary-reference" refTypeAc="MI:0358"**/>

</**xref**>

</**startStatus**>

<**begin position="85"**/>

<**endStatus**>

<**names**>

<**shortLabel**>certain</**shortLabel**>

<**fullName**>certain sequence position</**fullName**>

<**alias type="synonym" typeAc="MI:1041"**>certain</**alias**>

</**names**>

<**xref**>

<**primaryRef db="psi-mi" dbAc="MI:0488" id="MI:0335" refType="identity" refTypeAc="MI:0356"**/>

<**secondaryRef db="unknown" id="EBI-540564" refType="identity" refTypeAc="MI:0356"**/>

<**secondaryRef db="pubmed" dbAc="MI:0446" id="14755292" refType="primary-reference" refTypeAc="MI:0358"**/>

</**xref**>

</**endStatus**>

<**end position="287"**/>

</**featureRange**>

</**featureRangeList**>

</**feature**>

</**featureList**>

<**stoichiometry value="1"**/>

</**participant**>

<**participant id="29"**>

<**interactorRef**>3</**interactorRef**>

<**biologicalRole**>

<**names**>

<**shortLabel**>unspecified role</**shortLabel**>

<**fullName**>unspecified role</**fullName**>

</**names**>

<**xref**>

<**primaryRef db="psi-mi" dbAc="MI:0488" id="MI:0499" refType="identity" refTypeAc="MI:0356"**/>

<**secondaryRef db="unknown" id="EBI-77781" refType="identity" refTypeAc="MI:0356"**/>

<**secondaryRef db="pubmed" dbAc="MI:0446" id="14755292" refType="primary-reference" refTypeAc="MI:0358"**/>

</**xref**>

</**biologicalRole**>

<**featureList**>

<**feature id="30"**>

<**names**>

<**shortLabel**>terf1 binding region</**shortLabel**>

</**names**>

<**xref**>

<**primaryRef db="unknown" id="EBI-10897117" refType="identity" refTypeAc="MI:0356"**/>

</**xref**>

<**featureType**>

<**names**>

<**shortLabel**>binding region</**shortLabel**>

<**fullName**>binding-associated region</**fullName**>

</**names**>

<**xref**>

<**primaryRef db="psi-mi" dbAc="MI:0488" id="MI:0117" refType="identity" refTypeAc="MI:0356"**/>

<**secondaryRef db="unknown" id="EBI-456493" refType="identity" refTypeAc="MI:0356"**/>

<**secondaryRef db="pubmed" dbAc="MI:0446" id="14755292" refType="primary-reference" refTypeAc="MI:0358"**/>

</**xref**>

</**featureType**>

<**featureRangeList**>

<**featureRange**>

<**startStatus**>

<**names**>

<**shortLabel**>certain</**shortLabel**>

<**fullName**>certain sequence position</**fullName**>

<**alias type="synonym" typeAc="MI:1041"**>certain</**alias**>

</**names**>

<**xref**>

<**primaryRef db="psi-mi" dbAc="MI:0488" id="MI:0335" refType="identity" refTypeAc="MI:0356"**/>

<**secondaryRef db="unknown" id="EBI-540564" refType="identity" refTypeAc="MI:0356"**/>

<**secondaryRef db="pubmed" dbAc="MI:0446" id="14755292" refType="primary-reference" refTypeAc="MI:0358"**/>

</**xref**>

</**startStatus**>

<**begin position="62"**/>

<**endStatus**>

<**names**>

<**shortLabel**>certain</**shortLabel**>

<**fullName**>certain sequence position</**fullName**>

<**alias type="synonym" typeAc="MI:1041"**>certain</**alias**>

</**names**>

<**xref**>

<**primaryRef db="psi-mi" dbAc="MI:0488" id="MI:0335" refType="identity" refTypeAc="MI:0356"**/>

<**secondaryRef db="unknown" id="EBI-540564" refType="identity" refTypeAc="MI:0356"**/>

<**secondaryRef db="pubmed" dbAc="MI:0446" id="14755292" refType="primary-reference" refTypeAc="MI:0358"**/>

</**xref**>

</**endStatus**>

<**end position="265"**/>

</**featureRange**>

</**featureRangeList**>

</**feature**>

</**featureList**>

<**stoichiometry value="1"**/>

</**participant**>

</**participantList**>

<**bindingFeatureList**>

<**bindingFeatures**>

<**participantFeatureRef**>10</**participantFeatureRef**>

<**participantFeatureRef**>16</**participantFeatureRef**>

</**bindingFeatures**>

<**bindingFeatures**>

<**participantFeatureRef**>25</**participantFeatureRef**>

<**participantFeatureRef**>11</**participantFeatureRef**>

</**bindingFeatures**>

<**bindingFeatures**>

<**participantFeatureRef**>28</**participantFeatureRef**>

<**participantFeatureRef**>12</**participantFeatureRef**>

</**bindingFeatures**>

<**bindingFeatures**>

<**participantFeatureRef**>21</**participantFeatureRef**>

<**participantFeatureRef**>14</**participantFeatureRef**>

</**bindingFeatures**>

<**bindingFeatures**>

<**participantFeatureRef**>30</**participantFeatureRef**>

<**participantFeatureRef**>18</**participantFeatureRef**>

</**bindingFeatures**>

<**bindingFeatures**>

<**participantFeatureRef**>19</**participantFeatureRef**>

<**participantFeatureRef**>24</**participantFeatureRef**>

</**bindingFeatures**>

<**bindingFeatures**>

<**participantFeatureRef**>26</**participantFeatureRef**>

<**participantFeatureRef**>22</**participantFeatureRef**>

</**bindingFeatures**>

</**bindingFeatureList**>

<**interactionType**>

<**names**>

<**shortLabel**>physical association</**shortLabel**>

<**fullName**>physical association</**fullName**>

</**names**>

<**xref**>

<**primaryRef db="psi-mi" dbAc="MI:0488" id="MI:0915" refType="identity" refTypeAc="MI:0356"**/>

<**secondaryRef db="unknown" id="EBI-1813147" refType="identity" refTypeAc="MI:0356"**/>

<**secondaryRef db="pubmed" dbAc="MI:0446" id="14755292" refType="primary-reference" refTypeAc="MI:0358"**/>

</**xref**>

</**interactionType**>

<**organism ncbiTaxId="9606"**>

<**names**>

<**shortLabel**>human</**shortLabel**>

<**fullName**>Homo sapiens</**fullName**>

<**alias type="synonym" typeAc="MI:1041"**>Human</**alias**>

</**names**>

</**organism**>

<**interactorType**>

<**names**>

<**shortLabel**>stable complex</**shortLabel**>

<**fullName**>stable complex</**fullName**>

</**names**>

<**xref**>

<**primaryRef db="psi-mi" dbAc="MI:0488" id="MI:1302" refType="identity" refTypeAc="MI:0356"**/>

<**secondaryRef db="unknown" id="EBI-6862248" refType="identity" refTypeAc="MI:0356"**/>

<**secondaryRef db="pubmed" dbAc="MI:0446" id="12853464" refType="primary-reference" refTypeAc="MI:0358"**/>

</**xref**>

</**interactorType**>

<**evidenceType**>

<**names**>

<**shortLabel**>physical interaction evidence used in manual assertion</**shortLabel**>

<**fullName**>physical interaction evidence used in manual assertion</**fullName**>

</**names**>

<**xref**>

<**primaryRef db="evidence ontology" dbAc="MI:1331" id="ECO:0000353" refType="identity" refTypeAc="MI:0356"**/>

<**secondaryRef db="unknown" id="EBI-9828818" refType="identity" refTypeAc="MI:0356"**/>

</**xref**>

</**evidenceType**>

<**attributeList**>

<**attribute name="curated-complex"**>The Shelterin (Telosome) complex is a DNA-binding protein complex that associates with the telomeres that cap the ends of eukaryotic chromosomes and distinguishes them from sites of DNA damage thus sheltering chromosome ends from being inappropriately processed by the DNA repair machinery. Consequently it plays an essential role in maintaining telomere structure and integrity. Three subunits can interact directly either with single-stranded (POT1) or double-stranded telomeric DNA (TERF1 and TERF2).

</**attribute**>

<**attribute name="curation request" nameAc="MI:0873"**>by Nancy Campbell at BHF-UCL</**attribute**>

<**attribute name="complex-assembly"**>Hetero-octamer</**attribute**>

<**attribute name="complex-properties" nameAc="MI:0629"**>MW ~ 1 MDa</**attribute**>

<**attribute name="accepted"**>Accepted 2015-JUL-20 by BMELDAL</**attribute**>

</**attributeList**>

</**abstractInteraction**>

</**interactionList**>

</**entry**>

</**entrySet**>
