# Supplementary material for: Encompassing new use cases - level 3.0 of the HUPO-PSI format for molecular interactions
Source: BMC Bioinformatics. 2018 Apr 11;19:134. doi: 10.1186/s12859-018-2118-1 (PMC5896046; doi:10.1186/s12859-018-2118-1)
Supplement: Supplementary file 8 — Representation of a cooperative interaction in PSI-MI XML3.0.0 (Use case 1.3 h). (https://github.com/HUPO-PSI/miXML/blob/master/3.0/pub/Appendix%209.docx). (DOCX 28 kb) [file 12859_2018_2118_MOESM8_ESM.docx]

**Representation of a cooperative interaction in PSI-MI XML3.0**

The ability to describe cooperative or allosteric binding where a series of events are required to describe an interaction.

(Note - this example has been specifically created for this publication – there is currently no database representation).

*<?***xml version="1.0" encoding="UTF-8"***?>*

<**entrySet xmlns:xsi="http://www.w3.org/2001/XMLSchema-instance"**

**xmlns="http://psi.hupo.org/mi/mif300"**

**xsi:schemaLocation="http://psi.hupo.org/mi/mif300 https://raw.githubusercontent.com/HUPO-PSI/miXML/master/3.0/src/MIF300.xsd"**

**level="3" version="0" minorVersion="0"**>

<**entry**>

<**interactorList**>

<**interactor id="1"**>

<**names**>

<**shortLabel**>Pygo1</**shortLabel**>

<**fullName**>Pygopus homolog 1</**fullName**>

<**alias type="gene name" typeAc="MI:0301"**>PYGO1</**alias**>

<**alias type="display_short"**>Q9Y3Y4</**alias**>

<**alias type="display_long"**>pygo1_human</**alias**>

</**names**>

<**xref**>

<**primaryRef id="Q9Y3Y4" refTypeAc="MI:0356" refType="identity" dbAc="MI:0486" db="uniprotkb"**/>

<**secondaryRef id="ENSG00000171016" refTypeAc="MI:0251" refType="gene product" dbAc="MI:0476" db="ensembl"**/>

<**secondaryRef id="NP_056432.1" refTypeAc="MI:0356" refType="identity" dbAc="MI:0481" db="refseq"**/>

</**xref**>

<**interactorType**>

<**names**>

<**shortLabel**>protein</**shortLabel**>

<**fullName**>protein</**fullName**>

</**names**>

<**xref**>

<**primaryRef id="MI:0326" refTypeAc="MI:0356" refType="identity" dbAc="MI:0488" db="psi-mi"**/>

</**xref**>

</**interactorType**>

<**organism ncbiTaxId="9606"**>

<**names**>

<**shortLabel**>Human</**shortLabel**>

</**names**>

</**organism**>

</**interactor**>

<**interactor id="2"**>

<**names**>

<**shortLabel**>Bcl9</**shortLabel**>

<**fullName**>B-cell CLL/lymphoma 9 protein</**fullName**>

<**alias type="gene name" typeAc="MI:0301"**>BCL9</**alias**>

<**alias type="display_short"**>O00512</**alias**>

<**alias type="display_long"**>bcl9_human</**alias**>

</**names**>

<**xref**>

<**primaryRef id="O00512" refTypeAc="MI:0356" refType="identity" dbAc="MI:0486" db="uniprotkb"**/>

<**secondaryRef id="ENSG00000116128" refTypeAc="MI:0251" refType="gene product" dbAc="MI:0476" db="ensembl"**/>

<**secondaryRef id="NP_004317.2" refTypeAc="MI:0356" refType="identity" dbAc="MI:0481" db="refseq"**/>

</**xref**>

<**interactorType**>

<**names**>

<**shortLabel**>protein</**shortLabel**>

<**fullName**>protein</**fullName**>

</**names**>

<**xref**>

<**primaryRef id="MI:0326" refTypeAc="MI:0356" refType="identity" dbAc="MI:0488" db="psi-mi"**/>

</**xref**>

</**interactorType**>

<**organism ncbiTaxId="9606"**>

<**names**>

<**shortLabel**>Human</**shortLabel**>

</**names**>

</**organism**>

</**interactor**>

<**interactor id="3"**>

<**names**>

<**shortLabel**>H3K4me2</**shortLabel**>

<**fullName**>Histone_h3_peptide_fragment</**fullName**>

<**alias type="display_short"**>EBI-6146769</**alias**>

<**alias type="display_long"**>EBI-6146769</**alias**>

</**names**>

<**xref**>

<**primaryRef id="EBI-6146769" refTypeAc="MI:0356" refType="identity" dbAc="MI:0469" db="intact"**/>

</**xref**>

<**interactorType**>

<**names**>

<**shortLabel**>peptide</**shortLabel**>

<**fullName**>peptide</**fullName**>

</**names**>

<**xref**>

<**primaryRef id="MI:0327" refTypeAc="MI:0356" refType="identity" dbAc="MI:0488" db="psi-mi"**/>

</**xref**>

</**interactorType**>

<**organism ncbiTaxId="9606"**>

<**names**>

<**shortLabel**>Human</**shortLabel**>

</**names**>

</**organism**>

<**sequence**>ARTKQTARK</**sequence**>

</**interactor**>

</**interactorList**>

<**interactionList**>

<**abstractInteraction id="11"**>

<**names**>

<**shortLabel**>Pygo1-Bcl9-1</**shortLabel**>

<**fullName**>B-cell CLL/lymphoma 9 protein-Pygopus homolog 1-1</**fullName**>

<**alias type="display_short"**>O00512-Q9Y3Y4-1</**alias**>

</**names**>

<**xref**>

<**primaryRef id="EBI-6142552" refTypeAc="MI:0356" refType="identity" dbAc="MI:0469" db="intact"**/>

<**secondaryRef id="IM-17354-3" refTypeAc="MI:0662" refType="imex-primary" dbAc="MI:0670" db="imex"**/>

<**secondaryRef id="2VPD" refTypeAc="MI:0356" refType="identity" dbAc="MI:0469" db="rcsb pdb"**/>

</**xref**>

<**participantList**>

<**participant id="21"**>

<**interactorRef**>1</**interactorRef**>

<**biologicalRole**>

<**names**>

<**shortLabel**>unspecified role</**shortLabel**>

<**fullName**>unspecified role</**fullName**>

</**names**>

<**xref**>

<**primaryRef id="MI:0499" refTypeAc="MI:0356" refType="identity" dbAc="MI:0488" db="psi-mi"**/>

</**xref**>

</**biologicalRole**>

<**featureList**>

<**feature id="31"**>

<**names**>

<**shortLabel**>Znf_PHD-finger</**shortLabel**>

<**fullName**>Zinc finger, PHD-finger</**fullName**>

</**names**>

<**xref**>

<**primaryRef id="IPR019787" refTypeAc="MI:0356" refType="identity" dbAc="MI:0449" db="interpro"**/>

</**xref**>

<**featureType**>

<**names**>

<**shortLabel**>sufficient binding region</**shortLabel**>

<**fullName**>sufficient binding region</**fullName**>

</**names**>

<**xref**>

<**primaryRef id="MI:0116" refTypeAc="MI:0356" refType="identity" dbAc="MI:0488" db="psi-mi"**/>

</**xref**>

</**featureType**>

<**featureRangeList**>

<**featureRange**>

<**startStatus**>

<**names**>

<**shortLabel**>certain</**shortLabel**>

<**fullName**>certain sequence position</**fullName**>

</**names**>

<**xref**>

<**primaryRef id="MI:0335" refTypeAc="MI:0356" refType="identity" dbAc="MI:0488" db="psi-mi"**/>

</**xref**>

</**startStatus**>

<**begin position="333"**/>

<**endStatus**>

<**names**>

<**shortLabel**>certain</**shortLabel**>

<**fullName**>certain sequence position</**fullName**>

</**names**>

<**xref**>

<**primaryRef id="MI:0335" refTypeAc="MI:0356" refType="identity" dbAc="MI:0488" db="psi-mi"**/>

</**xref**>

</**endStatus**>

<**end position="398"**/>

<**isLink**>false</**isLink**>

</**featureRange**>

</**featureRangeList**>

</**feature**>

</**featureList**>

<**stoichiometry value="1"**/>

</**participant**>

<**participant id="22"**>

<**interactorRef**>2</**interactorRef**>

<**biologicalRole**>

<**names**>

<**shortLabel**>unspecified role</**shortLabel**>

<**fullName**>unspecified role</**fullName**>

</**names**>

<**xref**>

<**primaryRef id="MI:0499" refTypeAc="MI:0356" refType="identity" dbAc="MI:0488" db="psi-mi"**/>

</**xref**>

</**biologicalRole**>

<**featureList**>

<**feature id="32"**>

<**names**>

<**shortLabel**>region</**shortLabel**>

<**fullName**>region</**fullName**>

</**names**>

<**featureType**>

<**names**>

<**shortLabel**>sufficient binding region</**shortLabel**>

<**fullName**>sufficient binding region</**fullName**>

</**names**>

<**xref**>

<**primaryRef id="MI:0116" refTypeAc="MI:0356" refType="identity" dbAc="MI:0488" db="psi-mi"**/>

</**xref**>

</**featureType**>

<**featureRangeList**>

<**featureRange**>

<**startStatus**>

<**names**>

<**shortLabel**>certain</**shortLabel**>

<**fullName**>certain sequence position</**fullName**>

</**names**>

<**xref**>

<**primaryRef id="MI:0335" refTypeAc="MI:0356" refType="identity" dbAc="MI:0488" db="psi-mi"**/>

</**xref**>

</**startStatus**>

<**begin position="174"**/>

<**endStatus**>

<**names**>

<**shortLabel**>certain</**shortLabel**>

<**fullName**>certain sequence position</**fullName**>

</**names**>

<**xref**>

<**primaryRef id="MI:0335" refTypeAc="MI:0356" refType="identity" dbAc="MI:0488" db="psi-mi"**/>

</**xref**>

</**endStatus**>

<**end position="205"**/>

<**isLink**>false</**isLink**>

</**featureRange**>

</**featureRangeList**>

</**feature**>

</**featureList**>

<**stoichiometry value="1"**/>

</**participant**>

</**participantList**>

<**bindingFeatureList**>

<**bindingFeatures**>

<**participantFeatureRef**>31</**participantFeatureRef**>

<**participantFeatureRef**>32</**participantFeatureRef**>

</**bindingFeatures**>

</**bindingFeatureList**>

<**interactionType**>

<**names**>

<**shortLabel**>direct interaction</**shortLabel**>

</**names**>

<**xref**>

<**primaryRef id="MI:0407" refTypeAc="MI:0356" refType="identity" dbAc="MI:0488" db="psi-mi"**/>

</**xref**>

</**interactionType**>

<**intraMolecular**>false</**intraMolecular**>

<**organism ncbiTaxId="9606"**>

<**names**>

<**shortLabel**>Human</**shortLabel**>

</**names**>

</**organism**>

<**cooperativeEffectList**>

<**allostery**>

<**cooperativityEvidenceList**>

<**cooperativityEvidenceDescription**>

<**bibref**>

<**xref**>

<**primaryRef id="18498752" refTypeAc="MI:0358" refType="primary-reference" dbAc="MI:0446" db="pubmed"**/>

</**xref**>

<**attributeList**>

<**attribute nameAc="MI:0636" name="author-list"**>Fiedler M., Sanchez-Barrena M.J.,

Nekrasov M., Mieszczanek J., Rybin V., Mueller J., Evans P., Bienz M.

</**attribute**>

<**attribute nameAc="MI:0634" name="contact-email"**>mb2@mrc-lmb.cam.ac.uk

</**attribute**>

<**attribute nameAc="MI:0885" name="journal"**>Mol Cell. (507-518)</**attribute**>

<**attribute nameAc="MI:0886" name="publication year"**>2008</**attribute**>

</**attributeList**>

</**bibref**>

<**evidenceMethodList**>

<**evidenceMethod**>

<**names**>

<**shortLabel**>experimental evidence</**shortLabel**>

</**names**>

<**xref**>

<**primaryRef id="ECO:0000006" refTypeAc="MI:0356" refType="identity" dbAc="MI:1331" db="evidence ontology"**/>

</**xref**>

</**evidenceMethod**>

<**evidenceMethod**>

<**names**>

<**shortLabel**>x-ray diffraction</**shortLabel**>

</**names**>

<**xref**>

<**primaryRef id="MI:0114" refTypeAc="MI:0356" refType="identity" dbAc="MI:0488" db="psi-mi"**/>

</**xref**>

</**evidenceMethod**>

<**evidenceMethod**>

<**names**>

<**shortLabel**>itc</**shortLabel**>

</**names**>

<**xref**>

<**primaryRef id="MI:0065" refTypeAc="MI:0356" refType="identity" dbAc="MI:0488" db="psi-mi"**/>

</**xref**>

</**evidenceMethod**>

</**evidenceMethodList**>

</**cooperativityEvidenceDescription**>

</**cooperativityEvidenceList**>

<**affectedInteractionList**>

<**affectedInteractionRef**>12</**affectedInteractionRef**>

</**affectedInteractionList**>

<**cooperativeEffectOutcome**>

<**names**>

<**shortLabel**>positive cooperative effect</**shortLabel**>

</**names**>

<**xref**>

<**primaryRef id="MI:1154" refTypeAc="MI:0356" refType="identity" dbAc="MI:0488" db="psi-mi"**/>

</**xref**>

</**cooperativeEffectOutcome**>

<**cooperativeEffectResponse**>

<**names**>

<**shortLabel**>allosteric k-type response</**shortLabel**>

</**names**>

<**xref**>

<**primaryRef id="MI:1162" refTypeAc="MI:0356" refType="identity" dbAc="MI:0488" db="psi-mi"**/>

</**xref**>

</**cooperativeEffectResponse**>

<**allostericMoleculeRef**>21</**allostericMoleculeRef**>

<**allostericEffectorRef**>22</**allostericEffectorRef**>

<**allostericMechanism**>

<**names**>

<**shortLabel**>allosteric change in structure</**shortLabel**>

</**names**>

<**xref**>

<**primaryRef id="MI:1165" refTypeAc="MI:0356" refType="identity" dbAc="MI:0488" db="psi-mi"**/>

</**xref**>

</**allostericMechanism**>

<**allosteryType**>

<**names**>

<**shortLabel**>heterotropic allostery</**shortLabel**>

</**names**>

<**xref**>

<**primaryRef id="MI:1168" refTypeAc="MI:0356" refType="identity" dbAc="MI:0488" db="psi-mi"**/>

</**xref**>

</**allosteryType**>

</**allostery**>

</**cooperativeEffectList**>

</**abstractInteraction**>

<**abstractInteraction id="12"**>

<**names**>

<**shortLabel**>Pygo1_Bcl9-H3K4me2-1</**shortLabel**>

<**fullName**>B-cell CLL/lymphoma 9 protein_Pygopus homolog 1-Histone_h3_peptide_fragment-1</**fullName**>

<**alias type="display_short"**>O00512_Q9Y3Y4-EBI-6146769-1</**alias**>

</**names**>

<**xref**>

<**primaryRef id="EBI-6146771" refTypeAc="MI:0356" refType="identity" dbAc="MI:0469" db="intact"**/>

<**secondaryRef id="IM-17354-4" refTypeAc="MI:0662" refType="imex-primary" dbAc="MI:0670" db="imex"**/>

<**secondaryRef id="2VPE" refTypeAc="MI:0356" refType="identity" dbAc="MI:0469" db="rcsb pdb"**/>

</**xref**>

<**participantList**>

<**participant id="23"**>

<**interactionRef**>11</**interactionRef**>

<**biologicalRole**>

<**names**>

<**shortLabel**>unspecified role</**shortLabel**>

<**fullName**>unspecified role</**fullName**>

</**names**>

<**xref**>

<**primaryRef id="MI:0499" refTypeAc="MI:0356" refType="identity" dbAc="MI:0488" db="psi-mi"**/>

</**xref**>

</**biologicalRole**>

<**featureList**>

<**feature id="33"**>

<**names**>

<**shortLabel**>Znf_PHD-finger</**shortLabel**>

<**fullName**>Zinc finger, PHD-finger</**fullName**>

</**names**>

<**xref**>

<**primaryRef id="IPR019787" refTypeAc="MI:0356" refType="identity" dbAc="MI:0449" db="interpro"**/>

</**xref**>

<**featureType**>

<**names**>

<**shortLabel**>sufficient binding region</**shortLabel**>

<**fullName**>sufficient binding region</**fullName**>

</**names**>

<**xref**>

<**primaryRef id="MI:0116" refTypeAc="MI:0356" refType="identity" dbAc="MI:0488" db="psi-mi"**/>

</**xref**>

</**featureType**>

<**featureRangeList**>

<**featureRange**>

<**startStatus**>

<**names**>

<**shortLabel**>certain</**shortLabel**>

<**fullName**>certain sequence position</**fullName**>

</**names**>

<**xref**>

<**primaryRef id="MI:0335" refTypeAc="MI:0356" refType="identity" dbAc="MI:0488" db="psi-mi"**/>

</**xref**>

</**startStatus**>

<**begin position="333"**/>

<**endStatus**>

<**names**>

<**shortLabel**>certain</**shortLabel**>

<**fullName**>certain sequence position</**fullName**>

</**names**>

<**xref**>

<**primaryRef id="MI:0335" refTypeAc="MI:0356" refType="identity" dbAc="MI:0488" db="psi-mi"**/>

</**xref**>

</**endStatus**>

<**end position="398"**/>

<**isLink**>false</**isLink**>

<**participantRef**>21</**participantRef**>

</**featureRange**>

</**featureRangeList**>

</**feature**>

</**featureList**>

<**stoichiometry value="1"**/>

</**participant**>

<**participant id="24"**>

<**interactorRef**>3</**interactorRef**>

<**biologicalRole**>

<**names**>

<**shortLabel**>unspecified role</**shortLabel**>

<**fullName**>unspecified role</**fullName**>

</**names**>

<**xref**>

<**primaryRef id="MI:0499" refTypeAc="MI:0356" refType="identity" dbAc="MI:0488" db="psi-mi"**/>

</**xref**>

</**biologicalRole**>

<**featureList**>

<**feature id="34"**>

<**names**>

<**shortLabel**>region</**shortLabel**>

<**fullName**>region</**fullName**>

</**names**>

<**featureType**>

<**names**>

<**shortLabel**>sufficient binding region</**shortLabel**>

<**fullName**>sufficient binding region</**fullName**>

</**names**>

<**xref**>

<**primaryRef id="MI:0116" refTypeAc="MI:0356" refType="identity" dbAc="MI:0488" db="psi-mi"**/>

</**xref**>

</**featureType**>

<**featureRangeList**>

<**featureRange**>

<**startStatus**>

<**names**>

<**shortLabel**>certain</**shortLabel**>

<**fullName**>certain sequence position</**fullName**>

</**names**>

<**xref**>

<**primaryRef id="MI:0335" refTypeAc="MI:0356" refType="identity" dbAc="MI:0488" db="psi-mi"**/>

</**xref**>

</**startStatus**>

<**begin position="1"**/>

<**endStatus**>

<**names**>

<**shortLabel**>certain</**shortLabel**>

<**fullName**>certain sequence position</**fullName**>

</**names**>

<**xref**>

<**primaryRef id="MI:0335" refTypeAc="MI:0356" refType="identity" dbAc="MI:0488" db="psi-mi"**/>

</**xref**>

</**endStatus**>

<**end position="7"**/>

<**isLink**>false</**isLink**>

</**featureRange**>

</**featureRangeList**>

</**feature**>

<**feature id="35"**>

<**names**>

<**shortLabel**>required-dimethyllys4</**shortLabel**>

<**fullName**>required-dimethyllys4</**fullName**>

</**names**>

<**featureType**>

<**names**>

<**shortLabel**>dimethylated residue</**shortLabel**>

<**fullName**>dimethylated residue</**fullName**>

</**names**>

<**xref**>

<**primaryRef id="MOD:00429" refTypeAc="MI:0356" refType="identity" dbAc="MI:0897" db="psi-mod"**/>

</**xref**>

</**featureType**>

<**featureRangeList**>

<**featureRange**>

<**startStatus**>

<**names**>

<**shortLabel**>certain</**shortLabel**>

<**fullName**>certain sequence position</**fullName**>

</**names**>

<**xref**>

<**primaryRef id="MI:0335" refTypeAc="MI:0356" refType="identity" dbAc="MI:0488" db="psi-mi"**/>

</**xref**>

</**startStatus**>

<**begin position="4"**/>

<**endStatus**>

<**names**>

<**shortLabel**>certain</**shortLabel**>

<**fullName**>certain sequence position</**fullName**>

</**names**>

<**xref**>

<**primaryRef id="MI:0335" refTypeAc="MI:0356" refType="identity" dbAc="MI:0488" db="psi-mi"**/>

</**xref**>

</**endStatus**>

<**end position="4"**/>

<**isLink**>false</**isLink**>

</**featureRange**>

</**featureRangeList**>

<**featureRole**>

<**names**>

<**shortLabel**>prerequisite-ptm</**shortLabel**>

</**names**>

<**xref**>

<**primaryRef id="MI:0638" refTypeAc="MI:0356" refType="identical object" dbAc="MI:0488" db="psi-mi"**/>

</**xref**>

</**featureRole**>

</**feature**>

</**featureList**>

<**stoichiometry value="1"**/>

</**participant**>

</**participantList**>

<**bindingFeatureList**>

<**bindingFeatures**>

<**participantFeatureRef**>33</**participantFeatureRef**>

<**participantFeatureRef**>34</**participantFeatureRef**>

</**bindingFeatures**>

</**bindingFeatureList**>

<**interactionType**>

<**names**>

<**shortLabel**>direct interaction</**shortLabel**>

</**names**>

<**xref**>

<**primaryRef id="MI:0407" refTypeAc="MI:0356" refType="identity" dbAc="MI:0488" db="psi-mi"**/>

</**xref**>

</**interactionType**>

<**parameterList**>

<**parameter uncertainty="1.1" factor="11.4" exponent="-6" base="10" unitAc="MI:0648" unit="M" termAc="MI:0646" term="Kd"**>

<**bibref**>

<**xref**>

<**primaryRef id="18498752" refTypeAc="MI:0358" refType="primary-reference" dbAc="MI:0446" db="pubmed"**/>

</**xref**>

</**bibref**>

</**parameter**>

</**parameterList**>

</**abstractInteraction**>

</**interactionList**>

</**entry**>

</**entrySet**>

<?xml version="1.0"?>

-<entrySet xsi:schemaLocation="http://psi.hupo.org/mi/mif http://psidev.sourceforge.net/mi/rel25/src/MIF254.xsd" level="2" version="5" minorVersion="4" xmlns:xsi="http://www.w3.org/2001/XMLSchema-instance" xmlns="http://psi.hupo.org/mi/mif">
 -<entry>
 -<interactorList>
 -<interactor id="1">
 -<names>
 <shortLabel>Pygo1</shortLabel>
 <fullName>Pygopus homolog 1</fullName>
 <alias type="gene name" typeAc="MI:0301">PYGO1</alias>
 <alias type="display_short">Q9Y3Y4</alias>
 <alias type="display_long">pygo1_human</alias>
 </names>
 -<xref>
 <primaryRef id="Q9Y3Y4" refTypeAc="MI:0356" refType="identity" dbAc="MI:0486" db="uniprotkb"/>
 <secondaryRef id="ENSG00000171016" refTypeAc="MI:0251" refType="gene product" dbAc="MI:0476" db="ensembl"/>
 <secondaryRef id="NP_056432.1" refTypeAc="MI:0356" refType="identity" dbAc="MI:0481" db="refseq"/>

 </xref>
 -<interactorType>
 -<names>

<shortLabel>protein</shortLabel>
 <fullName>protein</fullName>
</names>

-<xref>
 <primaryRef id="MI:0326" refTypeAc="MI:0356" refType="identity" dbAc="MI:0488" db="psi-mi"/>
</xref>

</interactorType>

-<organism ncbiTaxId="9606">
-<names>
 <shortLabel>Human</shortLabel>
</names>

</organism>

</interactor>

-<interactor id="2">

-<names>
<shortLabel>Bcl9</shortLabel>
<fullName>B-cell CLL/lymphoma 9 protein</fullName>
<alias type="gene name" typeAc="MI:0301">BCL9</alias>
<alias type="display_short">O00512</alias>
<alias type="display_long">bcl9_human</alias>

</names>

-<xref>
<primaryRef id="O00512" refTypeAc="MI:0356" refType="identity" dbAc="MI:0486" db="uniprotkb"/>
<secondaryRef id="ENSG00000116128" refTypeAc="MI:0251" refType="gene product" dbAc="MI:0476" db="ensembl"/>
<secondaryRef id="NP_004317.2" refTypeAc="MI:0356" refType="identity" dbAc="MI:0481" db="refseq"/>

</xref>

-<interactorType>
-<names>
 <shortLabel>protein</shortLabel>
 <fullName>protein</fullName>
</names>

-<xref>
<primaryRef id="MI:0326" refTypeAc="MI:0356" refType="identity" dbAc="MI:0488" db="psi-mi"/>

</xref>

</interactorType>
-<organism ncbiTaxId="9606">
 -<names>
 <shortLabel>Human</shortLabel>
 </names>
</organism>

</interactor>
 -<interactor id="3">
 -<names>
 <shortLabel>H3K4me2</shortLabel>
 <fullName>Histone_h3_peptide_fragment</fullName>
 <alias type="display_short">EBI-6146769</alias>
 <alias type="display_long">EBI-6146769</alias>
 </names>
 -<xref>
 <primaryRef id="EBI-6146769" refTypeAc="MI:0356" refType="identity" dbAc="MI:0469" db="intact"/>

</xref>
 -<interactorType>
 -<names>
 <shortLabel>peptide</shortLabel>
 <fullName>peptide</fullName>
 </names>
 -<xref>
 <primaryRef id="MI:0327" refTypeAc="MI:0356" refType="identity" dbAc="MI:0488" db="psi-mi"/>
 </xref>
</interactorType>
-<organism ncbiTaxId="9606">
 -<names>
 <shortLabel>Human</shortLabel>
 </names>
</organism>
<sequence>ARTKQTARK</sequence>

</interactor>
</interactorList>
-<interactionList>
 -<abstractInteraction id="11">
 -<names>
 <shortLabel>Pygo1-Bcl9-1</shortLabel>
 <fullName>B-cell CLL/lymphoma 9 protein-Pygopus homolog 1-1</fullName>
 <alias type="display_short">O00512-Q9Y3Y4-1</alias>
 </names>
 -<xref>
 <primaryRef id="EBI-6142552" refTypeAc="MI:0356" refType="identity" dbAc="MI:0469" db="intact"/>
 <secondaryRef id="IM-17354-3" refTypeAc="MI:0662" refType="imex-primary" dbAc="MI:0670" db="imex"/>
 <secondaryRef id="2VPD" refTypeAc="MI:0356" refType="identity" dbAc="MI:0469" db="rcsb pdb"/>
 </xref>
 -<participantList>
 -<participant id="21">
 <interactorRef>1</interactorRef>
 -<biologicalRole>
 -<names>
 <shortLabel>unspecified role</shortLabel>
 <fullName>unspecified role</fullName>
 </names>
 -<xref>
 <primaryRef id="MI:0499" refTypeAc="MI:0356" refType="identity" dbAc="MI:0488" db="psi-mi"/>
 </xref>
 </biologicalRole>
 -<biologicalFeatureList>
 -<biologicalFeature id="31">
 -<names>
 <shortLabel>Znf_PHD-finger</shortLabel>
 <fullName>Zinc finger, PHD-finger</fullName>
 </names>
 -<xref>
 <primaryRef id="IPR019787" refTypeAc="MI:0356" refType="identity" dbAc="MI:0449" db="interpro"/>
 </xref>
 -<featureType>
 -<names>
 <shortLabel>sufficient binding region</shortLabel>
 <fullName>sufficient binding region</fullName>
 </names>
 -<xref>
 <primaryRef id="MI:0116" refTypeAc="MI:0356" refType="identity" dbAc="MI:0488" db="psi-mi"/>
 </xref>
 </featureType>
 -<featureRangeList>
 -<featureRange>
 -<startStatus>
 -<names>
 <shortLabel>certain</shortLabel>
 <fullName>certain sequence position</fullName>
 </names>
 -<xref>
 <primaryRef id="MI:0335" refTypeAc="MI:0356" refType="identity" dbAc="MI:0488" db="psi-mi"/>
 </xref>
 </startStatus>
 <begin position="333"/>
 -<endStatus>
 -<names>
 <shortLabel>certain</shortLabel>
 <fullName>certain sequence position</fullName>
 </names>
 -<xref>
 <primaryRef id="MI:0335" refTypeAc="MI:0356" refType="identity" dbAc="MI:0488" db="psi-mi"/>
 </xref>
 </endStatus>
 <end position="398"/>
 <isLink>false</isLink>
 </featureRange>
 </featureRangeList>
 </biologicalFeature>
 </biologicalFeatureList>
 <stoichiometry value="1"/>
 </participant>
 -<participant id="22">
 <interactorRef>2</interactorRef>
 -<biologicalRole>

-<names>
 <shortLabel>unspecified role</shortLabel>
 <fullName>unspecified role</fullName>
 </names>
 -<xref>
 <primaryRef id="MI:0499" refTypeAc="MI:0356" refType="identity" dbAc="MI:0488" db="psi-mi"/>
 </xref>
</biologicalRole>
-<biologicalFeatureList>
 -<biologicalFeature id="32">
 -<names>
 <shortLabel>region</shortLabel>
 <fullName>region</fullName>
 </names>
 -<featureType>
 -<names>
 <shortLabel>sufficient binding region</shortLabel>
 <fullName>sufficient binding region</fullName>
 </names>
 -<xref>
 <primaryRef id="MI:0116" refTypeAc="MI:0356" refType="identity" dbAc="MI:0488" db="psi-mi"/>
 </xref>
 </featureType>
 -<featureRangeList>
 -<featureRange>
 -<startStatus>
 -<names>
 <shortLabel>certain</shortLabel>
 <fullName>certain sequence position</fullName>
 </names>
 -<xref>
 <primaryRef id="MI:0335" refTypeAc="MI:0356" refType="identity" dbAc="MI:0488" db="psi-mi"/>
 </xref>
 </startStatus>
 <begin position="174"/>
 -<endStatus>
 -<names>
 <shortLabel>certain</shortLabel>
 <fullName>certain sequence position</fullName>
 </names>
 -<xref>
 <primaryRef id="MI:0335" refTypeAc="MI:0356" refType="identity" dbAc="MI:0488" db="psi-mi"/>
 </xref>
 </endStatus>
 <end position="205"/>
 <isLink>false</isLink>
 </featureRange>
 </featureRangeList>
 </biologicalFeature>
</biologicalFeatureList>
<stoichiometry value="1"/>

</participant>

</participantList>

-<bindingFeatureList>
-<bindingFeature>
 <participantFeatureRef>31</participantFeatureRef>

<participantFeatureRef>32</participantFeatureRef>

</bindingFeature>

</bindingFeatureList>

-<interactionType>

-<names>

<shortLabel>direct interaction</shortLabel>

</names>

-<xref>

<primaryRef id="MI:0407" refTypeAc="MI:0356" refType="identity" dbAc="MI:0488" db="psi-mi"/>

</xref>

</interactionType>

<intraMolecular>false</intraMolecular>
 -<organism ncbiTaxId="9606">
 -<names>
 <shortLabel>Human</shortLabel>
 </names>
</organism>
-<cooperativeEffectList>
 -<allostery>
 -<cooperativityEvidenceList>
 -<cooperativityEvidenceDescription>
 -<bibref>
 -<xref>
 <primaryRef id="18498752" refTypeAc="MI:0358" refType="primary-reference" dbAc="MI:0446" db="pubmed"/>
 </xref>
 -<attributeList>
 <attribute nameAc="MI:0636" name="author-list">Fiedler M., Sanchez-Barrena M.J., Nekrasov M., Mieszczanek J., Rybin V., Mueller J., Evans P., Bienz M.</attribute>
 <attribute nameAc="MI:0634" name="contact-email">mb2@mrc-lmb.cam.ac.uk</attribute>
 <attribute nameAc="MI:0885" name="journal">Mol Cell. (507-518)</attribute>
 <attribute nameAc="MI:0886" name="publication year">2008</attribute>
 </attributeList>
 </bibref>
 -<evidenceCode>
 -<names>
 <shortLabel>experimental evidence</shortLabel>
 </names>
 -<xref>
 <primaryRef id="ECO:0000006" refTypeAc="MI:0356" refType="identity" dbAc="MI:1331" db="evidence ontology"/>
 </xref>
 </evidenceCode>
 -<evidenceMethodList>
 -<evidenceMethod>
 -<names>
 <shortLabel>x-ray diffraction</shortLabel>
 </names>
 -<xref>
 <primaryRef id="MI:0114" refTypeAc="MI:0356" refType="identity" dbAc="MI:0488" db="psi-mi"/>
 </xref>
 </evidenceMethod>
 -<evidenceMethod>
 -<names>
 <shortLabel>itc</shortLabel>
 </names>
 -<xref>
 <primaryRef id="MI:0065" refTypeAc="MI:0356" refType="identity" dbAc="MI:0488" db="psi-mi"/>
 </xref>
 </evidenceMethod>
 </evidenceMethodList>
 </cooperativityEvidenceDescription>
 </cooperativityEvidenceList>
 <affectedInteraction>12</affectedInteraction>
 -<cooperativeEffectOutcome>
 -<names>
 <shortLabel>positive cooperative effect</shortLabel>
 </names>
 -<xref>
 <primaryRef id="MI:1154" refTypeAc="MI:0356" refType="identity" dbAc="MI:0488" db="psi-mi"/>
 </xref>
 </cooperativeEffectOutcome>
 -<cooperativeEffectResponse>
 -<names>
 <shortLabel>allosteric k-type response</shortLabel>
 </names>
 -<xref>
 <primaryRef id="MI:1162" refTypeAc="MI:0356" refType="identity" dbAc="MI:0488" db="psi-mi"/>
 </xref>
 </cooperativeEffectResponse>
 <allostericMolecule>21</allostericMolecule>
 <allostericEffector>22</allostericEffector>
 -<allostericMechanism>


 -<names>
 <shortLabel>allosteric change in structure</shortLabel>
 </names>
 -<xref>
 <primaryRef id="MI:1165" refTypeAc="MI:0356" refType="identity" dbAc="MI:0488" db="psi-mi"/>
 </xref>
 </allostericMechanism>
 -<allosteryType>
 -<names>
 <shortLabel>heterotropic allostery</shortLabel>
 </names>
 -<xref>
 <primaryRef id="MI:1168" refTypeAc="MI:0356" refType="identity" dbAc="MI:0488" db="psi-mi"/>
 </xref>
 </allosteryType>
</allostery>

</cooperativeEffectList>

</abstractInteraction>

-<abstractInteraction id="12">

-<names>

<shortLabel>Pygo1_Bcl9-H3K4me2-1</shortLabel>

<fullName>B-cell CLL/lymphoma 9 protein_Pygopus homolog 1-Histone_h3_peptide_fragment-1</fullName>

<alias type="display_short">O00512_Q9Y3Y4-EBI-6146769-1</alias>

</names>

-<xref>

<primaryRef id="EBI-6146771" refTypeAc="MI:0356" refType="identity" dbAc="MI:0469" db="intact"/>

<secondaryRef id="IM-17354-4" refTypeAc="MI:0662" refType="imex-primary" dbAc="MI:0670" db="imex"/>

<secondaryRef id="2VPE" refTypeAc="MI:0356" refType="identity" dbAc="MI:0469" db="rcsb pdb"/>

</xref>

-<participantList>

-<participant id="23">

<abstractInteractionRef>11</abstractInteractionRef>

-<biologicalRole>

-<names>

<shortLabel>unspecified role</shortLabel>

<fullName>unspecified role</fullName>

</names>

-<xref>

<primaryRef id="MI:0499" refTypeAc="MI:0356" refType="identity" dbAc="MI:0488" db="psi-mi"/>

</xref>

</biologicalRole>

-<biologicalFeatureList>

-<biologicalFeature id="33">

-<names>

<shortLabel>Znf_PHD-finger</shortLabel>

<fullName>Zinc finger, PHD-finger</fullName>

</names>

-<xref>

<primaryRef id="IPR019787" refTypeAc="MI:0356" refType="identity" dbAc="MI:0449" db="interpro"/>

</xref>
-<featureType>

-<names>

<shortLabel>sufficient binding region</shortLabel>
 <fullName>sufficient binding region</fullName>
</names>
-<xref>
 <primaryRef id="MI:0116" refTypeAc="MI:0356" refType="identity" dbAc="MI:0488" db="psi-mi"/>
</xref>

</featureType>
-<featureRangeList>
 -<featureRange>
 -<startStatus>
 -<names>

<shortLabel>certain</shortLabel>
 <fullName>certain sequence position</fullName>
 </names>
 -<xref>
 <primaryRef id="MI:0335" refTypeAc="MI:0356" refType="identity" dbAc="MI:0488" db="psi-mi"/>
 </xref>
 </startStatus>
 <begin position="333"/>
 -<endStatus>
 -<names>
 <shortLabel>certain</shortLabel>
 <fullName>certain sequence position</fullName>
 </names>
 -<xref>
 <primaryRef id="MI:0335" refTypeAc="MI:0356" refType="identity" dbAc="MI:0488" db="psi-mi"/>
 </xref>

</endStatus>
 <end position="398"/>
 <isLink>false</isLink>
 <participantRef>21</participantRef>
 </featureRange>
 </featureRangeList>
</biologicalFeature>

</biologicalFeatureList>

-<hostOrganismList>
 -<hostOrganism ncbiTaxId="9606">
 -<compartment>
 -<names>
 <shortLabel>nucleus</shortLabel>
 </names>
 -<xref>

<primaryRef id="GO:0005634" refTypeAc="MI:0354" refType="gene ontology term for cellular component" dbAc="MI:0448" db="gene ontology"/>
 </xref>
 </compartment>

</hostOrganism>

</hostOrganismList>

<stoichiometry value="1"/>

</participant>

-<participant id="24">

<interactorRef>3</interactorRef>

-<biologicalRole>

<names>

<shortLabel>unspecified role</shortLabel>

<fullName>unspecified role</fullName>

</names>

-<xref>

<primaryRef id="MI:0499" refTypeAc="MI:0356" refType="identity" dbAc="MI:0488" db="psi-mi"/>

</xref>

</biologicalRole>

-<biologicalFeatureList>

-<biologicalFeature id="34">

-<names>

<shortLabel>region</shortLabel>

<fullName>region</fullName>

</names>

-<featureType>

-<names>
 <shortLabel>sufficient binding region</shortLabel>
 <fullName>sufficient binding region</fullName>
 </names>
-<xref>
 <primaryRef id="MI:0116" refTypeAc="MI:0356" refType="identity" dbAc="MI:0488" db="psi-mi"/>
</xref>

</featureType>

-<featureRangeList>

-<featureRange>

-<startStatus>

-<names>

<shortLabel>certain</shortLabel>
 <fullName>certain sequence position</fullName>

</names>

-<xref>
 <primaryRef id="MI:0335" refTypeAc="MI:0356" refType="identity" dbAc="MI:0488" db="psi-mi"/>

</xref>
 </startStatus>
 <begin position="1"/>
 -<endStatus>
 -<names>
 <shortLabel>certain</shortLabel>

<fullName>certain sequence position</fullName>
 </names>
 -<xref>
 <primaryRef id="MI:0335" refTypeAc="MI:0356" refType="identity" dbAc="MI:0488" db="psi-mi"/>
 </xref>
 </endStatus>
 <end position="7"/>
 <isLink>false</isLink>
 </featureRange>
 </featureRangeList>
 </biologicalFeature>
 -<biologicalFeature id="35">
 -<names>
 <shortLabel>required-dimethyllys4</shortLabel>
 <fullName>required-dimethyllys4</fullName>
 </names>

-<featureType>
 -<names>
 <shortLabel>dimethylated residue</shortLabel>

<fullName>dimethylated residue</fullName>
 </names>
 -<xref>
 <primaryRef id="MOD:00429" refTypeAc="MI:0356" refType="identity" dbAc="MI:0897" db="psi-mod"/>
 </xref>
 </featureType>
 -<featureRangeList>
 -<featureRange>
 -<startStatus>
 -<names>
 <shortLabel>certain</shortLabel>
 <fullName>certain sequence position</fullName>
 </names>
 -<xref>
 <primaryRef id="MI:0335" refTypeAc="MI:0356" refType="identity" dbAc="MI:0488" db="psi-mi"/>
 </xref>
 </startStatus>
 <begin position="4"/>
 -<endStatus>
 -<names>
 <shortLabel>certain</shortLabel>
 <fullName>certain sequence position</fullName>
 </names>
 -<xref>
 <primaryRef id="MI:0335" refTypeAc="MI:0356" refType="identity" dbAc="MI:0488" db="psi-mi"/>
 </xref>
 </endStatus>
 <end position="4"/>
 <isLink>false</isLink>
 </featureRange>
 </featureRangeList>
 -<featureRole>
 -<names>
 <shortLabel>prerequisite-ptm</shortLabel>
 </names>
 -<xref>
 <primaryRef id="MI:0638" refTypeAc="MI:0356" refType="identical object" dbAc="MI:0488" db="psi-mi"/>
 </xref>
 </featureRole>
 </biologicalFeature>
 </biologicalFeatureList>
 -<hostOrganismList>
 -<hostOrganism ncbiTaxId="9606">
 -<compartment>
 -<names>
 <shortLabel>nucleus</shortLabel>

</names>
 -<xref>
 <primaryRef id="GO:0005634" refTypeAc="MI:0354" refType="gene ontology term for cellular component" dbAc="MI:0448" db="gene ontology"/>
 </xref>

</compartment>
 </hostOrganism>
 </hostOrganismList>
 <stoichiometry value="1"/>
 </participant>
 </participantList>
 -<bindingFeatureList>
 -<bindingFeature>
 <participantFeatureRef>33</participantFeatureRef>
 <participantFeatureRef>34</participantFeatureRef>
 </bindingFeature>
 </bindingFeatureList>
 -<interactionType>
 -<names>
 <shortLabel>direct interaction</shortLabel>
 </names>
 -<xref>
 <primaryRef id="MI:0407" refTypeAc="MI:0356" refType="identity" dbAc="MI:0488" db="psi-mi"/>
 </xref>
 </interactionType>
 -<parameterList>
 -<parameter uncertainty="1.1" factor="11.4" exponent="-6" base="10" unitAc="MI:0648" unit="M" termAc="MI:0646" term="Kd">
 -<bibref>
 -<xref>
 <primaryRef id="18498752" refTypeAc="MI:0358" refType="primary-reference" dbAc="MI:0446" db="pubmed"/>
 </xref>
 </bibref>
 </parameter>
 </parameterList>
 </abstractInteraction>
 </interactionList>
 </entry>
 </entrySet>
