# Supplementary material for: Encompassing new use cases - level 3.0 of the HUPO-PSI format for molecular interactions
Source: BMC Bioinformatics. 2018 Apr 11;19:134. doi: 10.1186/s12859-018-2118-1 (PMC5896046; doi:10.1186/s12859-018-2118-1)
Supplement: Supplementary file 9 — Representation of molecule sets i.e. cases where a participant may be one of a list of molecules (use case 1.3i). (https://github.com/HUPO-PSI/miXML/blob/master/3.0/pub/Appendix%2010.docx). (DOCX 25 kb) [file 12859_2018_2118_MOESM9_ESM.docx]

**Molecule sets**

The ability to describe molecule sets i.e. cases where a participant may be one of a list of molecules rather than an unambiguous identification of a single molecule.

PMID: 23892143

Experiment EBI-11328252 Interaction EBI-11361048

<**interactor id="2"**>

<**names**>

<**shortLabel**>hspa1a_hspa1b_human</**shortLabel**>

<**fullName**>HSPA1A/HSPA1B identical protein</**fullName**>

</**names**>

<**xref**>

<**primaryRef db="intact" dbAc="MI:0469" id="EBI-11820565" refType="identity" refTypeAc="MI:0356"**/>

<**secondaryRef db="uniprotkb" dbAc="MI:0486" id="P0DMV8" refType="set member" refTypeAc="MI:1341"**/>

</**xref**>

<**interactorType**>

<**names**>

<**shortLabel**>molecule set</**shortLabel**>

<**fullName**>molecule set</**fullName**>

</**names**>

<**xref**>

<**primaryRef db="psi-mi" dbAc="MI:0488" id="MI:1304" refType="identity" refTypeAc="MI:0356"**/>

<**secondaryRef db="intact" dbAc="MI:0469" id="EBI-6862256" refType="identity" refTypeAc="MI:0356"**/>

<**secondaryRef db="pubmed" dbAc="MI:0446" id="14755292" refType="primary-reference" refTypeAc="MI:0358"**/>

</**xref**>

</**interactorType**>

<**organism ncbiTaxId="9606"**>

<**names**>

<**shortLabel**>human</**shortLabel**>

<**fullName**>Homo sapiens</**fullName**>

<**alias type="synonym" typeAc="MI:1041"**>Human</**alias**>

</**names**>

</**organism**>

</**interactor**>

File:

*<?***xml version='1.0' encoding='UTF-8'***?>*

<**entrySet xmlns:xsi="http://www.w3.org/2001/XMLSchema-instance" xmlns="http://psi.hupo.org/mi/mif300"**

**xsi:schemaLocation="http://psi.hupo.org/mi/mif300 https://raw.githubusercontent.com/HUPO-PSI/miXML/master/3.0/src/MIF300.xsd"**

**level="3" version="0" minorVersion="0"**>

<**entry**>

<**source releaseDate="2017-05-18"**>

<**names**>

<**shortLabel**>HPIDb</**shortLabel**>

<**fullName**>Host Pathogen Interaction database</**fullName**>

</**names**>

<**xref**>

<**primaryRef db="psi-mi" dbAc="MI:0488" id="MI:1335" refType="identity" refTypeAc="MI:0356"**/>

<**secondaryRef db="intact" dbAc="MI:0469" id="EBI-6598961" refType="identity" refTypeAc="MI:0356"**/>

</**xref**>

<**attributeList**>

<**attribute name="url" nameAc="MI:0614"**>http://www.agbase.msstate.edu/hpi/main.html</**attribute**>

</**attributeList**>

</**source**>

<**experimentList**>

<**experimentDescription id="1"**>

<**names**>

<**fullName**>Human respiratory syncytial virus N, P and M protein interactions in HEK-293T cells.</**fullName**>

</**names**>

<**bibref**>

<**xref**>

<**primaryRef db="pubmed" dbAc="MI:0446" id="23892143" refType="primary-reference" refTypeAc="MI:0358"**/>

<**secondaryRef db="intact" dbAc="MI:0469" id="EBI-11327913" refType="identity" refTypeAc="MI:0356"**/>

</**xref**>

<**attributeList**>

<**attribute name="publication title" nameAc="MI:1091"**>

Human respiratory syncytial virus N, P and M protein interactions in HEK-293T cells.

</**attribute**>

<**attribute name="journal" nameAc="MI:0885"**>Virus research</**attribute**>

<**attribute name="publication year" nameAc="MI:0886"**>2013</**attribute**>

<**attribute name="curation depth" nameAc="MI:0955"**>imex curation</**attribute**>

<**attribute name="imex curation" nameAc="MI:0959"**/>

<**attribute name="author-list" nameAc="MI:0636"**>

Oliveira AP., Simabuco FM., Tamura RE., Guerrero MC., Ribeiro PG., Libermann TA., Zerbini LF., Ventura AM.

</**attribute**>

<**attribute name="dataset" nameAc="MI:0875"**>Virus - Publications including interactions involving viral proteins</**attribute**>

<**attribute name="contact-email" nameAc="MI:0634"**>amventur@usp.br, amventur@gmail.com</**attribute**>

<**attribute name="data-processing" nameAc="MI:0633"**>

Viral proteins N,P, and M, with optimized codons for expression in human cells retain >99% sequence homology with HRSV (strain A2) proteins.

</**attribute**>

<**attribute name="author-announcement"**>03-Dec-2015: Contacted by IntAct-Help.</**attribute**>

</**attributeList**>

</**bibref**>

<**xref**>

<**primaryRef db="pubmed" dbAc="MI:0446" id="23892143" refType="primary-reference" refTypeAc="MI:0358"**/>

</**xref**>

<**hostOrganismList**>

<**hostOrganism ncbiTaxId="9606"**>

<**names**>

<**shortLabel**>human-293t</**shortLabel**>

<**fullName**>Homo sapiens 293 cells transformed with SV40 large T antigen</**fullName**>

</**names**>

<**cellType**>

<**names**>

<**shortLabel**>293t</**shortLabel**>

<**fullName**>293 cells expressing SV40 large T antigen.</**fullName**>

</**names**>

<**xref**>

<**primaryRef db="cabri" dbAc="MI:0246" id="ICLC HTL04001" refType="identity" refTypeAc="MI:0356"**/>

<**secondaryRef db="intact" dbAc="MI:0469" id="IA:0074" refType="identity" refTypeAc="MI:0356"**/>

<**secondaryRef db="cabri" dbAc="MI:0246" id="ACC 635" refType="identity" refTypeAc="MI:0356"**/>

<**secondaryRef db="mint" dbAc="MI:0471" id="MINT-1891516" refType="identity" refTypeAc="MI:0356"**/>

<**secondaryRef db="intact" dbAc="MI:0469" id="EBI-308000" refType="identity" refTypeAc="MI:0356"**/>

<**secondaryRef db="pubmed" dbAc="MI:0446" id="3031469" refType="primary-reference" refTypeAc="MI:0358"**/>

</**xref**>

<**attributeList**>

<**attribute name="comment" nameAc="MI:0612"**>

The original designation of this cell line was 293tsA1609neo. It is not in Cabri,ATCC or HyperCLDB.

</**attribute**>

</**attributeList**>

</**cellType**>

</**hostOrganism**>

</**hostOrganismList**>

<**interactionDetectionMethod**>

<**names**>

<**shortLabel**>anti bait coip</**shortLabel**>

<**fullName**>anti bait coimmunoprecipitation</**fullName**>

</**names**>

<**xref**>

<**primaryRef db="psi-mi" dbAc="MI:0488" id="MI:0006" refType="identity" refTypeAc="MI:0356"**/>

<**secondaryRef db="intact" dbAc="MI:0469" id="EBI-976" refType="identity" refTypeAc="MI:0356"**/>

<**secondaryRef db="pubmed" dbAc="MI:0446" id="7708014" refType="primary-reference" refTypeAc="MI:0358"**/>

</**xref**>

</**interactionDetectionMethod**>

<**participantIdentificationMethod**>

<**names**>

<**shortLabel**>western blot</**shortLabel**>

<**fullName**>western blot</**fullName**>

<**alias type="go synonym" typeAc="MI:0303"**>Immuno blot</**alias**>

</**names**>

<**xref**>

<**primaryRef db="psi-mi" dbAc="MI:0488" id="MI:0113" refType="identity" refTypeAc="MI:0356"**/>

<**secondaryRef db="intact" dbAc="MI:0469" id="EBI-973" refType="identity" refTypeAc="MI:0356"**/>

<**secondaryRef db="pubmed" dbAc="MI:0446" id="14755292" refType="primary-reference" refTypeAc="MI:0358"**/>

</**xref**>

</**participantIdentificationMethod**>

<**attributeList**>

<**attribute name="journal" nameAc="MI:0885"**>Virus research</**attribute**>

<**attribute name="publication year" nameAc="MI:0886"**>2013</**attribute**>

<**attribute name="dataset" nameAc="MI:0875"**>Virus - Publications including interactions involving viral proteins</**attribute**>

<**attribute name="curation depth" nameAc="MI:0955"**>imex curation</**attribute**>

<**attribute name="author-list" nameAc="MI:0636"**>

Oliveira AP., Simabuco FM., Tamura RE., Guerrero MC., Ribeiro PG., Libermann TA., Zerbini LF., Ventura AM.

</**attribute**>

<**attribute name="contact-email" nameAc="MI:0634"**>amventur@usp.br, amventur@gmail.com</**attribute**>

<**attribute name="accepted"**>Accepted 2015-NOV-09 AT 10:28 GMT AT 10:28 GMT by MGT</**attribute**>

<**attribute name="data-processing" nameAc="MI:0633"**>

Viral proteins N,P, and M, with optimized codons for expression in human cells retain >99% sequence homology with HRSV (strain A2) proteins.

</**attribute**>

<**attribute name="correction comment"**/>

</**attributeList**>

</**experimentDescription**>

</**experimentList**>

<**interactorList**>

<**interactor id="2"**>

<**names**>

<**shortLabel**>hspa1a_hspa1b_human</**shortLabel**>

<**fullName**>HSPA1A/HSPA1B identical protein</**fullName**>

</**names**>

<**xref**>

<**primaryRef db="intact" dbAc="MI:0469" id="EBI-11820565" refType="identity" refTypeAc="MI:0356"**/>

<**secondaryRef db="uniprotkb" dbAc="MI:0486" id="P0DMV8" refType="set member" refTypeAc="MI:1341"**/>

</**xref**>

<**interactorType**>

<**names**>

<**shortLabel**>molecule set</**shortLabel**>

<**fullName**>molecule set</**fullName**>

</**names**>

<**xref**>

<**primaryRef db="psi-mi" dbAc="MI:0488" id="MI:1304" refType="identity" refTypeAc="MI:0356"**/>

<**secondaryRef db="intact" dbAc="MI:0469" id="EBI-6862256" refType="identity" refTypeAc="MI:0356"**/>

<**secondaryRef db="pubmed" dbAc="MI:0446" id="14755292" refType="primary-reference" refTypeAc="MI:0358"**/>

</**xref**>

</**interactorType**>

<**organism ncbiTaxId="9606"**>

<**names**>

<**shortLabel**>human</**shortLabel**>

<**fullName**>Homo sapiens</**fullName**>

<**alias type="synonym" typeAc="MI:1041"**>Human</**alias**>

</**names**>

</**organism**>

</**interactor**>

<**interactor id="3"**>

<**names**>

<**shortLabel**>ncap_hrsva</**shortLabel**>

<**fullName**>Nucleoprotein</**fullName**>

<**alias type="gene name" typeAc="MI:0301"**>N</**alias**>

<**alias type="gene name synonym" typeAc="MI:0302"**>Nucleocapsid protein</**alias**>

</**names**>

<**xref**>

<**primaryRef db="uniprotkb" dbAc="MI:0486" id="P03418" version="SP_62" refType="identity" refTypeAc="MI:0356"**/>

<**secondaryRef db="uniprotkb" dbAc="MI:0486" id="P88810" version="SP_62" refType="secondary-ac" refTypeAc="MI:0360"**/>

<**secondaryRef db="intact" dbAc="MI:0469" id="EBI-6930799" refType="identity" refTypeAc="MI:0356"**/>

<**secondaryRef db="rcsb pdb" dbAc="MI:0460" id="4UC6"**/>

<**secondaryRef db="rcsb pdb" dbAc="MI:0460" id="4UC7"**/>

<**secondaryRef db="rcsb pdb" dbAc="MI:0460" id="4UC8"**/>

<**secondaryRef db="rcsb pdb" dbAc="MI:0460" id="4UC9"**/>

<**secondaryRef db="rcsb pdb" dbAc="MI:0460" id="4UCA"**/>

<**secondaryRef db="rcsb pdb" dbAc="MI:0460" id="4UCB"**/>

<**secondaryRef db="rcsb pdb" dbAc="MI:0460" id="4UCC"**/>

<**secondaryRef db="rcsb pdb" dbAc="MI:0460" id="4UCD"**/>

<**secondaryRef db="rcsb pdb" dbAc="MI:0460" id="4UCE"**/>

<**secondaryRef db="rcsb pdb" dbAc="MI:0460" id="4BKK"**/>

<**secondaryRef db="interpro" dbAc="MI:0449" id="IPR004930"**/>

<**secondaryRef db="rcsb pdb" dbAc="MI:0460" id="2WJ8"**/>

<**secondaryRef db="rcsb pdb" dbAc="MI:0460" id="2YHM"**/>

<**secondaryRef db="go" dbAc="MI:0448" id="GO:0030430"**/>

<**secondaryRef db="go" dbAc="MI:0448" id="GO:0019013"**/>

<**secondaryRef db="go" dbAc="MI:0448" id="GO:0003723"**/>

<**secondaryRef db="go" dbAc="MI:0448" id="GO:0039580"**/>

<**secondaryRef db="go" dbAc="MI:0448" id="GO:0039502"**/>

<**secondaryRef db="go" dbAc="MI:0448" id="GO:0019029"**/>

</**xref**>

<**interactorType**>

<**names**>

<**shortLabel**>protein</**shortLabel**>

<**fullName**>protein</**fullName**>

</**names**>

<**xref**>

<**primaryRef db="psi-mi" dbAc="MI:0488" id="MI:0326" refType="identity" refTypeAc="MI:0356"**/>

<**secondaryRef db="intact" dbAc="MI:0469" id="EBI-619654" refType="identity" refTypeAc="MI:0356"**/>

<**secondaryRef db="pubmed" dbAc="MI:0446" id="14755292" refType="primary-reference" refTypeAc="MI:0358"**/>

<**secondaryRef db="so" dbAc="MI:0601" id="SO:0000358" refType="see-also" refTypeAc="MI:0361"**/>

</**xref**>

</**interactorType**>

<**organism ncbiTaxId="11259"**>

<**names**>

<**shortLabel**>hrsva</**shortLabel**>

<**fullName**>human respiratory sy</**fullName**>

</**names**>

</**organism**>

<**sequence**>

MALSKVKLNDTLNKDQLLSSSKYTIQRSTGDSIDTPNYDVQKHINKLCGMLLITEDANHKFTGLIGMLYAMSRLGREDTIKILRDAGYHVKANGVDVTTHRQDINGKEMKFEVLTLASLTTEIQINIEIESRKSYKKMLKEMGEVAPEYRHDSPDCGMIILCIAALVITKLAAGDRSGLTAVIRRANNVLKNEMKRYKGLLPKDIANSFYEVFEKHPHFIDVFVHFGIAQSSTRGGSRVEGIFAGLFMNAYGAGQVMLRWGVLAKSVKNIMLGHASVQAEMEQVVEVYEYAQKLGGEAGFYHILNNPKASLLSLTQFPHFSSVVLGNAAGLGIMGEYRGTPRNQDLYDAAKAYAEQLKENGVINYSVLDLTAEELEAIKHQLNPKDNDVEL

</**sequence**>

<**attributeList**>

<**attribute name="crc64"**>D06E84F4F88D382B</**attribute**>

</**attributeList**>

</**interactor**>

</**interactorList**>

<**interactionList**>

<**interaction id="4"**>

<**names**>

<**shortLabel**>n-hs71a_human_hs71b_human_1-1</**shortLabel**>

</**names**>

<**xref**>

<**primaryRef db="intact" dbAc="MI:0469" id="EBI-11361048" refType="identity" refTypeAc="MI:0356"**/>

</**xref**>

<**experimentList**>

<**experimentRef**>1</**experimentRef**>

</**experimentList**>

<**participantList**>

<**participant id="5"**>

<**interactorRef**>3</**interactorRef**>

<**biologicalRole**>

<**names**>

<**shortLabel**>unspecified role</**shortLabel**>

<**fullName**>unspecified role</**fullName**>

</**names**>

<**xref**>

<**primaryRef db="psi-mi" dbAc="MI:0488" id="MI:0499" refType="identity" refTypeAc="MI:0356"**/>

<**secondaryRef db="intact" dbAc="MI:0469" id="EBI-77781" refType="identity" refTypeAc="MI:0356"**/>

<**secondaryRef db="pubmed" dbAc="MI:0446" id="14755292" refType="primary-reference" refTypeAc="MI:0358"**/>

</**xref**>

</**biologicalRole**>

<**experimentalRoleList**>

<**experimentalRole**>

<**names**>

<**shortLabel**>bait</**shortLabel**>

<**fullName**>bait</**fullName**>

</**names**>

<**xref**>

<**primaryRef db="psi-mi" dbAc="MI:0488" id="MI:0496" refType="identity" refTypeAc="MI:0356"**/>

<**secondaryRef db="intact" dbAc="MI:0469" id="EBI-49" refType="identity" refTypeAc="MI:0356"**/>

<**secondaryRef db="pubmed" dbAc="MI:0446" id="14755292" refType="primary-reference" refTypeAc="MI:0358"**/>

</**xref**>

</**experimentalRole**>

</**experimentalRoleList**>

</**participant**>

<**participant id="6"**>

<**interactorRef**>2</**interactorRef**>

<**biologicalRole**>

<**names**>

<**shortLabel**>unspecified role</**shortLabel**>

<**fullName**>unspecified role</**fullName**>

</**names**>

<**xref**>

<**primaryRef db="psi-mi" dbAc="MI:0488" id="MI:0499" refType="identity" refTypeAc="MI:0356"**/>

<**secondaryRef db="intact" dbAc="MI:0469" id="EBI-77781" refType="identity" refTypeAc="MI:0356"**/>

<**secondaryRef db="pubmed" dbAc="MI:0446" id="14755292" refType="primary-reference" refTypeAc="MI:0358"**/>

</**xref**>

</**biologicalRole**>

<**experimentalRoleList**>

<**experimentalRole**>

<**names**>

<**shortLabel**>prey</**shortLabel**>

<**fullName**>prey</**fullName**>

</**names**>

<**xref**>

<**primaryRef db="psi-mi" dbAc="MI:0488" id="MI:0498" refType="identity" refTypeAc="MI:0356"**/>

<**secondaryRef db="intact" dbAc="MI:0469" id="EBI-58" refType="identity" refTypeAc="MI:0356"**/>

<**secondaryRef db="pubmed" dbAc="MI:0446" id="14755292" refType="primary-reference" refTypeAc="MI:0358"**/>

</**xref**>

</**experimentalRole**>

</**experimentalRoleList**>

</**participant**>

</**participantList**>

<**interactionType**>

<**names**>

<**shortLabel**>physical association</**shortLabel**>

<**fullName**>physical association</**fullName**>

</**names**>

<**xref**>

<**primaryRef db="psi-mi" dbAc="MI:0488" id="MI:0915" refType="identity" refTypeAc="MI:0356"**/>

<**secondaryRef db="intact" dbAc="MI:0469" id="EBI-1813147" refType="identity" refTypeAc="MI:0356"**/>

<**secondaryRef db="pubmed" dbAc="MI:0446" id="14755292" refType="primary-reference" refTypeAc="MI:0358"**/>

</**xref**>

</**interactionType**>

<**attributeList**>

<**attribute name="figure legend" nameAc="MI:0599"**>2A and S4</**attribute**>

</**attributeList**>

</**interaction**>

</**interactionList**>

</**entry**>

</**entrySet**>
