# Supplementary material for: Encompassing new use cases - level 3.0 of the HUPO-PSI format for molecular interactions
Source: BMC Bioinformatics. 2018 Apr 11;19:134. doi: 10.1186/s12859-018-2118-1 (PMC5896046; doi:10.1186/s12859-018-2118-1)
Supplement: Supplementary file 10 — Representation of the systematic capture of the stoichiometry of molecules within an interaction (use case 1.3j). (https://github.com/HUPO-PSI/miXML/blob/master/3.0/pub/Appendix%2011.docx). (DOCX 31 kb) [file 12859_2018_2118_MOESM10_ESM.docx]

**Stoichiometry**

The systematic capture of the stoichiometry of molecules within an interaction

PMID:26575439

Experiment EBI-11512968 Interaction EBI-11657948

<**participant id="7"**>

<**interactorRef**>3</**interactorRef**>

<**biologicalRole**>

<**names**>

<**shortLabel**>unspecified role</**shortLabel**>

<**fullName**>unspecified role</**fullName**>

</**names**>

<**xref**>

<**primaryRef db="psi-mi" dbAc="MI:0488" id="MI:0499" refType="identity" refTypeAc="MI:0356"**/>

<**secondaryRef db="intact" dbAc="MI:0469" id="EBI-77781" refType="identity" refTypeAc="MI:0356"**/>

<**secondaryRef db="pubmed" dbAc="MI:0446" id="14755292" refType="primary-reference" refTypeAc="MI:0358"**/>

</**xref**>

</**biologicalRole**>

<**experimentalRoleList**>

<**experimentalRole**>

<**names**>

<**shortLabel**>neutral component</**shortLabel**>

<**fullName**>neutral component</**fullName**>

</**names**>

<**xref**>

<**primaryRef db="psi-mi" dbAc="MI:0488" id="MI:0497" refType="identity" refTypeAc="MI:0356"**/>

<**secondaryRef db="intact" dbAc="MI:0469" id="EBI-55" refType="identity" refTypeAc="MI:0356"**/>

<**secondaryRef db="pubmed" dbAc="MI:0446" id="14755292" refType="primary-reference" refTypeAc="MI:0358"**/>

</**xref**>

</**experimentalRole**>

</**experimentalRoleList**>

<**hostOrganismList**>

<**hostOrganism ncbiTaxId="7108"**>

<**names**>

<**shortLabel**>spofr-sf_21</**shortLabel**>

<**fullName**>Spodoptera frugiperda insect cells</**fullName**>

</**names**>

<**cellType**>

<**names**>

<**shortLabel**>sf_21</**shortLabel**>

<**fullName**>Insect cells</**fullName**>

</**names**>

<**xref**>

<**primaryRef db="cabri" dbAc="MI:0246" id="ACC 119" refType="identity" refTypeAc="MI:0356"**/>

<**secondaryRef db="intact" dbAc="MI:0469" id="IA:0072" refType="identity" refTypeAc="MI:0356"**/>

<**secondaryRef db="mint" dbAc="MI:0471" id="MINT-7501077" refType="identity" refTypeAc="MI:0356"**/>

<**secondaryRef db="intact" dbAc="MI:0469" id="EBI-307869" refType="identity" refTypeAc="MI:0356"**/>

</**xref**>

<**attributeList**>

<**attribute name="comment" nameAc="MI:0612"**>

derived from immature ovaries of fall armyworm Spodoptera frugiperda pupae.

</**attribute**>

<**attribute name="comment" nameAc="MI:0612"**>full name: IPLB-SF-21-AE</**attribute**>

<**attribute name="comment" nameAc="MI:0612"**>cells are susceptible to Baculovirus infection.</**attribute**>

</**attributeList**>

</**cellType**>

</**hostOrganism**>

</**hostOrganismList**>

<**stoichiometry value="1"**/>

</**participant**>

File:

*<?***xml version='1.0' encoding='UTF-8'***?>*

<**entrySet xmlns:xsi="http://www.w3.org/2001/XMLSchema-instance" xmlns="http://psi.hupo.org/mi/mif300"**

**xsi:schemaLocation="http://psi.hupo.org/mi/mif300 https://raw.githubusercontent.com/HUPO-PSI/miXML/master/3.0/src/MIF300.xsd"**

**level="3" version="0" minorVersion="0"**>

<**entry**>

<**source releaseDate="2017-05-18"**>

<**names**>

<**shortLabel**>matrixdb</**shortLabel**>

</**names**>

<**bibref**>

<**xref**>

<**primaryRef db="pubmed" dbAc="MI:0446" id="19147664" refType="primary-reference" refTypeAc="MI:0358"**/>

</**xref**>

</**bibref**>

<**xref**>

<**primaryRef db="psi-mi" dbAc="MI:0488" id="MI:0917" refType="identity" refTypeAc="MI:0356"**/>

<**secondaryRef db="intact" dbAc="MI:0469" id="EBI-2566319" refType="identity" refTypeAc="MI:0356"**/>

<**secondaryRef db="pubmed" dbAc="MI:0446" id="19147664" refType="primary-reference" refTypeAc="MI:0358"**/>

</**xref**>

<**attributeList**>

<**attribute name="postaladdress"**>

Institut de Biologie et de Chimie des Prot\u00c3\u00a9ines, 7, passage du Vercors 69 367 Lyon cedex 07 FRANCE

</**attribute**>

<**attribute name="email"**>matrixdb@ibcp.fr</**attribute**>

<**attribute name="url" nameAc="MI:0614"**>http://matrixdb.ibcp.fr</**attribute**>

</**attributeList**>

</**source**>

<**experimentList**>

<**experimentDescription id="1"**>

<**names**>

<**fullName**>Structural characterization of human heparanase reveals insights into substrate recognition.</**fullName**>

</**names**>

<**bibref**>

<**xref**>

<**primaryRef db="pubmed" dbAc="MI:0446" id="26575439" refType="primary-reference" refTypeAc="MI:0358"**/>

<**secondaryRef db="intact" dbAc="MI:0469" id="EBI-11508739" refType="identity" refTypeAc="MI:0356"**/>

<**secondaryRef db="imex" dbAc="MI:0670" id="IM-24973" refType="imex-primary" refTypeAc="MI:0662"**/>

</**xref**>

<**attributeList**>

<**attribute name="publication title" nameAc="MI:1091"**>

Structural characterization of human heparanase reveals insights into substrate recognition.

</**attribute**>

<**attribute name="journal" nameAc="MI:0885"**>Nature structural **&amp;** molecular biology</**attribute**>

<**attribute name="publication year" nameAc="MI:0886"**>2015</**attribute**>

<**attribute name="curation depth" nameAc="MI:0955"**>imex curation</**attribute**>

<**attribute name="imex curation" nameAc="MI:0959"**/>

<**attribute name="author-list" nameAc="MI:0636"**>Wu L., Viola CM., Brzozowski AM., Davies GJ.</**attribute**>

<**attribute name="full coverage" nameAc="MI:0957"**>Only protein-protein interactions</**attribute**>

<**attribute name="imex curation" nameAc="MI:0959"**>imex curation</**attribute**>

<**attribute name="contact-email" nameAc="MI:0634"**>gideon.davies@york.ac.uk</**attribute**>

<**attribute name="author-announcement"**>22-Jun-2016: Contacted by IntAct-Help.</**attribute**>

</**attributeList**>

</**bibref**>

<**xref**>

<**primaryRef db="pubmed" dbAc="MI:0446" id="26575439" refType="primary-reference" refTypeAc="MI:0358"**/>

<**secondaryRef db="imex" dbAc="MI:0670" id="IM-24973" refType="imex-primary" refTypeAc="MI:0662"**/>

</**xref**>

<**hostOrganismList**>

<**hostOrganism ncbiTaxId="-1"**>

<**names**>

<**shortLabel**>in vitro</**shortLabel**>

<**fullName**>In vitro</**fullName**>

</**names**>

</**hostOrganism**>

</**hostOrganismList**>

<**interactionDetectionMethod**>

<**names**>

<**shortLabel**>x-ray diffraction</**shortLabel**>

<**fullName**>x-ray crystallography</**fullName**>

<**alias type="go synonym" typeAc="MI:0303"**>X-ray</**alias**>

</**names**>

<**xref**>

<**primaryRef db="psi-mi" dbAc="MI:0488" id="MI:0114" refType="identity" refTypeAc="MI:0356"**/>

<**secondaryRef db="intact" dbAc="MI:0469" id="EBI-1272" refType="identity" refTypeAc="MI:0356"**/>

<**secondaryRef db="pubmed" dbAc="MI:0446" id="14755292" refType="primary-reference" refTypeAc="MI:0358"**/>

</**xref**>

</**interactionDetectionMethod**>

<**participantIdentificationMethod**>

<**names**>

<**shortLabel**>predetermined</**shortLabel**>

<**fullName**>predetermined participant</**fullName**>

<**alias type="synonym" typeAc="MI:1041"**>predetermined</**alias**>

</**names**>

<**xref**>

<**primaryRef db="psi-mi" dbAc="MI:0488" id="MI:0396" refType="identity" refTypeAc="MI:0356"**/>

<**secondaryRef db="intact" dbAc="MI:0469" id="EBI-1465" refType="identity" refTypeAc="MI:0356"**/>

<**secondaryRef db="pubmed" dbAc="MI:0446" id="14755292" refType="primary-reference" refTypeAc="MI:0358"**/>

</**xref**>

</**participantIdentificationMethod**>

<**attributeList**>

<**attribute name="contact-email" nameAc="MI:0634"**>gideon.davies@york.ac.uk</**attribute**>

<**attribute name="journal" nameAc="MI:0885"**>Nature structural **&amp;** molecular biology</**attribute**>

<**attribute name="publication year" nameAc="MI:0886"**>2015</**attribute**>

<**attribute name="curation depth" nameAc="MI:0955"**>imex curation</**attribute**>

<**attribute name="author-list" nameAc="MI:0636"**>Wu L., Viola CM., Brzozowski AM., Davies GJ.</**attribute**>

<**attribute name="full coverage" nameAc="MI:0957"**>Only protein-protein interactions</**attribute**>

<**attribute name="imex curation" nameAc="MI:0959"**>imex curation</**attribute**>

<**attribute name="accepted"**>Accepted 2016-APR-27 AT 12:23 BST AT 12:23 BST by ORCHARD</**attribute**>

<**attribute name="correction comment"**/>

</**attributeList**>

</**experimentDescription**>

</**experimentList**>

<**interactorList**>

<**interactor id="2"**>

<**names**>

<**shortLabel**>q9y251-pro_0000042262</**shortLabel**>

<**fullName**>Heparanase 50 kDa subunit</**fullName**>

<**alias type="gene name" typeAc="MI:0301"**>HPSE</**alias**>

<**alias type="gene name synonym" typeAc="MI:0302"**>HEP</**alias**>

<**alias type="gene name synonym" typeAc="MI:0302"**>HPA</**alias**>

<**alias type="gene name synonym" typeAc="MI:0302"**>HPA1</**alias**>

<**alias type="gene name synonym" typeAc="MI:0302"**>HPR1</**alias**>

<**alias type="gene name synonym" typeAc="MI:0302"**>HPSE1</**alias**>

<**alias type="gene name synonym" typeAc="MI:0302"**>HSE1</**alias**>

<**alias type="gene name synonym" typeAc="MI:0302"**>Endo-glucoronidase</**alias**>

<**alias type="gene name synonym" typeAc="MI:0302"**>Heparanase-1</**alias**>

</**names**>

<**xref**>

<**primaryRef db="uniprotkb" dbAc="MI:0486" id="Q9Y251-PRO_0000042262" refType="identity" refTypeAc="MI:0356"**/>

<**secondaryRef db="intact" dbAc="MI:0469" id="EBI-11600831" refType="identity" refTypeAc="MI:0356"**/>

<**secondaryRef db="intact" dbAc="MI:0469" id="EBI-5453868" refType="chain-parent" refTypeAc="MI:0951"**/>

</**xref**>

<**interactorType**>

<**names**>

<**shortLabel**>protein</**shortLabel**>

<**fullName**>protein</**fullName**>

</**names**>

<**xref**>

<**primaryRef db="psi-mi" dbAc="MI:0488" id="MI:0326" refType="identity" refTypeAc="MI:0356"**/>

<**secondaryRef db="intact" dbAc="MI:0469" id="EBI-619654" refType="identity" refTypeAc="MI:0356"**/>

<**secondaryRef db="pubmed" dbAc="MI:0446" id="14755292" refType="primary-reference" refTypeAc="MI:0358"**/>

<**secondaryRef db="so" dbAc="MI:0601" id="SO:0000358" refType="see-also" refTypeAc="MI:0361"**/>

</**xref**>

</**interactorType**>

<**organism ncbiTaxId="9606"**>

<**names**>

<**shortLabel**>human</**shortLabel**>

<**fullName**>Homo sapiens</**fullName**>

<**alias type="synonym" typeAc="MI:1041"**>Human</**alias**>

</**names**>

</**organism**>

<**sequence**>

KKFKNSTYSRSSVDVLYTFANCSGLDLIFGLNALLRTADLQWNSSNAQLLLDYCSSKGYNISWELGNEPNSFLKKADIFINGSQLGEDFIQLHKLLRKSTFKNAKLYGPDVGQPRRKTAKMLKSFLKAGGEVIDSVTWHHYYLNGRTATKEDFLNPDVLDIFISSVQKVFQVVESTRPGKKVWLGETSSAYGGGAPLLSDTFAAGFMWLDKLGLSARMGIEVVMRQVFFGAGNYHLVDENFDPLPDYWLSLLFKKLVGTKVLMASVQGSKRRKLRVYLHCTNTDNPRYKEGDLTLYAINLHNVTKYLRLPYPFSNKQVDKYLLRPLGPHGLLSKSVQLNGLTLKMVDDQTLPPLMEKPLRPGSSLGLPAFSYSFFVIRNAKVAACI

</**sequence**>

<**attributeList**>

<**attribute name="chain-seq-start"**>158</**attribute**>

<**attribute name="chain-seq-end"**>543</**attribute**>

<**attribute name="crc64"**>3C6EF773788CB1F7</**attribute**>

</**attributeList**>

</**interactor**>

<**interactor id="3"**>

<**names**>

<**shortLabel**>q9y251-pro_0000042260</**shortLabel**>

<**fullName**>Heparanase 8 kDa subunit</**fullName**>

<**alias type="gene name" typeAc="MI:0301"**>HPSE</**alias**>

<**alias type="gene name synonym" typeAc="MI:0302"**>HEP</**alias**>

<**alias type="gene name synonym" typeAc="MI:0302"**>HPA</**alias**>

<**alias type="gene name synonym" typeAc="MI:0302"**>HPA1</**alias**>

<**alias type="gene name synonym" typeAc="MI:0302"**>HPR1</**alias**>

<**alias type="gene name synonym" typeAc="MI:0302"**>HPSE1</**alias**>

<**alias type="gene name synonym" typeAc="MI:0302"**>HSE1</**alias**>

<**alias type="gene name synonym" typeAc="MI:0302"**>Endo-glucoronidase</**alias**>

<**alias type="gene name synonym" typeAc="MI:0302"**>Heparanase-1</**alias**>

</**names**>

<**xref**>

<**primaryRef db="uniprotkb" dbAc="MI:0486" id="Q9Y251-PRO_0000042260" refType="identity" refTypeAc="MI:0356"**/>

<**secondaryRef db="intact" dbAc="MI:0469" id="EBI-11600845" refType="identity" refTypeAc="MI:0356"**/>

<**secondaryRef db="intact" dbAc="MI:0469" id="EBI-5453868" refType="chain-parent" refTypeAc="MI:0951"**/>

</**xref**>

<**interactorType**>

<**names**>

<**shortLabel**>protein</**shortLabel**>

<**fullName**>protein</**fullName**>

</**names**>

<**xref**>

<**primaryRef db="psi-mi" dbAc="MI:0488" id="MI:0326" refType="identity" refTypeAc="MI:0356"**/>

<**secondaryRef db="intact" dbAc="MI:0469" id="EBI-619654" refType="identity" refTypeAc="MI:0356"**/>

<**secondaryRef db="pubmed" dbAc="MI:0446" id="14755292" refType="primary-reference" refTypeAc="MI:0358"**/>

<**secondaryRef db="so" dbAc="MI:0601" id="SO:0000358" refType="see-also" refTypeAc="MI:0361"**/>

</**xref**>

</**interactorType**>

<**organism ncbiTaxId="9606"**>

<**names**>

<**shortLabel**>human</**shortLabel**>

<**fullName**>Homo sapiens</**fullName**>

<**alias type="synonym" typeAc="MI:1041"**>Human</**alias**>

</**names**>

</**organism**>

<**sequence**>QDVVDLDFFTQEPLHLVSPSFLSVTIDANLATDPRFLILLGSPKLRTLARGLSPAYLRFGGTKTDFLIFDPKKE</**sequence**>

<**attributeList**>

<**attribute name="chain-seq-start"**>36</**attribute**>

<**attribute name="chain-seq-end"**>109</**attribute**>

<**attribute name="crc64"**>CCD163EC9D4705B2</**attribute**>

</**attributeList**>

</**interactor**>

<**interactor id="4"**>

<**names**>

<**shortLabel**>alpha-d-glcnac-(1->4)-beta-d-glca-(1->4)-alpha-d-glcnac-(1->4)-beta-d-glca-o-pnp</**shortLabel**>

<**fullName**>alpha-D-GlcNAc-(1->4)-beta-D-GlcA-(1->4)-alpha-D-GlcNAc-(1->4)-beta-D-GlcA-O-pNP</**fullName**>

<**alias type="synonym" typeAc="MI:1041"**>

alpha-D-GlcpNAc-(1->4)-c-GlcpA-(1->4)-alpha-D-GlcpNAc-(1->4)-beta-D-GlcpA-O-pNP

</**alias**>

<**alias type="synonym" typeAc="MI:1041"**>

4-nitrophenyl alpha-D-N-acetylglucosaminyl-(1->4)-->-glucuronosyl-(1->4)-alpha-D-N-acetylglucosaminyl-(1->4)-->-glucuronic acid

</**alias**>

<**alias type="synonym" typeAc="MI:1041"**>GlcNAc-GlcUA-GlcNAc-GlcUA-pNP</**alias**>

<**alias type="synonym" typeAc="MI:1041"**>heparin oligosaccharide dp4, N-acetylated</**alias**>

<**alias type="iupac name" typeAc="MI:2007"**>

4-nitrophenyl 2-acetamido-2-deoxy-alpha-D-glucopyranosyl-(1->4

-->-glucopyranuronosyl-(1->4)-2-acetamido-2-deoxy-alpha-D-glucopyranosyl-(1->4)-->-glucopyranosiduronic acid

</**alias**>

</**names**>

<**xref**>

<**primaryRef db="chebi" dbAc="MI:0474" id="CHEBI:91145" refType="identity" refTypeAc="MI:0356"**/>

<**secondaryRef db="intact" dbAc="MI:0469" id="EBI-11657951" refType="identity" refTypeAc="MI:0356"**/>

</**xref**>

<**interactorType**>

<**names**>

<**shortLabel**>small molecule</**shortLabel**>

<**fullName**>small molecule</**fullName**>

</**names**>

<**xref**>

<**primaryRef db="psi-mi" dbAc="MI:0488" id="MI:0328" refType="identity" refTypeAc="MI:0356"**/>

<**secondaryRef db="intact" dbAc="MI:0469" id="EBI-619656" refType="identity" refTypeAc="MI:0356"**/>

<**secondaryRef db="pubmed" dbAc="MI:0446" id="14755292" refType="primary-reference" refTypeAc="MI:0358"**/>

</**xref**>

</**interactorType**>

<**organism ncbiTaxId="-2"**>

<**names**>

<**shortLabel**>chemical synthesis</**shortLabel**>

<**fullName**>Chemical synthesis (Chemical synthesis)</**fullName**>

</**names**>

</**organism**>

</**interactor**>

</**interactorList**>

<**interactionList**>

<**interaction id="5" imexId="IM-24973-1"**>

<**names**>

<**shortLabel**>hpse_human_1-1</**shortLabel**>

</**names**>

<**xref**>

<**primaryRef db="wwpdb" dbAc="MI:0805" id="5E97" refType="identity" refTypeAc="MI:0356"**/>

<**secondaryRef db="intact" dbAc="MI:0469" id="EBI-11657948" refType="identity" refTypeAc="MI:0356"**/>

<**secondaryRef db="imex" dbAc="MI:0670" id="IM-24973-1" refType="imex-primary" refTypeAc="MI:0662"**/>

</**xref**>

<**experimentList**>

<**experimentRef**>1</**experimentRef**>

</**experimentList**>

<**participantList**>

<**participant id="6"**>

<**interactorRef**>4</**interactorRef**>

<**biologicalRole**>

<**names**>

<**shortLabel**>unspecified role</**shortLabel**>

<**fullName**>unspecified role</**fullName**>

</**names**>

<**xref**>

<**primaryRef db="psi-mi" dbAc="MI:0488" id="MI:0499" refType="identity" refTypeAc="MI:0356"**/>

<**secondaryRef db="intact" dbAc="MI:0469" id="EBI-77781" refType="identity" refTypeAc="MI:0356"**/>

<**secondaryRef db="pubmed" dbAc="MI:0446" id="14755292" refType="primary-reference" refTypeAc="MI:0358"**/>

</**xref**>

</**biologicalRole**>

<**experimentalRoleList**>

<**experimentalRole**>

<**names**>

<**shortLabel**>neutral component</**shortLabel**>

<**fullName**>neutral component</**fullName**>

</**names**>

<**xref**>

<**primaryRef db="psi-mi" dbAc="MI:0488" id="MI:0497" refType="identity" refTypeAc="MI:0356"**/>

<**secondaryRef db="intact" dbAc="MI:0469" id="EBI-55" refType="identity" refTypeAc="MI:0356"**/>

<**secondaryRef db="pubmed" dbAc="MI:0446" id="14755292" refType="primary-reference" refTypeAc="MI:0358"**/>

</**xref**>

</**experimentalRole**>

</**experimentalRoleList**>

</**participant**>

<**participant id="7"**>

<**interactorRef**>3</**interactorRef**>

<**biologicalRole**>

<**names**>

<**shortLabel**>unspecified role</**shortLabel**>

<**fullName**>unspecified role</**fullName**>

</**names**>

<**xref**>

<**primaryRef db="psi-mi" dbAc="MI:0488" id="MI:0499" refType="identity" refTypeAc="MI:0356"**/>

<**secondaryRef db="intact" dbAc="MI:0469" id="EBI-77781" refType="identity" refTypeAc="MI:0356"**/>

<**secondaryRef db="pubmed" dbAc="MI:0446" id="14755292" refType="primary-reference" refTypeAc="MI:0358"**/>

</**xref**>

</**biologicalRole**>

<**experimentalRoleList**>

<**experimentalRole**>

<**names**>

<**shortLabel**>neutral component</**shortLabel**>

<**fullName**>neutral component</**fullName**>

</**names**>

<**xref**>

<**primaryRef db="psi-mi" dbAc="MI:0488" id="MI:0497" refType="identity" refTypeAc="MI:0356"**/>

<**secondaryRef db="intact" dbAc="MI:0469" id="EBI-55" refType="identity" refTypeAc="MI:0356"**/>

<**secondaryRef db="pubmed" dbAc="MI:0446" id="14755292" refType="primary-reference" refTypeAc="MI:0358"**/>

</**xref**>

</**experimentalRole**>

</**experimentalRoleList**>

<**hostOrganismList**>

<**hostOrganism ncbiTaxId="7108"**>

<**names**>

<**shortLabel**>spofr-sf_21</**shortLabel**>

<**fullName**>Spodoptera frugiperda insect cells</**fullName**>

</**names**>

<**cellType**>

<**names**>

<**shortLabel**>sf_21</**shortLabel**>

<**fullName**>Insect cells</**fullName**>

</**names**>

<**xref**>

<**primaryRef db="cabri" dbAc="MI:0246" id="ACC 119" refType="identity" refTypeAc="MI:0356"**/>

<**secondaryRef db="intact" dbAc="MI:0469" id="IA:0072" refType="identity" refTypeAc="MI:0356"**/>

<**secondaryRef db="mint" dbAc="MI:0471" id="MINT-7501077" refType="identity" refTypeAc="MI:0356"**/>

<**secondaryRef db="intact" dbAc="MI:0469" id="EBI-307869" refType="identity" refTypeAc="MI:0356"**/>

</**xref**>

<**attributeList**>

<**attribute name="comment" nameAc="MI:0612"**>

derived from immature ovaries of fall armyworm Spodoptera frugiperda pupae.

</**attribute**>

<**attribute name="comment" nameAc="MI:0612"**>full name: IPLB-SF-21-AE</**attribute**>

<**attribute name="comment" nameAc="MI:0612"**>cells are susceptible to Baculovirus infection.</**attribute**>

</**attributeList**>

</**cellType**>

</**hostOrganism**>

</**hostOrganismList**>

<**stoichiometry value="1"**/>

</**participant**>

<**participant id="8"**>

<**interactorRef**>2</**interactorRef**>

<**biologicalRole**>

<**names**>

<**shortLabel**>unspecified role</**shortLabel**>

<**fullName**>unspecified role</**fullName**>

</**names**>

<**xref**>

<**primaryRef db="psi-mi" dbAc="MI:0488" id="MI:0499" refType="identity" refTypeAc="MI:0356"**/>

<**secondaryRef db="intact" dbAc="MI:0469" id="EBI-77781" refType="identity" refTypeAc="MI:0356"**/>

<**secondaryRef db="pubmed" dbAc="MI:0446" id="14755292" refType="primary-reference" refTypeAc="MI:0358"**/>

</**xref**>

</**biologicalRole**>

<**experimentalRoleList**>

<**experimentalRole**>

<**names**>

<**shortLabel**>neutral component</**shortLabel**>

<**fullName**>neutral component</**fullName**>

</**names**>

<**xref**>

<**primaryRef db="psi-mi" dbAc="MI:0488" id="MI:0497" refType="identity" refTypeAc="MI:0356"**/>

<**secondaryRef db="intact" dbAc="MI:0469" id="EBI-55" refType="identity" refTypeAc="MI:0356"**/>

<**secondaryRef db="pubmed" dbAc="MI:0446" id="14755292" refType="primary-reference" refTypeAc="MI:0358"**/>

</**xref**>

</**experimentalRole**>

</**experimentalRoleList**>

<**featureList**>

<**feature id="9"**>

<**names**>

<**shortLabel**>region</**shortLabel**>

</**names**>

<**xref**>

<**primaryRef db="intact" dbAc="MI:0469" id="EBI-11665904" refType="identity" refTypeAc="MI:0356"**/>

</**xref**>

<**featureType**>

<**names**>

<**shortLabel**>sufficient to bind</**shortLabel**>

<**fullName**>sufficient binding region</**fullName**>

<**alias type="synonym" typeAc="MI:1041"**>sufficient to bind</**alias**>

</**names**>

<**xref**>

<**primaryRef db="psi-mi" dbAc="MI:0488" id="MI:0442" refType="identity" refTypeAc="MI:0356"**/>

<**secondaryRef db="intact" dbAc="MI:0469" id="EBI-608899" refType="identity" refTypeAc="MI:0356"**/>

<**secondaryRef db="pubmed" dbAc="MI:0446" id="14755292" refType="primary-reference" refTypeAc="MI:0358"**/>

</**xref**>

</**featureType**>

<**featureRangeList**>

<**featureRange**>

<**startStatus**>

<**names**>

<**shortLabel**>certain</**shortLabel**>

<**fullName**>certain sequence position</**fullName**>

<**alias type="synonym" typeAc="MI:1041"**>certain</**alias**>

</**names**>

<**xref**>

<**primaryRef db="psi-mi" dbAc="MI:0488" id="MI:0335" refType="identity" refTypeAc="MI:0356"**/>

<**secondaryRef db="intact" dbAc="MI:0469" id="EBI-540564" refType="identity" refTypeAc="MI:0356"**/>

<**secondaryRef db="pubmed" dbAc="MI:0446" id="14755292" refType="primary-reference" refTypeAc="MI:0358"**/>

</**xref**>

</**startStatus**>

<**begin position="2"**/>

<**endStatus**>

<**names**>

<**shortLabel**>certain</**shortLabel**>

<**fullName**>certain sequence position</**fullName**>

<**alias type="synonym" typeAc="MI:1041"**>certain</**alias**>

</**names**>

<**xref**>

<**primaryRef db="psi-mi" dbAc="MI:0488" id="MI:0335" refType="identity" refTypeAc="MI:0356"**/>

<**secondaryRef db="intact" dbAc="MI:0469" id="EBI-540564" refType="identity" refTypeAc="MI:0356"**/>

<**secondaryRef db="pubmed" dbAc="MI:0446" id="14755292" refType="primary-reference" refTypeAc="MI:0358"**/>

</**xref**>

</**endStatus**>

<**end position="386"**/>

</**featureRange**>

</**featureRangeList**>

</**feature**>

</**featureList**>

<**hostOrganismList**>

<**hostOrganism ncbiTaxId="7108"**>

<**names**>

<**shortLabel**>spofr-sf_21</**shortLabel**>

<**fullName**>Spodoptera frugiperda insect cells</**fullName**>

</**names**>

<**cellType**>

<**names**>

<**shortLabel**>sf_21</**shortLabel**>

<**fullName**>Insect cells</**fullName**>

</**names**>

<**xref**>

<**primaryRef db="cabri" dbAc="MI:0246" id="ACC 119" refType="identity" refTypeAc="MI:0356"**/>

<**secondaryRef db="intact" dbAc="MI:0469" id="IA:0072" refType="identity" refTypeAc="MI:0356"**/>

<**secondaryRef db="mint" dbAc="MI:0471" id="MINT-7501077" refType="identity" refTypeAc="MI:0356"**/>

<**secondaryRef db="intact" dbAc="MI:0469" id="EBI-307869" refType="identity" refTypeAc="MI:0356"**/>

</**xref**>

<**attributeList**>

<**attribute name="comment" nameAc="MI:0612"**>

derived from immature ovaries of fall armyworm Spodoptera frugiperda pupae.

</**attribute**>

<**attribute name="comment" nameAc="MI:0612"**>full name: IPLB-SF-21-AE</**attribute**>

<**attribute name="comment" nameAc="MI:0612"**>cells are susceptible to Baculovirus infection.</**attribute**>

</**attributeList**>

</**cellType**>

</**hostOrganism**>

</**hostOrganismList**>

<**stoichiometry value="1"**/>

</**participant**>

</**participantList**>

<**interactionType**>

<**names**>

<**shortLabel**>direct interaction</**shortLabel**>

<**fullName**>direct interaction</**fullName**>

</**names**>

<**xref**>

<**primaryRef db="psi-mi" dbAc="MI:0488" id="MI:0407" refType="identity" refTypeAc="MI:0356"**/>

<**secondaryRef db="intact" dbAc="MI:0469" id="EBI-608833" refType="identity" refTypeAc="MI:0356"**/>

<**secondaryRef db="pubmed" dbAc="MI:0446" id="14755292" refType="primary-reference" refTypeAc="MI:0358"**/>

</**xref**>

</**interactionType**>

<**attributeList**>

<**attribute name="figure legend" nameAc="MI:0599"**>Figure 2b</**attribute**>

<**attribute name="3d-resolution" nameAc="MI:0632"**>1.64 A</**attribute**>

<**attribute name="3d-r-factors" nameAc="MI:0631"**>Rworking 17% Rfree 20%</**attribute**>

</**attributeList**>

</**interaction**>

</**interactionList**>

</**entry**>

</**entrySet**>
